# Streamlining the synthesis of amides using Nickel-based nanocatalysts

**DOI:** 10.1038/s41467-023-40614-1

**Published:** 2023-08-17

**Authors:** Jie Gao, Rui Ma, Fairoosa Poovan, Lan Zhang, Hanan Atia, Narayana V. Kalevaru, Wenjing Sun, Sebastian Wohlrab, Denis A. Chusov, Ning Wang, Rajenahally V. Jagadeesh, Matthias Beller

**Affiliations:** 1 Leibniz-Institut für Katalyse e.V., Albert-Einstein-Street 29a, 18059 Rostock, Germany; 2 Faculty of Environment and Life, Beijing University of Technology, 100124 Beijing, China; 3 Guang-dong Medical University, 523808 Dongguan, China; 4 A. N. Nesmeyanov Institute of Organoelement Compounds, 119991 Moscow, Russia; 5 Nanotechnology Centre, Centre of Energy and Environmental Technologies, VŠB-Technical University of Ostrava, Ostrava-Poruba, Czech Republic

**Keywords:** Homogeneous catalysis, Catalytic mechanisms, Synthetic chemistry methodology

## Abstract

The synthesis of amides is a key technology for the preparation of fine and bulk chemicals in industry, as well as the manufacture of a plethora of daily life products. Furthermore, it constitutes a central bond-forming methodology for organic synthesis and provides the basis for the preparation of numerous biomolecules. Here, we present a robust methodology for amide synthesis compared to traditional amidation reactions: the reductive amidation of esters with nitro compounds under additives-free conditions. In the presence of a specific heterogeneous nickel-based catalyst a wide range of amides bearing different functional groups can be selectively prepared in a more step-economy way compared to previous syntheses. The potential value of this protocol is highlighted by the synthesis of drugs, as well as late-stage modifications of bioactive compounds. Based on control experiments, material characterizations, and DFT computations, we suggest metallic nickel and low-valent Ti-species to be crucial factors that makes this direct amide synthesis possible.

## Abstract (web summary)

The synthesis of amides is a key technology for the preparation of fine and bulk chemicals in industry. Here, the authors present the reductive amidation of esters with nitro compounds under additivesfree conditions as a robust methodology for amide synthesis.

## Introduction

**Fig. 1:**
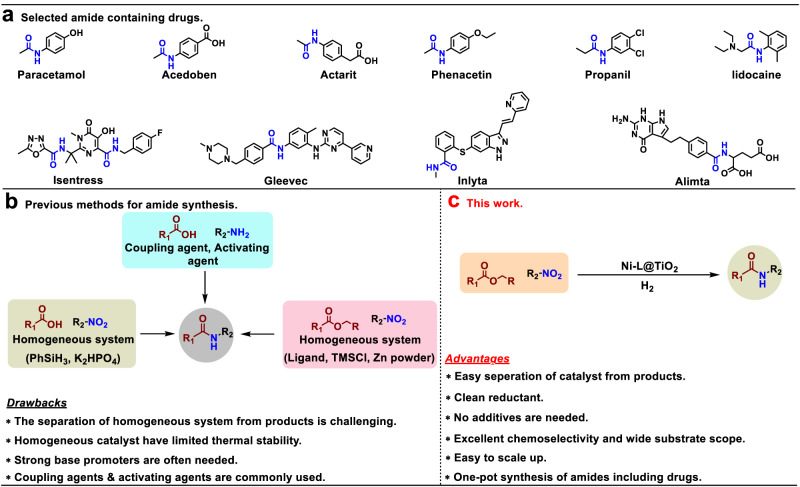
Examples of pharmaceuticals containing amide bonds and selected methodologies for amide synthesis. Selected amide containing drugs (**a**) and methods for the synthesis of amides (**b** and **c**).

Amides are of fundamental importance in chemistry and find a plethora of applications in organic synthesis, medicine, biology as well as material sciences (Fig. 1a)^1,2^. A characteristic feature of many amides is their strong hydrogen bonding ability which determines the structure of biomolecules, especially proteins, but also numerous pharmaceuticals. In fact, 73 of the top 200 selling drugs of the year 2020 in the US are amide derivatives, mainly aromatic ones^3^. Moreover, essential chemicals, building blocks, and advanced materials such as nylon and aramides are aromatic amides^4–6^. Thus, the creation of amide bonds continues to be a highly relevant task in organic and biochemistry and a toolbox of methodologies exist for their preparation^7^.

In general, amide bonds are created through condensation of a carboxylic acid (derivative) and an amine with the release of one equivalent of water^8,9^. To facilitate this process, functionalized amides are typically produced by the reaction of activated carboxylic acid derivatives such as acyl chlorides and anhydrides with amines or direct reaction of carboxylic acids with amines in presence of stoichiometric amounts of coupling reagents (Fig. 1b)^2,10–13^. These classic methods employ stoichiometric quantities of activating reagents, e.g. carbodiimides such as dicyclohexylcarbodiimide (DCC) or (1-ethyl-3-(3-dimethylaminopropyl)carbodiimide (EDC), ammonium or phosphonium salts such as (1-[bis(dimethyllamino)methylene]−1*H*−1,2,3-triazolo[4,5b] pyridinium hexafluorophosphate-3-oxide (HATU), thionyl chloride or *n*-propylphosphonic acid anhydride, which results in the generation of significant amounts of waste^14^. In addition to the cost and toxicity of the activation reagents itself, product purification from the reaction mixture is also a tedious and expensive process^2^. Due to these problems, as well as the importance of the amide bond, the ACS Green Chemistry Institute and members of leading pharmaceutical companies recognized ‘the synthesis of amides by avoiding poor atom economy reagents’ as one of the major challenges in process chemistry of the pharmaceutical industry^15^. Hence, the development of more sustainable methodologies for the synthesis of amides by avoiding stochiometric reagents or activated compounds continues to be important topic both in academia and industry^1^. In this respect, carboxylic esters constitute a promising alternative to the corresponding acids as reaction partner for amide bond formation (Fig. 1b, c)^16^.

Considering that anilines are mainly derived by reduction of nitroarenes, the direct use of nitroarenes for amide synthesis features a clear step-economic advantage. So far, nitroarenes have been rarely used for a direct catalytic synthesis of amides^7,17,18^. As one of the few examples, a homogeneous Ni catalyst has been applied in the presence of stochiometric amounts of Zn/TMSCl. Furthermore, an Ir-Fe-homogeneous photocatalyst system was employed using expensive PhSiH_3_ as reducing agent^19,20^. Considering the current limitations of the desired cascade hydrogenation-amidation reaction, the application of a heterogeneous catalyst would be especially desired^21,22^. Such a methodology has obvious benefits with respect to availability of substrates, cost- and step-economy as well as practicability. Despite the potential advantages, such transformation in the presence of molecular hydrogen remained to the best of our knowledge unexplored^19,20,23,24^.

In the past decade, we prepared a variety of nanostructured 3d metal catalysts supported on carbon^25,26^ or SiO_2_^27,28^ as well as other inorganic oxides. These materials showed excellent performances in various redox transformations. In this context, we became interested to prepare such nanoparticles on TiO_2_, which should result in interesting catalyst materials^29,30^, particularly for valorization of esters^31^. Based on this concept, here, we report a general synthesis protocol for the reductive amidation of nitro compounds and esters using an inexpensive heterogeneous catalyst in the presence of hydrogen. Key for this transformation is the synergistic effect between metallic nickel and low-valent Ti-species, which allows for the preparation of functionalized fine chemicals and structurally complex amides including important drug molecules.

## Results

### Catalyst synthesis

**Fig. 2:**
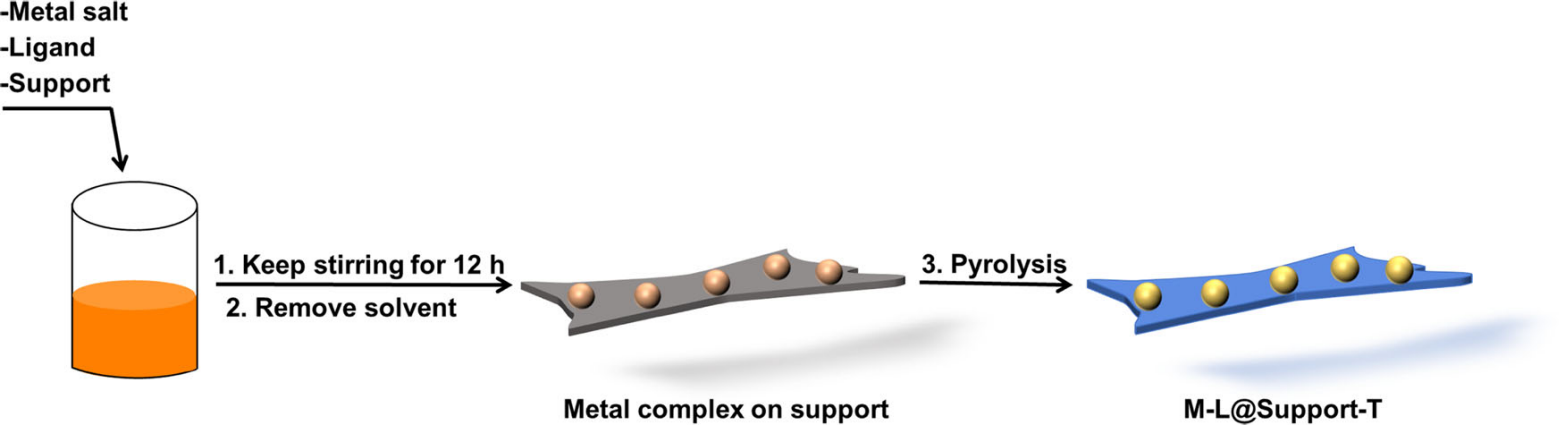
Catalysts preparation. Pictorial representation for the synthesis of catalytic materials.

To realize the reductive amidation of nitro compounds with esters in a straightforward manner, we planned to use a suitable multifunctional catalyst^32^. In this transformation, the initial hydrogenation steps as well as the subsequent amide formation should proceed efficiently to avoid unwanted side reactions^33^. Ideally, the catalyst material should be based on inexpensive and stable supports as well as 3d-metals, due to their inherent advantages (availability, toxicity, price, robustness, recyclability)^34–36^. In the past decade, some of us developed a general approach for the synthesis of such nanoparticles on inert supports and stable supported single atom catalysts^25,26,37–39^. Based on these previous works, we prepared Fe-, Co-, and Ni-nanoparticles in the presence of inexpensive aniline ligands (o-phenylenediamine L1, p-phenylenediamine L2, aniline L3), which were supported on TiO_2_, γ-Al_2_O_3_, SiO_2_ and Vulcan carbon. In a typical procedure, the respective metal nitrates were mixed in situ with the ligand in methanol and then immobilized on a given heterogeneous support. Finally, the immobilized materials were pyrolyzed at given temperatures under argon atmosphere to obtain supported nanoparticles-based catalysts (Fig. 2). The obtained materials are labelled as M-L@Suppport-T, where M, L, T denotes metal, ligand, and pyrolysis temperature.

### Reaction design and catalyst evaluation

All the catalytic materials were tested for the reductive amidation of 4-nitrophenol in ethyl acetate to give 4-acetaminophen (paracetamol). The target product is one of the most common medications against fever and mild to moderate pain. Thus, it is an active ingredient of a wide range of cold and flu remedies. The currently used processes for paracetamol preparation are shown in Supplementary Fig. 1. As shown in Table 1, a selection of Fe-, Ni-, and Co-based nanoparticles on different supports were prepared in the presence of L1. The performance of all these materials was compared in the model reaction under industrially relevant conditions (20 bar H_2_, 130 °C). Noteworthy, no additional solvent or sophisticated activation reagents, etc. were included in this reaction system. In the presence of most catalytic materials, mixtures of **3** and **4** were obtained (Table 1, entries 1–17). Nevertheless, depending on the support and the metal source the individual reaction steps can be controlled. For example, the carbon supported material only promoted hydrogenation of the nitroarene to 4-hydroxyaniline **3** (Table 1, entry 1).

**Table 1:** Synthesis of 4-acetaminophen (paracetamol) from nitrophenol and ethyl acetate: Catalytic activities and selectivities


Entry	Catalyst	Metal loading^a^ (wt %)	Conversion (%)	Yield (%)	
				3	4
1	Ni-L1@Carbon-800	4.76	>99	97	<1
2	Ni-L1@SiO_2_-800	4.68	>99	33	65
3	Ni-L1@g-Al_2_O_3_-800	4.85	>99	48	50
4	Ni-L1@TiO_2_-800	4.78	>99	13	85
5	Fe-L1@TiO_2_-800	4.83	17	4	12
6	Co-L1@TiO_2_−800	4.62	>99	45	53
7	Ni-L2@TiO_2_-800	4.81	>99	21	77
8	Ni-L3@TiO_2_-800	4.65	>99	19	79
9	Ni@TiO_2_-800	4.66	<1	<1	<1
10	Ni(NO_3_)_2_·6H_2_O@TiO_2_	4.80	<1	<1	<1
11	Ni(NO_3_)_2_·6H_2_O + L1	4.80	<1	<1	<1
12	Ni-L1@TiO_2_−1000	4.81	30	14	15
13	Ni-L1@TiO_2_-600	4.79	>99	21	76
14	Ni-L1@TiO_2_−400	4.83	63	46	16
15^b^	Ni-L1@TiO_2_-800	4.78	>99	10	88
16^c^	Ni-L1@TiO_2_-800	4.78	>99	<1	52
17^d^	Ni_3_S_2_		<1	<1	<1


Reaction conditions: 0.5 mmol 4-nitrophenol, 1 mL ethyl acetate, 60 mg catalyst (10 mol% Ni), 20 bar H_2_, 130 °C, 24 h. Yields were determined by GC using *n*-hexadecane as the standard.

^a^Metal loading is determined by ICP-OES analysis.

^b^Reaction conditions: 0.5 mmol 4-nitrophenol, 1 mL ethyl acetate, 0.5 mL H_2_O, 60 mg catalyst, 20 bar CO, 130 °C, 24 h.

^c^Reaction conditions: 0.5 mmol 4-nitrophenol, 0.3 mL formic acid, 1 mL ethyl acetate, 60 mg catalyst, 20 bar N_2_, 130 °C, 24 h.

^d^0.5 mmol 4-nitrophenol, 1 mL ethyl acetate, 4 mg Ni_3_S_2_ (10 mol% Ni), 20 bar H_2_, 130 °C, 24 h.

Comparing the activity of the different supports for the desired tandem transformation, SiO_2_- and γ-Al_2_O_3_-supported nickel catalysts were less selective than Ni-L1@TiO_2_−800 (Table 1, entries 2–4), which provided the best result towards paracetamol **4**. Among the tested supported metals, Fe-L1@TiO_2_−800 showed little activity (Table 1, entry 5), while Co-L1@TiO_2_−800 and Ni-L1@TiO_2_−800 gave the desired product 4 in 53% and 85% yield, respectively (Table 1, entry 6 vs 4). Interestingly, using other simple anilines (L2, L3) as modifiers for the preparation of the corresponding materials also produced active catalysts, while the material prepared in the absence of the ligand (Ni@TiO_2_−800) and the non-pyrolyzed materials were completely inactive for the benchmark reaction (Table 1, entries 9–11). Optimization of the temperature (400–1000 °C) of the pyrolysis process also revealed a strong influence of this parameter (Table 1, entries 4, 12–14)^40^.

The optimal supported nickel catalyst system not only allows to perform the reductive amidation of **1** in the presence of hydrogen, but also permits the desired reaction under transfer hydrogenation conditions in the presence of formic acid (FA)^41^ or applying CO as reductant in presence of water^42^ (Water-Gas Shift reaction), which shows the tolerance (robustness) of this system against acid, CO, and H_2_O. Thus, paracetamol **4** is obtained in 52% and 88% yield, respectively (Table 1, entries 15–16). However, in the presence of FA, the N-formylation product of 4-nitrophenol was formed as a side-product in 48% yield^41^.

Next, we examined the stability and recycling of the optimal catalyst in the benchmark reaction. As shown in Supplementary Fig. 2, the material is stable for 7th reaction cycles and only after that a significant decrease of activity was observed. For comparison, the remaining mass of catalyst after each cycle is shown in Supplementary Table 1. ICP-OES analysis of the reaction mixture confirmed no significant leaching of nickel species (detection limit: 0.03 wt%). Consequently, the observed productivity loss cannot be an effect of nickel leaching. TEM characterization of the recycled catalyst (after 8th run) shows that the average size of nickel nanoparticles is significantly increased to 24.2 nm (Supplementary Fig. 3a). Hence, we attribute the productivity loss to the aggregation of nickel nanoparticles.

### Catalyst characterization

**Fig. 3:**
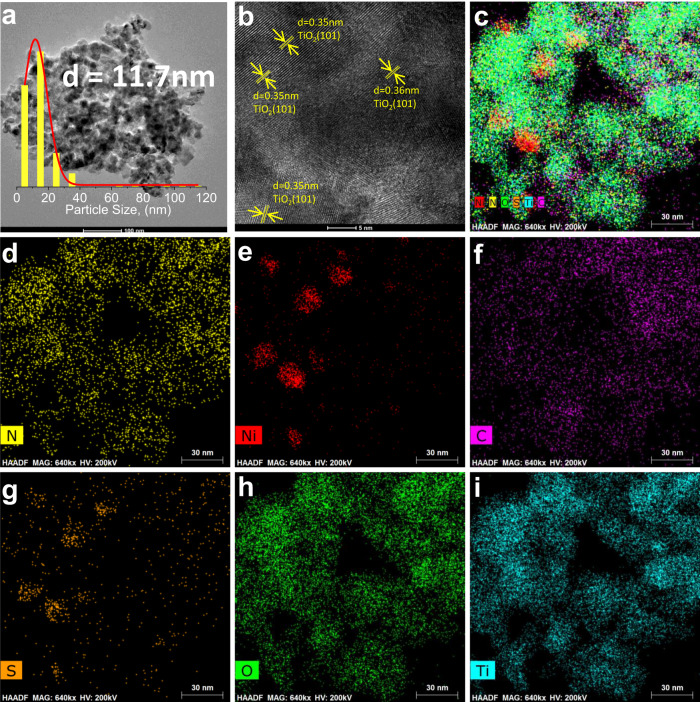
Electron microscopy analysis of Ni-L1@TiO_2_-800. **a**, **b** TEM images. In (**a**) it is shown that the particles are distributed on the support with an average diameter of 11.7 nm. The particle size distribution is obtained by measuring more than 200 particles. In (**b**) the lattice spacings are assigned to (101) plane of anatase TiO_2_. **c** EDS elemental mapping image, and (**d**–**i**) the visualization of elements: (**d**) N, (**e**) Ni, (**f**) C, (**g**) S, (**h**) O, (**i**) Ti.

To understand the structure differences of Ni-L@TiO_2_-T samples, synthesized materials have been characterized by the means of TEM, XRD, XPS, TPD and BET. According to the TEM images, nickel particles were uniformly distributed on the titania surface (Fig. 3a and Supplementary Figs. 4–9). Ni particle size distributions are displayed in Fig. 3a (average size of 11.7 nm). The lattice spacings of 0.35 nm were assigned to (101) plane of anatase TiO_2_ (Fig. 3b)^43^. In this case EDS mapping images showed an element distribution of N, Ni, C, S, O, and Ti (Fig. 3c–i). Notably, the TiO_2_ support was prepared via sulphate process. Hence, the sulphur content is 2.3 wt%, and Ni_3_S_2_ was detected by XRD in all these samples, too (Supplementary Figs. 10a, 11). TEM images confirmed this observation (Fig. 3e, g). Interestingly, commercially available Ni_3_S_2_ is inactive for the model reaction (Table 1, entry 17), implying a synergistic effect of all constituents in the as-prepared material.

**Fig. 4:**
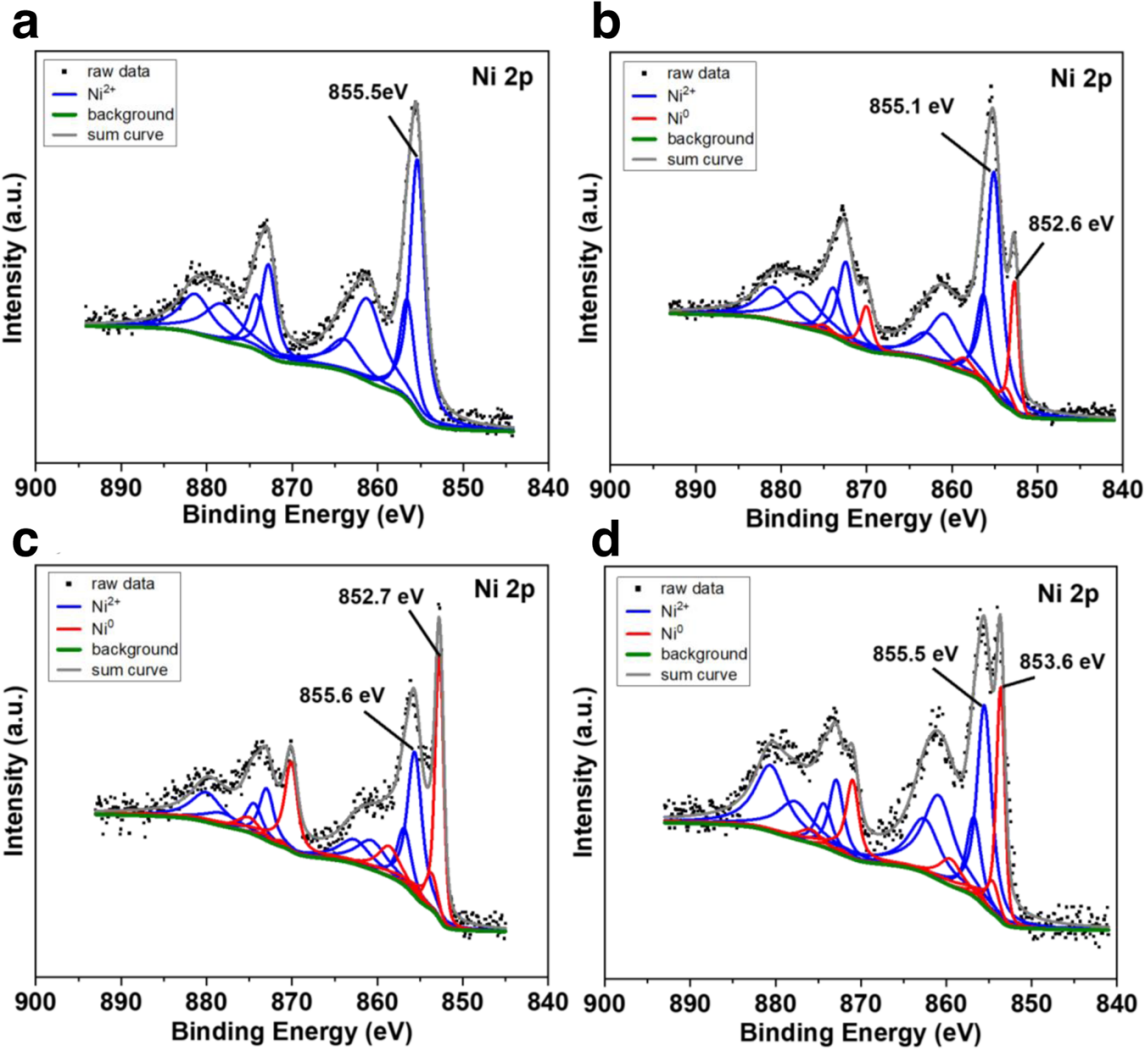
XPS Ni 2p spectra of different catalysts. (**a**) Ni-L1@TiO_2_-400, (**b**) Ni-L1@TiO_2_−600, (**c**) Ni-L1@TiO_2_−800 and (**d**) Ni-L1@TiO_2_−1000.

In XRD measurements, the metallic nickel diffraction peak is observed at 44°, 52° and 76° in Ni-L1@TiO_2_-800 and Ni-L1@TiO_2_-1000, while Ni-L1@TiO_2_-400 and Ni-L1@TiO_2_-600 did not show this pattern (Supplementary Fig. 10a)^44^. In Ni-L1@TiO_2_-400, Ni-L1@TiO_2_-600, and Ni-L1@TiO_2_-800, the original titania anatase phase prevailed, while phases including rutile TiO_2_, Ti_4_O_7_ and Ti_6_O_11_ are found in Ni-L1@TiO_2_-1000 (Supplementary Fig. 10a)^45,46^. Further, we performed XRD characterizations of Ni@TiO_2_-800, Ni-L1@TiO_2_-800, Ni-L2@TiO_2_-800, and Ni-L3@TiO_2_-800. The XRD results of these samples demonstrated that pyrolysis of the ligated nickel species promoted formation of metallic nickel, while nickel exists mainly as Ni_3_S_2_ and NiTiO_3_ in Ni@TiO_2_-800 (Supplementary Fig. 11). Materials prepared in the presence of ligands are more active compared with Ni@TiO_2_-800. XRD of the recycled catalyst (after the 8th run) proved the aggregation of nickel nanoparticles as the peak intensity of nickel is relatively increased (Supplementary Fig. 3b). Next, XPS characterizations were conducted to understand the surface properties of these materials. The sample surface mainly consists of C, N, O, Ni, Ti, and S which confirms the STEM results (Supplementary Table 6) and the surface area of the studied materials is in the range of 25–160 m^2^/g (Supplementary Fig. 15a). It is worth mentioning that the surface concentration of Ni decreases with pyrolysis temperature from 2.7 to 1.1 at.% from Ni-L1@TiO_2_-400 to Ni-L1@TiO_2_-1000 (Supplementary Table 6). Figure 4 shows the Ni 2p spectra of the Ni-L1@TiO_2_ samples with pyrolysis temperatures 400, 600, 800, and 1000 C. The Ni 2p spectra of Ni-L1@TiO_2_-400 in Fig. 4a shows the main Ni 2p_3/2_ peak at 855.5 eV and strong satellite features at higher binding energies, thus suggesting an oxidation state of +2 for Ni, probably as Ni(OH)_2_ or NiOOH^47,48^. At higher pyrolysis temperatures a rather sharp peak at lower binding energies around 852.7 eV appears which can be identified as metallic Ni^48^. Assuming a combination of Ni^0^ and Ni^2+^ species for deconvolution, the relative Ni^0^ concentration at the surface can be calculated and reaches its maximum with about 43% for Ni-L1@TiO_2_-800 (Supplementary Fig. 12). Note that different satellite peak hights might reflect different Ni^2+^ species possibly present at the surface but cannot be distinguished here. Furthermore, all tested materials showed very similar Ti 2p spectra (Supplementary Fig. 13) typical for Ti^4+^^49^. The N 1 s spectra in Supplementary Fig. 14 is deconvoluted with four peaks indicating the presence of pyridinic N (398.8 eV), pyrrolic N (400.5 eV), graphitic N (401.4 eV), and pyridinic N-oxide (403.3 eV) on all samples^50,51^. However, the ratio of the different nitrogen species changes significantly with the pyrolysis temperature. In detail, the maximum pyrrolic N content (77%) is observed in Ni-L1@TiO_2_-400, while for Ni-L1@TiO_2_-600 the highest pyridinic N content (56%) is observed.

To address the effect of metallic nickel on the hydrogenation activity of the identified catalyst systems, we also performed the reduction of 4-nitrophenol. The best yield of 4-aminophenol is observed in the presence of Ni-L1@TiO_2_-800 (Supplementary Table 2). Further, we conducted CO_2_-TPD and NH_3_-TPD of the respective catalysts to identify the distributions of basic and acidic sites necessary for the second amidation reaction step (Supplementary Fig. 16). Notably, for Ni-L1@TiO_2_-800 more (weak) acidic sites (Supplementary Fig. 16a, NH_3_ desorbed between 50–100 °C) were identified compared to Ni-L1@TiO_2_-600. To proof whether the identified acidic sites facilitate the formation of paracetamol, we further conducted the reaction of 4-aminophenol with ethyl acetate (Supplementary Table 3). As shown in Supplementary Table 3, Ni-L1@TiO_2_-800 afforded a 47% yield of paracetamol, while Ni-L1@TiO_2_-600 only gave 34%. Thus, both Ni^0^ and weak acidic sites on the support material are advantageous for the overall catalyst activity.

### Mechanistic investigations

**Fig. 5:**
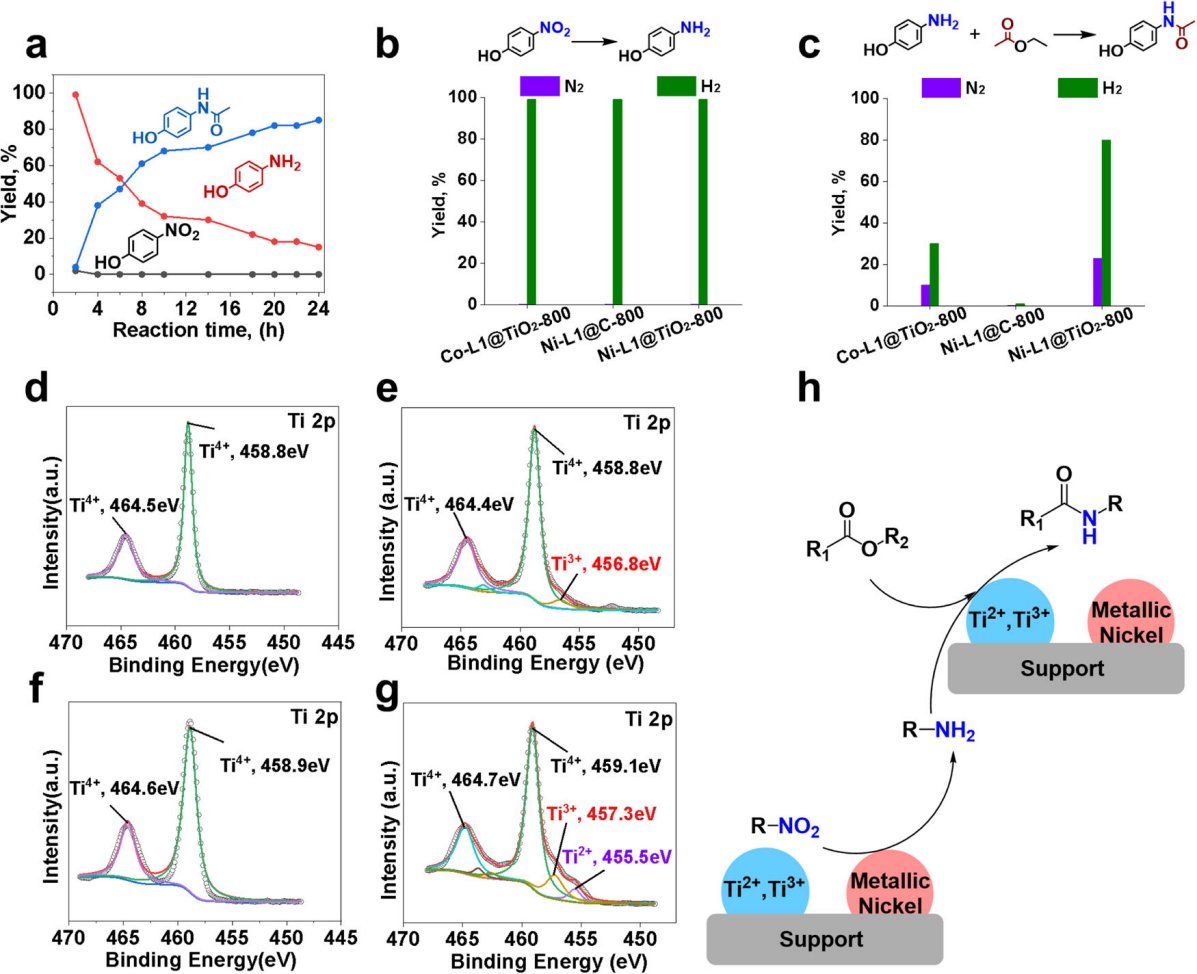
Mechanistic investigations. **a** Kinetic studies. Reaction conditions: 0.5 mmol 4-nitrophenol, 1 mL ethyl acetate, 60 mg Ni-L1@TiO_2_−800, 20 bar H_2_, 130 °C, 24 h. **b**, **c** Control experiments. Reaction conditions: (**b**) 0.5 mmol 4-nitrophenol, 1 mL ethanol, 60 mg Ni-L1@TiO_2_−800, 20 bar H_2_, 130 °C, 24 h. (**c**) 0.5 mmol 4-aminophenol, 1 mL ethyl acetate, 60 mg Ni-L1@TiO_2_−800, 20 bar H_2_, 130 °C, 17 h. In situ XPS characterization was performed at 130 °C under 1 bar N_2_ (**d**, **f**) and H_2_ (**e**, **g**) for 1 h, (**d**, **e**) Ti 2p for Co-L1@TiO_2_−800, (**f**, **g**) Ti 2p for Ni-L1@TiO_2_−800. (**h**) Proposed reaction pathway.

Apparently, the direct synthesis of paracetamol proceeds first by hydrogenation of **1** to give **3** and subsequent formation of amide **4**. Indeed, as shown in the kinetic profile in Fig. 5a, the hydrogenation step in the presence of the optimal catalyst is finished within 4 h (99% conversion). Amide formation is slower, starts only after 5 h and needs >20 h to be completed. To understand the influence of the catalyst material on both reaction steps, several control experiments were performed (Fig. 5b, c). As shown below the hydrogenation of 4-nitrophenol to 4-aminophenol proceeds under the standard conditions with nearly quantitative yield in the presence of Co-L1@TiO_2_-800, Ni-L1@C-800 and Ni-L1@TiO_2_-800 (Fig. 5b). Obviously, such hydrogenations easily take place with different types of supported 3d metal nanoparticles^52^. More interestingly, the amidation of 4-aminophenol with ethyl acetate only occurred with Co-L1@TiO_2_-800 and Ni-L1@TiO_2_-800, while Ni-L1@C-800 did not show any reactivity (Fig. 5c). Using the titania supported nickel catalyst several observations are interesting: under nitrogen atmosphere the target product is afforded in 23% yield (Fig. 5c), while surprisingly the paracetamol yield is increased to 80% under H_2_ atmosphere. A similar behaviour is observed for the reaction of nitrobenzene to give acetanilide (Supplementary Fig. 17). Compared with Co-L1@TiO_2_-800, Ni-L1@TiO_2_-800 is more active in the amidation reaction of ethyl acetate with 4-aminophenol (Fig. 5c). To better understand this observation, in situ XPS characterization experiments of Co-L1@TiO_2_-800 and Ni-L1@TiO_2_-800 catalysts in presence of nitrogen or hydrogen were performed using a separate reaction cell in the XPS apparatus (Fig. 5d–g, Supplementary Fig. 18). In presence of H_2_ at 130 °C, Ni^2+^ is reduced to metallic Ni as shown by the strong increase of the Ni^0^ peak together with the decrease of Ni^2+^ intensity (Supplementary Fig. 18). Surprisingly, in addition to Ti^4+^ titanium species Ti^3+^ and Ti^2+^ with a binding energy at ~457 eV and ~455 eV are observed after H_2_ treatment (Fig. 5e, g)^53^. Compared with Co-L1@TiO_2_-800, more Ti^2+^ and Ti^3+^ species are found in Ni-L1@TiO_2_-800 (Fig. 5e, g). In contrast, a clear Ti^4+^ XPS spectrum after a treatment in N_2_ atmosphere at 130 °C is observed (Fig. 5d, f). The above analysis indicates that metallic nickel particles are responsible for the reduction of the nitro group to the corresponding aniline^54^. In addition, the subsequent amidation step is also positively influenced by the Ni nanoparticles and low-valent Ti-species. Hence, we propose the reduction of the nitro compound by metallic nickel in presence of hydrogen, subsequently aniline will react with the ester to produce the desired amide facilitated by the support (Fig. 5h).

**Fig. 6:**
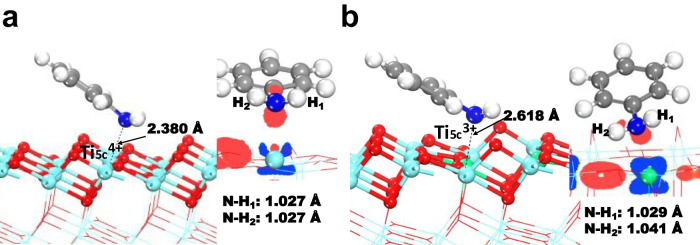
The most stable configuration of aniline adsorbed on anatase-TiO_2_ (101) surface. **a** aniline is interacting with a Ti^4+^ ion, and (**b**) aniline is interacting with a Ti^3+^ ion in anatase-TiO_2_ (101) surfaces. The inset was the different charge density of the complex. Blue areas represent electron depletion and red areas means electron accumulation with an isosurface value of 0.1 electrons/Å^3^.

To understand the role of low-valent Ti-species in the second amidation step, we performed also DFT computations (Fig. 6, details are shown in Supplementary Information). These calculations showed that the most stable configuration of aniline adsorbs on the top of a 5-fold coordinated Ti^4+^ atom (denoted as Ti_5c_^4+^, Fig. 6a) and a 5-fold coordinated Ti^3+^ atom (denoted as Ti_5c_^3+^, Fig. 6b) sites of anatase-TiO_2_ (101) surfaces. In both adsorbed systems, the N atom is bonded on the top of the Ti atom. The accumulation of electron density between N and Ti atom suggests the formation of a N–Ti bond in the two adsorbed systems. The E_ads_ of aniline molecule on Ti_5c_^4+^ was calculated to be −0.959 eV and the Ti–N bond length was 2.380 Å. The bond length of two N–H bonds were elongated from 1.018 Å in an original aniline molecule to 1.027 Å in the chemisorbed state. The E_ads_ of aniline adsorbed on the top of Ti_5c_^3+^ was calculated to be −1.153 eV. This suggests that aniline interacts thermodynamically more favorable with the computed Ti^3+^ species compared to the corresponding Ti^4+^ species. The distance of Ti_5c_^3+^–N was 2.618 Å and the NH_2_ groups in the aniline molecule are slightly leaned to an adjacent bridge oxygen (O_b_) on the anatase-TiO_2_ (101) surfaces. The hydrogen-bond interactions between O_b_ and H in the NH_2_ group leading to one of the N–H bonds was elongated to 1.041 and 1.029 Å, respectively. Apparently, the longer bond length of N–H bonds in aniline/ Ti^3+^ suggests that the interaction of aniline with Ti^3+^ promotes the activation and weaken of N–H bonds more effectively than the interaction with the respective Ti^4+^ system.

Next, we aimed to test the general applicability of Ni-L1@TiO_2_-800 in various amide syntheses. As shown from the kinetic experiment (Fig. 5a), the second step is relatively slower compared with the nitro reduction. Thus, in order to balance the catalyst loading and reaction rate, we had the idea to speed up the rate determining amidation step by adding cheaper titania support. Indeed, even at low metal catalyst loading in the presence of titania quantitative yield of the desired product can be achieved (Supplementary Fig 19). To further demonstrate the role of the support in this transformation, we pyrolyzed freshly prepared TiO_2_ at 400–1000 °C and the resulting five materials were tested in the reaction of 4-aminophenol and ethyl acetate under standard conditions. The following order of activity is observed: fresh TiO_2_ > TiO_2_-400 > TiO_2_-600 > TiO_2_-800 > TiO_2_-1000 (Supplementary Fig. 20b). Next, all these samples were characterized by XRD (Supplementary Fig. 10b), BET (Supplementary Fig. 15b), and NH_3_-TPD (Supplementary Fig. 20a). In general, the specific surface area and the number of acidic sites of these TiO_2_ samples decreased significantly with increasing pyrolysis temperature, which also correlates well with the product yield (Supplementary Figs. 15b, 20b). Hence, we believe that both the number of acidic sites and the surface area of TiO_2_ are the major factors that affect the amidation process in this model reaction.

### Ni-catalyzed synthesis of amides

**Fig. 7:**
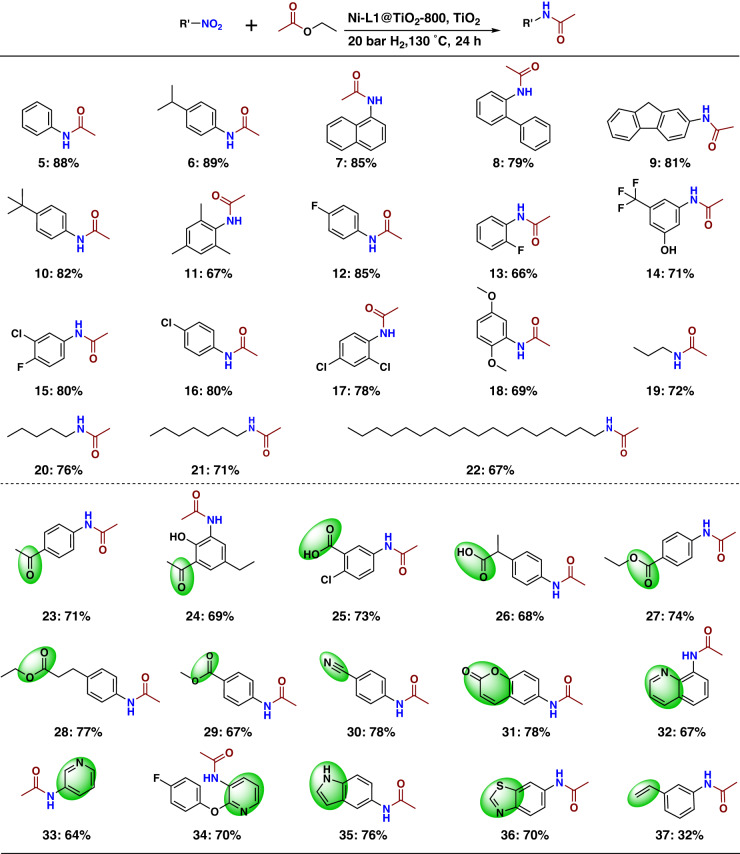
Ni-L1@TiO_2_−800 catalysed synthesis of amides: scope of nitro compounds. Reaction conditions: 0.5 mmol nitro compound, 1 mL ethyl acetate, 30 mg Ni-L1@TiO_2_−800, 20 mg TiO_2_, 20 bar H_2_, 130 °C, 24 h. Isolated yields based on nitro compound.

With an active material for the straight synthesis of paracetamol in hand, we investigated its applicability for other substrates under a standardized set of conditions (30 mg Ni-L1@TiO_2_-800, 20 mg TiO_2_, 20 bar H_2_, 130 °C, 24 h). Diverse nitroarenes with electron-donating (**6**–**11**) or electron-withdrawing groups (**12**–**18**) underwent reductive amidation with ethyl acetate and provided the corresponding amides in up to 89% isolated yield (Fig. 7). Halogenated nitroarenes are well tolerated and produced the corresponding amides (**12**–**17**) without noticeable dehalogenation side reactions. Interestingly, aliphatic nitro compounds, e.g. 1-nitropropane and 1-nitrooctadecane are also converted into amides (**19**–**22**) in 67–72% yield.

For the application of any synthetic methodology, specifically for advanced organic synthesis, it is essential to achieve a high degree of chemoselectivity and functional group tolerance. In this regard, we performed reductive amidation of many functionalized nitroarenes. As shown in Fig. 7, the presented catalytic system tolerates well other potentially reducible groups like ketones (**23**–**24**), carboxylic acids and esters (**25**–**29**), nitrile (**30**), and vinyl substituted derivatives (**37**). In addition, heterocycles (**31**–**36**) were successfully reacted with ethyl acetate and afforded the desired amides in up to 78% yield.

**Fig. 8:**
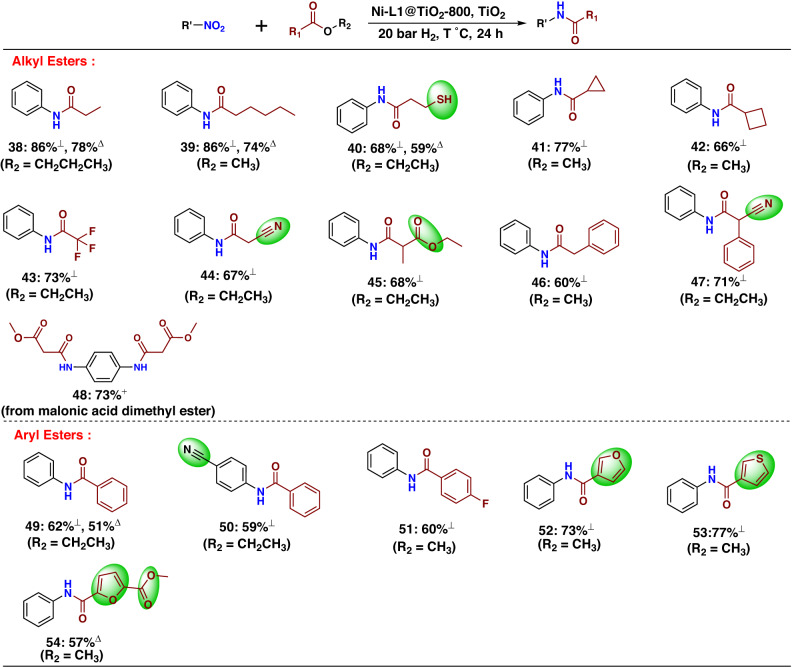
Ni-L1@TiO_2_−800 catalysed synthesis of amides: scope of esters. ^⊥^Reaction conditions: 0.5 mmol nitro compound, 1 mL ester, 30 mg Ni-L1@TiO_2_−800, 20 mg TiO_2_, 20 bar H_2_, 130 °C, 24 h. ^Δ^Reaction conditions: 0.5 mmol nitro compound, 2 mmol ester, 1 mL toluene, 30 mg Ni-L1@TiO_2_−800, 20 mg TiO_2_, 20 bar H_2_, 140 °C, 24 h. ^+^Reaction conditions: 0.5 mmol 4-nitroaniline, 1 mL ester, 30 mg Ni-L1@TiO_2_-800, 20 mg TiO_2_, 20 bar H_2_, 130 °C, 48 h. All are isolated yields based on nitro compound. The detailed information of these esters is provided in Supplementary Fig. 21.

Next, we tested the coupling of different esters (Fig. 8). Apart from ethyl acetate various other alkyl esters including fluorinated ones reacted smoothly to give the corresponding amides (**38**–**48**). Notably, functional groups such as sulfhydryl and nitrile were well tolerated, too (**40**, **44**, **47**, **50**). Interestingly, two carboxylic diesters reacted selectively to provide the mono-amide derivatives (**45**, **54**). Utilizing 4-nitroaniline it is also possible to obtain the corresponding diamide, which is interesting for the preparation of polyamides (**48**). To show that the reductive amidation is possible without excess of ester, we performed further test reactions in solvents and varied the ratio between nitro compound and ester (Supplementary Table 4). As shown in Supplementary Table 4, utilizing toluene as solvent gave the best results, and the optimal ratio between nitro compound and ester is 1:4. This variation of reaction conditions was tested with five selected substrates (**38**–**40**, **49**, **54**) to demonstrate the general applicability. In all cases, the corresponding amides were produced with good yield.

**Fig. 9:**
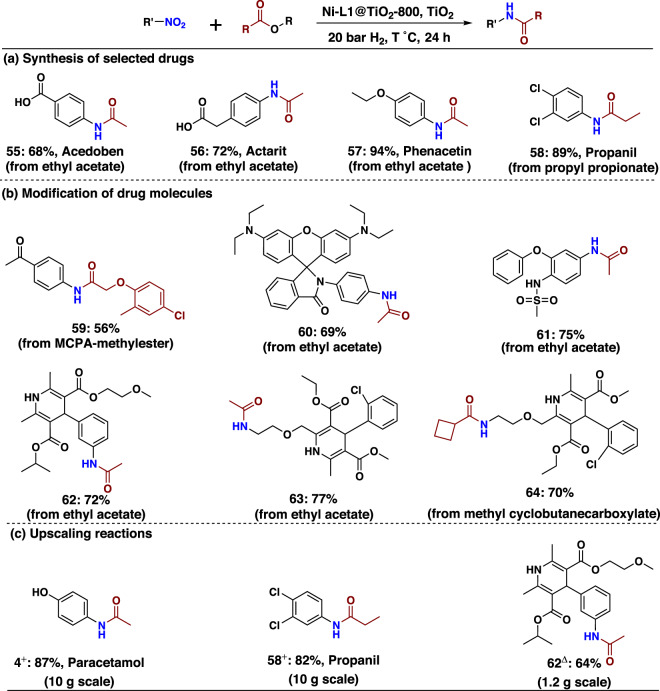
Applications of nickel-catalysed amidation. **a** Synthesis of selected drugs. Reaction conditions: 0.5 mmol nitro compound, 1 mL ester, 30 mg Ni-L1@TiO_2_-800, 20 mg TiO_2_, 20 bar H_2_, 130 °C, 24 h. Isolated yields based on nitro compound. **b** Amidation of nitro and ester containing drugs. Reaction conditions: 0.5 mmol nitro compound, 1 mL ester, 30 mg Ni-L1@TiO_2_-800, 20 mg TiO_2_, 20 bar H_2_, 130 °C, 24 h. Isolated yield based on nitro compound. **c** Scale-up reactions. ^+^Reaction conditions: 10 g nitro compound, 30 mL ethyl acetate, required amount of catalyst and TiO_2_ (30 mg of Ni-L1@TiO_2_-800 and 20 mg TiO_2_ for each 0.5 mmol nitro compound), 20 bar H_2_, 130 °C, 24 h. ^Δ^1.2 g nitro compound, 4 mL ethyl acetate, required amount of catalyst and TiO_2_ (30 mg of Ni-L1@TiO_2_-800 and 20 mg TiO_2_ for each 0.5 mmol nitro compound), 20 bar H_2_, 130 °C, 24 h. Isolated yields based on nitro compound. The detailed information of these esters is provided in Supplementary Fig. 22.

To showcase the utility of this synthetic protocol, we carried out the preparation of selected drug molecules (Fig. 9a). Specifically, Acedoben (**55**), Actarit (**56**), Phenacetin (**57**), and Propanil (**58**) are produced smoothly without further optimization. From a synthetic point of view, late-stage modification of bioactive compounds is interesting, too (Fig. 9b). Hence, MCPA-methylester, a common herbicide, was converted directly to amide (**59**). Similarly, rhodamine fluorescent probe^55^ can be successfully transformed into the corresponding amide (**60**). Likewise, amidation of nimesulide, a non-steroidal anti-inflammatory drug (NSAID), and nimodipine afforded the desired products in 72–75% yield (**61**–**62**). Finally, we performed upscaling reactions of selected products on 1–10 g scale (Fig. 9c). No significant deviations of the products yields were observed compared to the 0.5 mmol-scale experiments.

## Discussions

In conclusion, a practical reductive amidation methodology has been developed for the direct synthesis of amides from nitro compounds and esters using nickel-based nanostructured catalyst. Direct cascade transformation of nitro compounds to amides was realized utilizing molecular hydrogen as green reductant in the presence of a heterogeneous catalyst and to the best of our knowledge no previous examples exist. The presented catalytic material showed excellent substrate scope and high functional group tolerance. The utility of this methodology is demonstrated by a more efficient synthesis of industrially relevant fine chemicals and selected drug molecules including paracetamol. Further, the method allows for fast and clean modifications of many bioactive compounds. The utility of the catalyst material is also demonstrated by gram-scale reactions and recycling experiments. By performing control experiments, material characterizations, and DFT computation, we found metallic nickel and low-valent Ti-species are two crucial factors that allow for this straightforward amide synthesis. We envision that the established methodology will inspire other scientists both in industry and academia to develop even more efficient amide syntheses.

## Methods

### Materials and methods

All esters and nitro compounds were obtained commercially from various chemical companies and used directly without further purification. Nickel(II) nitrate hexahydrate [Ni(NO_3_)_2_·6H_2_O, cat no. 203874-20 G], cobalt(II) nitrate hexahydrate [Co(NO_3_)_2_·6H_2_O, cat no. 239267-5 G] and iron(III) nitrate nonahydrate [Fe(NO_3_)_3_·9H_2_O, cat no. 216828-100 G] were purchased from Sigma Aldrich. Ni_3_S_2_ (CAS No.12035-72-2, Lot: C12Y018) was obtained from Thermo Fischer Scientific. Ethyl acetate (HPLC grade, cat no. 5582024) and toluene (HPLC grade, cat no. 10109731) were obtained from Thermo Fisher Scientific. Carbon powder, VULCAN® XC72R with Code XVC72R and CAS No. 1333-86-4 was obtained from Cabot Corporation Prod. The catalyst support γ-Al_2_O_3_ (cat no. 199443-100 G) and SiO_2_ (cat no. 85356-100 G) were purchased from Sigma Aldrich. The pyrolysis experiments were performed using the tube furnace (Bayferrox® 110, LANXESS, GmbH).

XRD powder pattern were recorded on a Panalytical X’Pert θ/2θ -diffractometer equipped with Xcelerator detector using automatic divergence slits and Cu kα1/α2 radiation (40 kV, 40 mA; λ = 0.15406 nm, 0.154443 nm). Cu beta-radiation was excluded using a nickel filter foil. Measurements were performed with either 0.021 or 0.005°s^−1^. Finely pestled samples were mounted on silicon zero background holders. After data collection obtained intensities were converted from automatic to fixed divergence slits (0.25°) for further analysis. Peak positions and profile were fitted with Pseudo-Voigt function using the HighScore Plus software package (Panalytical). Phase identification was done by using the PDF-2 database of the International Center of Diffraction Data (ICDD).

The XPS measurements were performed on an ESCALAB 220iXL (ThermoFischer Scientific) with monochromated Al Kα radiation (E = 1486.6 eV). Samples are prepared on a stainless-steel holder with conductive double-sided adhesive carbon tape. The electron binding energies were obtained without charge compensation leading a main C 1 s peak at around 284.6 eV. For quantitative analysis the peaks were deconvoluted with Gaussian-Lorentzian curves using the software Unifit 2021, the peak areas were divided by the transmission function of the spectrometer and the element specific sensitivity factor of Scofield. To determine the relative Ni^0^ content the Ni 2p spectra are deconvoluted using an experimentally gained peak shape of metallic Ni from a sputter cleaned Ni foil together with peaks corresponding to Ni^2+^.

In-situ XPS was conducted using a Thermofischer ESCALAB 250Xi X-ray photoelectron spectrometer with a pass energy of 40.00 eV and an Al Kα excitation source (hv = 1253.6 eV). The analysis chamber pressure is 8*10^−10^Pa. The Operating Voltage is 12.5 kV. The filament current is 16 mA, and the signal is accumulated 5–10 times. The step size is 0.1 eV. To characterize the effect of gas on the sample structure, the catalyst was placed in a reaction chamber connected to the XPS equipment and exposed to H_2_ or N_2_ (20 mL/min) at room temperature. Then, once the chamber is heated to 130 °C with 10 °C/min, the sample is treated for 1 h. Afterward, the treated sample was directly transferred into the analysis chamber in vacuum (8 × 10^−10^Pa) to avoid exposure to air.

The TEM measurement, the high-angle annular dark-field scanning transmission electron microscopic (HAADF-STEM) images were collected on a FEI Talos F200x S/TEM instrument at 300 kV accelerating voltage. HAADF-STEM images were acquired using a HAADF detector. To prepare the TEM samples, an appropriate amount of sample powder was dispersed in ethanol and then dropped on a 3 mm TEM Cu grid. The images of energy dispersive spectroscopy (EDS) elemental mapping in the STEM mode were obtained from the Titan electron microscope using SuperX system.

Surface area and porosity are carried out by the N_2_ adsorption isotherm using the Brunauer-Emmett-Teller (BET) method on an ASAP 2020 Micromeritics instrument. Before analysis, all samples are degassed at 200 °C for 6 h to desorb moisture and impurities from their surfaces.

NH_3_-TPD and CO_2_-TPD measurements were done using a Micromeritics Autochem II 2910 instrument. A 150 mg sample was loaded in U shaped quartz reactor and heated from RT (room temperature) to 150 °C with 10 K/min in He (50 mL/min) to remove adsorbed water from the surface of the solid material. The temperature remained constant for 60 min, then cooled down to 100 °C in a flow of He (50 mL/min). Following, the sample was exposed to 1% NH_3_ or 5% CO_2_ in He (50 mL/min) for 60 min at 100 °C, followed by removal of physisorbed NH_3_ or CO_2_ by flushing with He (50 mL/min) for 60 min at 100 °C. The temperature was decreased for 70 °C for 10 min. The sample was then ramped to 650 °C at a heating rate of 10 K/min in flowing of He (50 mL/min). The temperature was held at 650 °C for 60 min. The analysis of the effluent gases was performed with Quadrupol mass spectrometer (Balzers Omnistar).

NMR spectra were recorded on Bruker AV 300 and 400 spectrometers. Chemical shifts are reported in parts per million relatives to CDCl_3_ (7.26 and 77.16 ppm for ^1^H and ^13^C respectively) or DMSO-d6 (2.50 and 39.52 ppm for ^1^H and ^13^C respectively). Chemical shifts δ are reported in ppm relative to the solvent resonance signal as an internal standard. The following abbreviations were used to designate chemical shift multiplicities: s = singlet, d = doublet, t = triplet, q = quartet, sept = septet, m = multiplet, br = broad; coupling constants are given in Hertz (Hz).

HRMS data were recorded on (1) ESI-HRMS: HPLC System 1200 /ESI-TOF-MS 6210 (Agilent) and (2) EI-HRMS: Mass Spectrometer MAT 95XP (Thermo Electron), 70 eV. GC-MS was performed on an ISQ Trace 1300 in the electron ionization (EI) mode.

GC analyses was performed on an Agilent 7890 A instrument (Column: Agilent 19091J-413: 30 m × 320 μm × 0.25 μm, carrier gas: H_2_, FID detection.

Details of DFT computations can be found in Supplementary Information. DFT Computational details.

All catalytic reactions were carried out in 300- and 100-mL autoclaves (PARR Instrument Company).

### Preparation of catalytic materials

#### Preparation of TiO_2_

TiO_2_ slurry/gel prepared via sulfate process was commercially obtained from Kronos International, Inc., Research and Development, Germany and labelled as GL102/10826. The following procedure has been used to prepare final form of TiO_2_ powder with significantly high surface area: The TiO_2_ gel was added to distilled water and the dispersed solution was stirred for 20 min. Then, the mixture was allowed to settle for overnight or for several hours and then the upper layer of water was decanted. This procedure was repeated for 3 to 4 times until almost all soluble components in the slurry were removed by washing. Next, the resulting slurry was centrifuged for 15 min at a speed of 4240 rpm on Heraus instrument, Model: Sepatech 6000, Germany to obtain the solid material, which was then oven dried for 72 h (at 100 °C/24 h, at 150 °C/24 h and at 200 °C/24 h). The obtained TiO_2_ clumps were powdered to get fine powder. The surface area of TiO_2_ (oven dried at 200 °C) was found to be 295–315 m^2^/g. In addition, the preparation of TiO2 support is reproducible and the elemental analysis result is show in Supplementary Table 5.

#### Preparation of Ni-L1@TiO_2_-800 and other catalytic materials

In a 50 mL round bottomed flask, 576 mg Ni(NO_3_)_2_•6H_2_O and 300 mg ligand (L1, o-phenylenediamine) were stirred in 50 mL methanol at room temperature for 5 min. Then 2 g of TiO_2_ support was added and stirring was continued for 12 h. Then, the methanol was removed by rotary evaporation and the solid material was dried in oven at 60 °C. The resulting material was grounded to a fine powder and transferred to quartz boat. The material was placed in tube furnace and pyrolyzed at 800 °C for 2 h under argon with a heating rate of 5 °C/min and then tube furnace was cooled to room temperature. After cooling the furnace to room temperature, the catalytic material was taken out from the furnace and stored in glass vial. In addition, we repeated the preparation of Ni-L1@TiO_2_-800 and its catalytic performance is reproducible (see Supplementary Table 7).

Similar procedure was applied for the preparation of other materials such as Fe-L1@TiO_2_-800, Co-L1@TiO_2_-800, Ni-L1@C-800, Ni-L1@SiO_2_-800 and Ni-L1@γ-Al_2_O_3_-800.

Note: Elemental analysis of ligands, Ni(NO_3_)_2_•6H_2_O, TiO_2_ and Ni-L1@TiO_2_-800 are shown in Supplementary Table 5.

### General procedure for the amidation of nitro compounds and esters

#### Solvent free condition

A magnetic stirring bar and 0.5 mmol nitro compound were transferred to 4 mL reaction vial and then 1 mL ethyl acetate or respective ester was added. Then, 30 mg catalyst (Ni-L1@TiO_2_-800) and 20 mg TiO_2_ were added, and the vial was fitted with septum, cap, and needle. Next, reaction vials (9 vials with different substrates at a time) were placed into a 300 mL autoclave. The autoclave was flushed with hydrogen for two times with 20 bar and then it was pressurized with 20 bar H_2_. The autoclave was placed into an aluminium block preheated at 150–160 °C (placed 30 min before counting the reaction time to attain reaction temperature) and the reactions were stirred for required time. During the reaction the inside temperature of the autoclave was measured to be 130 °C–140 °C and this temperature was used as the reaction temperature. After the completion of the reactions, the autoclave was cooled to room temperature. The remaining H_2_ gas was discharged slowly, and the reaction mixture were taken out from the autoclave. The reaction mixture was analysed by GC and GC-MS. The corresponding products were purified by column chromatography (silica, *n*-hexane, ethyl acetate). The purified products were analysed by NMR (^1^H, ^13^C), HRMS, and GC-MS.

#### Using toluene as solvent

For the reactions conducted in toluene solvent, similar experimental procedure was applied with 0.5 mmol nitro compound, 2 mmol corresponding ester and 1 mL toluene.

#### Scale-up reactions

10-gram scale reactions were performed in Teflon fitted 300 mL autoclave. 10 g of respective nitro compound and 30 mL ethyl acetate were added to the Teflon fitted 300 mL autoclave and then required amount of Ni-L1@TiO_2_-800 catalyst and TiO_2_ (30 mg of Ni-L1@TiO_2_-800 and 20 mg TiO_2_ for each 0.5 mmol nitro compound). The autoclave was flushed with hydrogen for two times and then it was pressurized with 20 bar H_2_. The autoclave was placed into an aluminium block preheated at the desired temperatures (placed 20 min before counting the reaction time to attain reaction temperature) and the reaction was stirred for required time. After the completion of the reactions, the autoclave was cooled to room temperature. The H_2_ gas was released slowly, and the reaction mixture was taken out from the autoclave and analysed by GC and GC-MS. The corresponding products were purified by column chromatography (silica, *n*-hexane, ethyl acetate). The purified products were analysed by NMR (^1^H, ^13^C) and HRMS.

Note: for 1.2 g scale reaction carried out in 100 mL autoclave and similar procedure mentioned above has been applied.

#### Catalyst recycling

A 4 mL dried glass vial was charged with 0.5 mmol 4-nitrophenol and magnetic stirring bar. Then, 1 mL ethyl acetate was added followed by the addition of 60 mg Ni-L1@TiO_2_-800. Then, the vial was fitted with septum, cap and needle and placed into a 300 mL autoclave. Then autoclave was flushed with 20 bar H_2_ for two times, and it was pressured with 20 bar H_2_. The autoclave was placed into an aluminium block preheated at 150 °C (placed 30 min before counting the reaction time to attain reaction temperature) and the reactions were stirred for required time. During the reaction the inside temperature of the autoclave was measured to be 130 °C and this temperature was used as the reaction temperature. After the completion of the reactions, the autoclave was cooled to room temperature. The H_2_ gas was discharged slowly, and the reaction vials were taken out from the autoclave. The catalyst was separated by centrifugation and washed with ethel acetate. Then the recycled catalyst was dried and used for the next run without any reactivation.

### Supplementary information

Supplementary Information

## Data Availability

The data that support the findings of this study are available from the corresponding authors (M.B. and R.V.J.) upon request.
