# Supplementary material for: Streamlining the synthesis of amides using Nickel-based nanocatalysts
Source: Nat Commun. 2023 Aug 17;14:5013. doi: 10.1038/s41467-023-40614-1 (PMC10435480; doi:10.1038/s41467-023-40614-1)
Supplement: Supplementary file 1 — Supplementary Information [file 41467_2023_40614_MOESM1_ESM.pdf]

Supplementary Information

Streamlining the Synthesis of Amides using Nickel-based Nanocatalysts

Jie Gao,[1] Rui Ma,[1] Fairoosa Poovan,[1] Lan Zhang,[2] Hanan Atia,[1] Narayana V. Kalevaru,[1] Wenjing Sun,[3] Sebastian Wohlrab,[1] Denis A. Chusov,[4,*] Ning Wang,[2,*] Rajenahally V. Jagadeesh,[1,5*] Matthias Beller[1,*]

[1]Leibniz-Institut für Katalyse e.V., Albert-Einstein-Street 29a, 18059 Rostock, Germany.

[2]Faculty of Environment and Life, Beijing University of Technology, 100124 Beijing, China.

[3]Guang-dong Medical University, 523808 Dongguan, China.

[4]A. N. Nesmeyanov Institute of Organoelement Compounds, 119991 Moscow, Russia.

[5]Nanotechnology Centre, Centre for Energy and Environmental Technologies, VŠB-Technical University of Ostrava, Ostrava-Poruba, Czech Republic.

*Corresponding authors. Email: denis.chusov@gmail.com, ning.wang.1@bjut.edu.cn, jagadeesh.rajenahally@catalysis.de, matthias.beller@catalysis.de

Contents:

S1. Supplementary Tables……………………………………………………….........2

S2. Supplementary Figures…………………………………………………………..4

S3. NMR and HRMS Data………………………………………………………......23

S4. NMR Spectra………………………………………………………………….....40

S5. HRMS Data……….……………………………………………………............101

S6. References…………………………………………………………………...115

S1. Supplementary Tables.

Supplementary Table 1. Weight of catalyst after each recycling experiment.

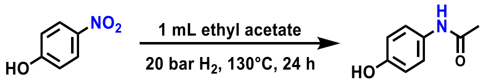

Recycle time	Weight of catalyst (mg)
0	60
1	60
2	59
3	58
4	58
5	57
6	56
7	56
8	55

Reaction conditions: 0.5 mmol 4-nitrophenol, 1 mL ethyl acetate, 20 bar H_2, 60 mg Ni-L1@TiO_2-800, 130 °C, 24 h.

Supplementary Table 2. Hydrogenation of 4-nitrophenol to 4-aminophenol using Ni-L1@TiO_2-400-1000.

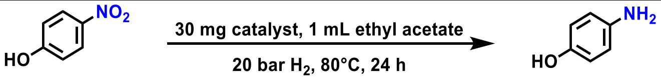

Entry	Catalyst	Conversion (%)	Yield (%)
1	Ni-L1@TiO_2-400	trace	trace
2	Ni-L1@TiO_2-600	31	29
3	Ni-L1@TiO_2-800	37	35
4	Ni-L1@TiO_2-1000	trace	trace

Reaction conditions: 0.5 mmol 4-nitrophenol, 1 mL ethyl acetate, 30 mg catalyst, 20 bar H_2, 80 °C, 24 h. Conversion and yields were determined by GC using *n*-hexadecane as the standard.

Supplementary Table 3. Amidation of 4-aminophenol to Paracetamol using Ni-L1@TiO_2-400-1000.

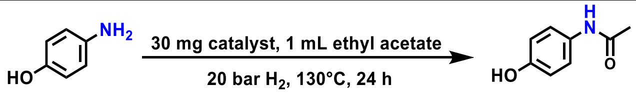

Entry	Catalyst	Conversion (%)	Yield(%)
1	Ni-L1@TiO_2-400	64	62
2	Ni-L1@TiO_2-600	35	34
3	Ni-L1@TiO_2-800	50	47
4	Ni-L1@TiO_2-1000	17	14

Reaction conditions: 0.5 mmol 4-aminophenol, 1 mL ethyl acetate, 30 mg catalyst, 20 bar H_2, 130 °C, 24 h. Conversion and yields were determined by GC using *n*-hexadecane as the standard.

Supplementary Table 4. Screening of reaction parameters for the benchmark reaction.

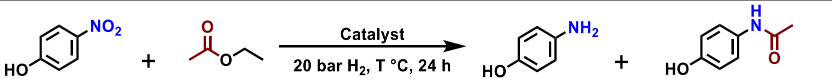

1 EA 2 3 (Drug: Paracetamol)

Entry	Solvent	Nitrophenol (mmol)	EA (mmol)	Temperature	Conversion of 1, %	Yield of 2, %	Yield of 3, %
1	THF	0.5	0.5	130 °C	63	63	0
2	Toluene	0.5	0.5	130 °C	55	26	29
3	Methanol	0.5	0.5	130 °C	100	100	0
4	Ethanol	0.5	0.5	130 °C	100	100	0
5	Isopropanol	0.5	0.5	130 °C	98	98	0
6	n-hexane	0.5	0.5	130 °C	16	4	12
7	Toluene	0.5	1.0	130 °C	100	60	40
8	Toluene	0.5	2.0	130 °C	100	44	56
9	Toluene	0.5	2.0	140 °C	100	30	70

Reaction conditions: 30 mg Ni-L1@TiO_2-800, 20 mg TiO_2, 20 bar H_2, 1 mL solvent, 130-140 °C, 24 h. Conversion and yields were determined by GC using *n*-hexadecane as the standard.

Supplementary Table 5. Element analysis (EA) of catalyst materials and precursors.

Entry	Sample	Element, wt%			
		C	N	H	S
1	L1	65.2	24.9	7.1	0
2	L2	66.6	24.5	7.6	0
3	L3	51.3	4.3	8.8	0
4	$Ni(NO_3)_2 \cdot 6H_2O$	0.1	3.2	7.7	0
5	TiO_2	0.1	0.1	0.5	2.3
6	Ni-L1@TiO_2-800	6.6	0.4	0.5	1.1

Supplementary Table 6. Elemental surface composition of pyrolyzed materials from XPS.

Entry	Sample	Element, at%						
		C	N	Ni	O	S	Si	Ti
1	Ni-L1@TiO_2-400	45.3	6.6	2.7	32.5	1.1	-	11.9
2	Ni-L1@TiO_2-600	43.0	4.7	2.2	34.3	1.2	0.6	14.1
3	Ni-L1@TiO_2-800	54.4	3.4	1.5	27.5	2.0	0.6	10.7
4	Ni-L1@TiO_2-1000	63.0	0.8	1.1	24.4	0.3	0.7	9.6

Supplementary Table 7. Reproducibility of catalytic activity of Ni-L1@TiO_2-800.

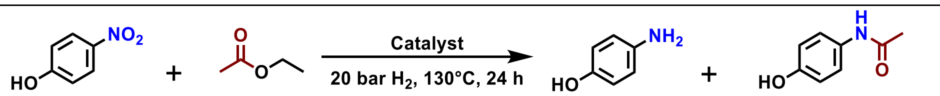

1 2 3 4 (Drug: Paracetamol)

Entry	Catalyst	Conversion (%)	Yield (%)	
			3	4
1	Ni-L1@TiO_2-800	>99	15	85
2	Ni-L1@TiO_2-800	>99	13	87
2	Ni-L1@TiO_2-800	>99	15	84

Reaction conditions: 0.5 mmol 4-nitrophenol, 1 mL ethyl acetate, 60 mg catalyst (10 mol% Ni), 20 bar H_2, 130 °C, 24 h. Conversion and yields were determined by GC using *n*-hexadecane as the standard.

S2. Supplementary Figures.

S2.1. General methods for the preparation of Paracetamol.

The industrial process for the preparation of paracetamol involves two steps: first, 4-nitrophenol is hydrogenated to 4-aminophenol in the presence of Raney nickel or suitable catalyst and then acetylation of 4-aminophenol with acetic anhydride takes place (Supplementary Fig. 1a). An alternative multi-step synthesis was developed at Celanese, which involves the direct acylation of phenol with acetic anhydride in the presence of hydrogen fluoride and then conversion of the resulting ketone to the corresponding ketoxime with hydroxylamine, followed by acid-catalysed Beckmann rearrangement (Supplementary Fig. 1b).

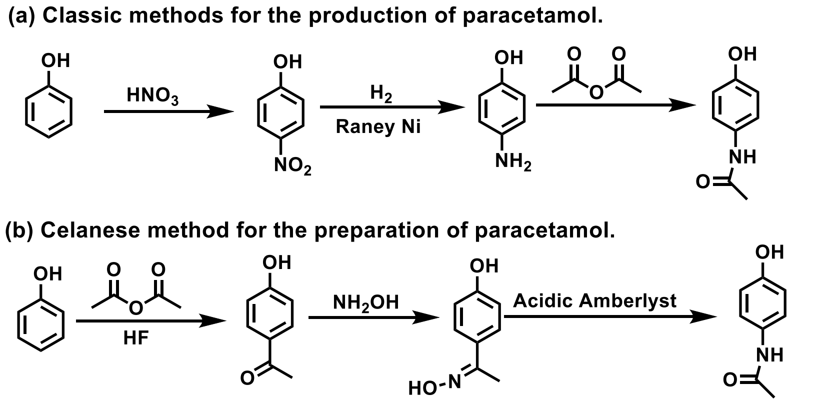

Supplementary Figure 1. General methods for the preparation of Paracetamol.(a) Raney Ni catalyzed amidation reaction. (b) Celanese method for the preparation of paracetamol.

S2.2. Recycling experiments.

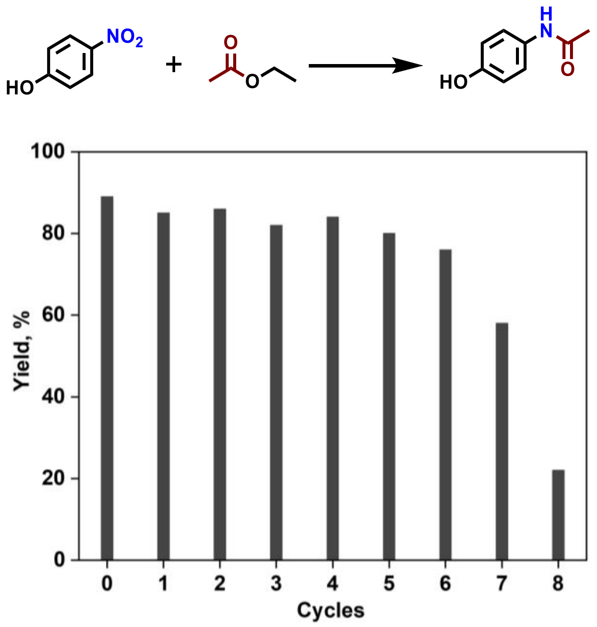

Supplementary Figure 2. **Recycling of Ni-L1@TiO2-800 for the synthesis of paracetamol**. Reaction conditions: 0.5 mmol 4-nitropheneol, 1 mL ethyl acetate, 20 bar H_2, 60 mg Ni-L1@TiO_2-800, 130 °C, 24 h. Conversions and yields were determined by GC using n-hexadecane as standard.

S2.3. Characterization of catalytic materials.

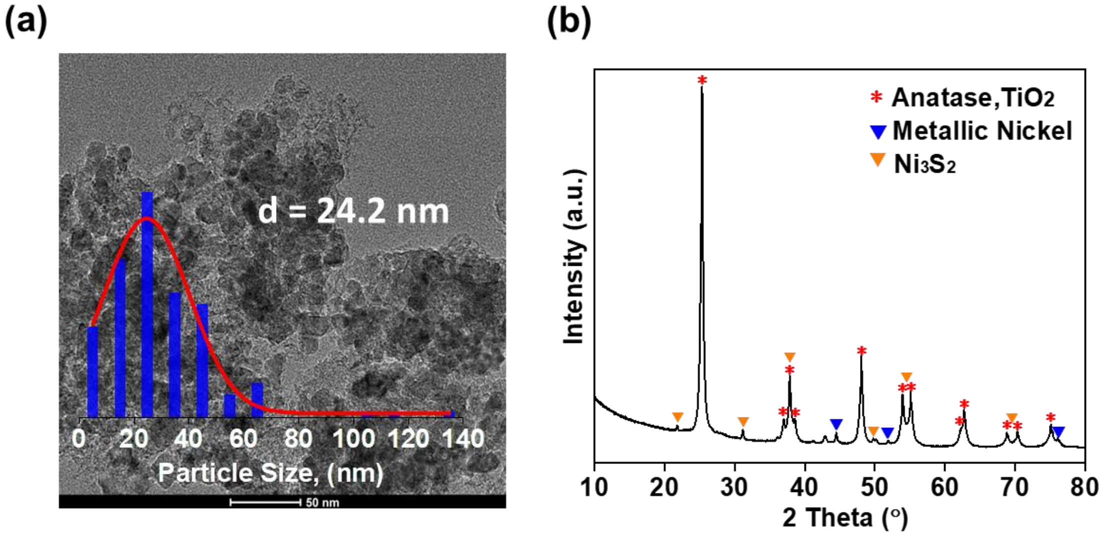

Supplementary Figure 3. TEM images and XRD pattens. (a) TEM image and (b) XRD patterns of recycled Ni-L1@TiO_2-800. The inset of (a) shows that the particles are distributed on the support with an average diameter of 24.2 nm.

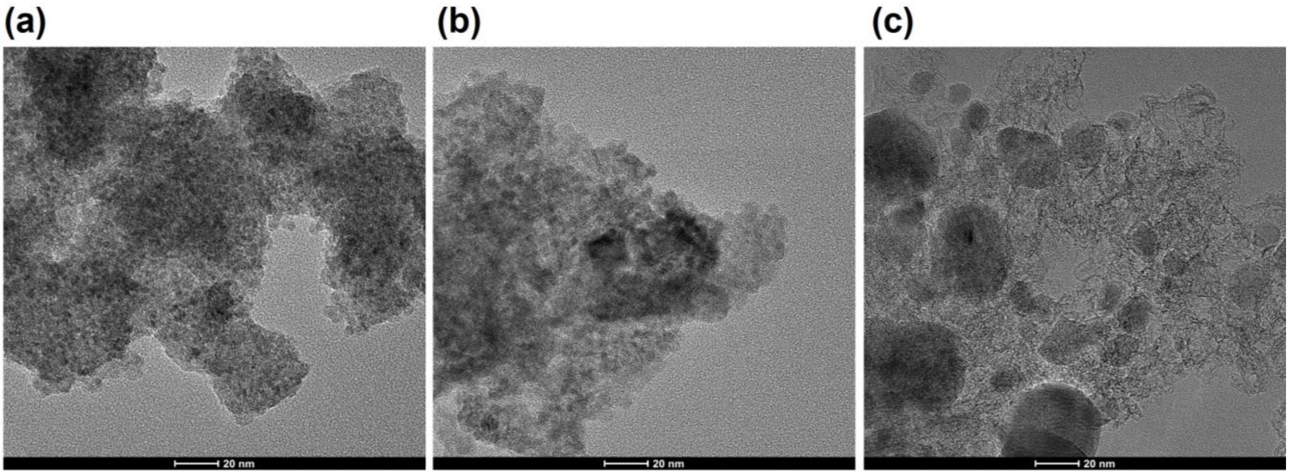

Supplementary Figure 4. TEM images. (a) Ni-L1@TiO_2-400, (b) Ni-L1@TiO_2-600 and (c) Ni-L1@TiO_2-1000.

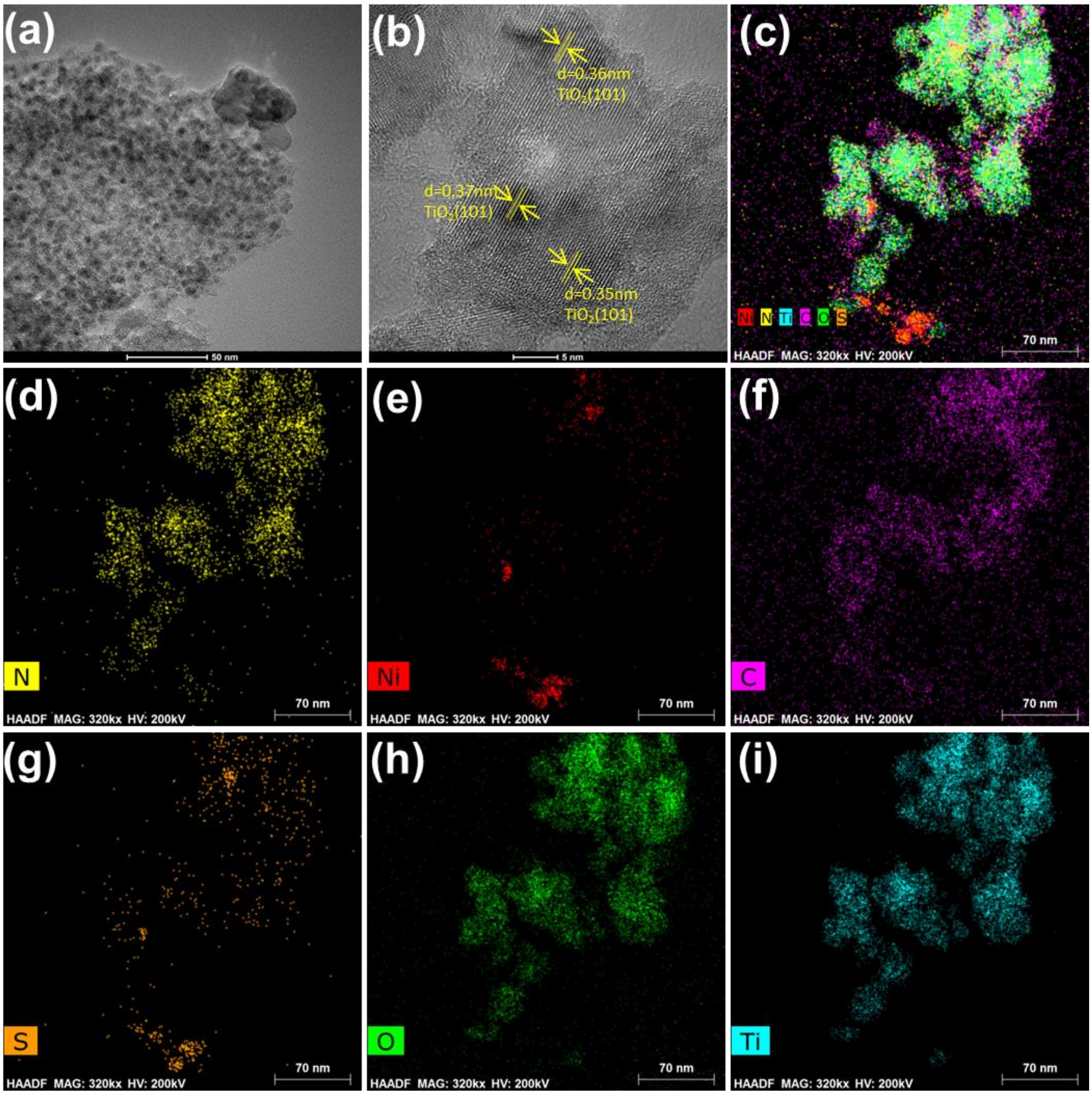

Supplementary Figure 5. TEM images of Ni-L2@TiO_2-800 (a). TiO_2 (101) facet with lattice spacings of 0.35 nm (b) were observed. The EDS-mapping of the Ni-L2@TiO_2-800 catalyst (c) showed homogeneous dispersion of C (f), N (d), O (h), and Ti (i) species. The distribution of Ni (e) is consistent with that of S (g), indicating the existing of Ni_3S_2.

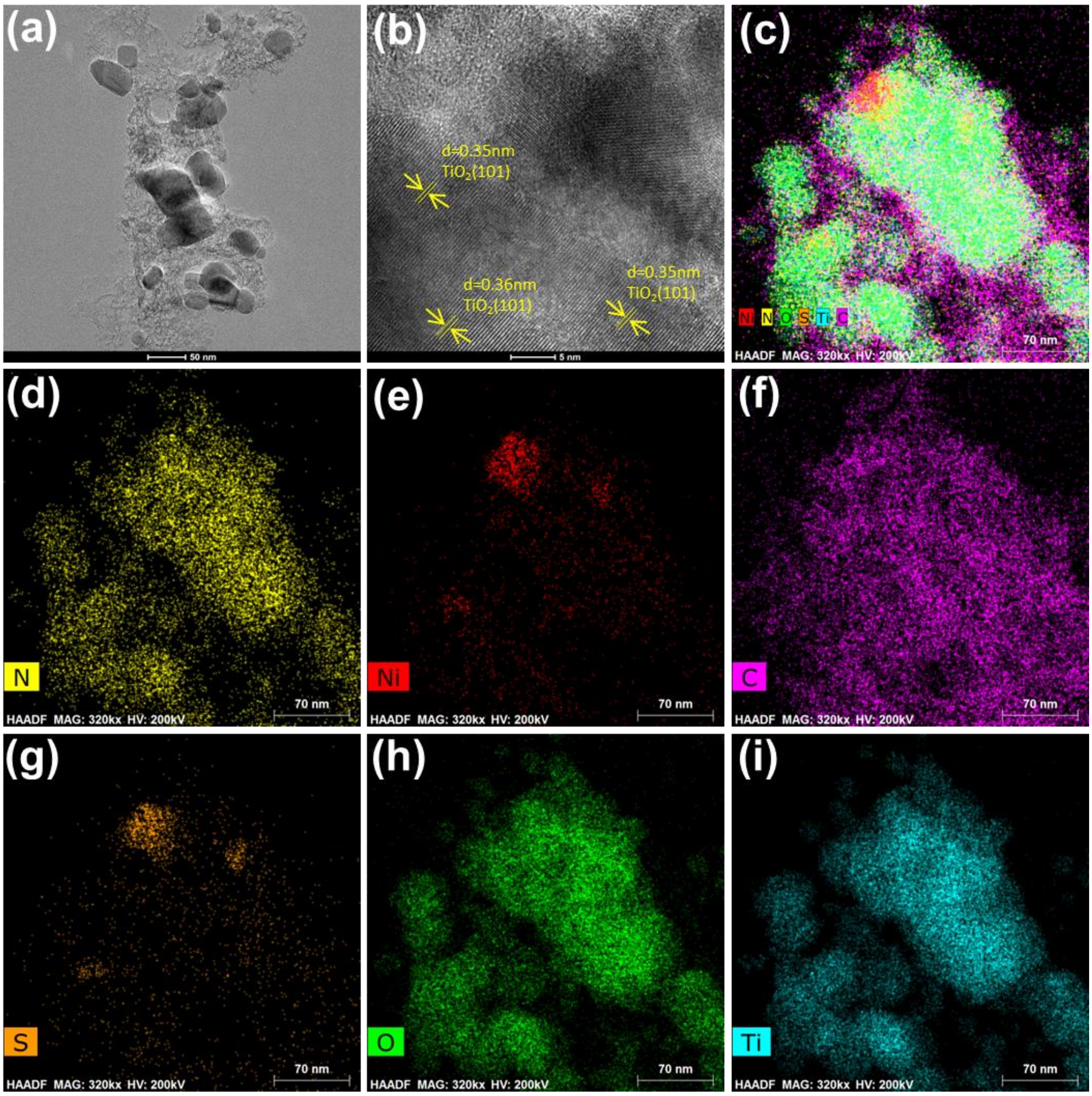

Supplementary Figure 6. TEM images of Ni-L3@TiO$_2$-800 (a). TiO$_2$ (101) facet with lattice spacings of 0.35 nm (b) were observed. The EDS-mapping of the Ni-L3@TiO$_2$-800 catalyst (c) showed homogeneous dispersion of C (f), N (d), O (h), and Ti (i) species. The distribution of Ni (e) is consistent with that of S (g), indicating the existing of Ni$_3$S$_2$.

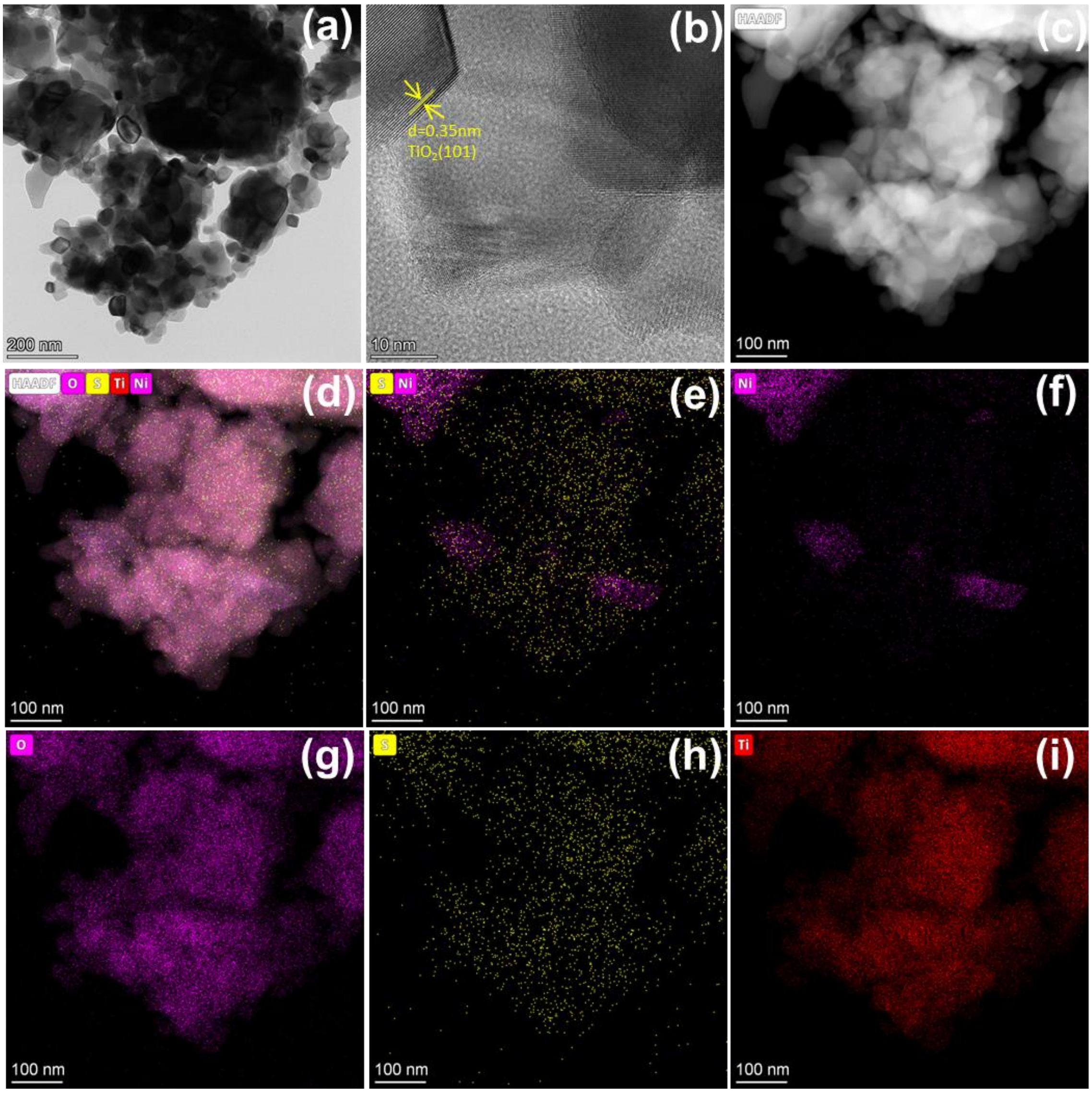

Supplementary Figure 7. TEM images of Ni@TiO_2-800 (material prepared without any ligand) (a, c). TiO_2 (101) facet with lattice spacings of 0.35 nm (b) were observed. The EDS-mapping of the Ni@TiO_2-800 (d) showed dispersion of O (g), S (e, h), Ni (e, f) and Ti (i) species.

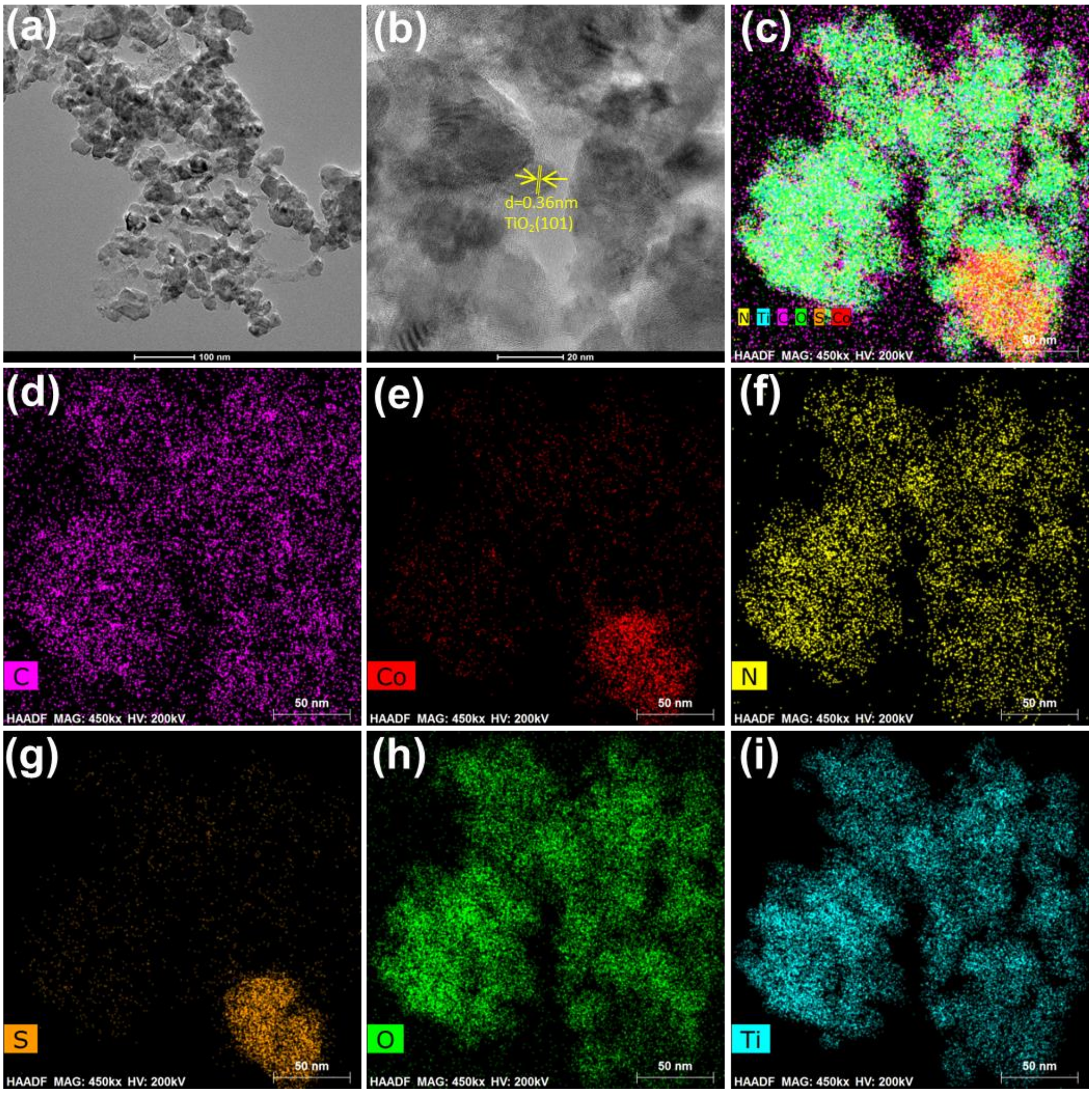

Supplementary Figure 8. TEM images of Co-L1@TiO$_2$-800 (a). TiO$_2$ (101) facet with lattice spacings of 0.35 nm (b) were observed. The EDS-mapping of the Co-L1@TiO$_2$-800 catalyst (c) showed homogeneous dispersion of C (d), N (f), O (h), and Ti (i) species. The distribution of Co (e) is consistent with that of S (g).

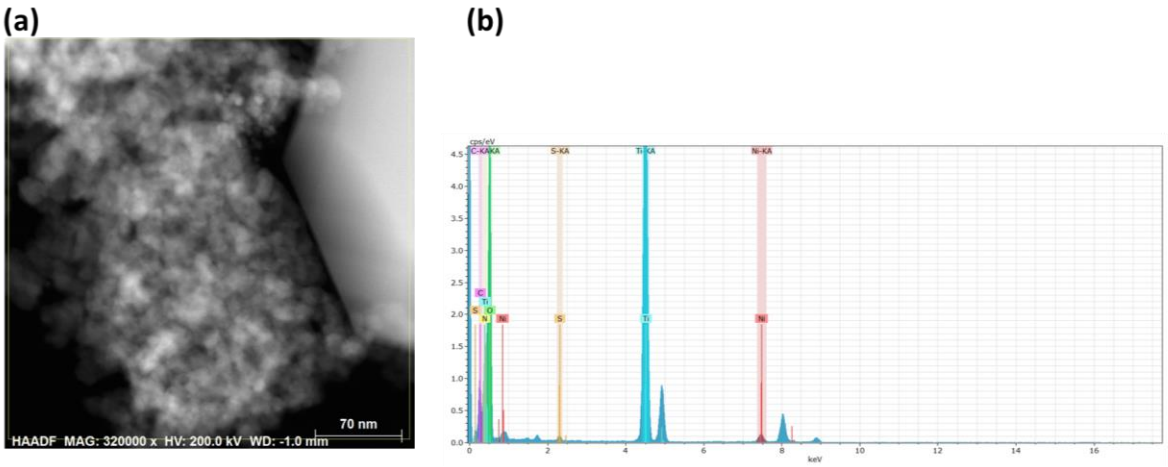

Supplementary Figure 9. (a) TEM image and (b) the EDX data of image (a), obtained by Ni-L1@TiO$_2$-800 material. Elements of Sulfur (S), Nickel (Ni), Carbon (C), Nitrogen (N), Oxygen (O) and Titanium (Ti) are clearly detected.

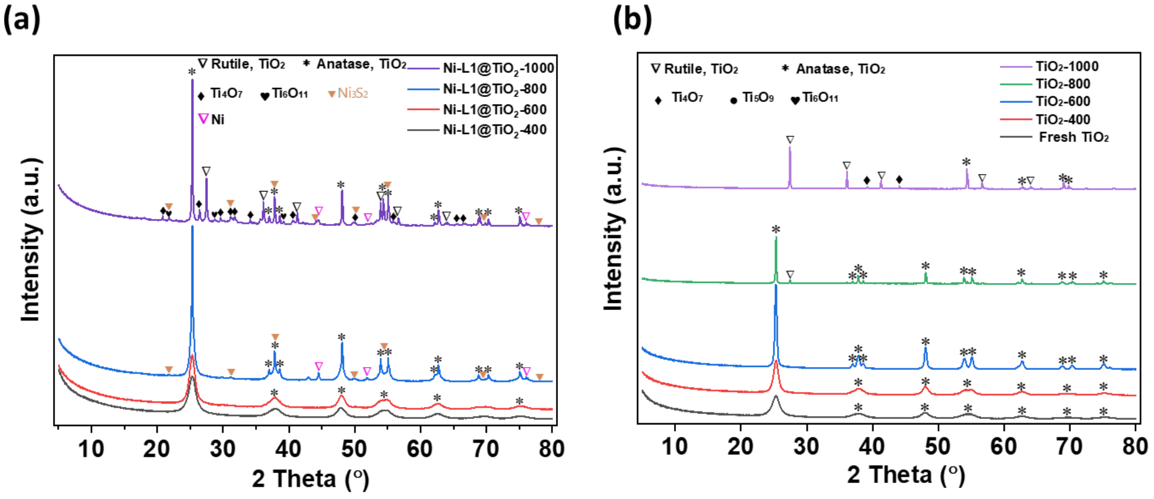

Supplementary Figure 10. XRD patterns. (a) Ni-L1@TiO_2-400, Ni-L1@TiO_2-600, Ni-L1@TiO_2-800, Ni-L1@TiO_2-1000, and (b) Fresh TiO_2, TiO_2-400, TiO_2-600, TiO_2-800, TiO_2-1000.

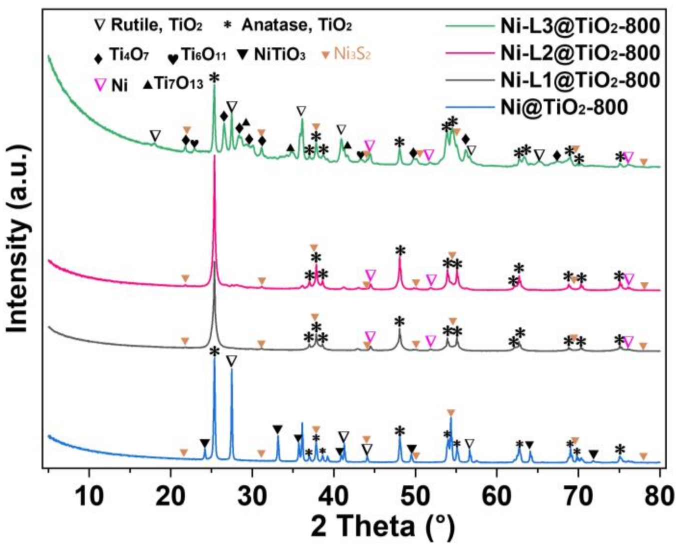

Supplementary Figure 11. XRD patterns. Ni@TiO_2-800, Ni-L1@TiO_2-800, Ni-L2@TiO_2-800, Ni-L3@TiO_2-800.

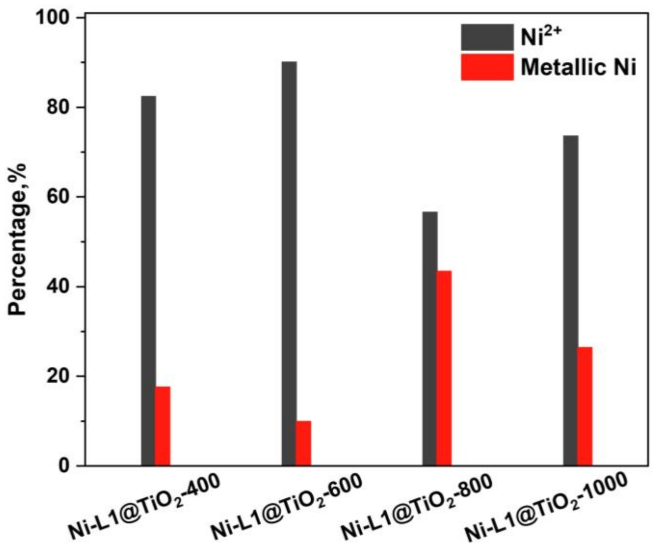

Supplementary Figure 12. The percentage of Ni^{2+} and metallic Ni based on XPS measurements. Ni-L1@TiO_2-400, Ni-L1@TiO_2-600, Ni-L1@TiO_2-800 and Ni-L1@TiO_2-1000.

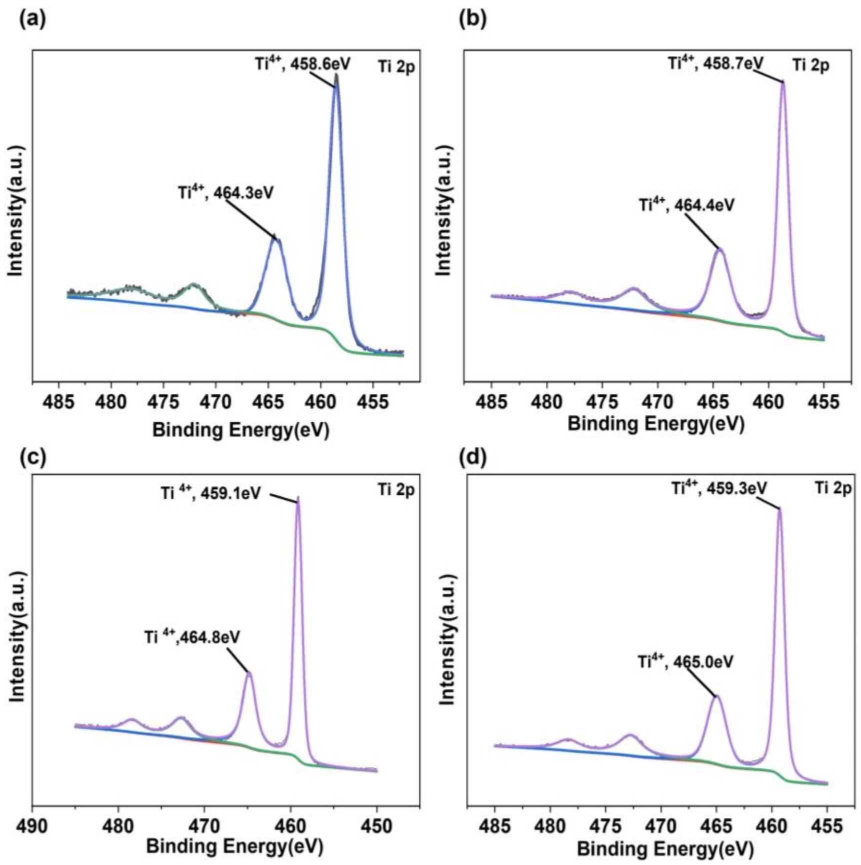

Supplementary Figure 13. XPS spectra of Ti 2p. (a) Ni-L1@TiO_2-400, (b) Ni-L1@TiO_2-600, (c) Ni-L1@TiO_2-800 and (d) Ni-L1@TiO_2-1000.

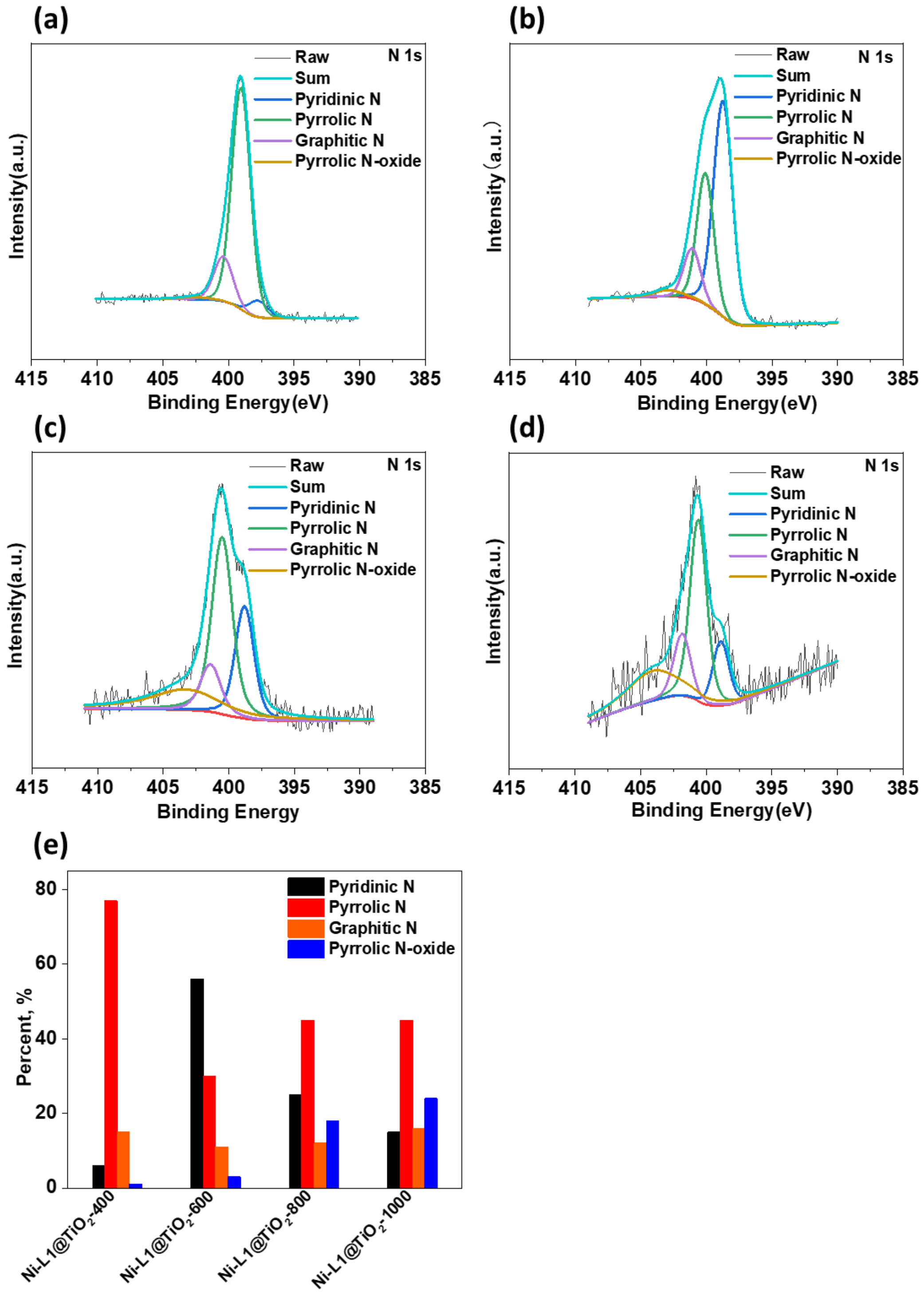

Supplementary Figure 14. XPS spectra of N 1s. (a) Ni-L1@TiO_2-400, (b) Ni-L1@TiO_2-600, (c) Ni-L1@TiO_2-800, (d) Ni-L1@TiO_2-1000 and (e) the percentage of N based on the XPS result.

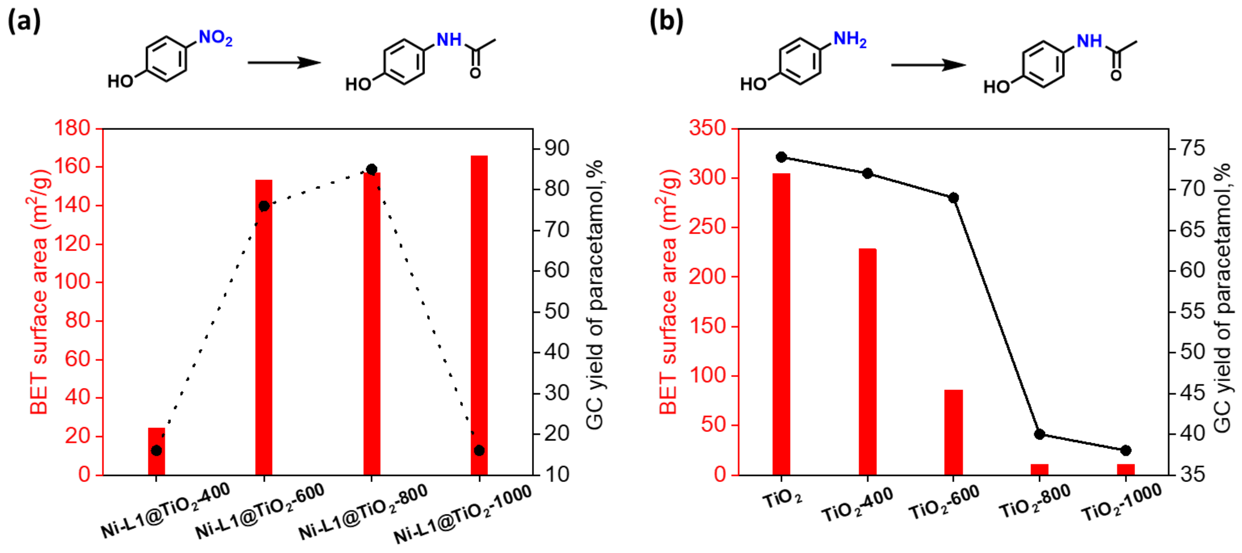

Supplementary Figure 15. (a) BET surface area (read column) of different Ni-catalysts and yield of paracetamol in nickel catalyzed amidation of 4-nitrophenol and ethyl acetate (black dots). Reaction conditions: 0.5 mmol 4-nitrophenol, 1 mL ethyl acetate, 60 mg Ni-L1@TiO_2-T, 20 bar H_2, 130 °C, 24 h. (b) BET surface area of TiO_2 pyrolyzed at different temperatures (read column) and yield of paracetamol by the TiO_2-cataylzed amidation of 4-aminophenol and ethyl acetate (black dots). Reaction conditions: 0.5 mmol 4-aminophenol, 1 mL ethyl acetate, 20 mg TiO_2-T, 20 bar H_2, 130 °C, 24 h.

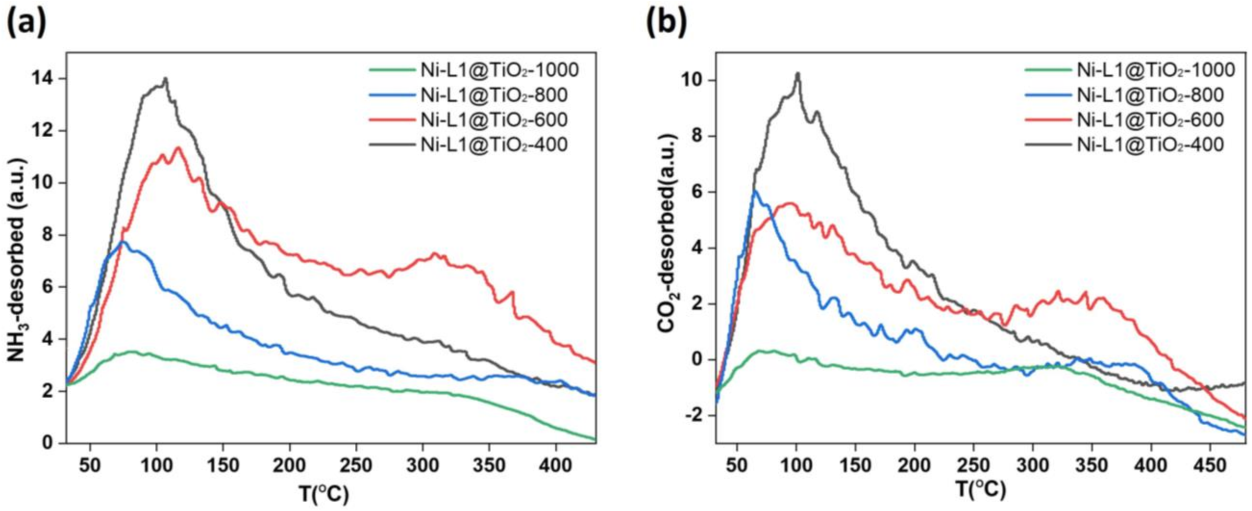

Supplementary Figure 16. **TPD measurements.** (a) NH_3-TPD and (b) CO_2-TPD of Ni-L1@TiO_2-400, Ni-L1@TiO_2-600, Ni-L1@TiO_2-800 and Ni-L1@TiO_2-1000.

S2.4. Kinetic and control experiments.

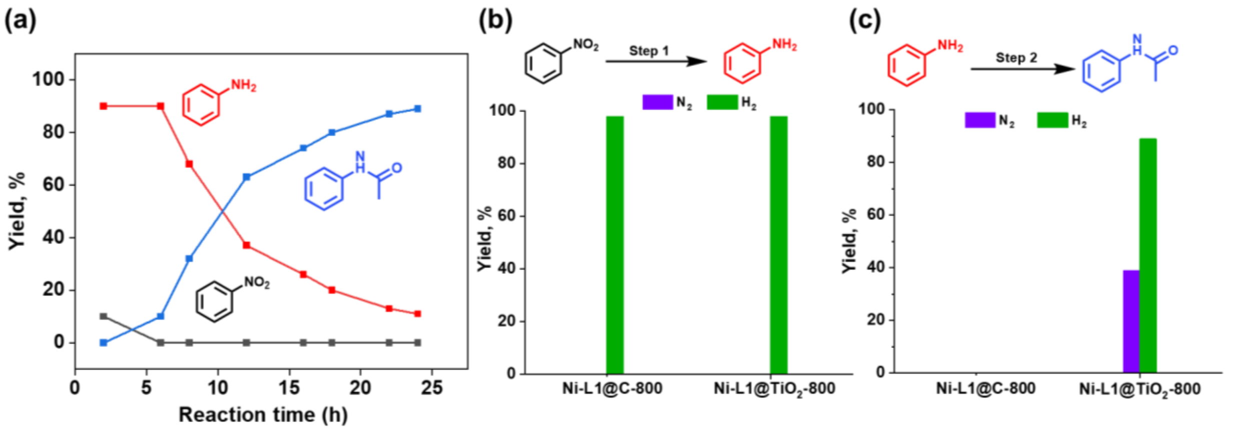

Supplementary Figure 17. Kinetic and control experiments. (a) Kinetic studies. Reaction conditions: 0.5 mmol nitrobenzene, 1 mL ethyl acetate, 60 mg Ni-L1@TiO_2-800, 20 bar H_2, 130 °C, 24 h. (b-c) Control experiments. Reaction conditions: 60 mg Ni-L1@TiO_2-800, 20 bar H_2 or N_2, 130 °C, 24 h. (b) 0.5 mmol nitrobenzene, 1 mL ethanol. (c) 0.5 mmol aniline, 1 mL ethyl acetate.

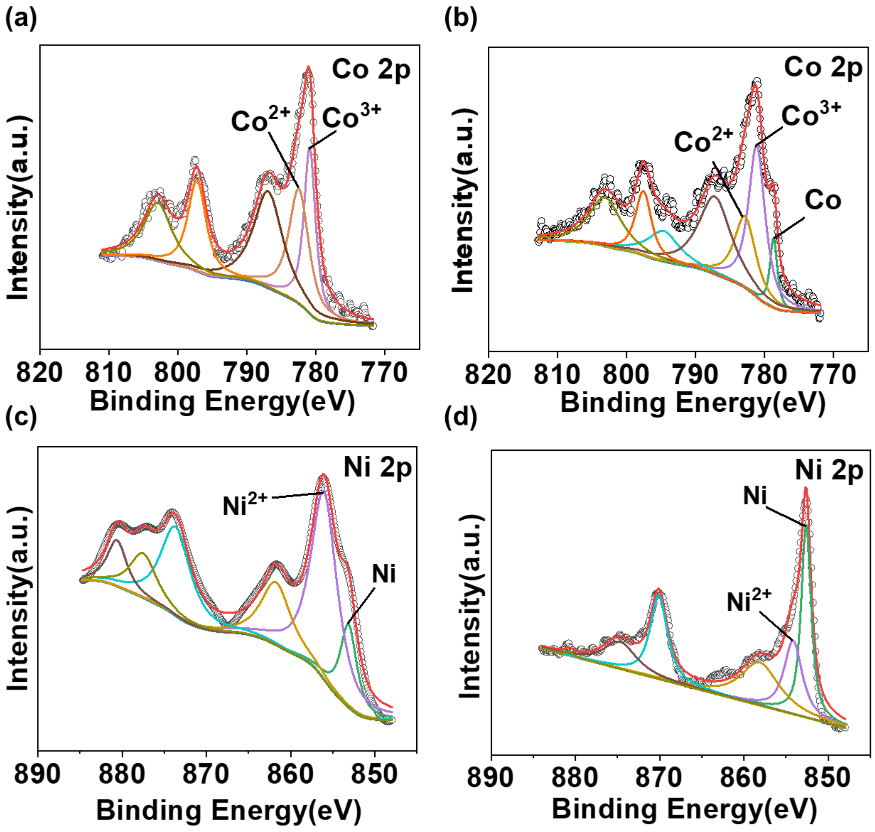

Supplementary Figure 18. In-situ XPS spectra. (a, c) 130 °C, 1 bar N_2, (b, d) 130 °C, 1 bar H_2, (a, b) Co 2p of Co-L1@TiO_2-800. (c, d) Ni 2p of Ni-L1@TiO_2-800. In presence of H_2, metallic cobalt is found, and Ni^{2+} is transferred into metallic nickel.

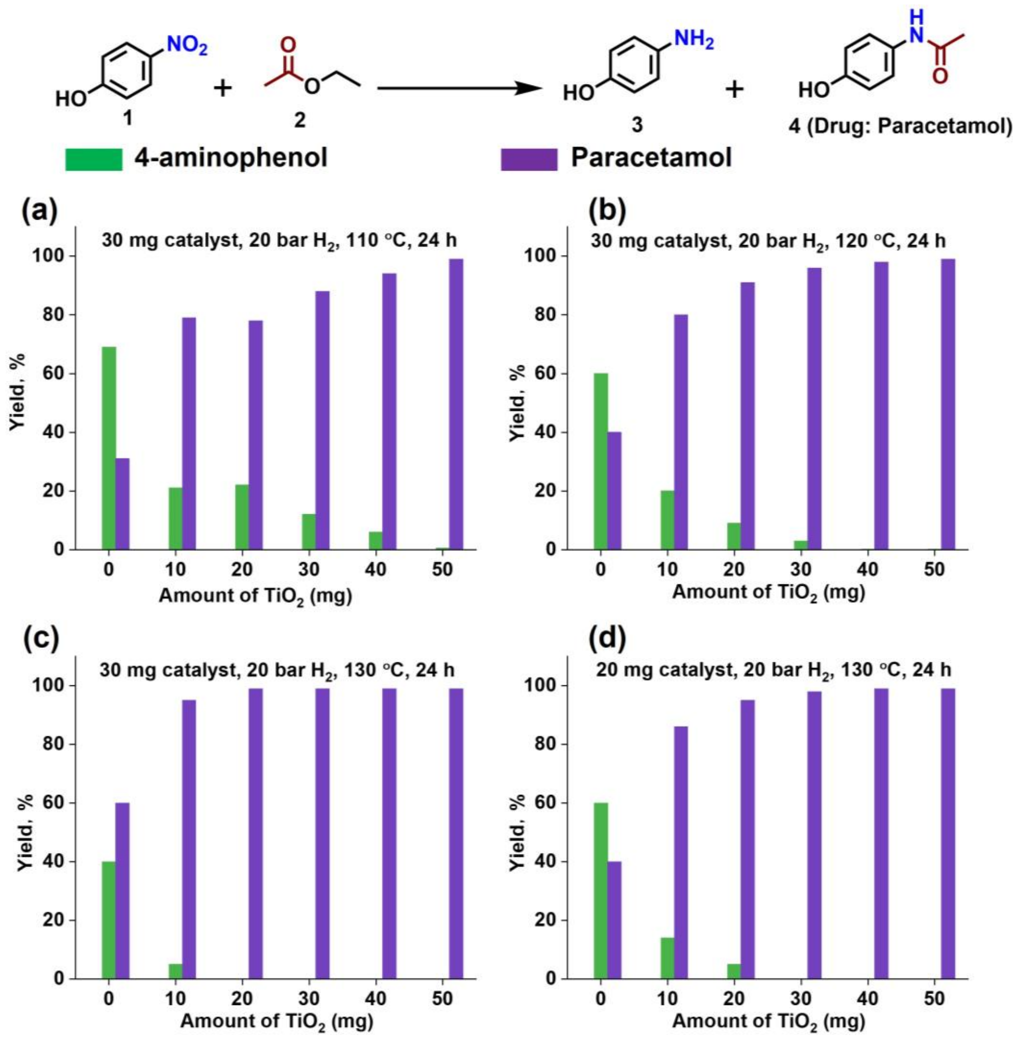

Supplementary Figure 19. Ni-catalysed amidation of 4-nitrophenol and ethyl acetate in presence of additional TiO_2 support. Reaction conditions: 0.5 mmol 4-nitrophenol, 1 mL ethyl acetate. (a) 30 mg Ni-L1@TiO_2-800, 20 bar H_2, 110 ℃, 24 h. (b) 30 mg Ni-L1@TiO_2-800, 20 bar H_2, 120 ℃, 24 h. (c) 30 mg Ni-L1@TiO_2-800, 20 bar H_2, 130 ℃, 24 h. (d) 20 mg Ni-L1@TiO_2-800, 20 bar H_2, 130 ℃, 24 h.

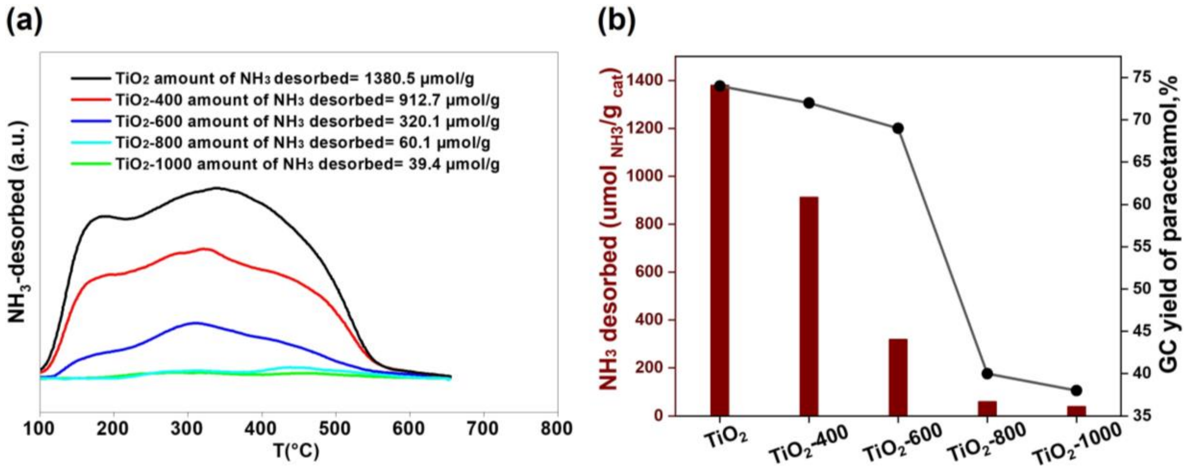

Supplementary Figure 20. NH_3-TPD, and the correlation of the amount of desorbed NH_3 and Paracetamol yield (a) NH_3-TPD of TiO_2 series materials (TiO_2, TiO_2-400, TiO_2-600, TiO_2-800, TiO_2-1000). (b) The correlation of the amount of desorbed NH_3 and Paracetamol yield (TiO_2, TiO_2-400, TiO_2-600, TiO_2-800, TiO_2-1000).

S2.5. Physical states of esters used in this work.

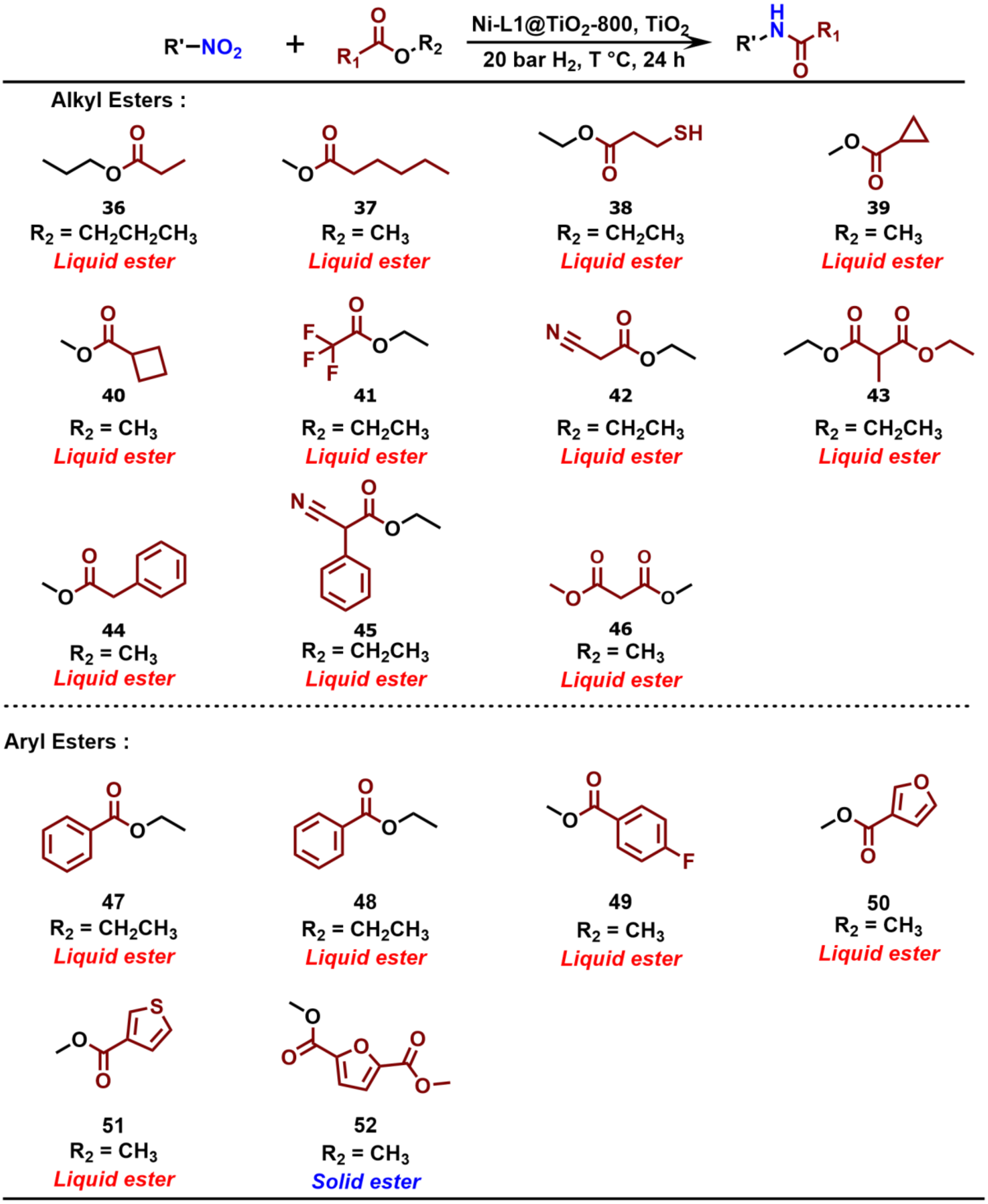

Supplementary Figure 21. Information of esters. The structure and physical state of the esters at room temperature used in Figure 8.

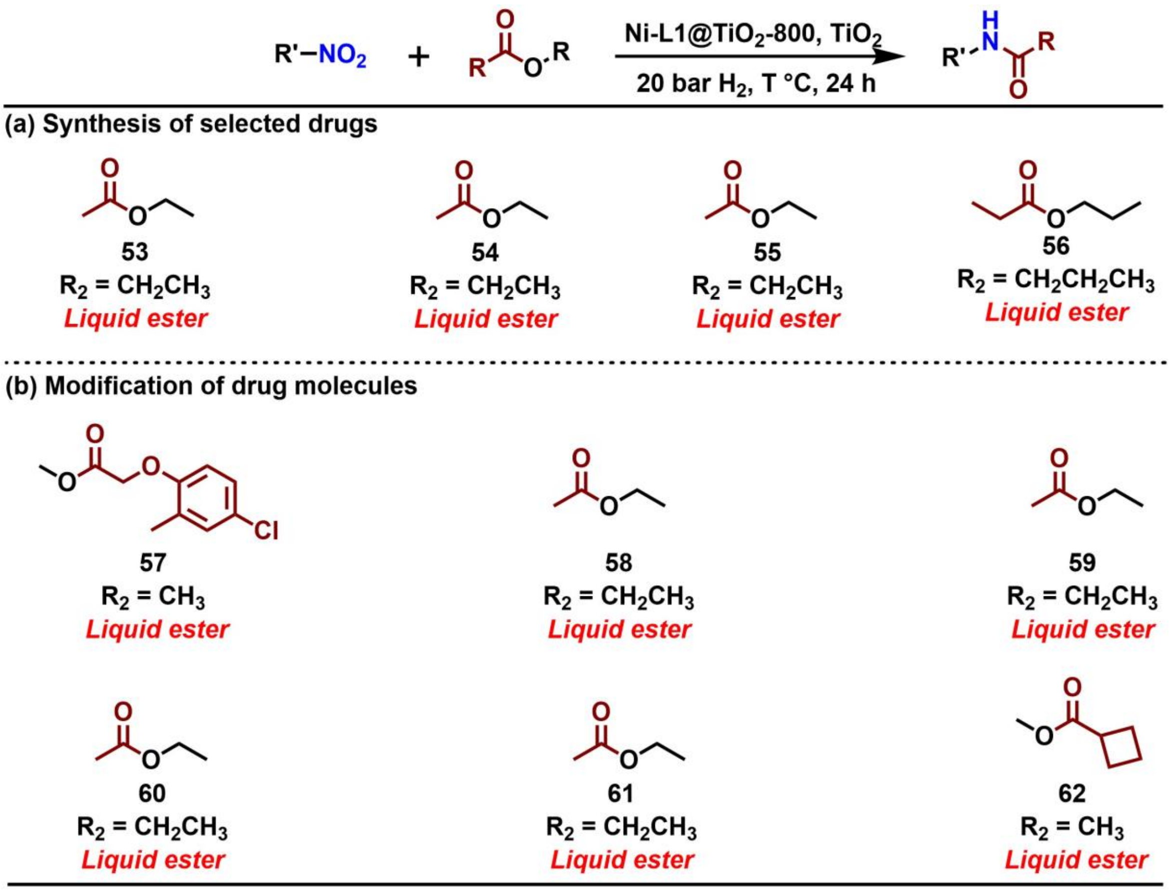

Supplementary Figure 22. Information of esters:(a) esters used for the synthesis of drugs; (b) esters used for the modification of drug molecules. The structure and physical state of the esters at room temperature used in Figure 9.

S2.6. DFT Computational details.

The density functional theory (DFT) calculations were performed using the Cambridge Serial Total Energy Package (CASTEP) codes.[1] The generalized gradient approximation (GGA) of Perdew−Burke−Ernzerhof (PBE) was used.[2] Electron-ion interactions were described by the Ultrasoft pseudopotentials.[3.] The valence electron configurations were $3S^23P^64s^2$ for Ti atom, $2S^22P^4$ for O and, $2S^22p^2$ for C, $2S^22P^3$ for N and $1S^1$ for H, respectively. The minimization algorithm was the Broyden-Fletcher-Goldfarb-Shanno (BFGS) scheme. The convergence tolerances for the geometry optimization were set to 10^{-5} eV/atom for the energy, 0.03 eV/Å for the force, and 0.001 Å for the displacement. The energy cutoff of plane-wave basis set was 500 eV. and the self-consistent field (SCF) convergence tolerance was set to 1.0×10^{-6} eV/atom. The long-range van der Vaals interactions were corrected with Grimme's method.[4]

As shown in the HRTEM images (Figure 3b), the anatase-TiO_2 (101) surface is the main crystal surface, thus, the (101) anatase was simulated by periodic (3 × 3) slab models with three O–Ti–O layers. A 15 Å thick area of vacuum in the z-direction are employed to avoid non-physical

interaction between neighboring slabs. For the optimization of the unit cell geometry, the bottom O–Ti–O atomic layer was considered as constrained and the upper two atom layers were relaxed.

It is reported that the hydrogen treated TiO_2 generates oxygen vacancies (O_v).[5] This O_v leads to the creation of unpaired electrons, or Ti^{3+} centers, ($O^{2-} + 2Ti^{4+} \rightarrow O_v + 1/2O_2 + 2Ti^{3+}$). Thus, the reduced TiO_2 was molded by creating oxygen vacancies on the TiO_2(101) surface. To localized Ti^{3+} centers, we firstly removed a bridging oxygen atom from anatase TiO_2 (101) surface. Then, a polaronic distortion around the two Ti atoms with localize unpaired electrons was created by stretching Ti-O bond by about 0.05 Å. The excess of electrons associated with the Ti 3d orbitals were treated using DFT+U method. In order to delocalized excess charge, different U value from 4 to 8 eV was tested, and U= 8 eV could effectively locate the unpaired electrons on Ti^{3+} atom (the spin density in Figure 23 indicates the positions of the electrons).

The adsorption energies (Eads) of aniline on TiO_2 surfaces were calculated as:

$$E_{ads} = E_{aniline/Surface} - E_{aniline} - E_{Surface}$$

where $E_{aniline/Surface}$, $E_{aniline}$ and $E_{Surface}$ is the energy of TiO_2 slab with adsorbed aniline,

$E_{aniline}$ is the energy of the aniline calculated in a 10 × 10 cubic cell, $E_{Surface}$ is the energy of the TiO_2(010) slab. The Hirshfeld analysis was employed to determine the atomic charges and charge-transfer values.[6]

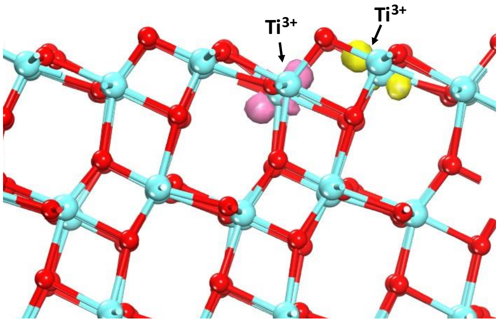

Supplementary Figure 23. The spin density of anatase-TiO_2 (101) surfaces. The blue contour indicates spin up, whereas the purple contour indicates spin down.

Supplementary Figure 24 shows the most stable configuration of aniline adsorbed on Ti_{5c}^{4+} and Ti_{5c}^{3+} and Ti_{4c}^{3+}atoms of TiO_2(010) surface. In all these configurations, the aniline is tilted to bond with a Ti atom. The molecular plane was tilted relative to the TiO_2(010) surface and the N atom downward and H atoms upward. These variations mainly result from the interaction between N_{2p} electrons and 3d electrons of Ti_{5c} atom, forming the new N–Ti bond, which leads to the weakening of the repulsion interaction between the bonding electrons of the N–H bonds in adsorbed aniline. The Ti–N bond

length is measured to be 2.380 Å when aniline adsorbed on $TiO_2(010)$ surface with Ti^{4+} atom. These results are consistent with the experimental observations of the interactions of aniline with the rutile TiO_2 (110) surface by scanned-energy electron diffraction (PhD) and by near-edge X-ray absorption fine structure (NEXAFS) (2.27±0.04 Å).[7]

The E_{ads} of aniline molecule on the atop a 5-fold coordinated Ti^{4+} atom (denoted as Ti_{5c}^{4+} (Fig.1a) was -0.959 eV. The H–N–H bond angles vary from 118.4° in the gas-phase aniline to 109.98° and the bond length of two N–H bonds were elongated from 1.018 Å in an original aniline molecule to 1.027 Å in the chemisorbed state.

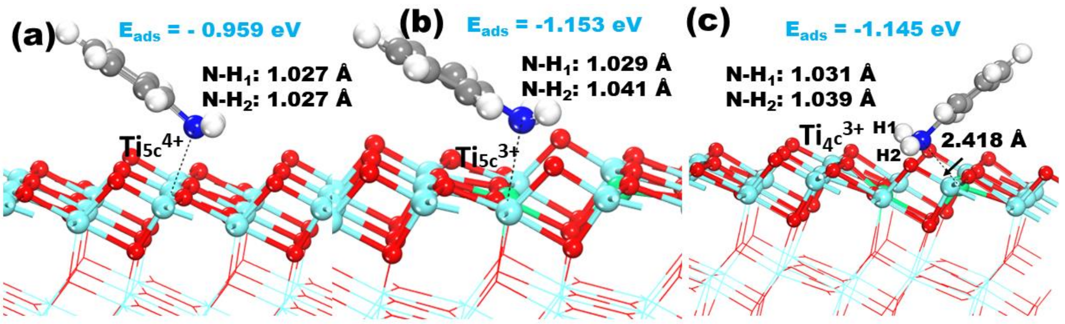

Supplementary Figure 24. Configuration. The most stable configuration of aniline adsorbed on a 5-fold coordinated Ti^{4+} atom (Ti_{5c}^{4+}, a), 5-fold coordinated Ti^{3+} atom (Ti_{5c}^{3+}, b) and a 4-fold coordinated Ti^{3+} atom (Ti_{4c}^{3+}, c).

The E_{ads} is -1.153 eV and -1.034 eV when aniline molecule interacts with a 5-fold coordinated Ti^{3+} atom (Ti_{5c}^{3+}) and a 4-fold coordinated Ti^{3+} atom (Ti_{4c}^{3+}), respectively. It suggests that aniline is thermodynamically favorable to interact with Ti^{3+} ion rather than Ti^{4+} ion. The distance of Ti_{5c}^{3+}–N and Ti_{4c}^{3+}–N is 2.618 Å and 2.418 Å, respectively. In both of the configurations, the NH_2 group in the aniline molecule are slightly leaned to an adjacent bridge oxygen (O_b) on the TiO_2 surface. The hydrogen-bond interactions between O_b and H in NH_2 group leading to one of the N–H bonds elongation. The N–H bond length is found to be 1.041 and 1.029 Å as well as 1.039 Å and 1.031 Å in aniline when it interacts with Ti_{5c}^{3+} and Ti_{4c}^{3+} respectively. The closest distance between H in aniline and O_b is 2.156 and 2.322 Å in aniline/Ti_{5c}^{3+} and aniline/Ti_{4c}^{3+} systems. Thus, stronger interactions in the aniline/Ti_{5c}^{3+} system are associated with an increase in hydrogen bond strength. Apparently, when aniline interacts with Ti^{3+}, the N–H bonds are longer than that the interaction of aniline-Ti^{4+}. It suggests that the interaction of aniline with Ti^{3+} promotes the activation and dissociation of N–H bonds more effectively than the interaction with Ti^{4+}.

S2.7. Elemental analysis (EA) report.

service – elemental analytics

Quantitative determination of various elements:

C, H, N, S (combustion analysis) ☒

Device: C/H/N/S – microanalyzer -TruSpec CHNS Micro- company Leco

Specifications:

Range: @ 2 mg:	Precision @ 2 mg:
Carbon: 0,002- 100 %	<1 % RSD bzw. ± 0,001 % (whichever is greater)
Hydrogen: 0,020- 50 %	<1 % RSD bzw. ± 0,010 % (whichever is greater)
Nitrogen: 0,020- 50 %	<1 % RSD bzw. ± 0,010 % (whichever is greater)
Sulfur: 0,040- 65 %	<1 % RSD bzw. ± 0,020 % (whichever is greater)

**Measuring method**	Sample Size	Temperature	Detectors
CHNS	up to 1 mg	1100°C	IR- C, H, S und WL- N

The sample is catalytically combusted with pure oxygen in a helium flow (combustion method). The evaluation is carried out with IR detectors respectively for C, H, S and for N a thermal conductivity detector is used.

Cl, Br, I (potentiometric titration) ☐ ***F (ion selective electrode)*** ☐

general titration ☐ ***Karl Fischer titration*** ☐

Digestion type for halogens: Combustion with oxygen in the Schöniger flask

Device: Titrator Excellence T7 - company Mettler Toledo

**Measuring methods:**	Method type	Measuring range	Error limit	Electrode
pH measurement	Titration general, measurement normal	pH 0-12		DGi 113-SC
OHZ:	Equivalent point titration	-20000-+2000 mV	0,2 mV	DGi 113-SC
halogens:	Equivalent point titration	-20000-+2000 mV	0,2 mV	DMi 145-SC
fluoride:	measurement normal	1*10-6 mol/L–saturated	(0,02 mg/L–saturated)	perfectION comb F
Water content:	KF volumetric	0-+2000 mV /0-200µA	2,0 mV / 0,2 µA	DM143-SC

service – elemental analytics

Quantitative determination of various elements:

Digestion types

☐ acidic H2SO4 - KHSO4 metal digestion in quartz crucible

☐ aqua regia digestion in quartz crucible or microwave for silicon with hydrofluoric acid

☐ Soda - Potash - Digestion for Silicium

☐ acidic HNO3 - $Mg(NO_3)_2$ digestion for mainly phosphorus in the quartz crucible

Elemental analyses (photometric) ☐ ***Overview spectrum*** ☐

Device: UV/ VIS spectrometer - Lambda 2 - company Perkin Elmer

Operating principle:

Dual-beam spectrometer for measuring absorbance and concentration units in the visible range VIS from 1100-322.4 nm and ultraviolet range UV from 322.4- 190 nm. The element P is quantitatively determined photometrically.

Measuring methods:	Method typ	Wavelengths	Radiation sources
overview spectrum	Scan Mode	190- 1100 nm	deuterium and tungsten halogen lamps
phosphorus	Conc	Calibration curve	deuterium and tungsten halogen lamps

Metal analyses in the range of 190 - 900 nm (atomic absorption spectroscopy) ☒

Device: AAS – contrAA800D - company Analytik Jena

Operating principle:

AAS- instrument is a computer controlled routine atomic absorption, emission, flame graphite furnace spectrometer for flame and graphite furnace technique, Echelle- double monochromator, line evaluation of metals in the range of 190-900 nm with one lamp (Xenon short arc lamp), Burner system for air/ acetylene (temperature 2150-2400 °C) and nitrous oxide/ acetylene (temperature 2650-2800 °C), CCD array detector, sample sampler, mixing chamber atomizer unit with injection switch or graphite tube furnace and extensive safety system.

Measuring methods:

The characteristic concentration, the sensitivity test, the linear range, the burner system and other indications for the metal to be determined can be found in the cookbook for the instrument in the measuring program.

Supplementary Figure 25. EA. The Elemental analysis procedure.

3. NMR and HRMS Data.

In this work, 61 products were synthesized, and all the products were isolated using column chromatography. NMR data and spectra of all these products are given bellow. Among the 61 synthesized products, 46 products are already reported, and the corresponding references are given to complement the NMR characterizations. For the 15 new products, NMR data and spectra as well as HRMS data and spectra are shown (see section 5, HRMS Spectra).

Paracetamol (4)[8]

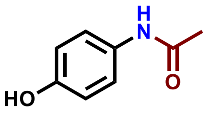

^{1}H NMR (300 MHz, DMSO) δ 9.63 (s, 1H), 9.10 (s, 1H), 7.51 – 7.23 (m, 2H), 6.80 – 6.51 (m, 2H), 1.98 (s, 3H).

^{13}C NMR (75 MHz, DMSO) δ 167.96, 153.59, 131.47, 121.29, 115.42, 24.17.

N-phenylacetamide (5)[9]

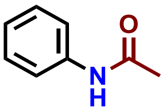

^{1}H NMR (300 MHz, $CDCl_3$) δ 8.31 (s, 1H), 7.58 – 7.50 (m, 2H), 7.36 – 7.25 (m, 2H), 7.16 – 7.07 (m, 1H), 2.15 (s, 3H).

^{13}C NMR (75 MHz, $CDCl_3$) δ 169.19, 138.11, 128.91, 124.31, 120.25, 24.39.

N-(4-isopropylphenyl)acetamide (6)[10]

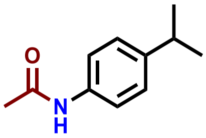

^{1}H NMR (300 MHz, DMSO) δ 9.85 (s, 1H), 7.59 – 7.38 (m, 2H), 7.24 – 7.05 (m, 2H), 2.81 (h, J = 6.9 Hz, 1H), 2.03 (s, 3H), 1.17 (d, J = 6.9 Hz, 6H).

^{13}C NMR (75 MHz, DMSO) δ 168.47, 143.44, 137.57, 126.77, 119.57, 33.31, 24.41, 24.36.

N-(naphthalen-1-yl)acetamide (7)[11]

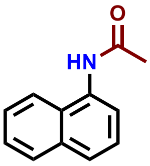

¹H NMR (300 MHz, DMSO) δ 9.94 (s, 1H), 8.14 – 8.07 (m, 1H), 7.97 – 7.90 (m, 1H), 7.74 (dd, *J* = 12.9, 7.8 Hz, 2H), 7.59 – 7.46 (m, 3H), 2.21 (s, 3H).

¹³C NMR (75 MHz, DMSO) δ 169.43, 134.18, 128.58, 128.15, 126.44, 126.21, 126.03, 125.52, 123.21, 122.01, 23.96.

N-([1,1'-biphenyl]-2-yl)acetamide (8)[12]

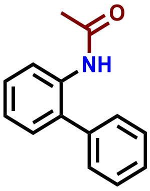

¹H NMR (300 MHz, DMSO) δ 9.25 (s, 1H), 7.54 – 7.19 (m, 9H), 1.89 (s, 3H).

¹³C NMR (75 MHz, DMSO) δ 169.18, 139.50, 137.19, 135.34, 130.69, 129.17, 128.83, 128.12, 127.88, 127.65, 126.46, 23.44.

N-(9H-fluoren-2-yl)acetamide (9)[13]

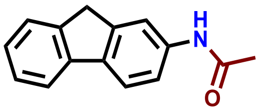

¹H NMR (300 MHz, DMSO) δ 10.03 (s, 1H), 7.94 (q, *J* = 0.9 Hz, 1H), 7.82 – 7.76 (m, 2H), 7.57 – 7.51 (m, 2H), 7.35 (td, *J* = 7.5, 1.2 Hz, 1H), 7.25 (td, *J* = 7.4, 1.2 Hz, 1H), 3.89 (s, 2H), 2.09 (s, 3H).

¹³C NMR (75 MHz, DMSO) δ 168.70, 144.17, 143.22, 141.52, 138.91, 136.59, 127.17, 126.48, 125.45, 120.52, 119.84, 118.25, 116.27, 36.97, 24.55.

N-(4-(tert-butyl)phenyl)acetamide (10)[14]

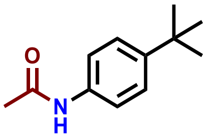

¹H NMR (300 MHz, DMSO) δ 9.93 (s, 1H), 7.56 – 7.43 (m, 2H), 7.38 – 7.22 (m, 2H), 2.03 (s, 3H), 1.25 (s, 9H).

¹³C NMR (75 MHz, DMSO) δ 168.52, 145.66, 137.26, 125.65, 119.28, 34.40, 31.66, 24.36:

N-mesitylacetamide (11)[15]

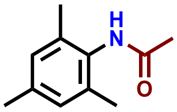

^{1}H NMR (300 MHz, DMSO) δ 9.16 (s, 1H), 6.87 – 6.82 (m, 2H), 2.22 (s, 3H), 2.08 (s, 6H), 2.02 (s, 3H).

^{13}C NMR (75 MHz, DMSO) δ 168.35, 135.62, 135.28, 133.28, 128.63, 23.00, 20.94, 18.49.

N-(4-fluorophenyl)acetamide (12)[16]

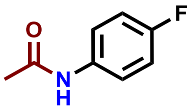

^{1}H NMR (300 MHz, DMSO) δ 9.98 (s, 1H), 7.59 (dd, J = 9.2, 5.1 Hz, 2H), 7.12 (dd, J = 9.2, 8.7 Hz, 2H), 2.03 (s, 3H).

^{13}C NMR (75 MHz, DMSO) δ 168.19, 157.84 (d, J = 239.3 Hz), 135.75 (d, J = 2.7 Hz), 120.70 (d, J = 7.8 Hz), 115.20 (d, J = 22.2 Hz), 23.86.

N-(2-fluorophenyl)acetamide (13)[17]

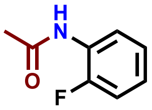

^{1}H NMR (300 MHz, $CDCl_3$) δ 8.28 (td, *J* = 8.1, 1.8 Hz, 1H), 7.56 (s, 1H), 7.22 – 6.93 (m, 3H), 2.23 (s, 3H).

^{13}C NMR (75 MHz, CDCl3) δ 168.59, 152.51 (d, J = 242.8 Hz), 126.41 (d, J = 10.3 Hz), 124.62 (d, J = 3.7 Hz), 124.45 (d, J = 7.7 Hz), 122.06, 114.87 (d, J = 19.2 Hz), 24.67.

N-(3-hydroxy-5-(trifluoromethyl)phenyl)acetamide (14)[18]

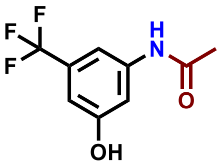

^{1}H NMR (300 MHz, DMSO) δ 10.13 (s, 1H), 7.51 – 7.30 (m, 2H), 6.71 (ddt, *J* = 2.3, 1.6, 0.7 Hz, 1H), 2.05 (s, 3H).

^{13}C NMR (75 MHz, DMSO) δ 168.85, 158.30, 141.25, 130.28 (q, J = 31.4 Hz), 124.08 (q, J = 272.2 Hz), 109.15, 106.37 (q, J = 4.0 Hz), 106.00 (q, J = 4.0 Hz), 24.12.

N-(3-chloro-4-fluorophenyl)acetamide (15)[19]

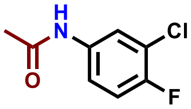

1H NMR (300 MHz, DMSO) δ 10.12 (s, 1H), 7.90 (dd, J = 6.9, 2.6 Hz, 1H), 7.42 (ddd, J = 9.1, 4.4, 2.6 Hz, 1H), 7.31 (t, J = 9.1 Hz, 1H), 2.04 (s, 3H).

13C NMR (75 MHz, DMSO) δ 168.55, 152.92 (d, J = 242.3 Hz), 136.56 (d, J = 3.0 Hz), 120.30, 119.14 (d, J = 6.7 Hz), 118.94, 116.82 (d, J = 21.7 Hz), 23.90.

N-(4-chlorophenyl)acetamide (16)[20]

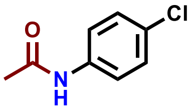

^{1}H NMR (300 MHz, DMSO) δ 10.12 (s, 1H), 7.72 – 7.58 (m, 2H), 7.46 – 7.13 (m, 2H), 2.05 (s, 3H).

^{13}C NMR (75 MHz, DMSO) δ 168.93, 138.75, 129.01, 126.94, 120.94, 24.44.

N-(2,4-dichlorophenyl)acetamide (17)[21]

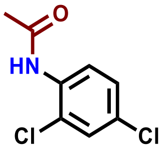

^{1}H NMR (300 MHz, $CDCl_3$) δ 8.35 (d, *J* = 8.9 Hz, 1H), 7.66 – 7.52 (m, 1H), 7.39 (d, *J* = 2.4 Hz, 1H), 7.26 (ddd, *J* = 8.9, 2.4, 0.5 Hz, 1H), 2.26 (s, 3H).

^{13}C NMR (75 MHz, $CDCl_3$) δ 168.25, 133.37, 129.05, 128.68, 127.91, 123.02, 122.32, 24.85.

N-(2,5-dimethoxyphenyl)acetamide (18)[22]

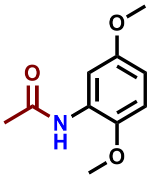

^{1}H NMR (300 MHz, DMSO) δ 9.10 (s, 1H), 7.69 (d, *J* = 3.1 Hz, 1H), 6.93 (d, *J* = 8.9 Hz, 1H), 6.61 (dd, *J* = 8.9, 3.1 Hz, 1H), 3.78 (s, 3H), 3.68 (s, 3H), 2.09 (s, 3H).

^{13}C NMR (75 MHz, DMSO) δ 169.07, 153.32, 143.88, 128.75, 112.14, 108.76, 108.17, 56.58, 55.75, 24.45.

N-propylacetamide (19)[17]

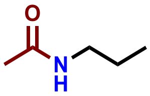

^{1}H NMR (400 MHz, DMSO) δ 7.83 (s, 1H), 2.97 (td, *J* = 7.1, 5.7 Hz, 2H), 1.79 (s, 3H), 1.39 (td, *J* = 7.4, 6.6 Hz, 2H), 0.83 (t, *J* = 7.4 Hz, 3H).

^{13}C NMR (101 MHz, DMSO) δ 169.43, 40.77, 23.05, 22.85, 11.88.

N-pentylacetamide (20)[23]

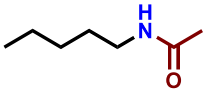

^{1}H NMR (300 MHz, DMSO) δ 7.81 (s, 1H), 3.00 (td, *J* = 7.0, 5.6 Hz, 2H), 1.77 (d, *J* = 5.7 Hz, 3H), 1.43 – 1.17 (m, 6H), 0.90 – 0.81 (m, 3H).

^{13}C NMR (75 MHz, DMSO) δ 169.36, 38.91, 29.28, 29.11, 23.04, 22.31, 14.35.

N-heptylacetamide (21)[24]

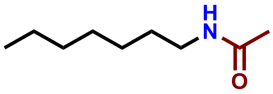

^{1}H NMR (300 MHz, DMSO) δ 7.77 (d, *J* = 5.8 Hz, 1H), 3.00 (td, *J* = 6.9, 5.6 Hz, 2H), 1.78 (s, 3H), 1.45 – 1.10 (m, 10H), 0.94 – 0.75 (m, 3H).

^{13}C NMR (75 MHz, DMSO) δ 169.30, 38.95, 31.74, 29.63, 28.92, 26.90, 23.02, 22.53, 14.37.

N-octadecylacetamide (22)[25]

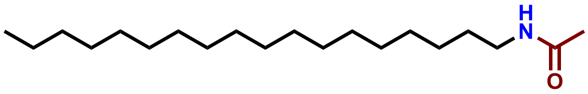

^{1}H NMR (300 MHz, $CDCl_3$) δ 5.77 (s, 1H), 3.22 (td, *J* = 7.2, 5.7 Hz, 2H), 1.97 (s, 3H), 1.48 (q, *J* = 7.0 Hz, 2H), 1.36 – 1.21 (m, 30H), 0.92 – 0.83 (m, 3H).

^{13}C NMR (75 MHz, $CDCl_3$) δ 170.07, 39.72, 31.93, 29.70, 29.66, 29.60, 29.56, 29.37, 29.32, 26.95, 23.29, 22.69, 14.12.

N-(4-acetylphenyl)acetamide (23)[26]

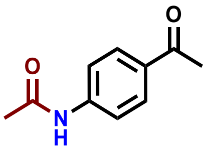

^{1}H NMR (300 MHz, DMSO) δ 10.28 (s, 1H), 7.95 – 7.88 (m, 2H), 7.74 – 7.69 (m, 2H), 2.52 (s, 3H), 2.09 (s, 3H).

^{13}C NMR (75 MHz, DMSO) δ 196.94, 169.41, 144.11, 131.96, 129.94, 118.59, 26.87, 24.64.

N-(3-acetyl-5-ethyl-2-hydroxyphenyl)acetamide (24)

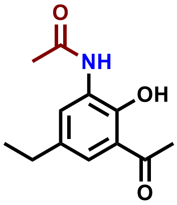

^{1}H NMR (300 MHz, DMSO) δ 12.54 (s, 1H), 9.34 (s, 1H), 8.04 (d, J = 2.1 Hz, 1H), 7.51 (d, J = 2.1 Hz, 1H), 2.67 (s, 3H), 2.62 – 2.52 (m, 2H), 2.11 (s, 3H), 1.21 – 1.14 (m, 3H).

^{13}C NMR (75 MHz, DMSO) δ 206.18, 169.35, 150.98, 134.10, 128.77, 127.68, 125.29, 119.66, 28.03, 27.63, 24.17, 16.18.

HRMS (ESI) expected mass for C12H15NO3 $[M+H]^+$: 222.1161, Observed mass: 221.1163.

5-acetamido-2-chlorobenzoic acid (25)

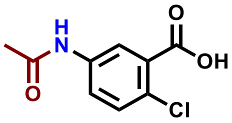

^{1}H NMR (400 MHz, DMSO) δ 10.24 (s, 1H), 7.95 (d, *J* = 2.6 Hz, 1H), 7.82 – 7.55 (m, 1H), 7.36 (d, *J* = 8.7 Hz, 1H), 2.05 (s, 3H).

^{13}C NMR (101 MHz, DMSO) δ 169.09, 168.22, 138.50, 135.23, 130.81, 124.88, 121.78, 120.85, 24.44.

HRMS (ESI) expected mass for C9H8ClNO3 $[M+H]^+$: 214.0271, Observed mass: 214.0276.

2-(4-acetamidophenyl)propanoic acid (26)[27]

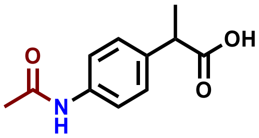

^{1}H NMR (300 MHz, DMSO) δ 9.97 (s, 1H), 7.51 (d, J = 8.3 Hz, 2H), 7.20 (d, J = 8.5 Hz, 2H), 3.57 (s, 1H), 2.03 (d, J = 1.5 Hz, 3H), 1.33 (d, J = 6.6 Hz, 3H).

^{13}C NMR (75 MHz, DMSO) δ 176.51, 168.66, 138.35, 136.70, 128.06, 119.52, 45.00, 24.38, 19.11.

ethyl 4-acetamidobenzoate (27)[28]

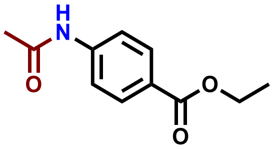

^{1}H NMR (400 MHz, DMSO) δ 10.27 (s, 1H), 7.98 – 7.79 (m, 2H), 7.77 – 7.65 (m, 2H), 4.28 (q, *J* = 7.1 Hz, 2H), 2.09 (s, 3H), 1.30 (t, *J* = 7.1 Hz, 3H).

^{13}C NMR (101 MHz, DMSO) δ 169.38, 165.80, 144.11, 130.66, 124.43, 118.70, 60.83, 24.61, 14.66.

ethyl 3-(4-acetamidophenyl)propanoate (28)

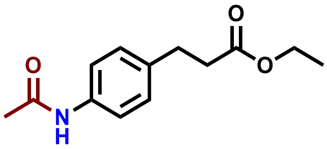

^{1}H NMR (300 MHz, $CDCl_3$) δ 8.15 (s, 1H), 7.47 – 7.38 (m, 2H), 7.17 – 7.06 (m, 2H), 4.12 (q, *J* = 7.1 Hz, 2H), 2.90 (t, *J* = 7.7 Hz, 2H), 2.64 – 2.52 (m, 2H), 2.12 (s, 3H), 1.23 (t, *J* = 7.1 Hz, 3H).

^{13}C NMR (75 MHz, $CDCl_3$) δ 173.04, 168.90, 136.40, 136.37, 128.70, 120.29, 60.50, 35.98, 30.37, 24.33, 14.20.

HRMS (ESI) expected mass for C13H17NO3 $[M+H]^+$: 236.1286, Observed mass: 236.1281.

methyl 4-acetamidobenzoate (29)

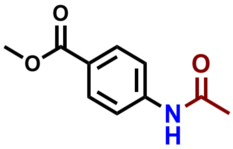

^{1}H NMR (300 MHz, $CDCl_3$) δ 8.02 – 7.95 (m, 2H), 7.66 – 7.58 (m, 2H), 3.91 (s, 3H), 2.21 (s, 3H).

^{13}C NMR (75 MHz, $CDCl_3$) δ 168.93, 166.75, 142.30, 130.79, 125.49, 118.88, 52.06, 24.68.

HRMS (ESI) expected mass for C10H11NO3 $[M+H]^+$: 194.0817, Observed mass: 194.0819.

N-(4-cyanophenyl)acetamide (30)[29]

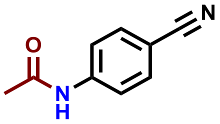

^{1}H NMR (300 MHz, DMSO) δ 10.38 (s, 1H), 7.76 (s, 4H), 2.09 (s, 3H).

^{13}C NMR (75 MHz, $CDCl_3$)δ 169.66, 143.93, 133.72, 119.56, 119.38, 105.15, 24.66.

N-(2-oxo-2H-chromen-6-yl)acetamide (31)[30]

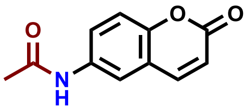

^{1}H NMR (300 MHz, DMSO) δ 10.16 (s, 1H), 8.14 – 7.95 (m, 2H), 7.64 (dd, *J* = 9.0, 2.6 Hz, 1H), 7.36 (d, *J* = 8.9 Hz, 1H), 6.48 (d, *J* = 9.6 Hz, 1H), 2.07 (s, 3H).

^{13}C NMR (75 MHz, DMSO) δ 168.93, 160.51, 149.68, 144.88, 136.21, 123.62, 119.12, 117.97, 117.08, 116.95, 24.37.

N-(quinolin-8-yl)acetamide (32)[31]

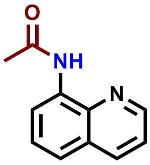

^{1}H NMR (300 MHz, $CDCl_3$) δ 9.80 (s, 1H), 8.91 – 8.67 (m, 2H), 8.16 (dd, *J* = 8.3, 1.7 Hz, 1H), 7.58 – 7.41 (m, 3H), 2.36 (s, 3H).

^{13}C NMR (75 MHz, $CDCl_3$) δ 168.81, 148.11, 138.22, 136.42, 134.51, 127.93, 127.41, 121.60, 121.48, 116.45, 25.13.

N-(pyridin-3-yl)acetamide (33)[32]

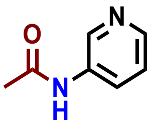

^{1}H NMR (300 MHz, CDCl3) δ 8.62 (d, J = 2.6 Hz, 1H), 8.35 – 8.13 (m, 2H), 7.28 – 7.23 (m, 1H), 2.19 (d, J = 1.4 Hz, 3H).

^{13}C NMR (75 MHz, CDCl3) δ 169.67, 144.39, 140.84, 135.68, 127.64, 123.92, 24.21.

N-(2-(4-fluorophenoxy)pyridin-3-yl)acetamide (34)

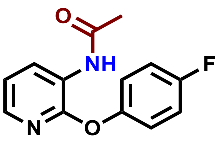

^{1}H NMR (300 MHz, CDCl3) δ 9.71 (s, 1H), 8.43 (dd, J = 7.8, 1.8 Hz, 1H), 7.79 (dd, J = 4.8, 1.8 Hz, 1H), 7.30 – 7.15 (m, 4H), 7.09 (ddd, J = 7.9, 4.9, 0.4 Hz, 1H), 2.15 (s, 3H).

^{13}C NMR (75 MHz, CDCl3) δ 169.91, 160.96, 150.14, 141.20, 130.90, 123.96, 123.84, 123.73, 119.43, 116.61, 116.30, 24.24.

HRMS (ESI) expected mass for C13H11FN2O2 $[M+H]^+$: 247.0883, Observed mass: 247,0880.

N-(1H-indol-5-yl)acetamide (35)[17]

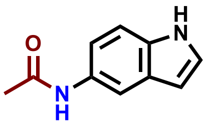

^{1}H NMR (300 MHz, DMSO) δ 10.97 (s, 1H), 9.72 (s, 1H), 7.91 – 7.84 (m, 1H), 7.35 – 7.26 (m, 2H), 7.20 (dd, *J* = 8.7, 2.0 Hz, 1H), 6.38 (ddd, *J* = 3.0, 2.0, 0.9 Hz, 1H), 2.05 (s, 3H).

^{13}C NMR (75 MHz, DMSO) δ 168.10, 133.07, 131.80, 127.91, 126.23, 115.29, 111.54, 111.09, 101.53, 24.37.

N-(benzo[d]thiazol-6-yl)acetamide (36)

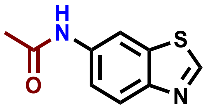

^{1}H NMR (400 MHz, DMSO) δ 10.22 (s, 1H), 9.25 (s, 1H), 8.55 (d, *J* = 2.0 Hz, 1H), 8.01 (dd, *J* = 8.8, 0.5 Hz, 1H), 7.57 (dd, *J* = 8.8, 2.1 Hz, 1H), 2.10 (s, 3H).

^{13}C NMR (101 MHz, DMSO) δ 169.03, 154.94, 149.43, 137.54, 134.69, 123.39, 119.05, 111.83, 24.52.

HRMS (ESI) expected mass for C9H8N2OS $[M+H]^+$: 193.0435, Observed mass: 193.0439.

N-(3-vinylphenyl)acetamide (37)[33]

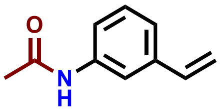

^{1}H NMR (400 MHz, DMSO) δ 9.95 (s, 1H), 7.68 (t, *J* = 2.0 Hz, 1H), 7.48 (ddd, *J* = 8.1, 2.2, 1.1 Hz, 1H), 7.27 (t, *J* = 7.8 Hz, 1H), 7.15 (dt, *J* = 7.8, 1.5 Hz, 1H), 6.70 (dd, *J* = 17.6, 10.9 Hz, 1H), 5.75 (dd, *J* = 17.6, 1.0 Hz, 1H), 5.26 (dd, *J* = 10.8, 1.0 Hz, 1H), 2.05 (s, 3H).

^{13}C NMR (101 MHz, DMSO) δ 168.84, 140.08, 137.96, 137.20, 129.39, 121.37, 119.15, 117.09, 114.74, 24.48.

N-phenylpropionamide (38)[24]

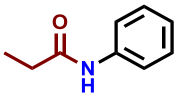

^{1}H NMR (300 MHz, DMSO) δ 9.91 (s, 1H), 7.63 – 7.50 (m, 2H), 7.36 – 7.20 (m, 2H), 7.10 – 6.91 (m, 1H), 2.32 (q, *J* = 7.6 Hz, 2H), 1.09 (t, *J* = 7.5 Hz, 3H).

^{13}C NMR (75 MHz, DMSO) δ 172.45, 139.88, 129.08, 123.31, 119.46, 29.97, 10.14.

N-phenylhexanamide (39)[34]

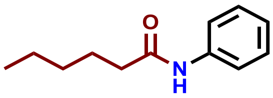

^{1}H NMR (400 MHz, DMSO) δ 9.85 (s, 1H), 7.64 – 7.57 (m, 2H), 7.31 – 7.25 (m, 2H), 7.05 – 6.98 (m, 1H), 2.30 (t, *J* = 7.5 Hz, 2H), 1.66 – 1.46 (m, 2H), 1.38 – 1.22 (m, 4H), 0.92 – 0.82 (m, 3H).

^{13}C NMR (101 MHz, DMSO) δ 171.73, 139.83, 129.07, 123.35, 119.48, 36.86, 34.10, 31.38, 31.24, 25.29, 24.66, 22.39, 22.31, 14.32, 14.27.

3-mercapto-N-phenylpropanamide (40)

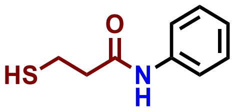

^{1}H NMR (300 MHz, $CDCl_3$) δ 8.05 (s, 1H), 7.60 – 7.46 (m, 2H), 7.37 – 7.23 (m, 2H), 7.17 – 7.07 (m, 1H), 2.89 – 2.63 (m, 4H), 1.67 (d, *J* = 16.4 Hz, 1H).

^{13}C NMR (75 MHz, $CDCl_3$) δ 169.70, 137.64, 129.03, 124.64, 120.36, 41.22, 20.40.

HRMS (ESI) expected mass for C9H11NOS $[M+H]^+$: 182.0644, Observed mass: 182.0640.

N-phenylcyclopropanecarboxamide (41)[35]

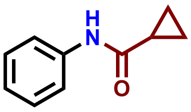

^{1}H NMR (300 MHz, DMSO) δ 10.18 (s, 1H), 7.64 – 7.51 (m, 2H), 7.38 – 7.24 (m, 2H), 7.11 – 6.92 (m, 1H), 1.81 – 1.73 (m, 1H), 0.91 – 0.72 (m, 4H).

^{13}C NMR (75 MHz, DMSO) δ 172.09, 139.82, 129.13, 123.36, 119.44, 14.99, 7.57.

N-phenylcyclobutanecarboxamide (42)[36]

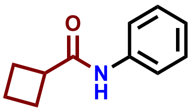

^{1}H NMR (300 MHz, DMSO) δ 9.71 (s, 1H), 7.71 – 7.57 (m, 2H), 7.36 – 7.17 (m, 2H), 7.10 – 6.91 (m, 1H), 3.36 – 3.18 (m, 1H), 2.33 – 2.18 (m, 2H), 2.18 – 2.06 (m, 2H), 1.99 – 1.73 (m, 2H).

^{13}C NMR (75 MHz, DMSO) δ 173.37, 139.86, 129.02, 123.33, 119.62, 37.92, 25.09, 18.23.

2,2,2-trifluoro-N-phenylacetamide (43)[37]

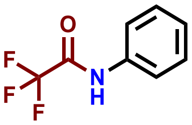

^{1}H NMR (300 MHz, DMSO) δ 11.25 (s, 1H), 7.72 – 7.65 (m, 2H), 7.46 – 7.36 (m, 2H), 7.26 – 7.19 (m, 1H).

^{13}C NMR (75 MHz, DMSO) δ 154.53 (q, J = 36.9 Hz), 136.35, 129.01, 125.67, 121.10, 115.84 (q, J = 288.8 Hz).

2-cyano-N-phenylacetamide (44)[38]

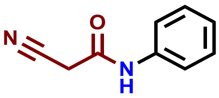

^{1}H NMR (300 MHz, DMSO) δ 10.29 (s, 1H), 7.59 – 7.51 (m, 2H), 7.39 – 7.29 (m, 2H), 7.14 – 7.06 (m, 1H), 3.90 (s, 2H).

^{13}C NMR (75 MHz, DMSO) δ 161.47, 138.83, 129.38, 124.37, 119.70, 116.40, 27.19.

ethyl 2-methyl-3-oxo-3-(phenylamino)propanoate (45)[39]

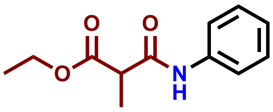

^{1}H NMR (300 MHz, $CDCl_3$) δ 8.78 (s, 1H), 7.61 – 7.51 (m, 2H), 7.38 – 7.22 (m, 2H), 7.12 (ddt, *J* = 7.9, 6.9, 1.2 Hz, 1H), 4.24 (q, *J* = 7.1 Hz, 2H), 3.48 (q, *J* = 7.3 Hz, 1H), 1.54 (d, *J* = 7.3 Hz, 3H), 1.30 (t, *J* = 7.1 Hz, 3H).

^{13}C NMR (75 MHz, $CDCl_3$) δ 172.48, 167.41, 137.69, 128.97, 124.51, 120.06, 61.81, 47.52, 15.20, 14.07.

N,2-diphenylacetamide (46)[20]

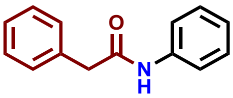

^{1}H NMR (400 MHz, DMSO) δ 10.17 (s, 1H), 7.66 – 7.55 (m, 2H), 7.39 – 7.23 (m, 7H), 7.07 – 7.01 (m, 1H), 3.65 (s, 2H).

^{13}C NMR (101 MHz, DMSO) δ 169.56, 139.69, 136.48, 129.57, 129.26, 129.18, 128.77, 126.99, 123.67, 119.57, 43.81.

2-cyano-N,2-diphenylacetamide (47)

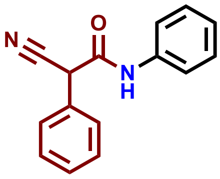

^{1}H NMR (300 MHz, DMSO) δ 10.59 (s, 1H), 7.60 – 7.53 (m, 4H), 7.48 – 7.40 (m, 3H), 7.37 – 7.30 (m, 2H), 7.11 (ddt, *J* = 7.8, 7.0, 1.2 Hz, 1H), 5.31 (s, 1H).

^{13}C NMR (75 MHz, DMSO) δ 163.19, 138.58, 132.59, 129.66, 129.41, 129.20, 128.22, 124.77, 119.99, 118.15, 45.00.

HRMS (ESI) expected mass for C15H12N2O $[M+H]^{+}$: 237.1028, Observed mass: 237.1029.

dimethyl 3,3'-(1,4-phenylenebis(azanediyl))bis(3-oxopropanoate) (48)

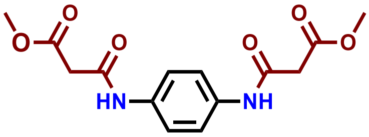

^{1}H NMR (300 MHz, DMSO) δ 10.16 (s, 2H), 7.51 (s, 4H), 3.66 (s, 6H), 3.46 (s, 4H).

^{13}C NMR (75 MHz, DMSO) δ 168.64, 164.16, 134.97, 119.99, 52.42, 43.85.

HRMS (ESI) expected mass for C14H16N2O6 $[M+H]^{+}$: 309.1086, Observed mass: 309.1091.

N-phenylbenzamide (49)[40]

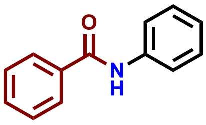

^{1}H NMR (300 MHz, DMSO) δ 10.26 (s, 1H), 8.02 – 7.92 (m, 2H), 7.85 – 7.74 (m, 2H), 7.65 – 7.48 (m, 3H), 7.43 – 7.29 (m, 2H), 7.17 – 7.06 (m, 1H).

^{13}C NMR (75 MHz, DMSO) δ 166.03, 139.66, 135.47, 132.01, 129.07, 128.85, 128.12, 124.12, 120.83.

N-(4-cyanophenyl)benzamide (50)[41]

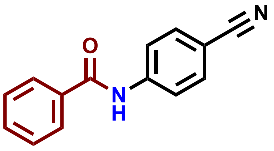

^{1}H NMR (300 MHz, DMSO) δ 8.00 – 7.93 (m, 2H), 7.90 – 7.87 (m, 1H), 7.65 – 7.59 (m, 1H), 7.59 – 7.52 (m, 1H), 7.51 – 7.42 (m, 3H), 6.58 – 6.52 (m, 1H).

^{13}C NMR (75 MHz, DMSO) δ 167.83, 153.47, 133.91, 133.30, 131.29, 129.73, 129.02, 121.15, 113.94, 96.03.

4-fluoro-N-phenylbenzamide (51)[35]

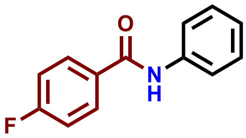

^{1}H NMR (300 MHz, DMSO) δ 8.07 – 7.96 (m, 3H), 7.39 – 7.28 (m, 3H), 7.05 – 6.96 (m, 1H), 6.58 – 6.43 (m, 2H).

^{13}C NMR (75 MHz, DMSO) δ 166.85, 163.72, 149.06, 132.64, 132.51, 129.26, 129.08, 127.86, 120.88, 116.23, 116.09, 115.94, 114.33.

N-phenylfuran-3-carboxamide (52)[42]

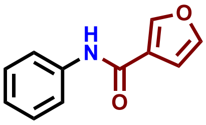

^{1}H NMR (300 MHz, $CDCl_3$) δ 8.04 (dd, J = 1.6, 0.8 Hz, 2H), 7.55 – 7.48 (m, 1H), 7.38 (dd, J = 1.9, 1.5 Hz, 2H), 7.32 – 7.23 (m, 1H), 6.70 (dd, J = 1.9, 0.8 Hz, 2H).

^{13}C NMR (75 MHz, $CDCl_3$) δ 168.54, 149.08, 145.35, 144.07, 129.10, 126.51, 118.81, 109.90.

N-phenylthiophene-3-carboxamide (53)[43]

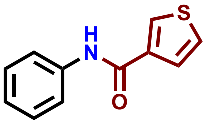

^{1}H NMR (300 MHz, $CDCl_3$) δ 8.24 (dd, *J* = 3.1, 1.2 Hz, 1H), 8.00 (dd, *J* = 3.0, 1.4 Hz, 1H), 7.59 (dd, *J* = 5.1, 1.2 Hz, 2H), 7.44 (dd, *J* = 5.1, 1.4 Hz, 1H), 7.40 – 7.38 (m, 1H), 7.37 – 7.33 (m, 2H).
^{13}C NMR (75 MHz, $CDCl_3$) δ 167.30, 136.01, 134.17, 133.31, 129.92, 128.16, 126.86, 126.27, 126.25.

methyl 5-(phenylcarbamoyl)furan-2-carboxylate (54)

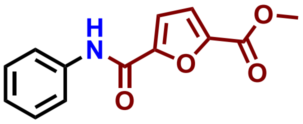

^{1}H NMR (300 MHz, $CDCl_3$) δ 8.44 (s, 1H), 7.73 – 7.64 (m, 2H), 7.41 – 7.33 (m, 2H), 7.28 (d, *J* = 3.6 Hz, 1H), 7.24 (d, *J* = 3.6 Hz, 1H), 7.21 – 7.13 (m, 1H), 3.94 (s, 3H).
^{13}C NMR (75 MHz, $CDCl_3$) δ 158.61, 155.25, 150.14, 144.98, 136.99, 129.12, 124.98, 120.28, 119.32, 116.11, 52.41.
HRMS (ESI) expected mass for C13H11NO4 $[M+H]^+$: 246.0766, Observed mass: 246.0770.

Acedoben (55)[44]

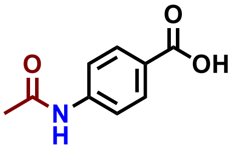

^{1}H NMR (400 MHz, DMSO) δ 12.96 (s, 1H)10.12 (s, 1H), 8.22 (t, *J* = 1.9 Hz, 1H), 7.81 (ddd, *J* = 8.1, 2.3, 1.1 Hz, 1H), 7.63 – 7.58 (m, 1H), 7.41 (t, *J* = 7.9 Hz, 1H), 2.06 (s, 3H).
^{13}C NMR (101 MHz, DMSO) δ 169.03, 167.67, 139.98, 131.70, 129.39, 124.27, 123.51, 120.16, 24.46.

Actarit (56)[45]

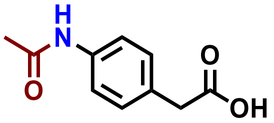

^{1}H NMR (300 MHz, DMSO) δ 9.92 (s, 1H), 7.57 – 7.46 (m, 2H), 7.20 – 7.13 (m, 2H), 3.45 (s, 2H), 2.03 (s, 3H).
^{13}C NMR (75 MHz, DMSO) δ 174.08, 168.62, 138.12, 130.81, 129.98, 119.32, 41.46, 24.41.

Phenacetin (57)[46]

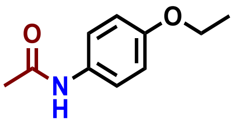

^{1}H NMR (300 MHz, $CDCl_3$) δ 7.40 – 7.36 (m, 2H), 6.85 – 6.78 (m, 2H), 4.00 (q, J = 7.0 Hz, 2H), 2.12 (s, 3H), 1.40 (t, J = 7.0 Hz, 3H).

^{13}C NMR (75 MHz, $CDCl_3$) δ 168.67, 155.75, 131.03, 122.04, 114.69, 63.69, 24.19, 14.83.

Propanil (58)[47]

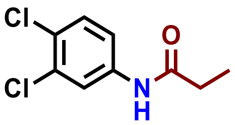

^{1}H NMR (300 MHz, $CDCl_3$) δ 7.69 (dd, J = 1.9, 1.0 Hz, 1H), 7.28 – 7.24 (m, 2H), 2.32 (q, J = 7.5 Hz, 2H), 1.16 (t, J = 7.5 Hz, 3H).

^{13}C NMR (75 MHz, $CDCl_3$) δ 172.31, 137.42, 132.74, 130.47, 127.33, 121.53, 119.03, 30.67, 9.51.

N-(4-acetylphenyl)-2-(4-chloro-2-methylphenoxy)acetamide (59)

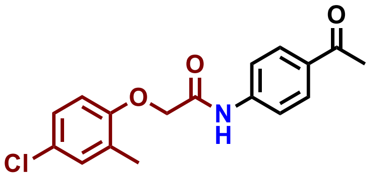

^{1}H NMR (300 MHz, $CDCl_3$) δ 8.53 (s, 1H), 8.08 – 7.91 (m, 2H), 7.82 – 7.65 (m, 3H), 6.76 (d, J = 8.6 Hz, 1H), 6.68 – 6.59 (m, 1H), 4.60 (s, 2H), 2.59 (s, 3H), 2.35 (s, 3H).

^{13}C NMR (75 MHz, $CDCl_3$) δ 196.98, 166.41, 153.73, 141.02, 131.10, 130.86, 129.83, 128.49, 127.02, 119.25, 113.16, 112.38, 68.05, 26.45, 16.30.

HRMS (ESI) expected mass for C17H16ClNO3 $[M+H]^{+}$: 318.0897, Observed mass: 318.0903.

N-(4-(3',6'-bis(diethylamino)-3-oxospiro[isoindoline-1,9'-xanthen]-2-yl)phenyl)acetamide (60)

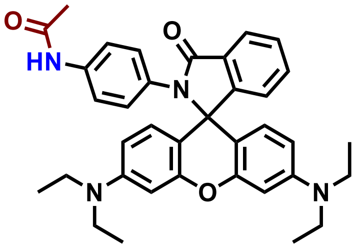

^{1}H NMR (300 MHz, DMSO) δ 9.94 (s, 1H), 7.96 (s, 1H), 7.81 – 7.73 (m, 2H), 7.62 – 7.53 (m, 2H), 7.49 – 7.41 (m, 1H), 7.35 – 7.18 (m, 2H), 7.01 – 6.87 (m, 3H), 6.42 – 6.27 (m, 4H), 5.39 (s, 1H), 3.30

(d, *J* = 7.1 Hz, 4H), 2.89 (d, *J* = 0.5 Hz, 2H), 2.74 (d, *J* = 0.6 Hz, 2H), 2.05 (s, 3H), 1.07 (t, *J* = 6.9 Hz, 12H).

^{13}C NMR (75 MHz, DMSO) δ 168.48, 162.77, 151.89, 148.78, 147.71, 147.23, 136.56, 135.77, 134.99, 131.15, 130.52, 126.74, 125.95, 120.68, 119.80, 111.68, 107.82, 98.35, 44.14, 40.81, 40.53, 40.25, 39.97, 39.70, 39.42, 39.14, 37.73, 36.24, 31.23, 24.38, 12.90.

HRMS (ESI) expected mass for C36H38N4O3 [M+H]$^+$: 575.3022, Observed mass: 575.3030.

N-(4-(methylsulfonamido)-3-phenoxyphenyl)acetamide (61)

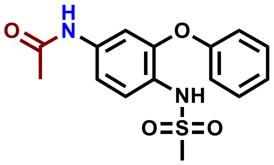

^{1}H NMR (300 MHz, DMSO) δ 9.99 (s, 1H), 9.22 (s, 1H), 7.47 – 7.39 (m, 2H), 7.31 (d, *J* = 1.3 Hz, 2H), 7.24 – 7.15 (m, 2H), 7.10 – 7.04 (m, 2H), 2.95 (s, 3H), 1.98 (d, *J* = 5.8 Hz, 3H).

^{13}C NMR (75 MHz, DMSO) δ 168.84, 156.44, 151.55, 138.86, 130.49, 128.44, 124.35, 122.85, 119.66, 114.30, 109.10, 40.81, 24.38.

HRMS (ESI) expected mass for C15H16N2O4S [M+H]$^+$: 321.0909, Observed mass: 321.0912.

3-isopropyl 5-(2-methoxyethyl) 4-(3-acetamidophenyl)-2,6-dimethyl-1,4dihydropyridine-3,5-dicarboxylate (62)

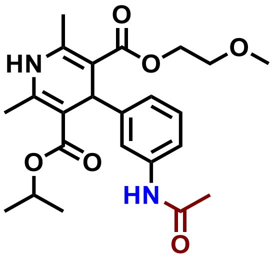

^{1}H NMR (300 MHz, DMSO) δ 10.05 (s, 1H), 7.66 – 7.48 (m, 2H), 7.33 (ddd, *J* = 8.1, 7.6, 0.5 Hz, 1H), 6.82 (ddd, *J* = 7.6, 1.7, 1.0 Hz, 1H), 4.84 (p, *J* = 6.2 Hz, 1H), 4.10 (t, *J* = 4.7 Hz, 2H), 3.32 – 3.25 (m, 2H), 3.15 (s, 3H), 2.05 (s, 3H), 0.95 (d, *J* = 6.3 Hz, 6H).

^{13}C NMR (75 MHz, DMSO) δ 168.90, 167.36, 166.83, 155.13 (d, *J* = 1.8 Hz), 145.52, 139.82, 136.50, 129.11, 127.09, 126.71, 122.84, 119.31, 118.65, 69.66, 69.40, 64.42, 58.34, 24.46, 22.84 (d, *J* = 2.4 Hz), 21.32.

HRMS (ESI) expected mass for C23H30N2O6 [M+H]$^+$: 431.2182, Observed mass: 431.2175.

3-ethyl 5-methyl 2-((2-acetamidoethoxy)methyl)-4-(2-chlorophenyl)-6-methyl-1,4-dihydropyridine-3,5-dicarboxylate (63)[48]

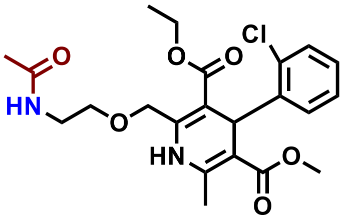

^{1}H NMR (400 MHz, DMSO) δ 8.52 (s, 1H), 8.07 (t, J = 5.7 Hz, 1H), 7.32 (dd, J = 7.8, 1.7 Hz, 1H), 7.29 – 7.20 (m, 2H), 7.12 (ddd, J = 7.9, 7.2, 1.8 Hz, 1H), 5.30 (s, 1H), 4.66 – 4.51 (m, 2H), 4.05 – 3.89 (m, 2H), 3.50 (s, 3H), 3.47 (td, J = 5.8, 2.0 Hz, 2H), 3.27 (qd, J = 5.6, 4.1 Hz, 2H), 2.31 (s, 3H), 1.84 (s, 3H), 1.10 (t, J = 7.1 Hz, 3H).

^{13}C NMR (101 MHz, DMSO) δ 170.02, 167.61, 166.78, 146.28, 145.93, 145.39, 131.54, 131.42, 129.43, 128.26, 127.91, 102.97, 102.20, 69.86, 66.77, 59.86, 50.97, 38.83, 37.17, 23.10, 18.66, 14.52.

3-ethyl 5-methyl 4-(2-chlorophenyl)-2-((2-(cyclobutanecarboxamido)ethoxy)methyl)-6-methyl-1,4-dihydropyridine-3,5-dicarboxylate (64)

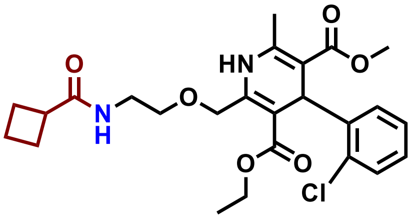

^{1}H NMR (300 MHz, $CDCl_3$) δ 7.39 (dd, *J* = 7.7, 1.8 Hz, 1H), 7.24 (dd, *J* = 7.8, 1.5 Hz, 1H), 7.14 (td, *J* = 7.5, 1.5 Hz, 1H), 7.05 (ddd, *J* = 7.8, 7.2, 1.8 Hz, 1H), 5.42 (s, 1H), 4.72 (qd, *J* = 15.7, 0.7 Hz, 2H), 4.06 (qd, *J* = 7.1, 2.0 Hz, 2H), 3.67 – 3.60 (m, 5H), 3.58 – 3.50 (m, 2H), 3.09 – 2.98 (m, 1H), 2.41 (d, *J* = 0.5 Hz, 3H), 2.35 – 2.10 (m, 6H), 1.19 (t, *J* = 7.1 Hz, 3H).

^{13}C NMR (75 MHz, $CDCl_3$) δ 175.68, 168.08, 167.21, 145.81, 145.05, 144.42, 132.32, 131.47, 129.22, 127.35, 126.85, 103.78, 101.61, 70.73, 68.00, 59.82, 50.77, 39.98, 39.10, 37.16, 25.41, 25.34, 25.23, 19.34, 18.15, 14.25.

HRMS (ESI) expected mass for C25H16ClNO3 $[M+H]^+$: 491.1949, Observed mass: 491.1953.

4. NMR Spectra.

Paracetamol (4)

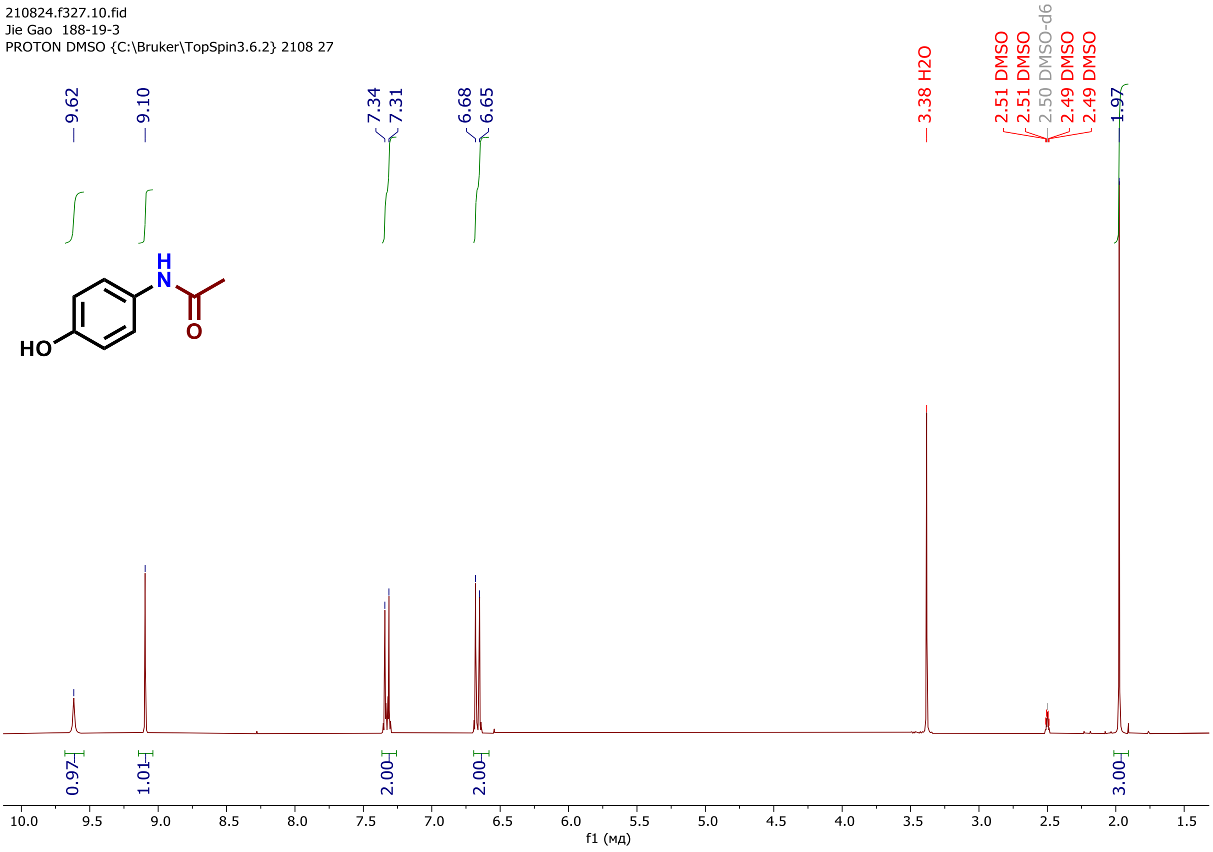

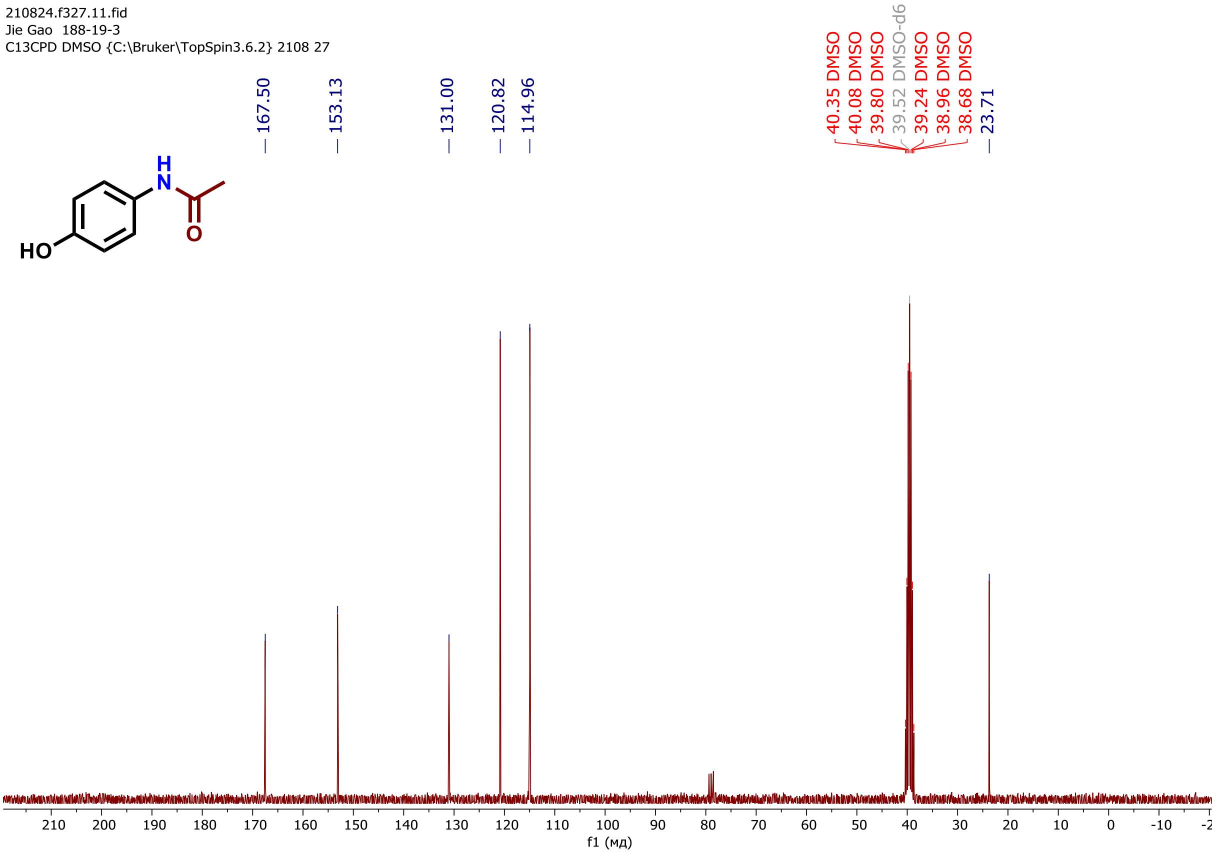

Supplementary Figure 26. NMR spectra of Paracetamol.

N-phenylacetamide (5)

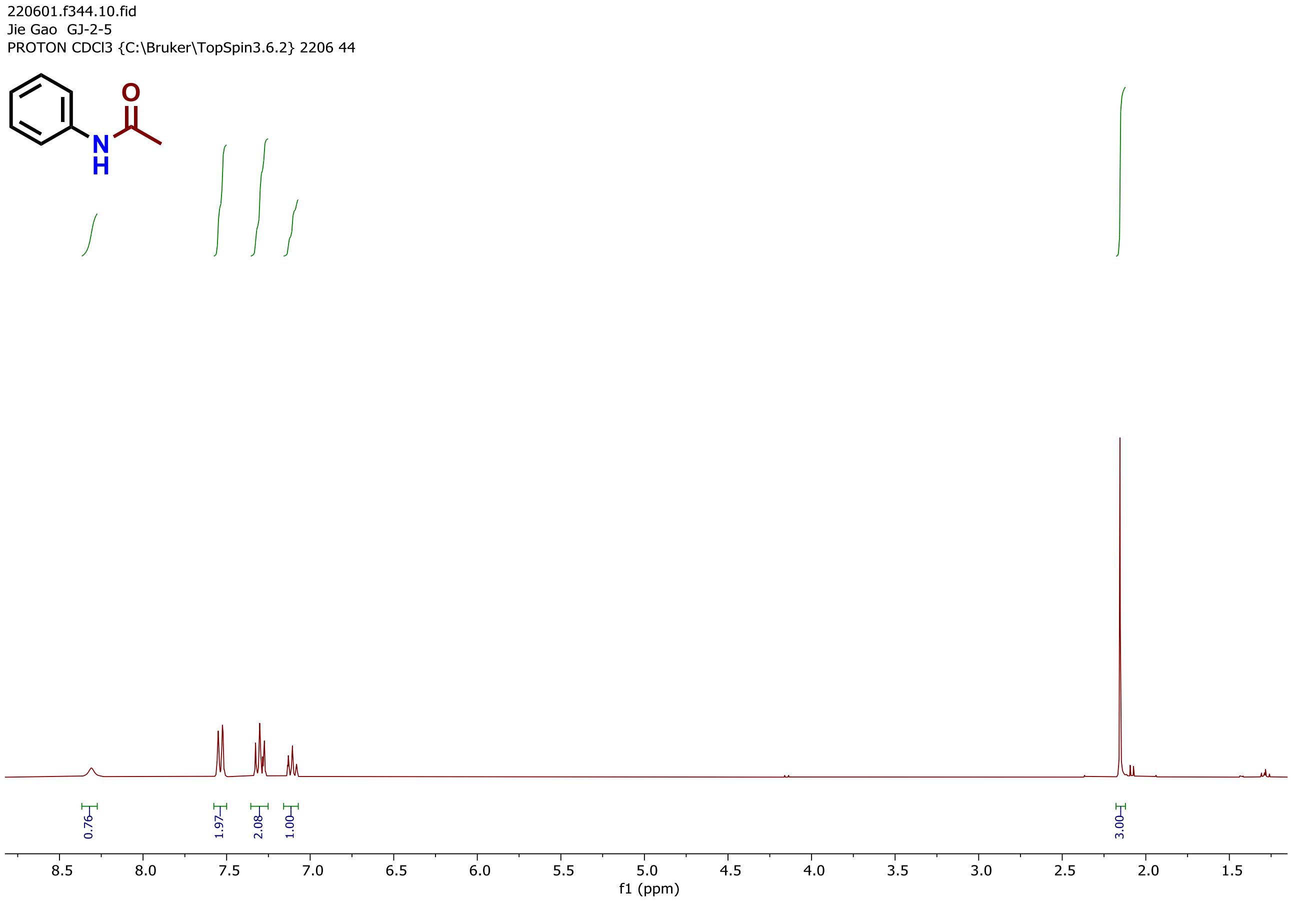

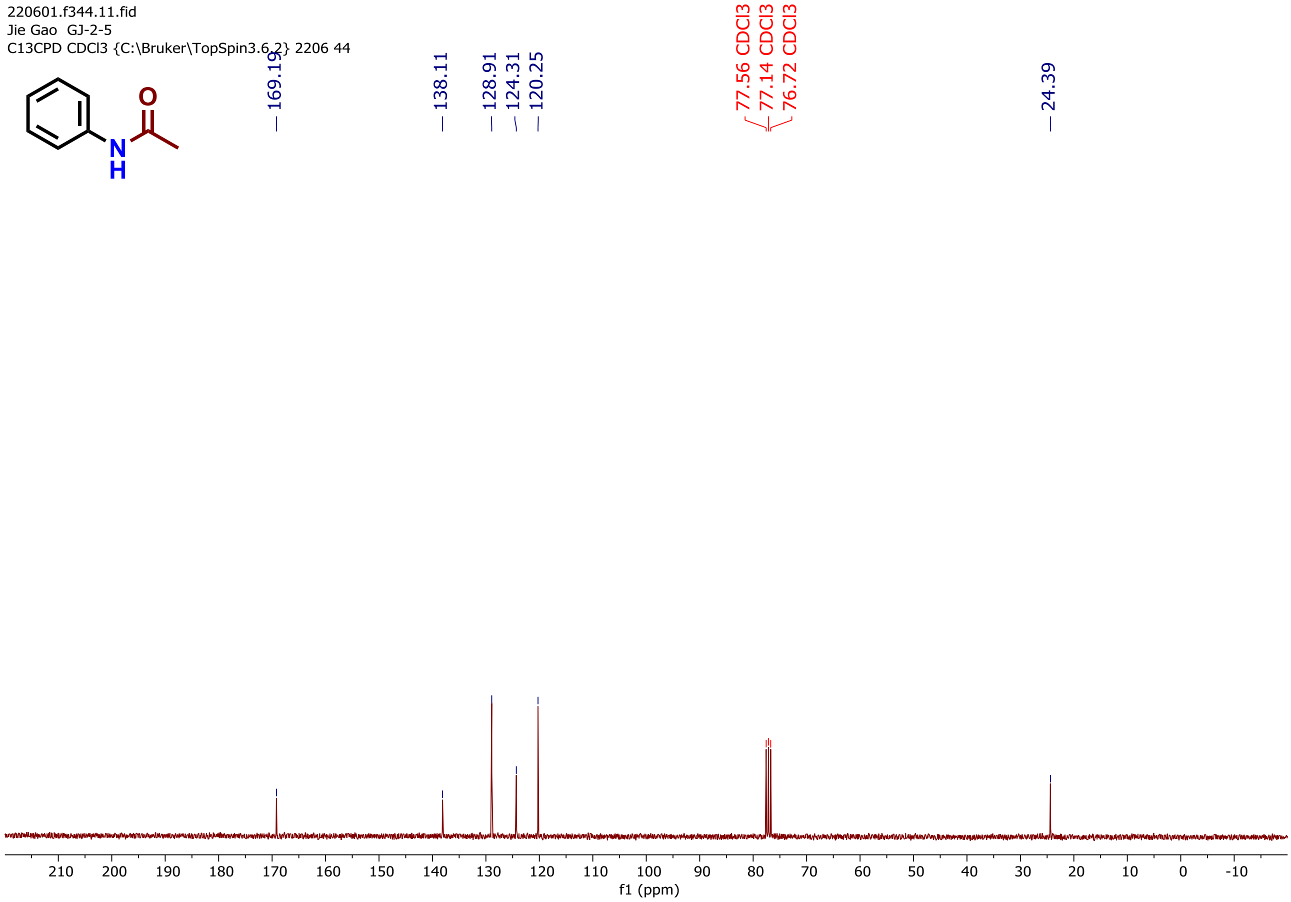

Supplementary Figure 27. NMR spectra of N-phenylacetamide.

N-(4-isopropylphenyl)acetamide (6)

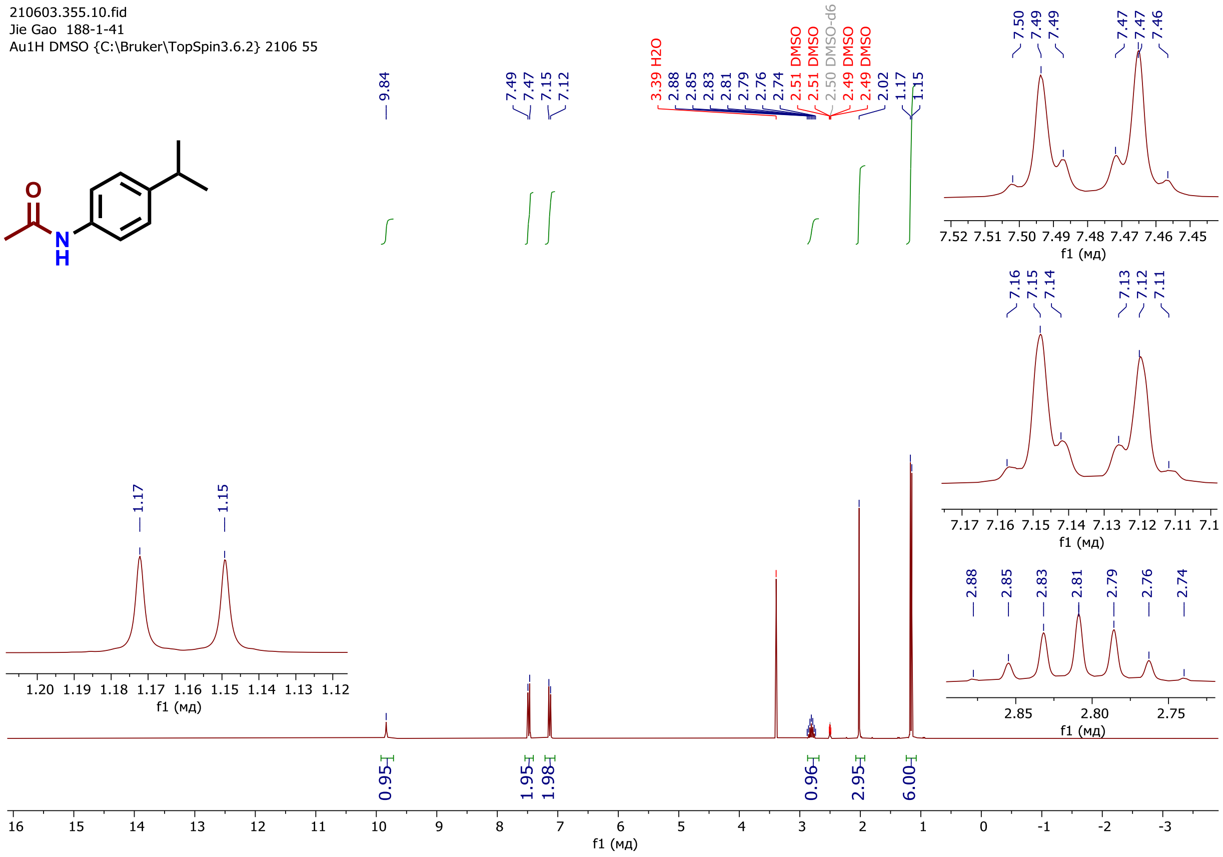

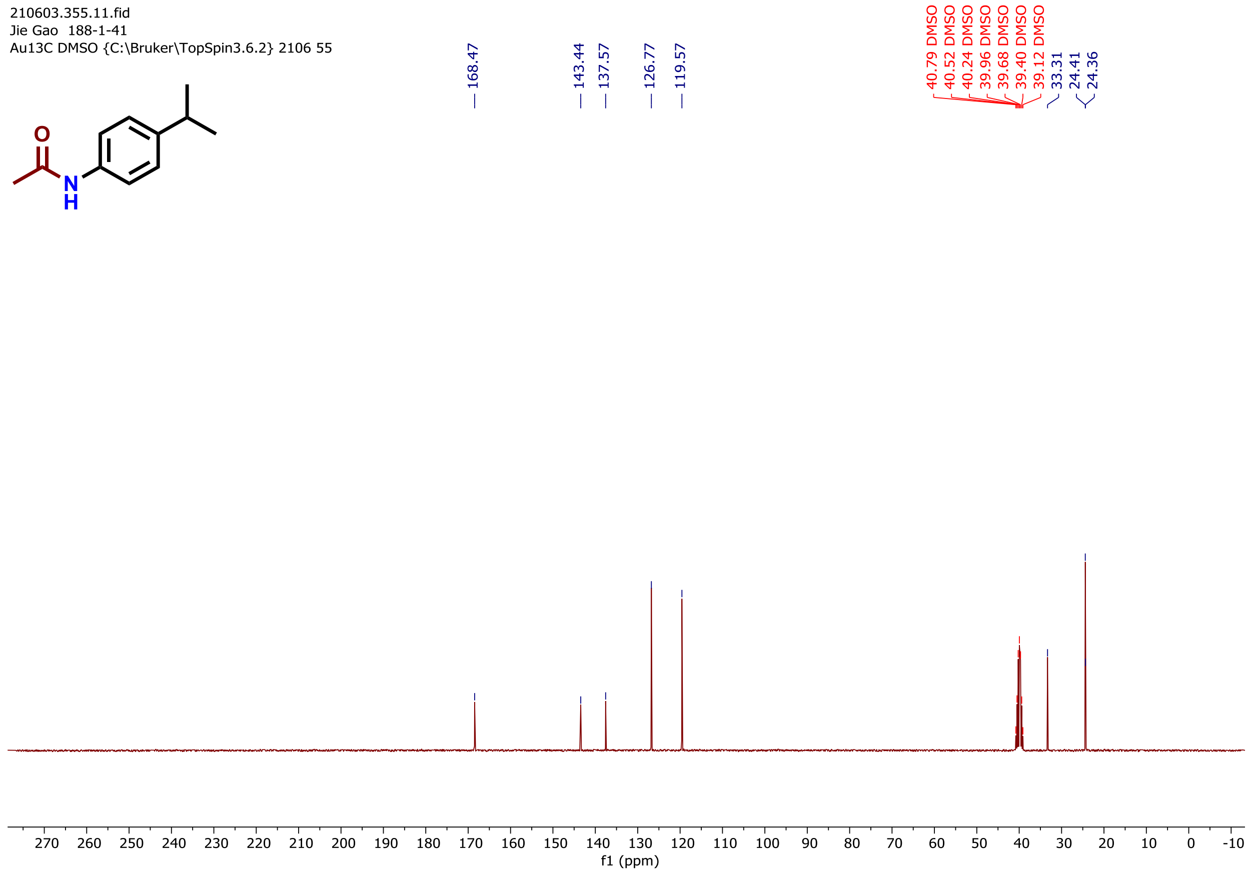

Supplementary Figure 28. NMR spectra of N-(4-isopropylphenyl)acetamide.

N-(naphthalen-1-yl)acetamide (7)

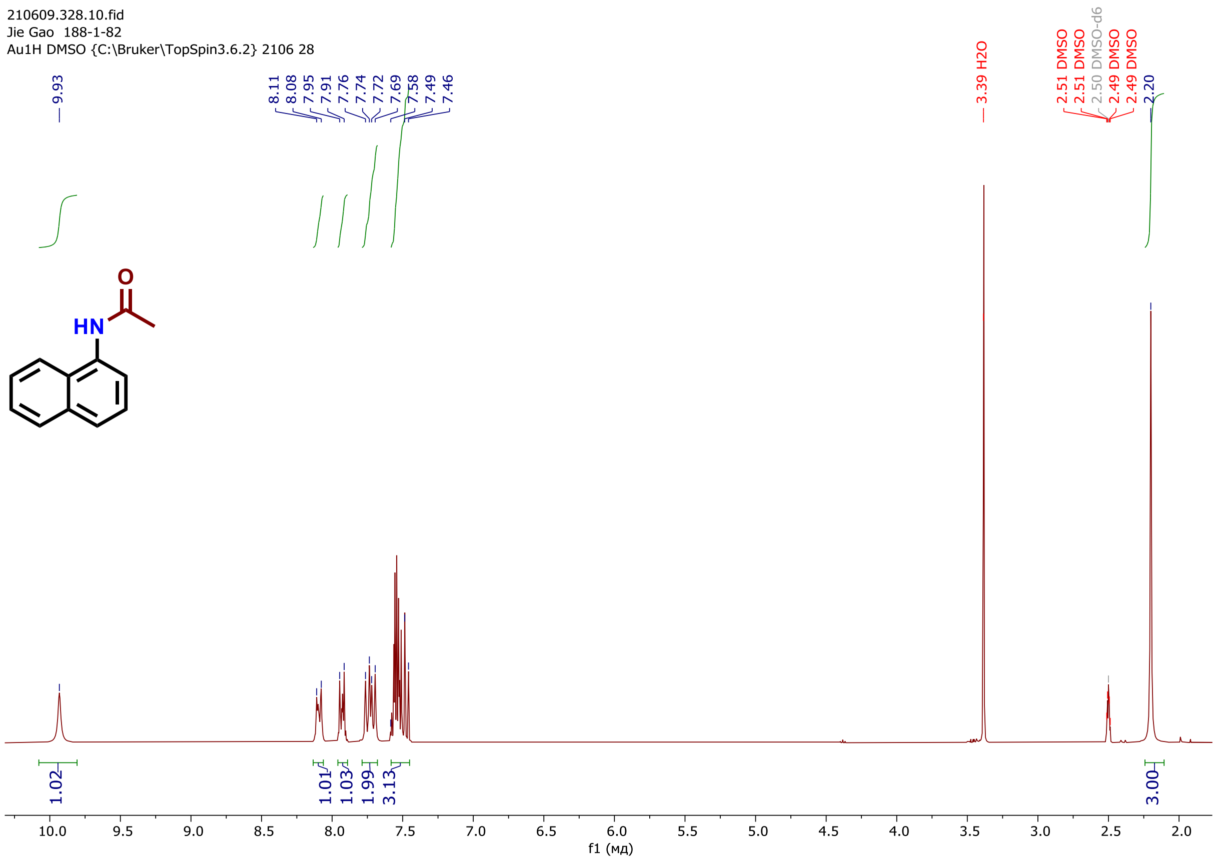

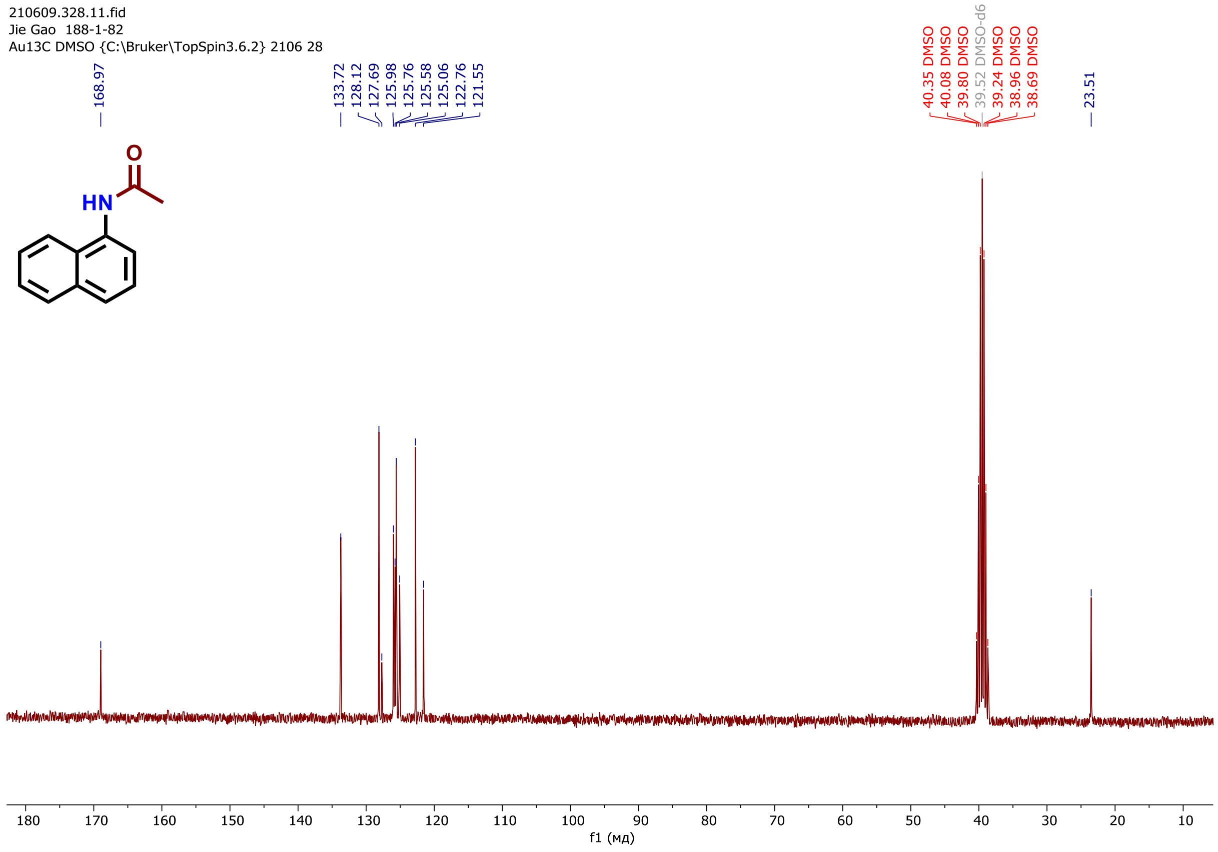

Supplementary Figure 29. NMR spectra of N-(naphthalen-1-yl)acetamide.

N-([1,1'-biphenyl]-2-yl)acetamide (8)

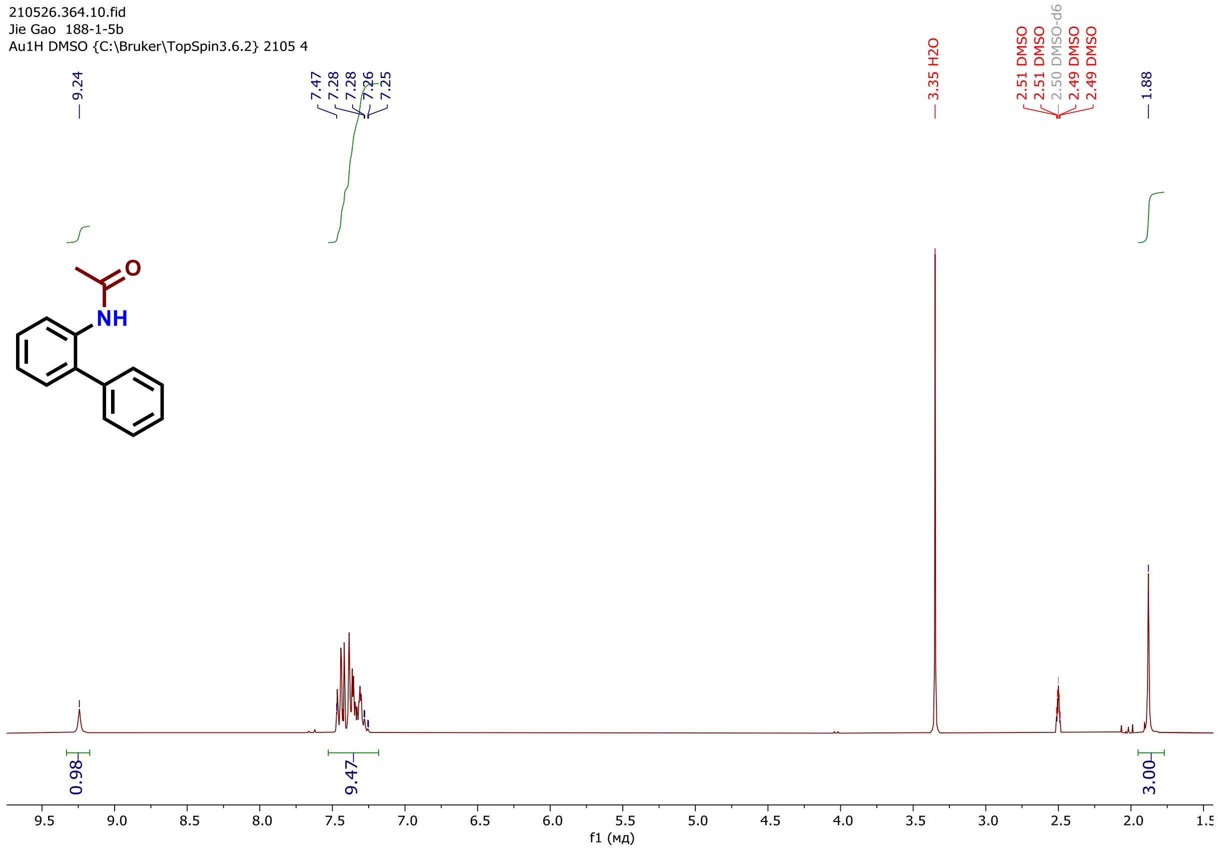

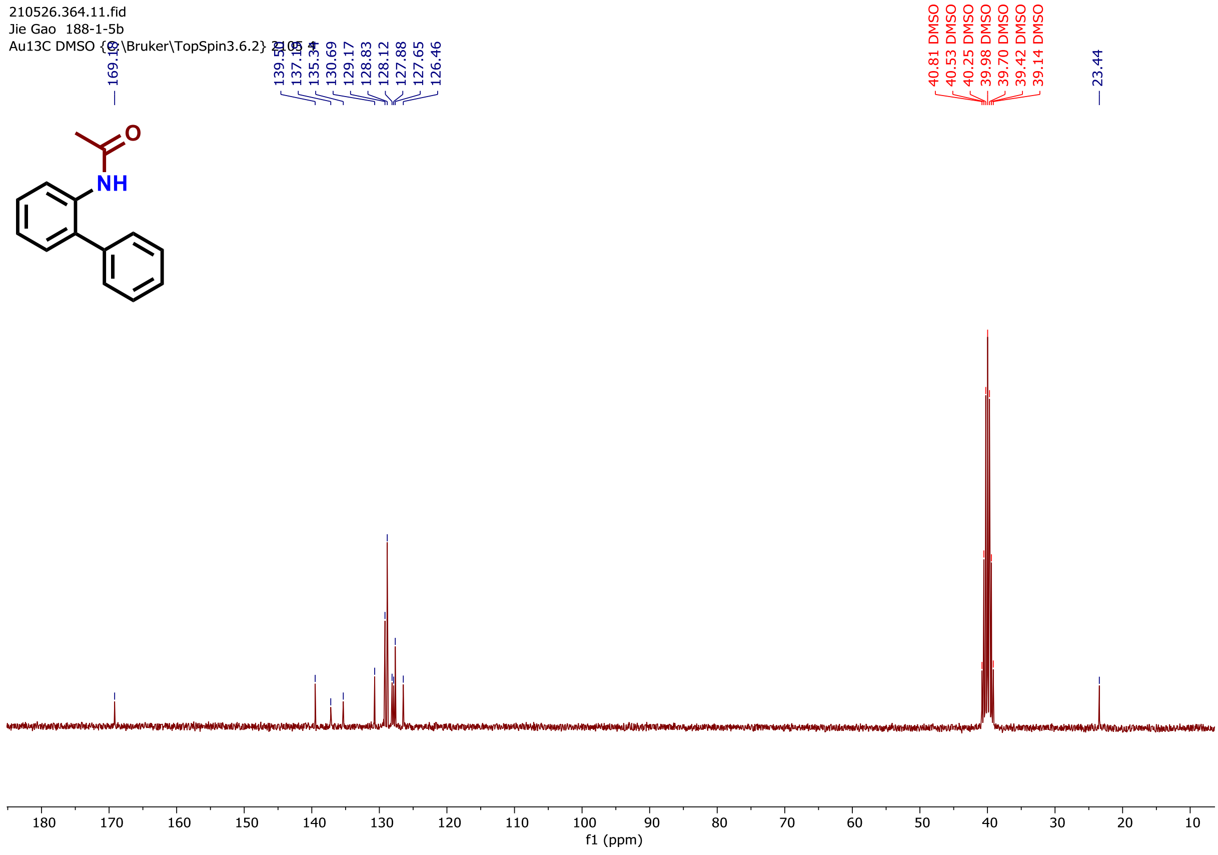

Supplementary Figure 30. NMR spectra of N-([1,1'-biphenyl]-2-yl)acetamide.

N-(9H-fluoren-2-yl)acetamide (9)

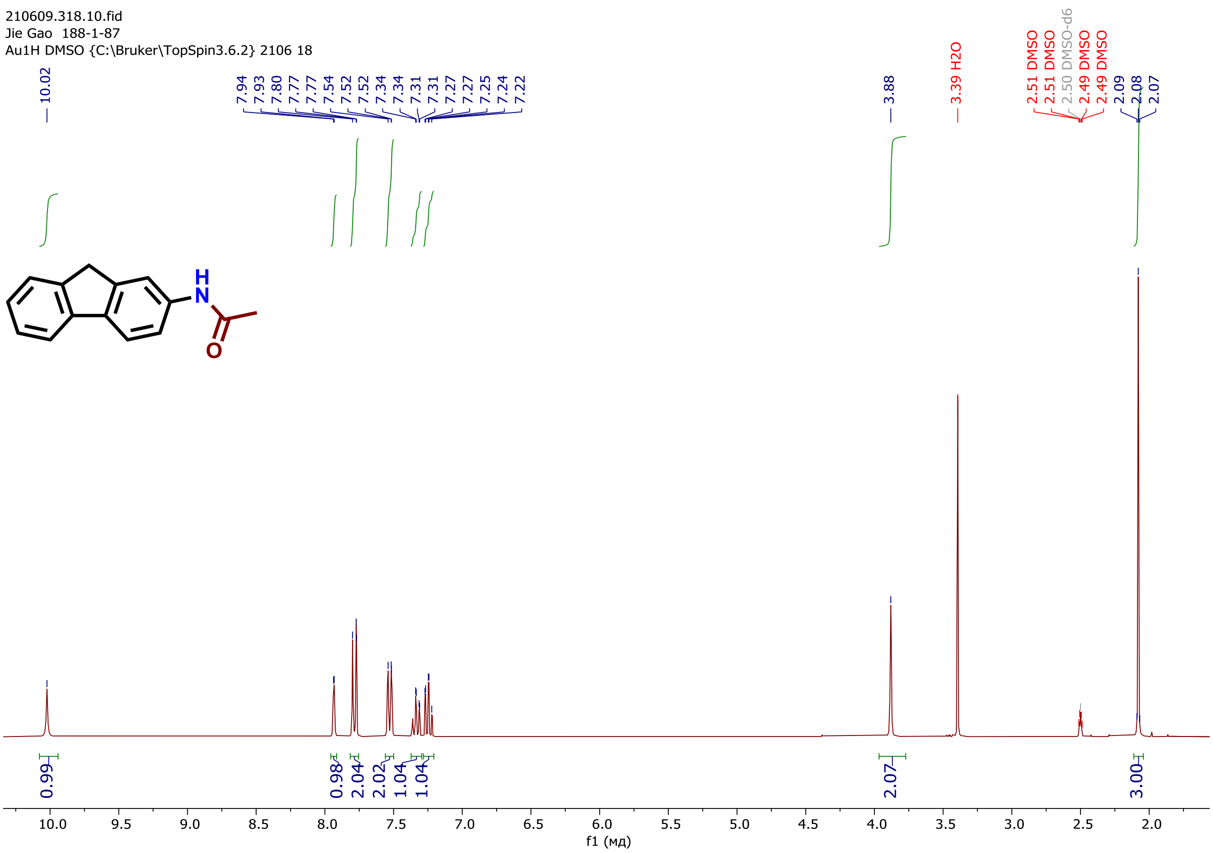

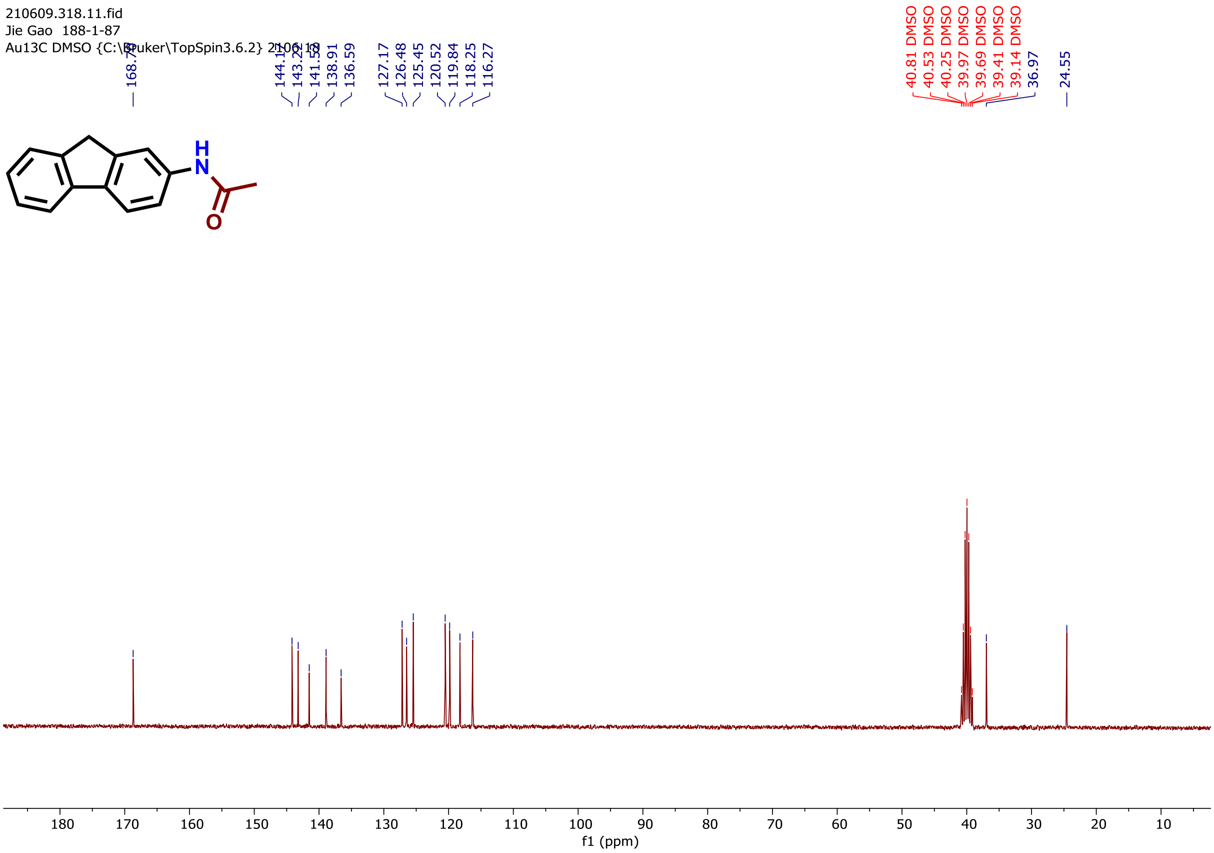

Supplementary Figure 31. NMR spectra of N-(9H-fluoren-2-yl)acetamide.

N-(4-(tert-butyl)phenyl)acetamide (10)

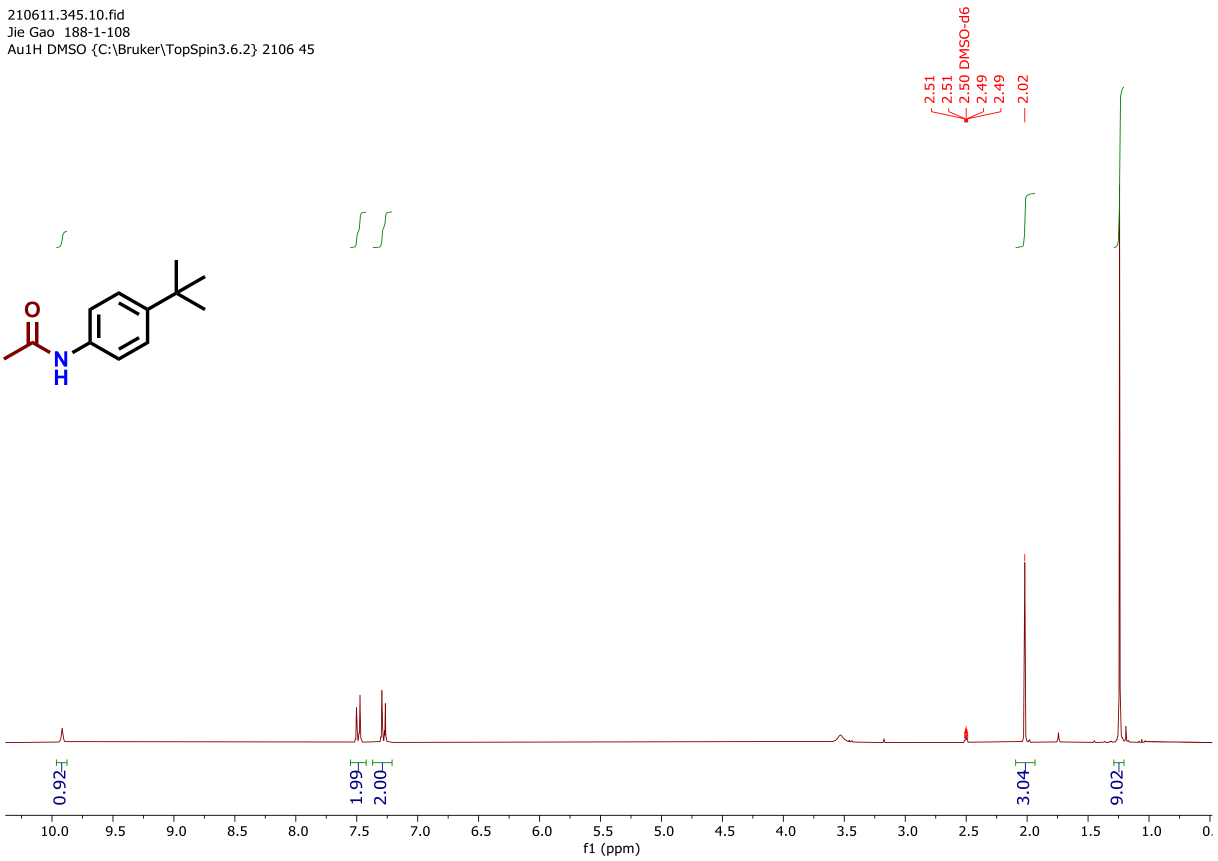

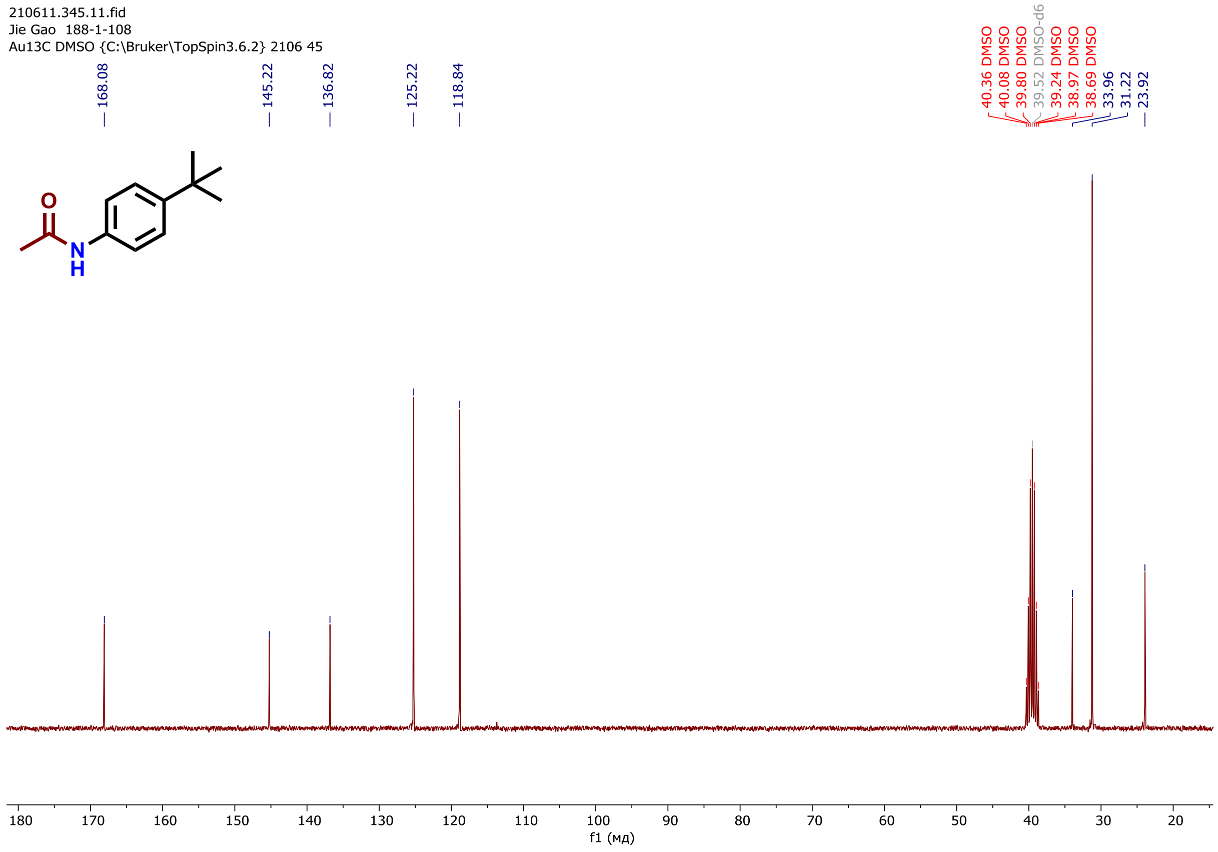

Supplementary Figure 32. NMR spectra of N-(4-(tert-butyl)phenyl)acetamide.

N-mesitylacetamide (11)

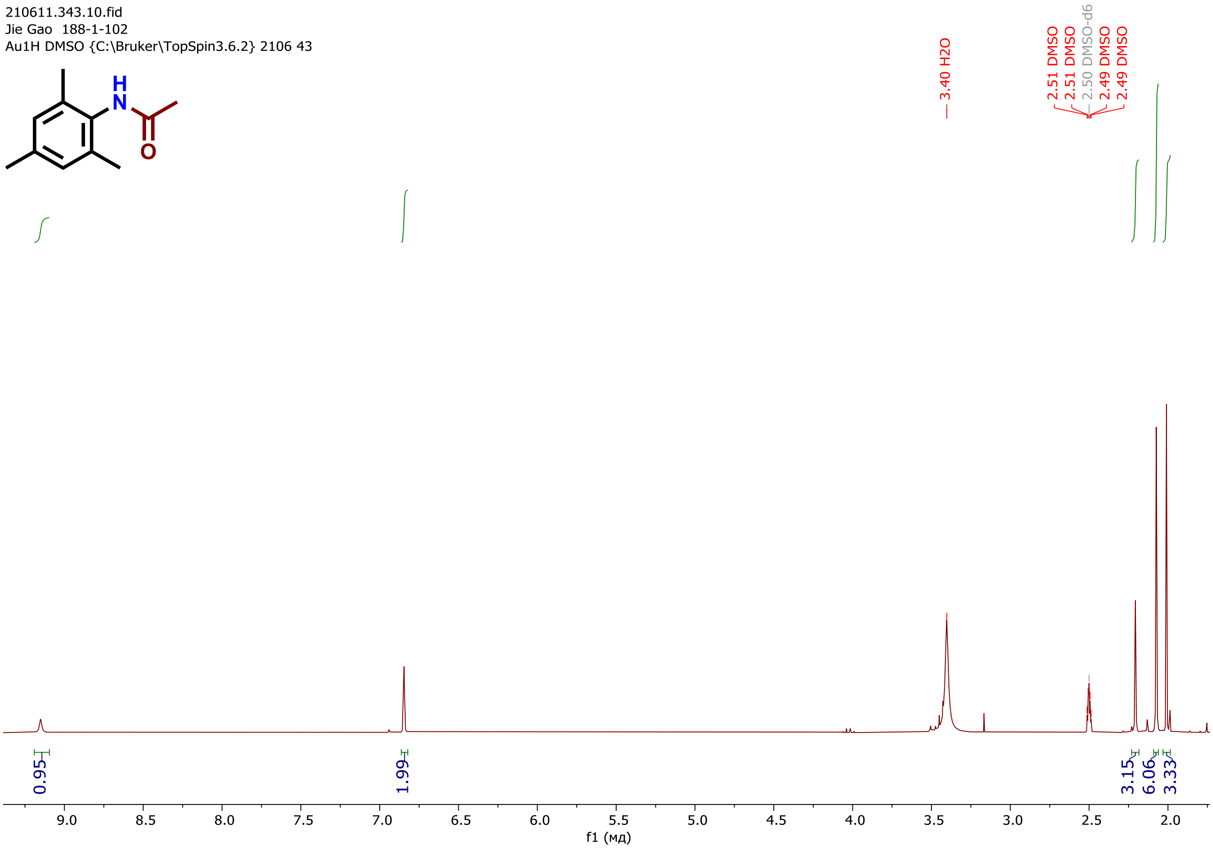

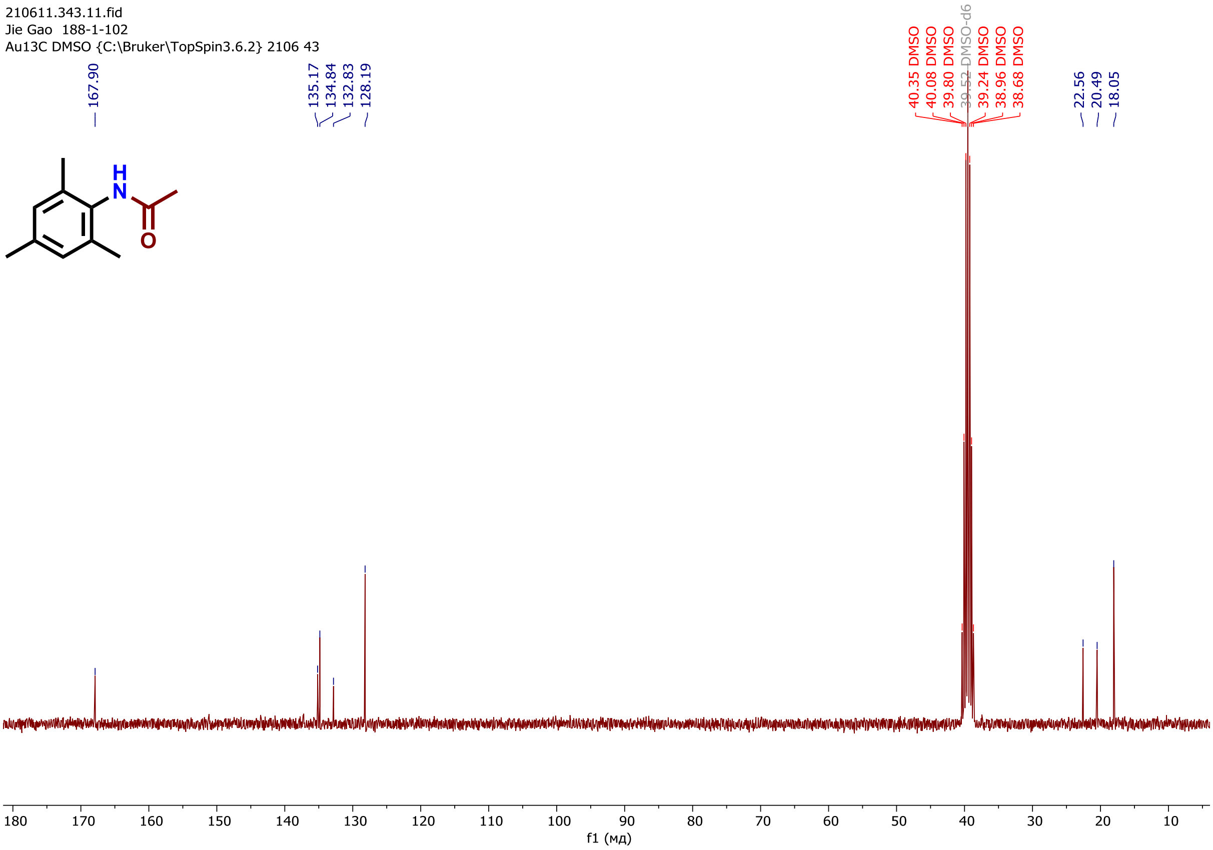

Supplementary Figure 33. NMR spectra of N-mesitylacetamide.

N-(4-fluorophenyl)acetamide (12)

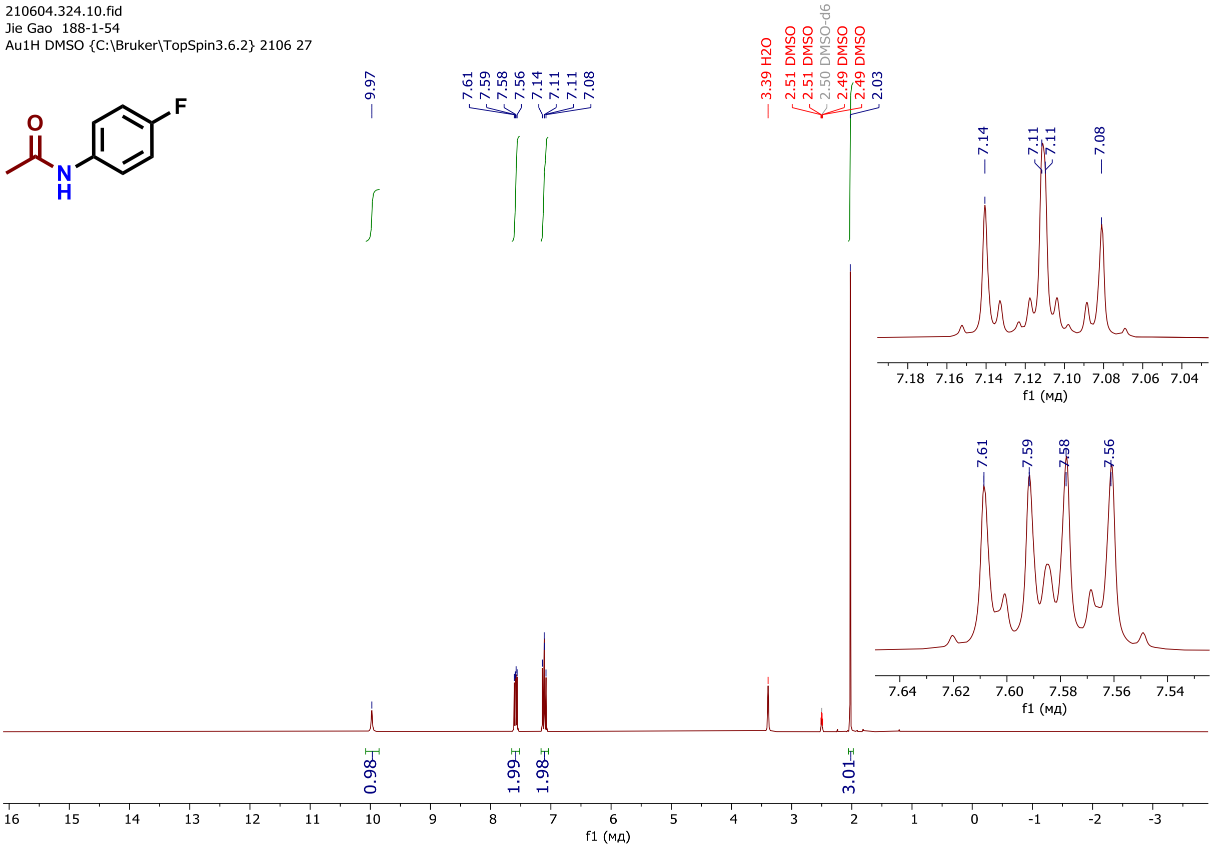

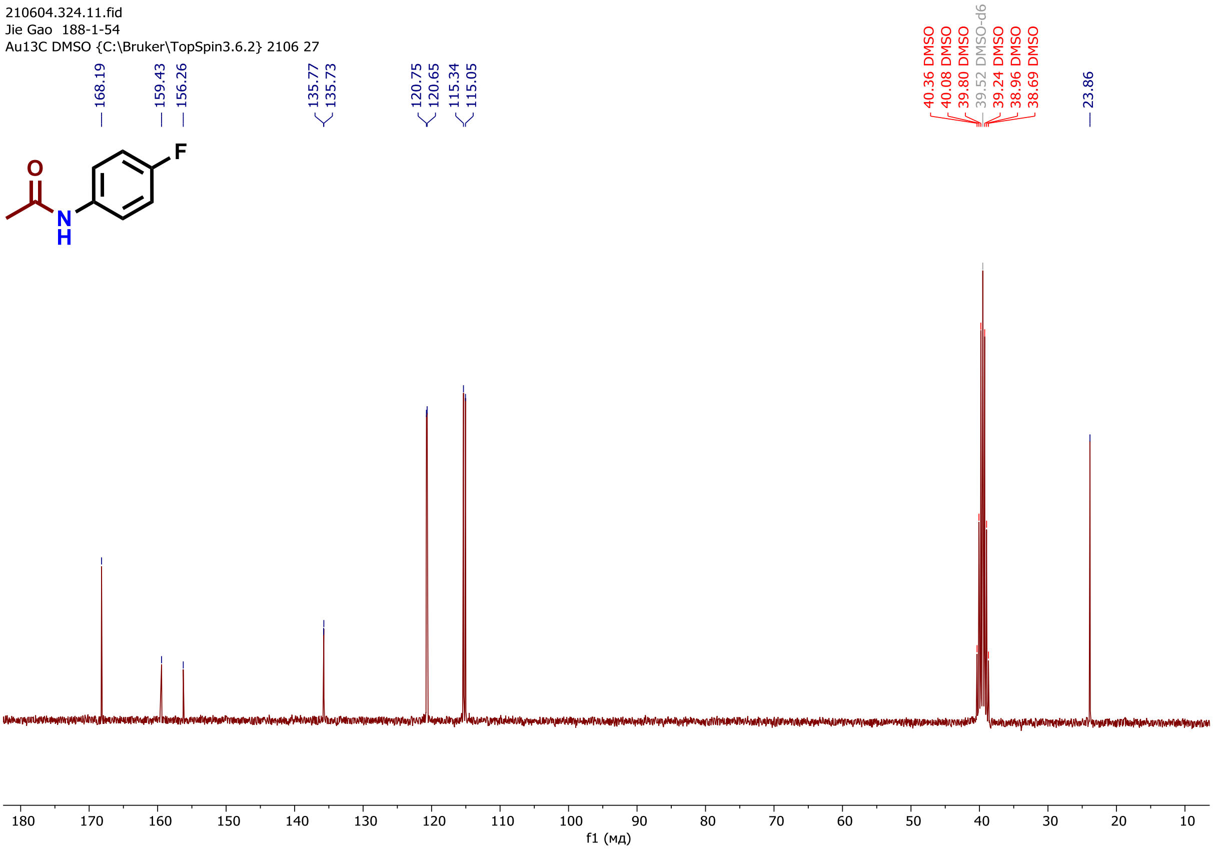

Supplementary Figure 34. NMR spectra of N-(4-fluorophenyl)acetamide.

N-(2-fluorophenyl)acetamide (13)

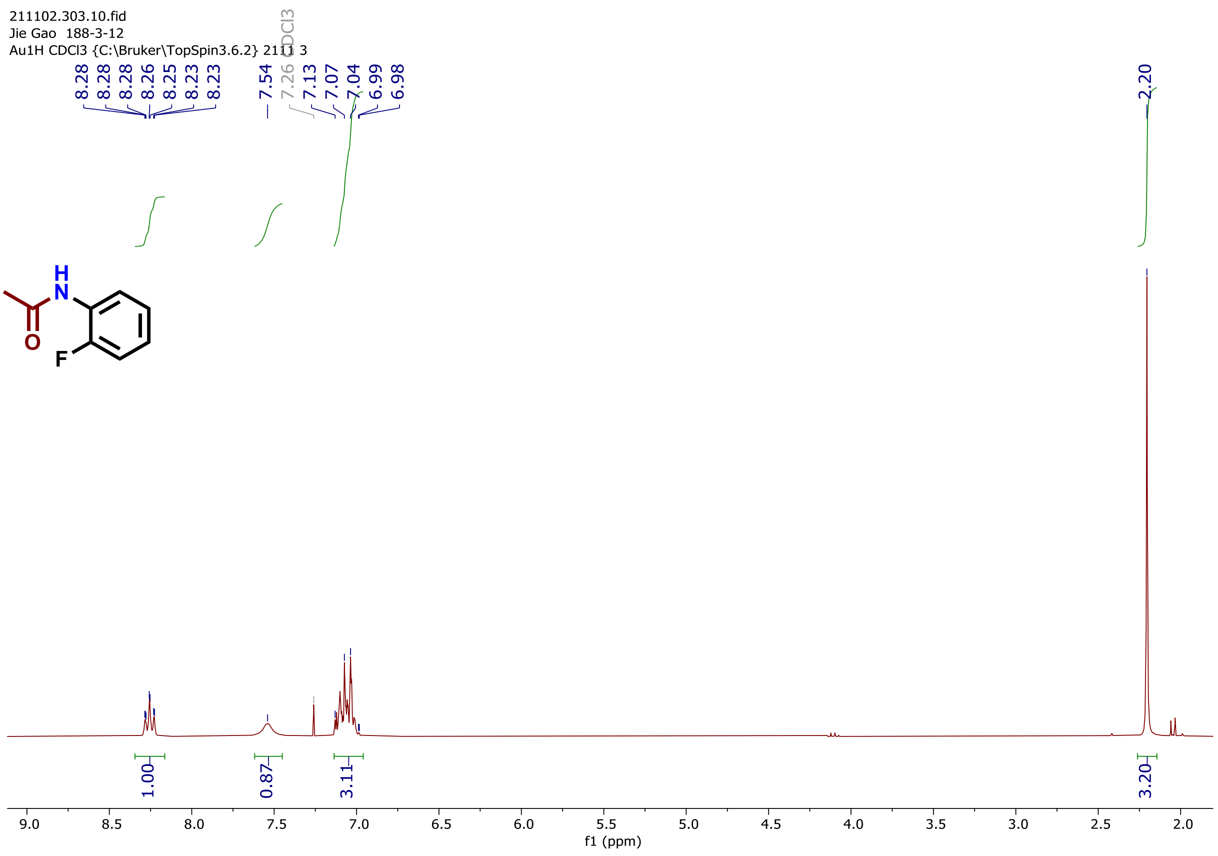

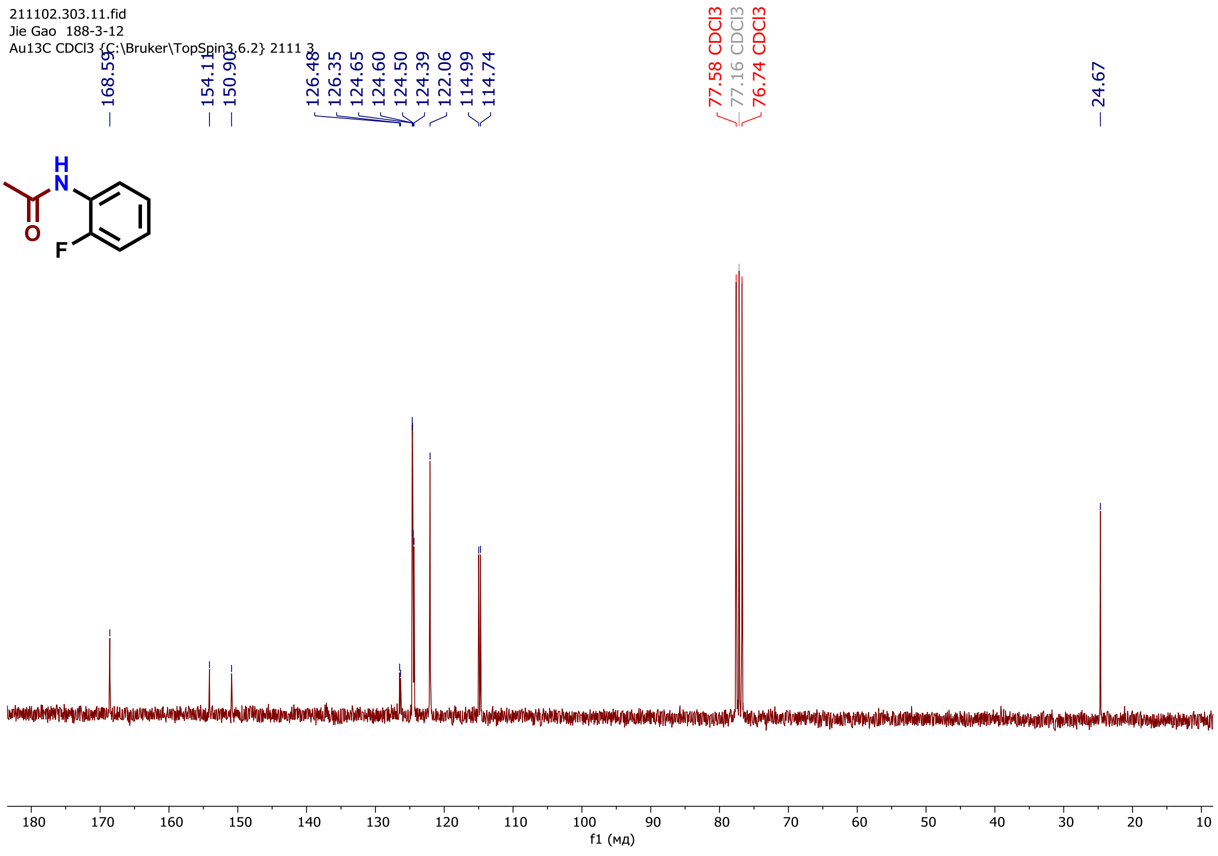

Supplementary Figure 35. NMR spectra of N-(2-fluorophenyl)acetamide.

N-(3-hydroxy-5-(trifluoromethyl)phenyl)acetamide (14)

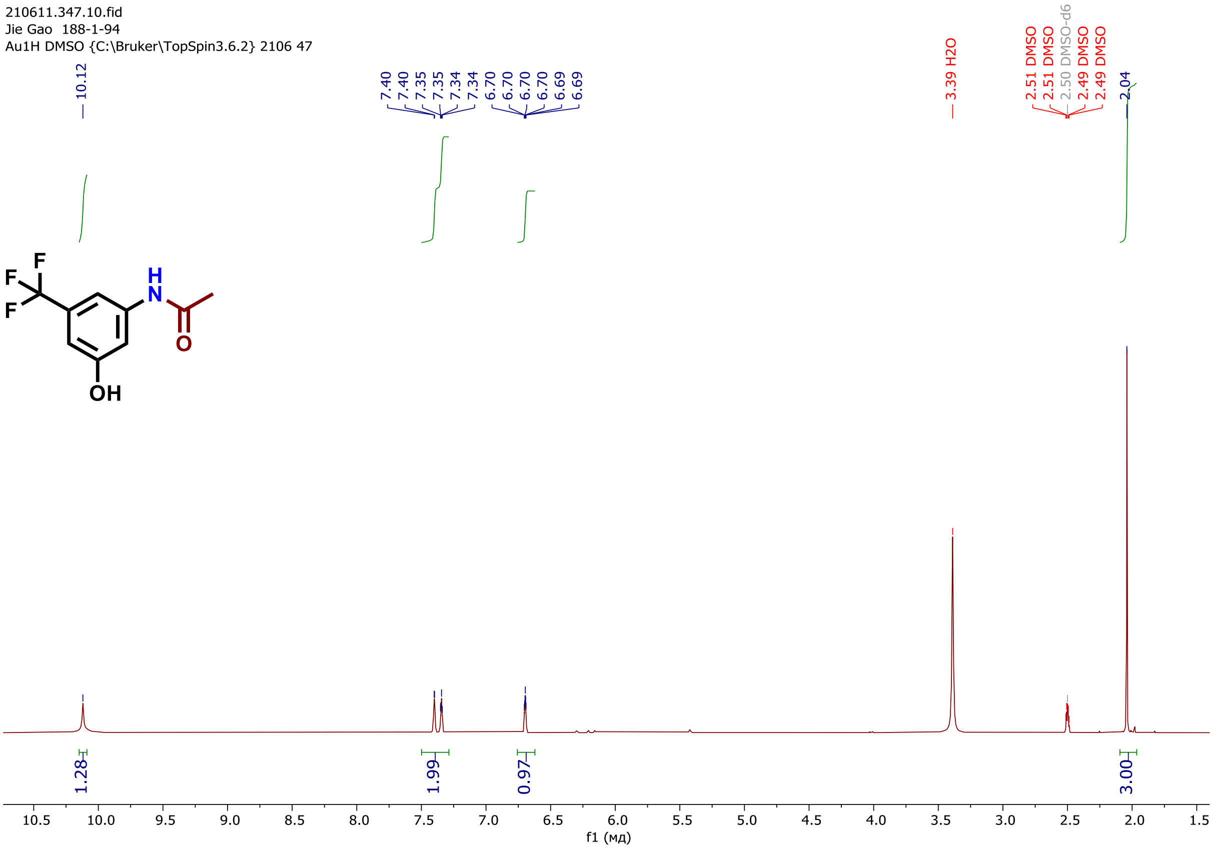

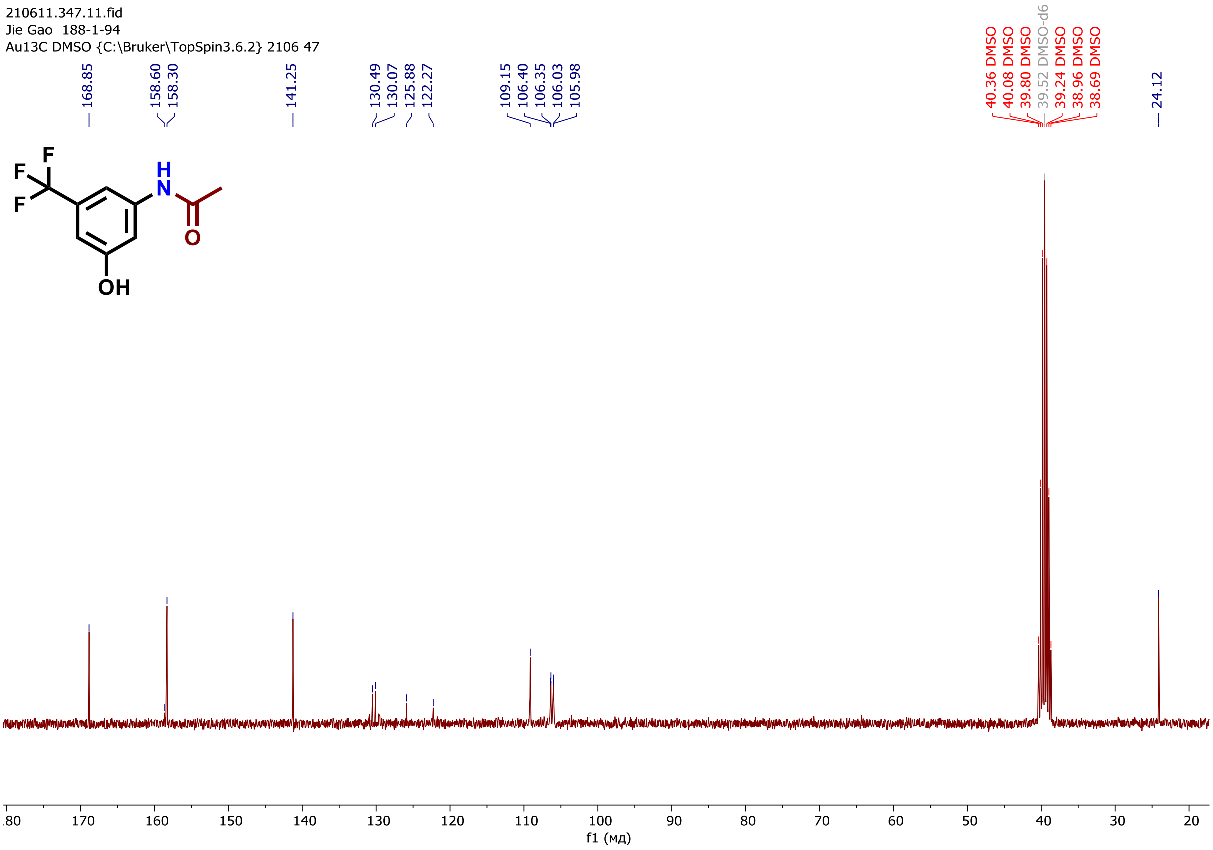

Supplementary Figure 36. NMR spectra of N-(3-hydroxy-5-(trifluoromethyl)phenyl)acetamide.

N-(3-chloro-4-fluorophenyl)acetamide (15)

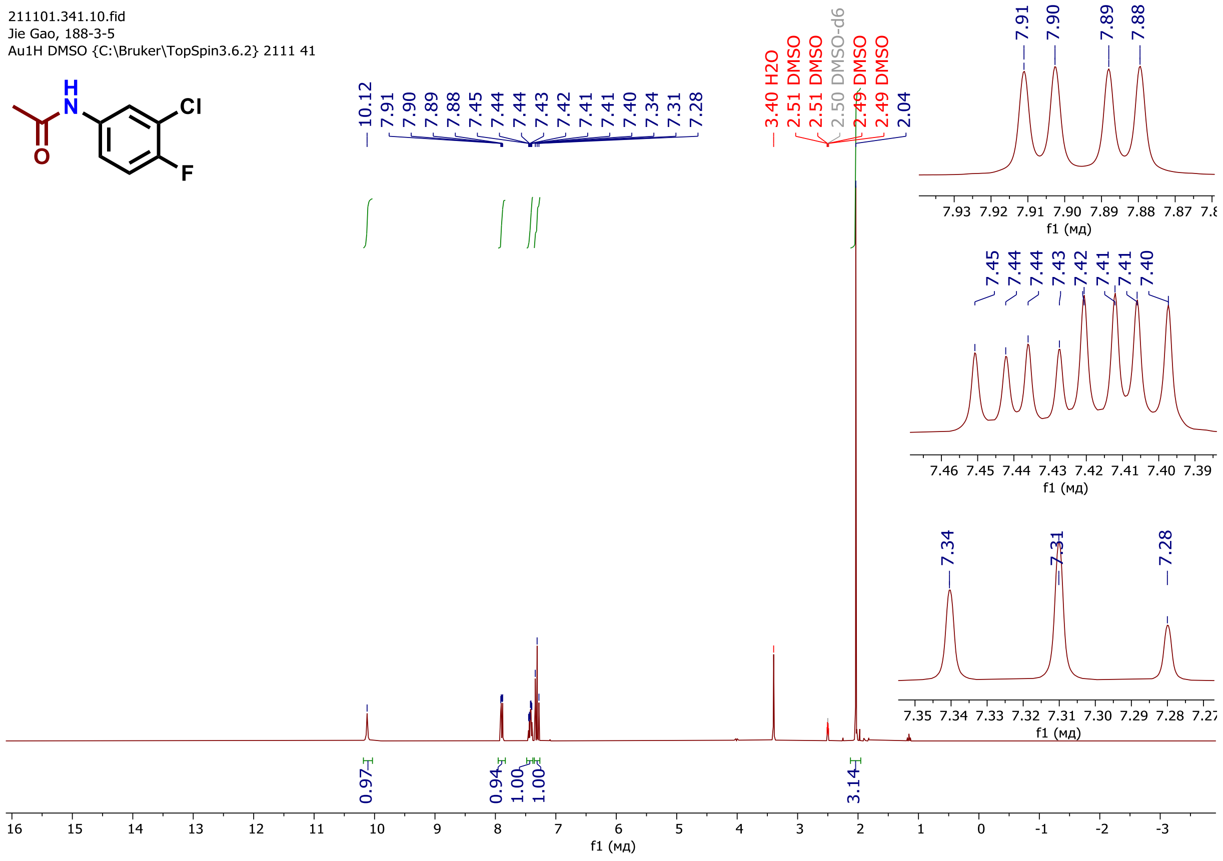

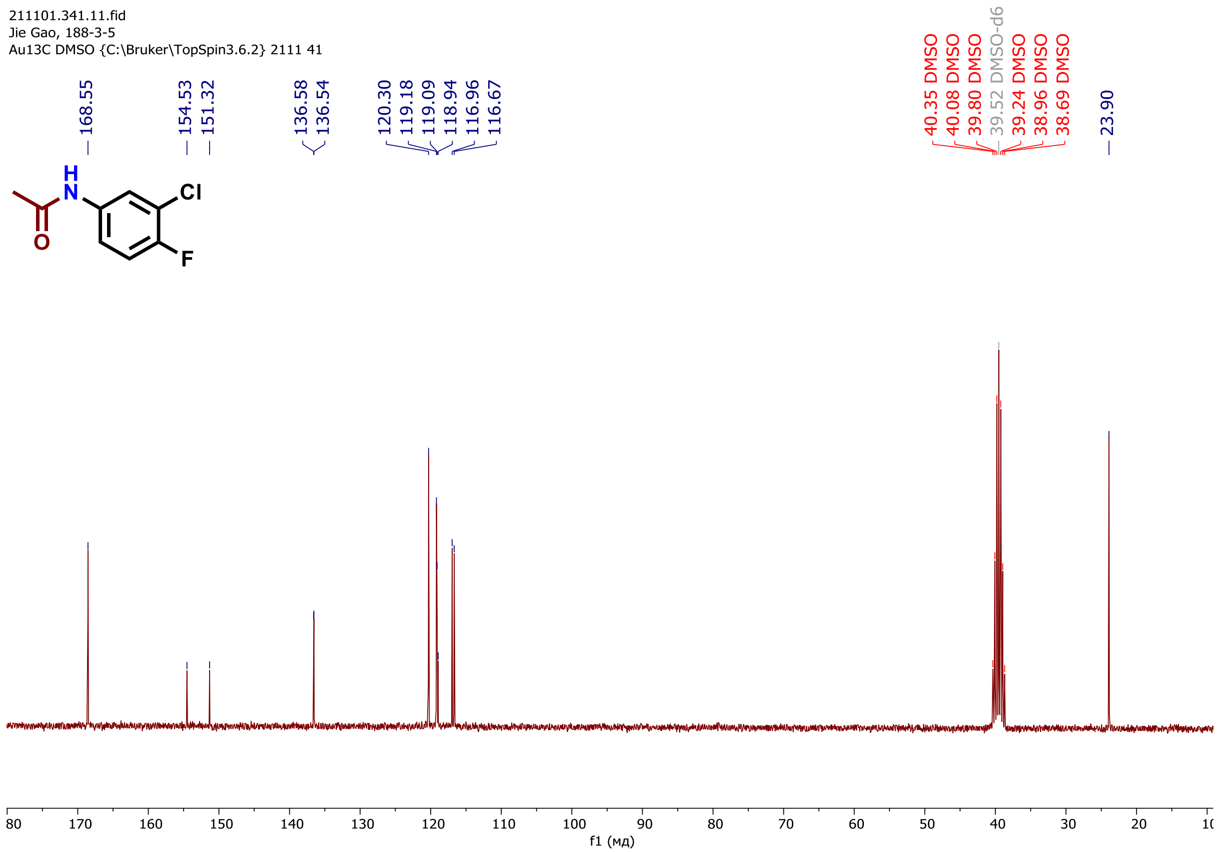

Supplementary Figure 37. NMR spectra of N-(3-chloro-4-fluorophenyl)acetamide.

N-(4-chlorophenyl)acetamide (16)

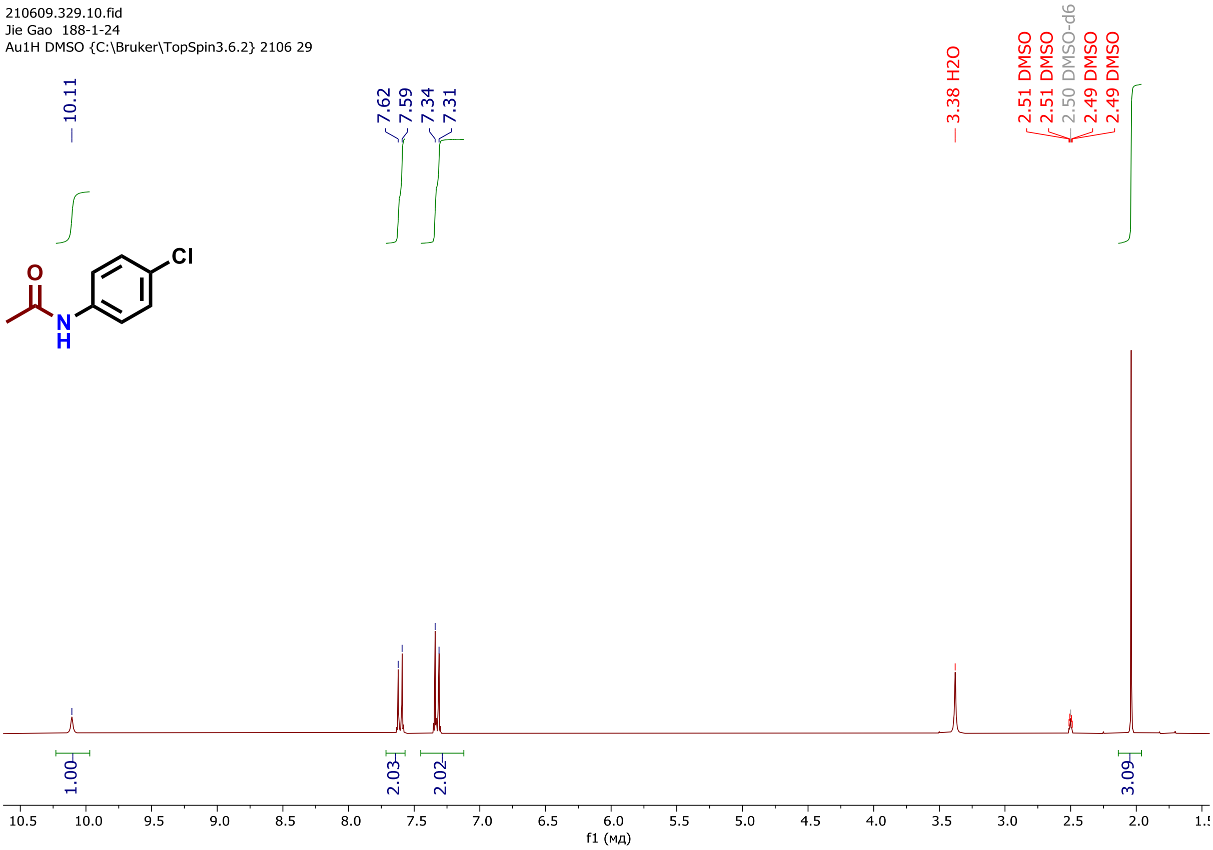

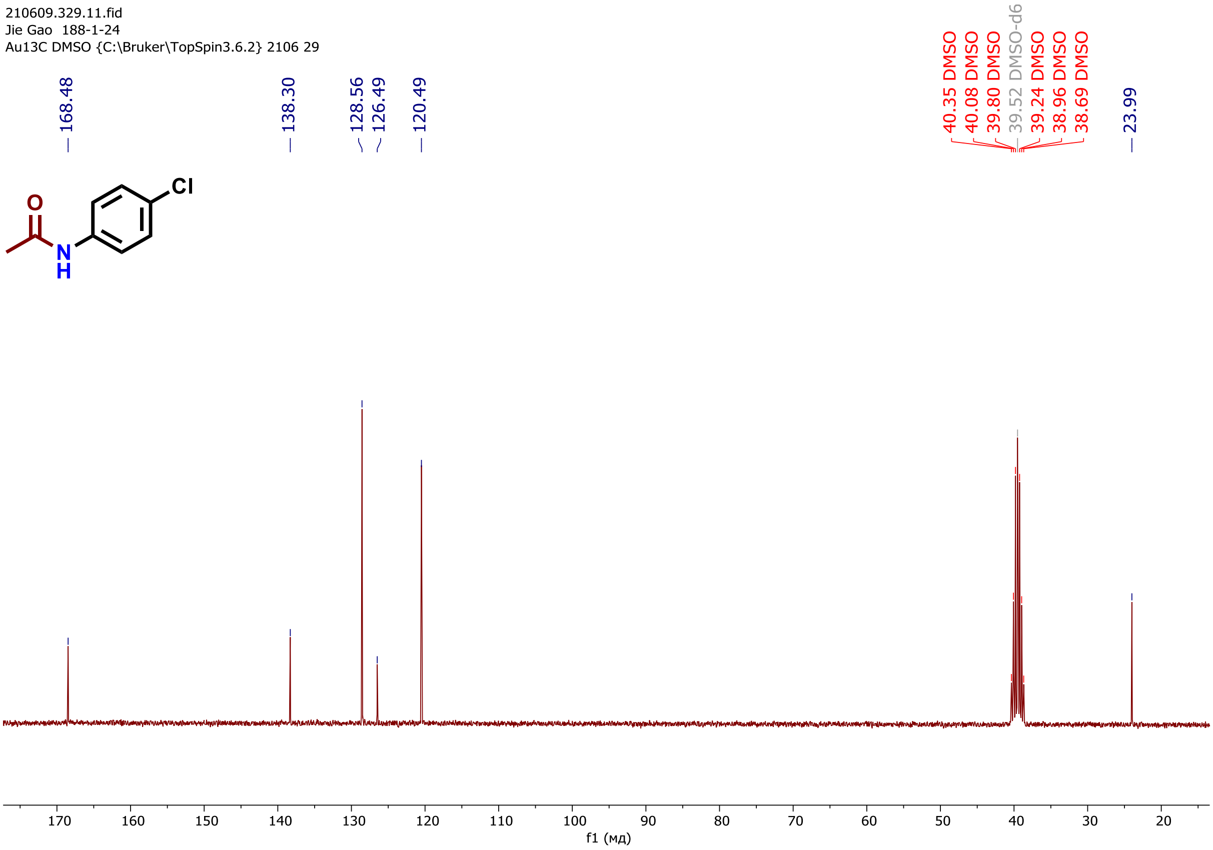

Supplementary Figure 38. NMR spectra of N-(4-chlorophenyl)acetamide.

N-(2,4-dichlorophenyl)acetamide (17)

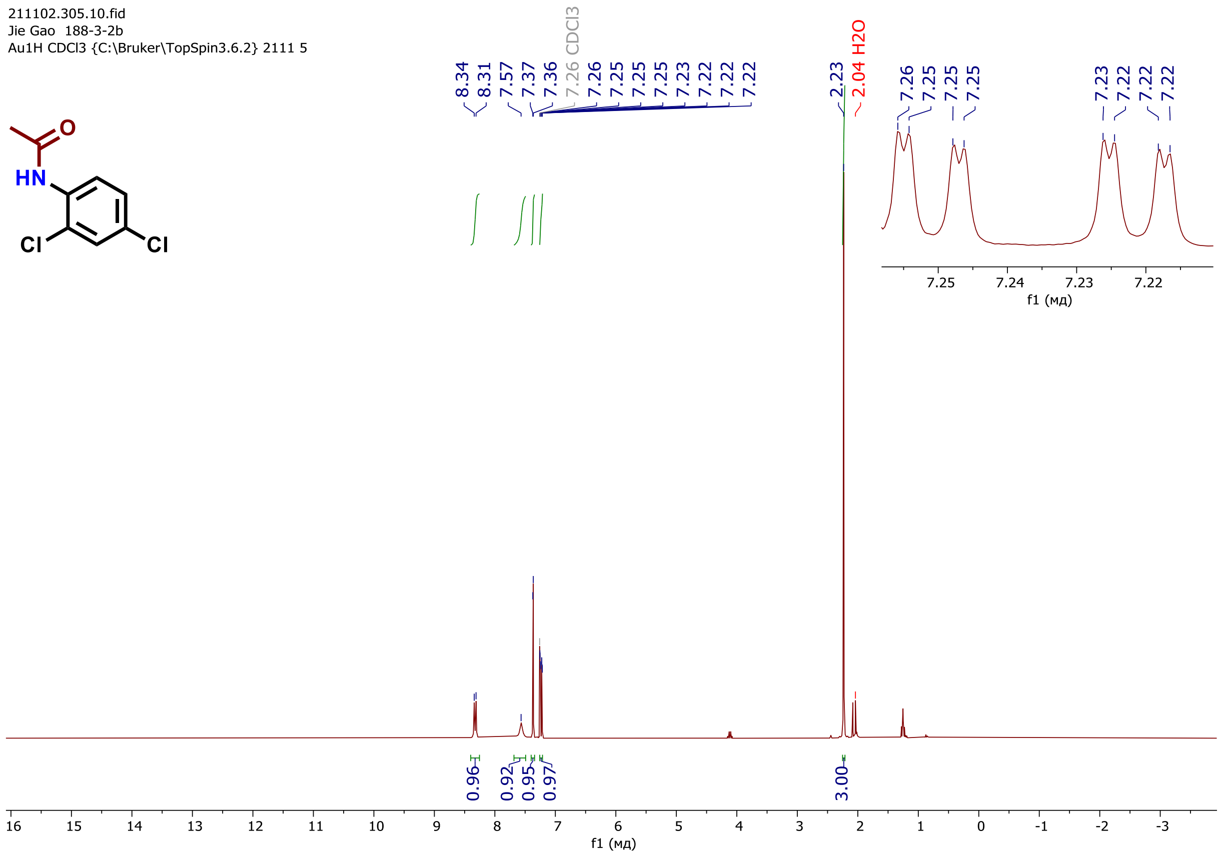

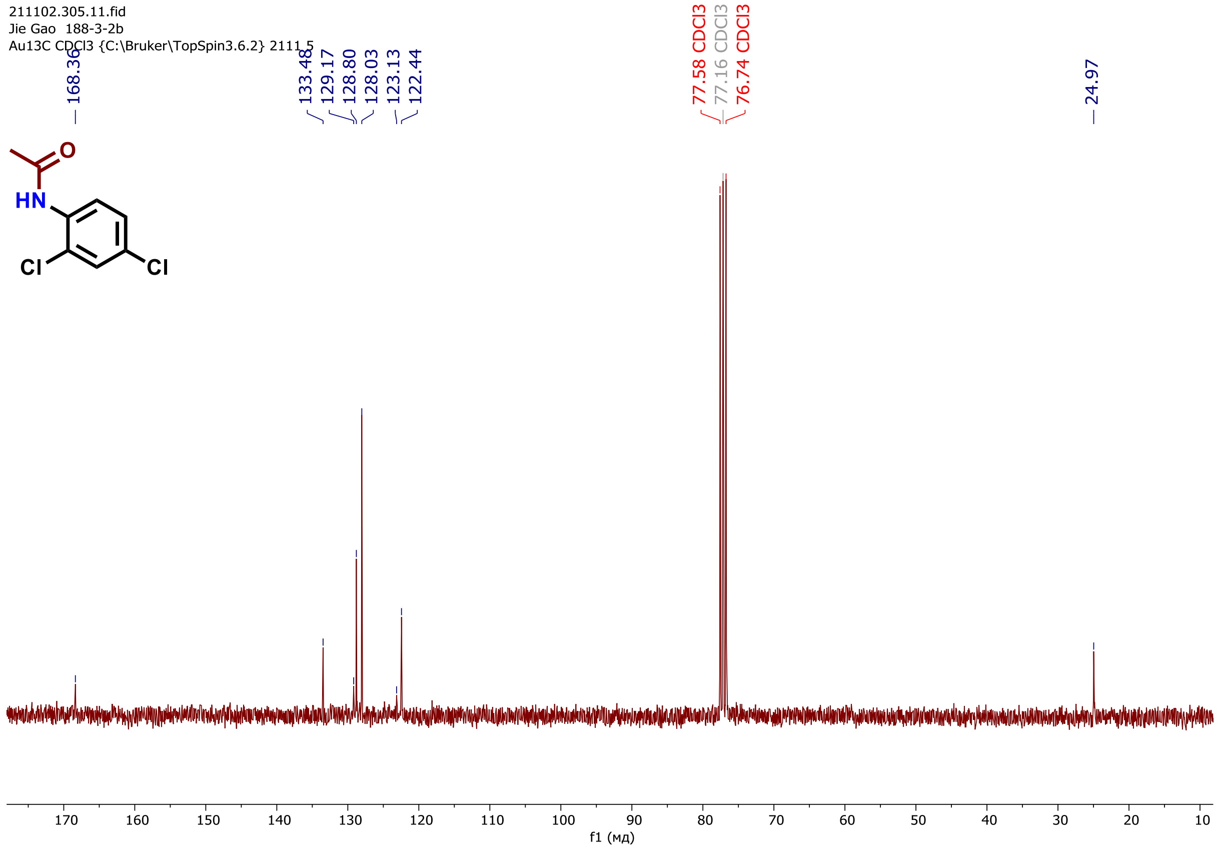

Supplementary Figure 39. NMR spectra of N-(2,4-dichlorophenyl)acetamide.

N-(2,5-dimethoxyphenyl)acetamide (18)

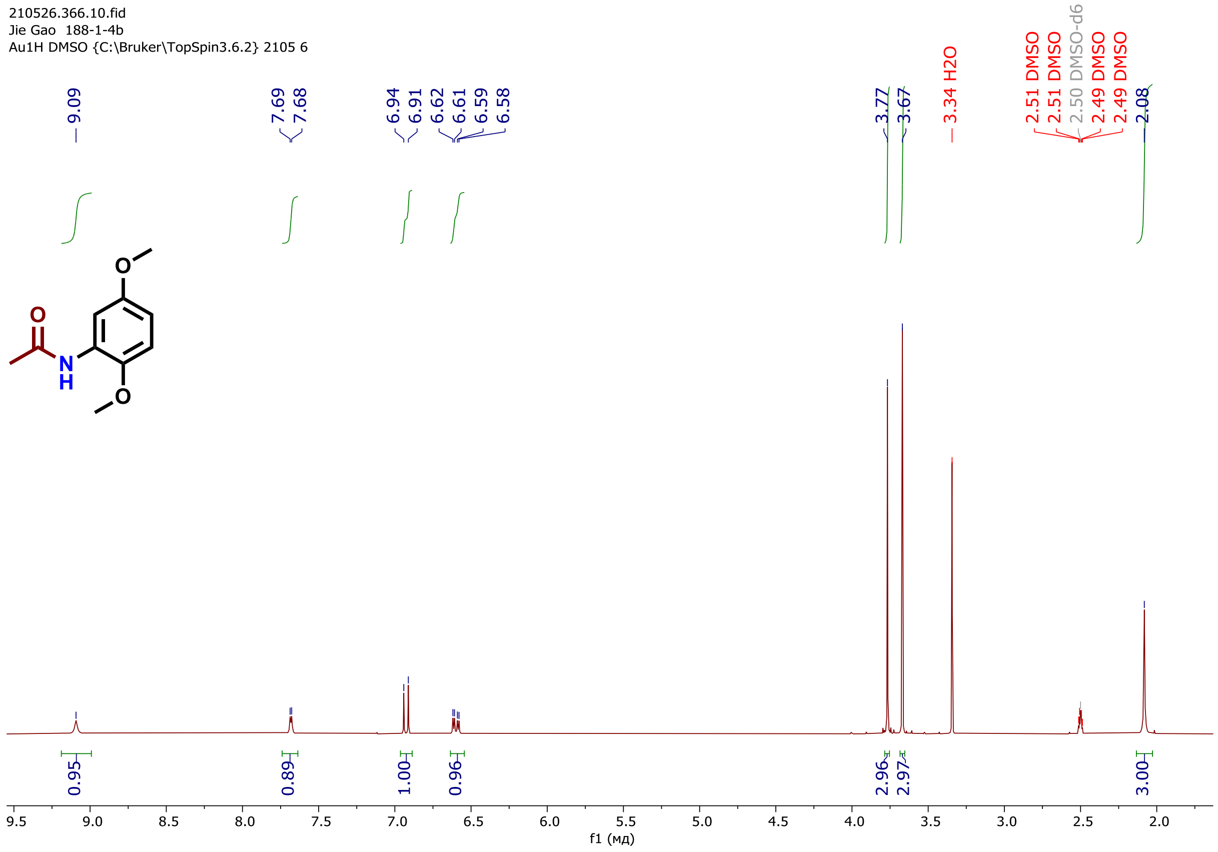

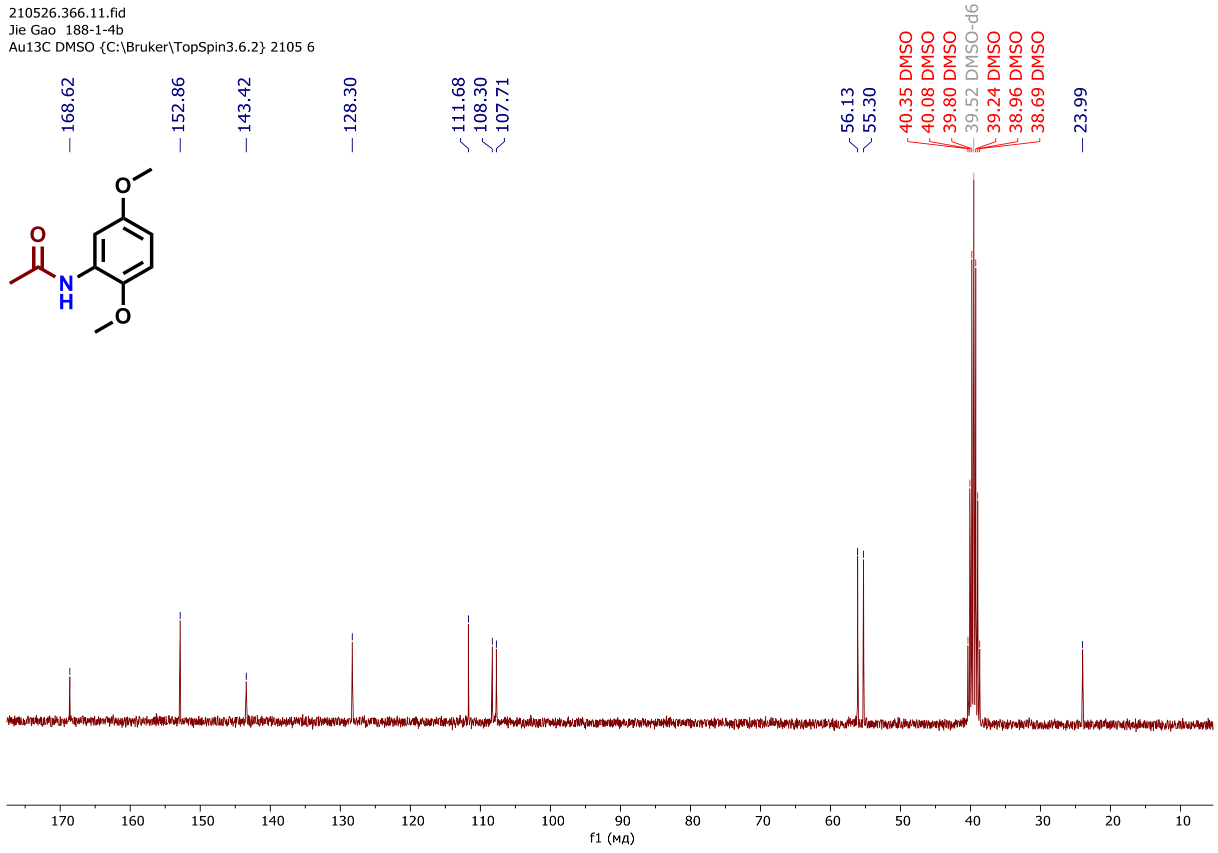

Supplementary Figure 40. NMR spectra of N-(2,5-dimethoxyphenyl)acetamide.

N-propylacetamide (19)

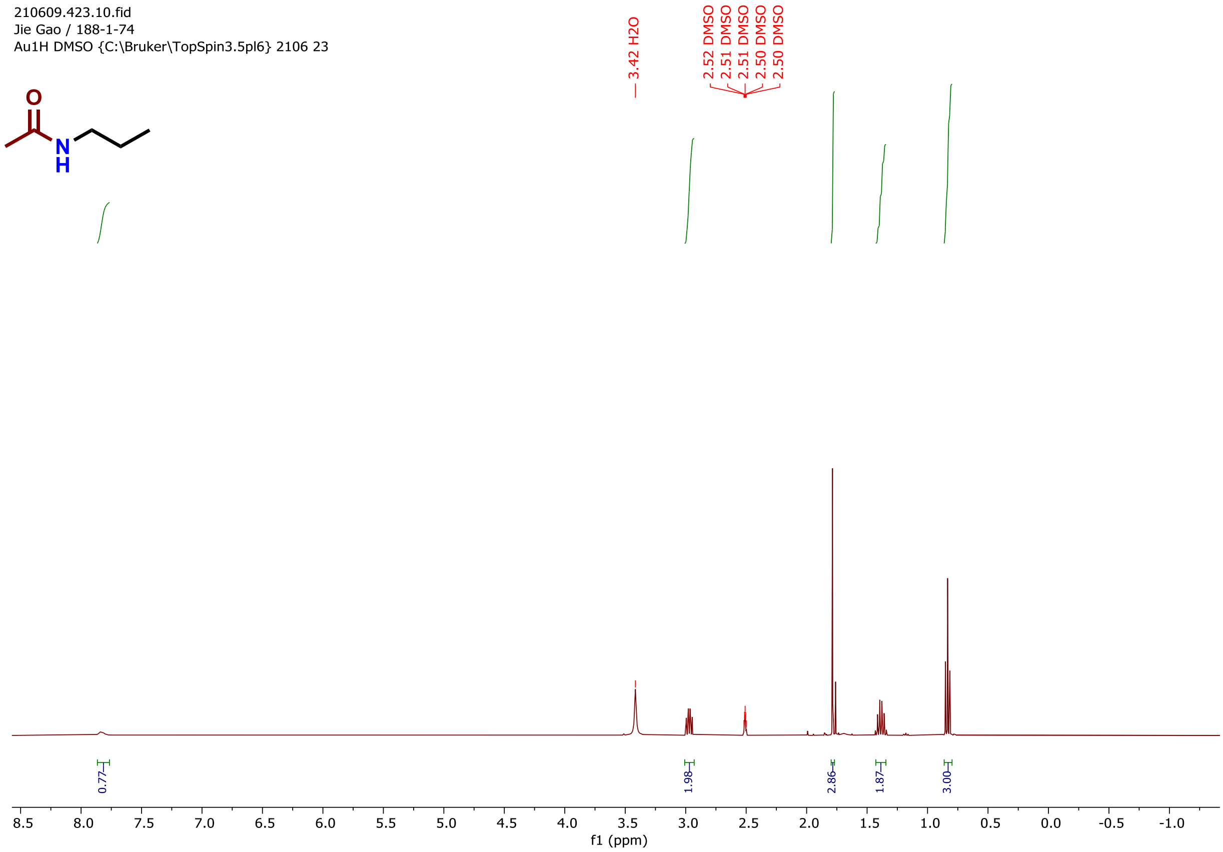

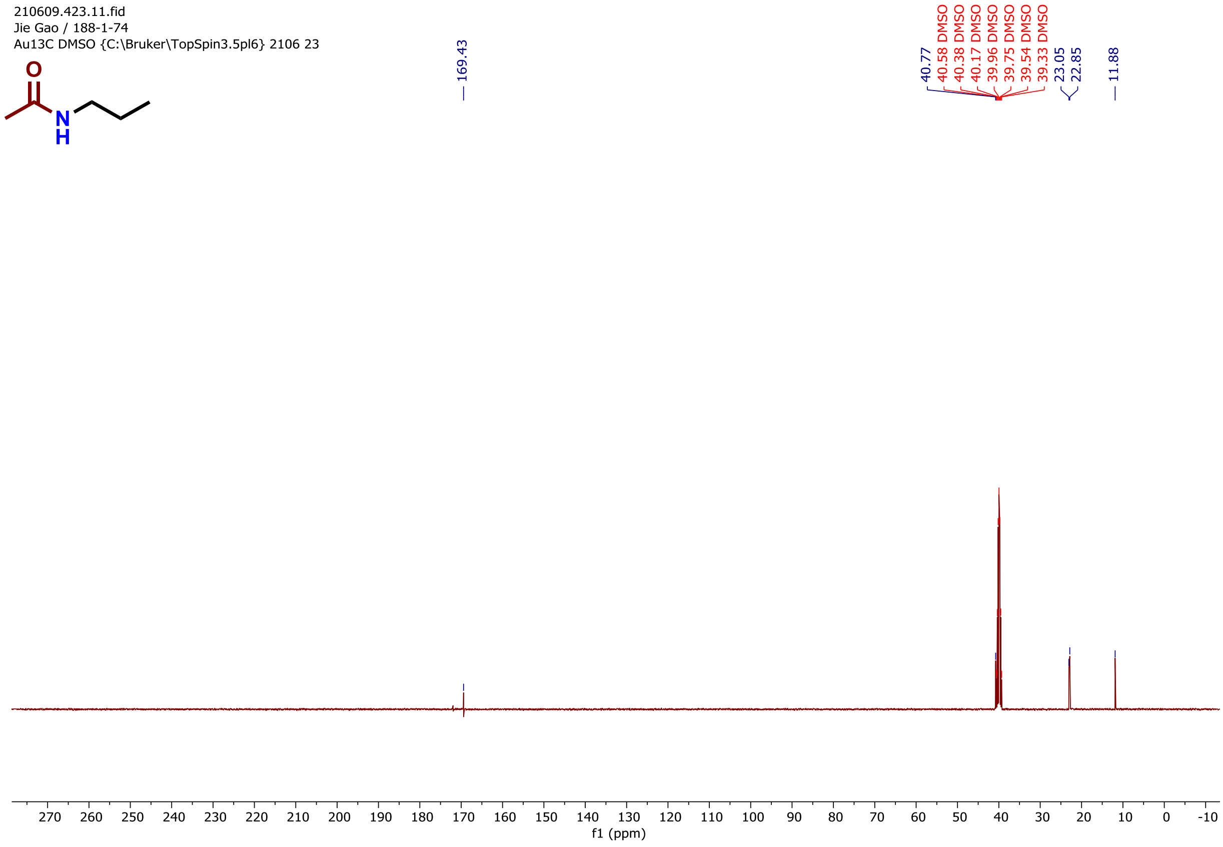

Supplementary Figure 41. NMR spectra of N-propylacetamide.

N-pentylacetamide (20)

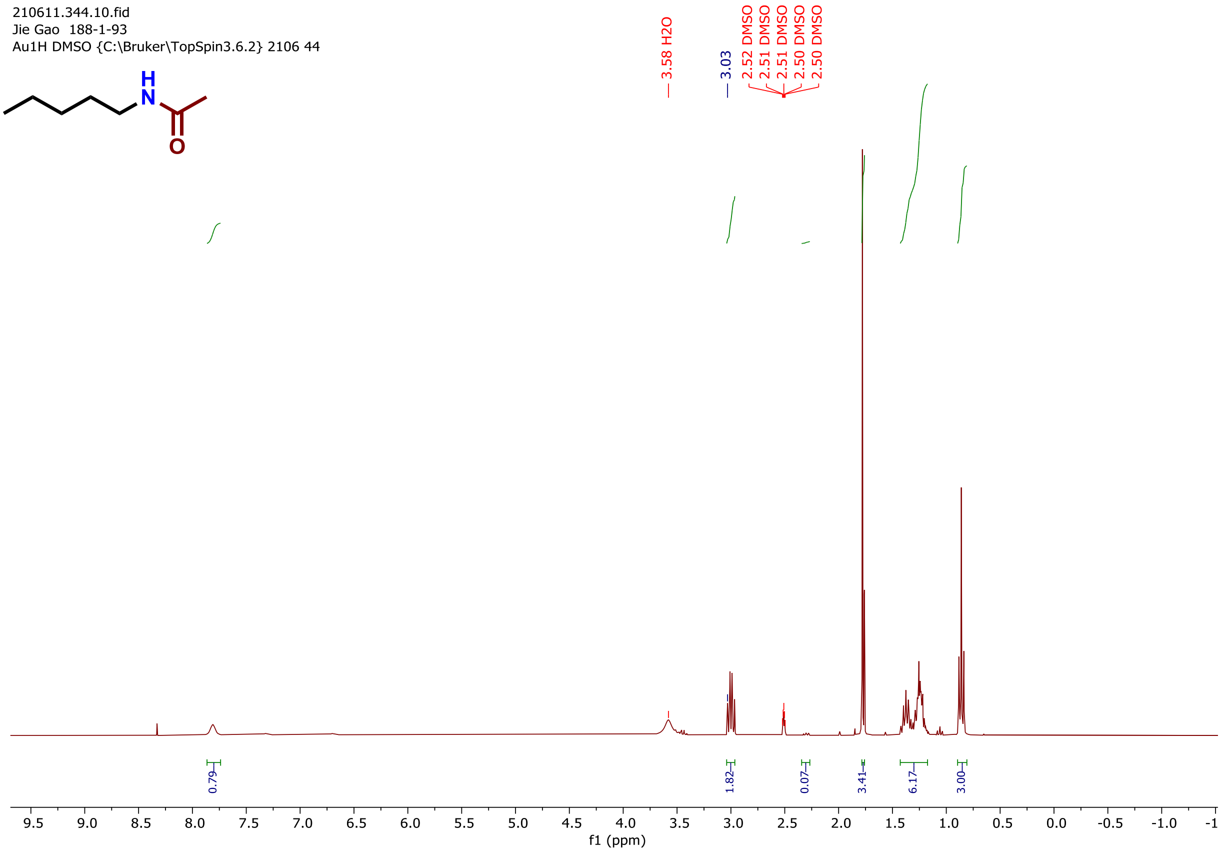

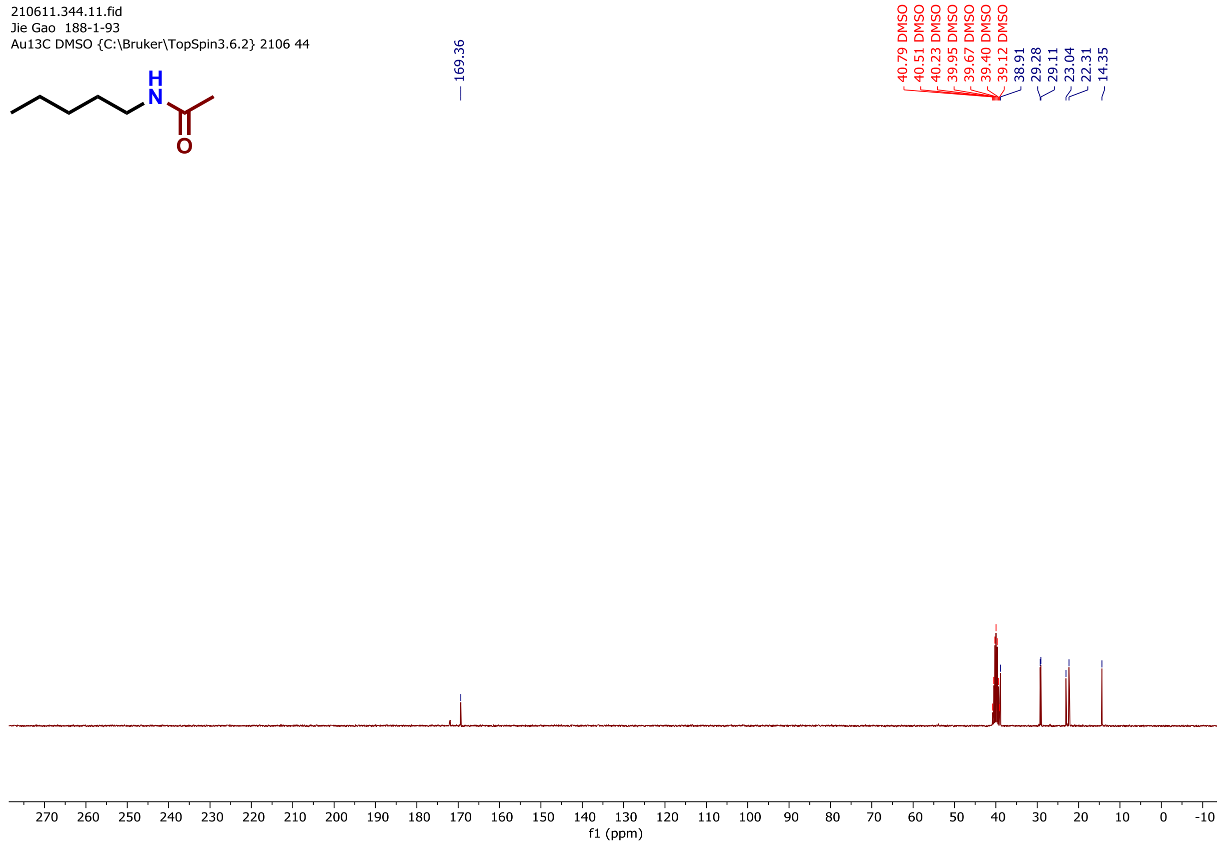

Supplementary Figure 42. NMR spectra of N-pentylacetamide.

N-heptylacetamide (21)

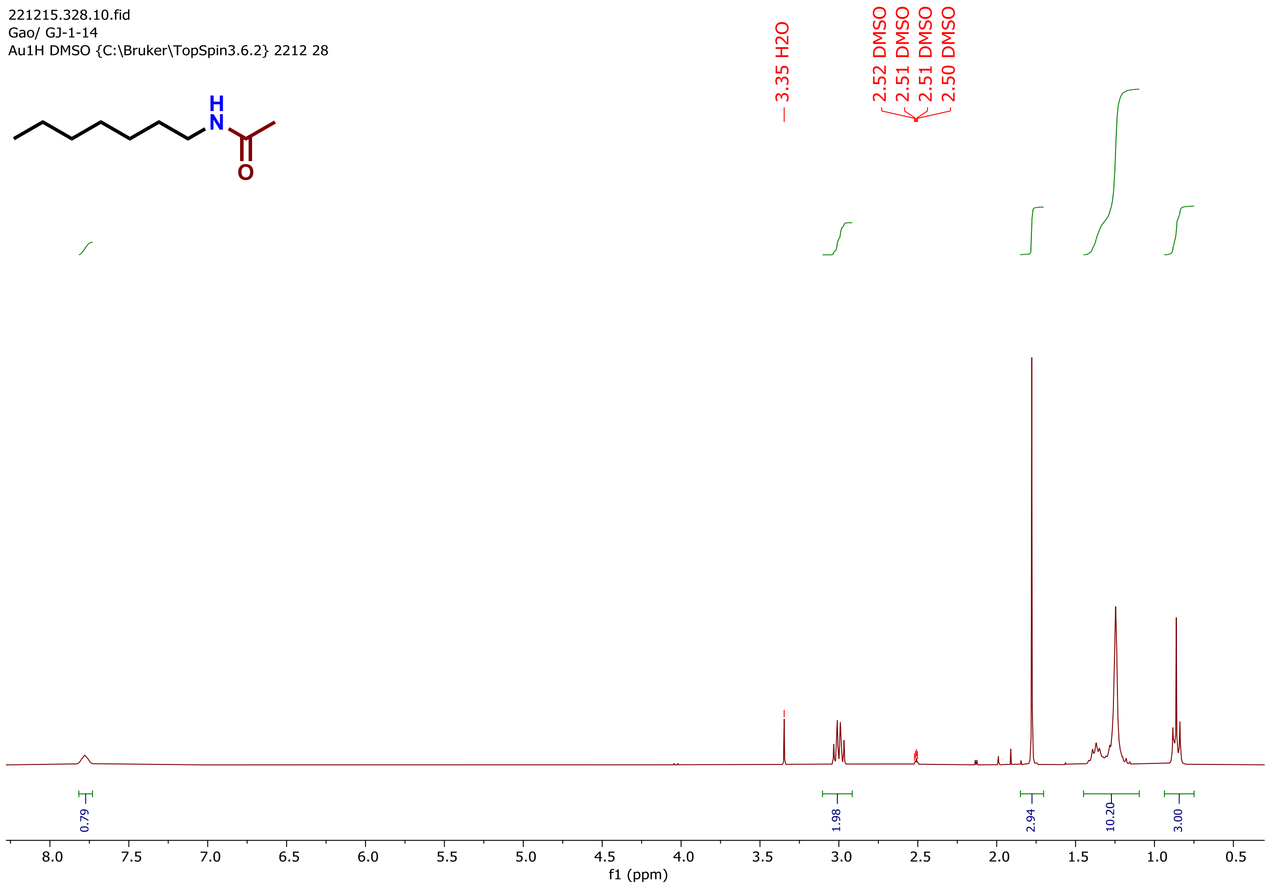

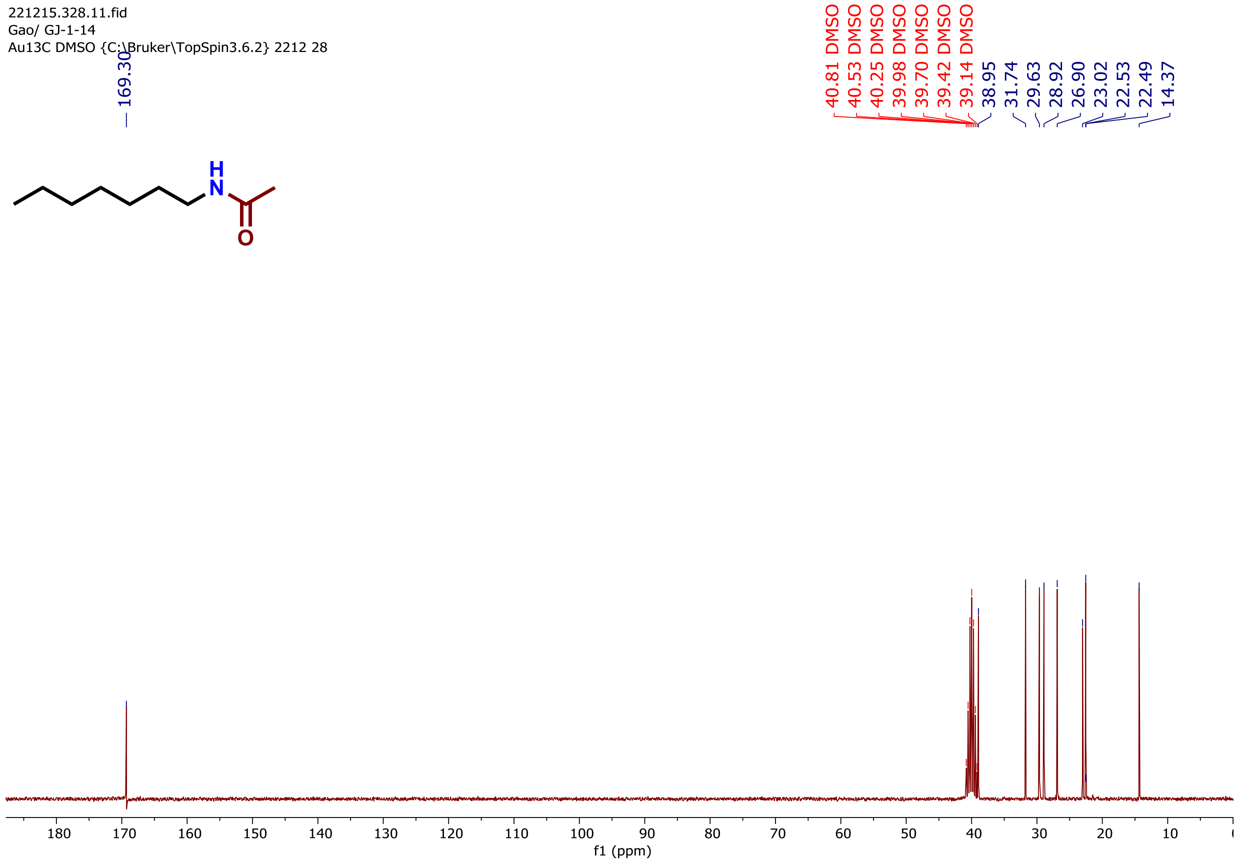

Supplementary Figure 43. NMR spectra of N-heptylacetamide.

N-octadecylacetamide (22)

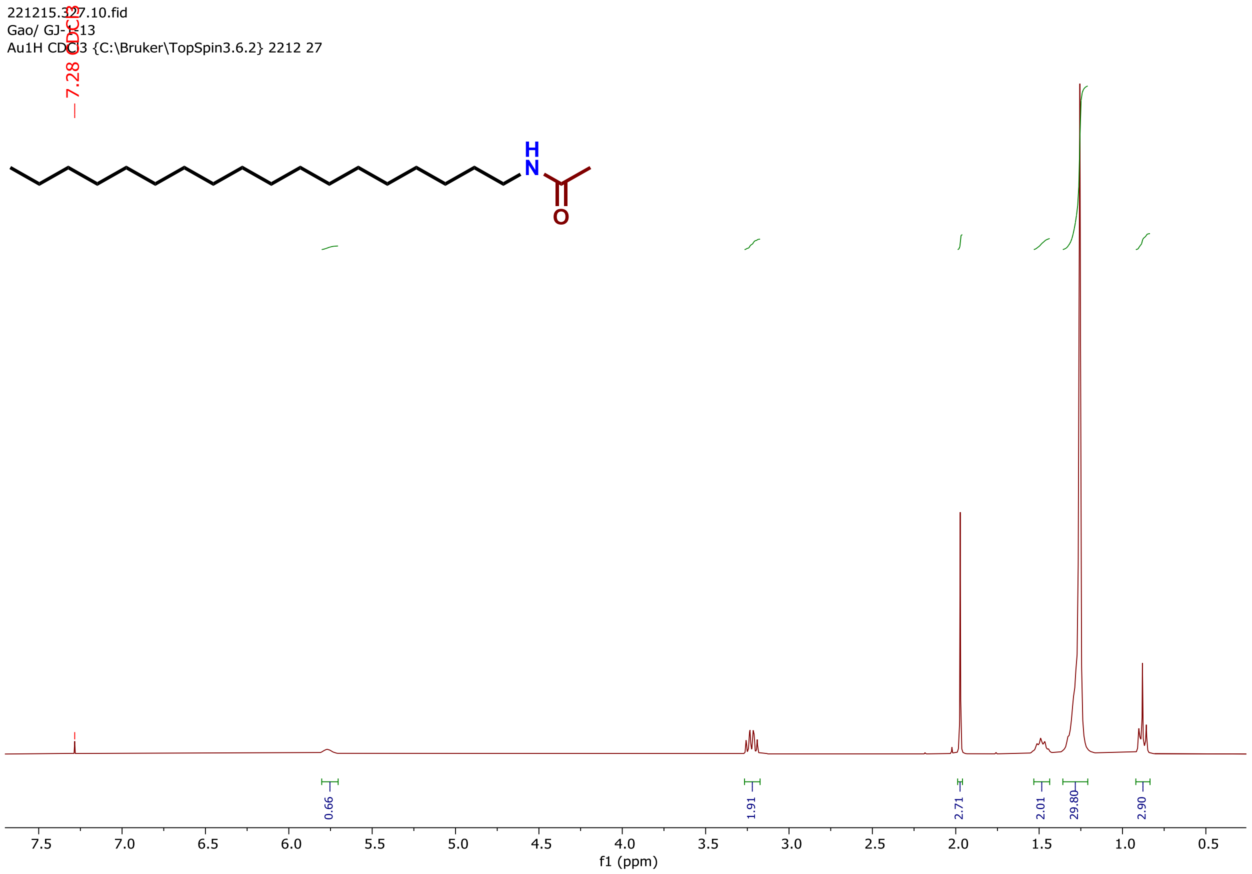

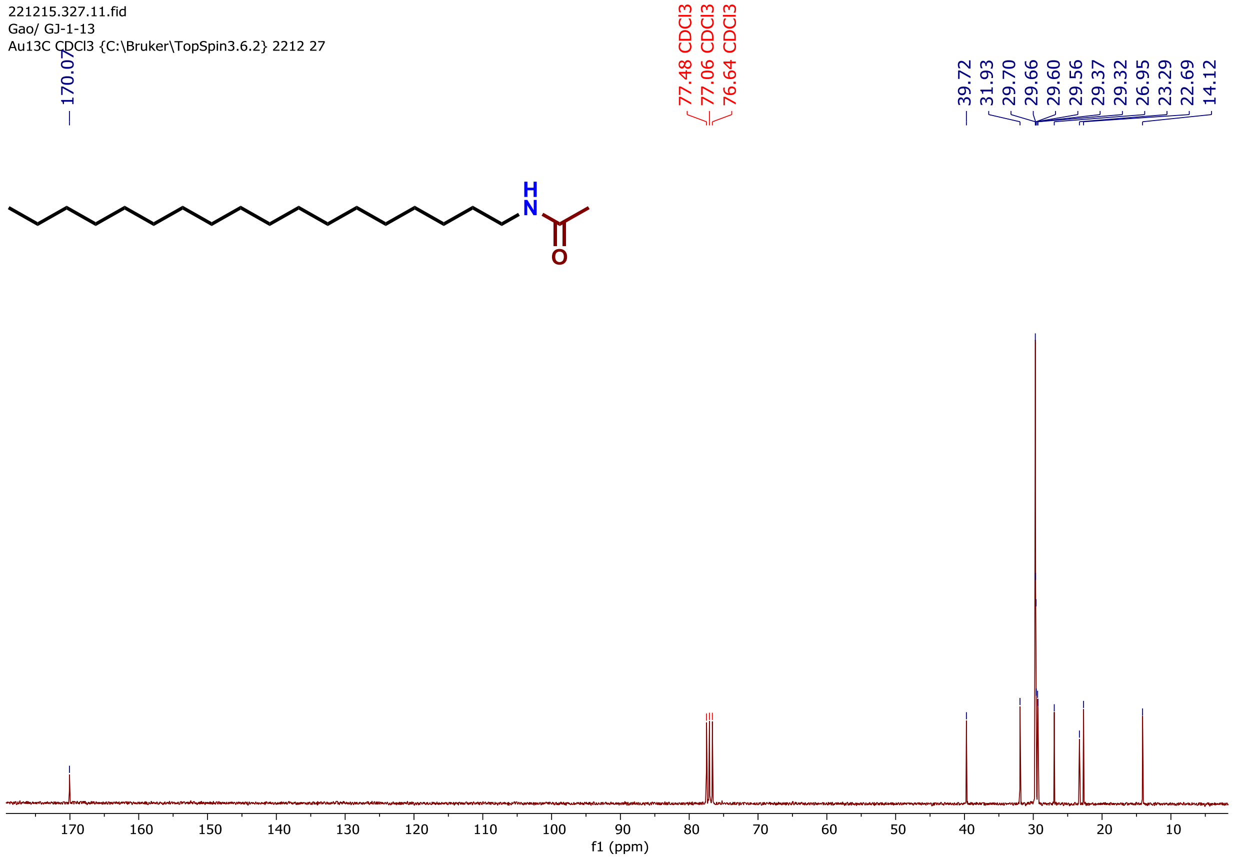

Supplementary Figure 44. NMR spectra of N-octadecylacetamide.

N-(4-acetylphenyl)acetamide (23)

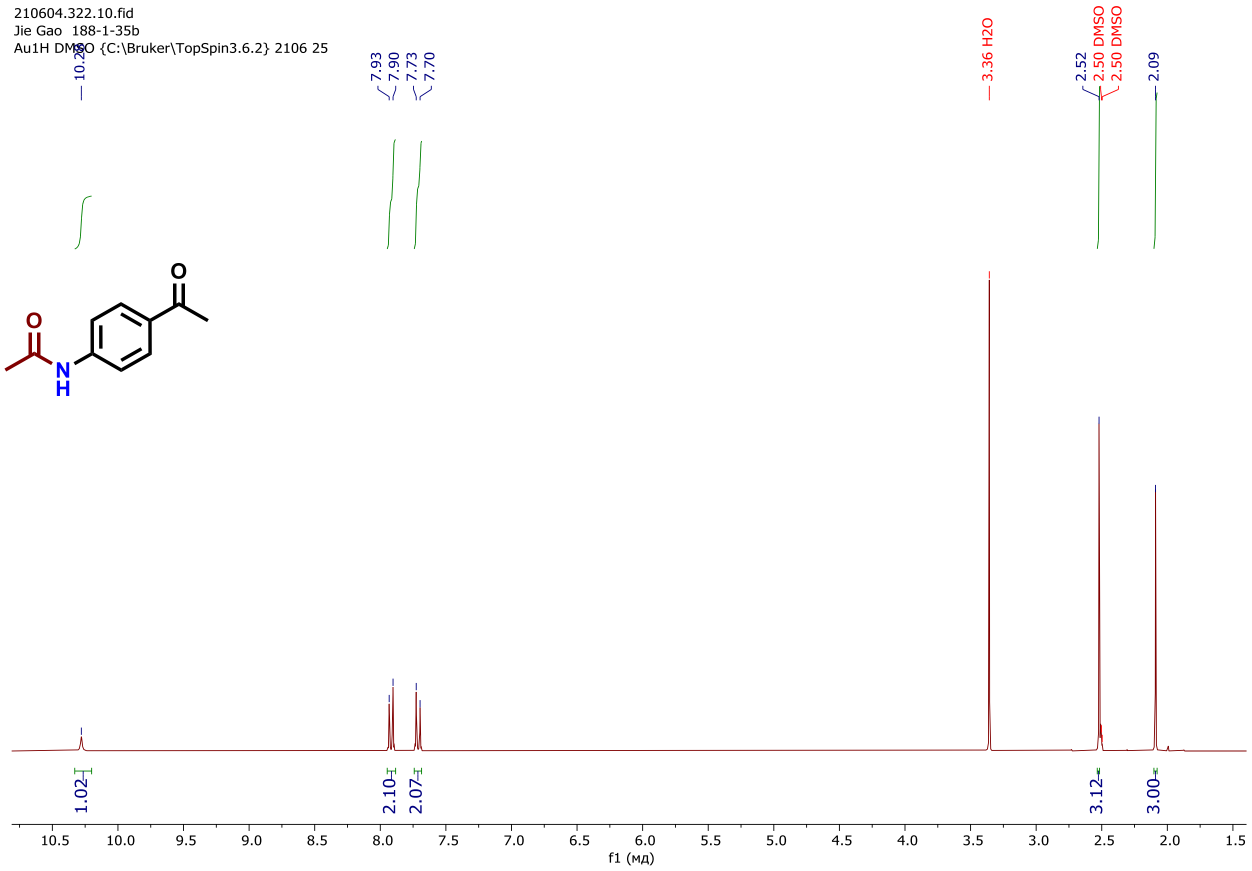

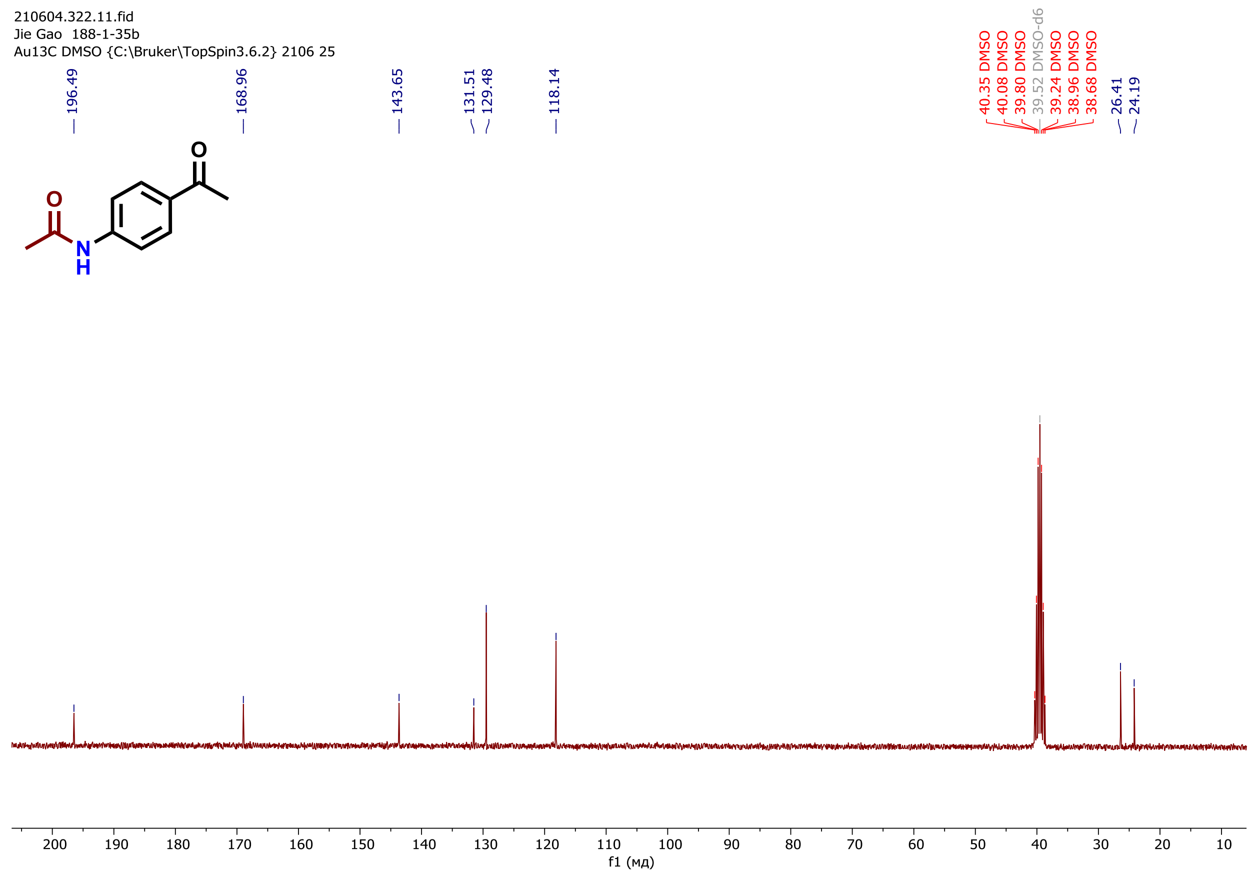

Supplementary Figure 45. NMR spectra of N-(4-acetylphenyl)acetamide.

N-(3-acetyl-5-ethyl-2-hydroxyphenyl)acetamide (24)

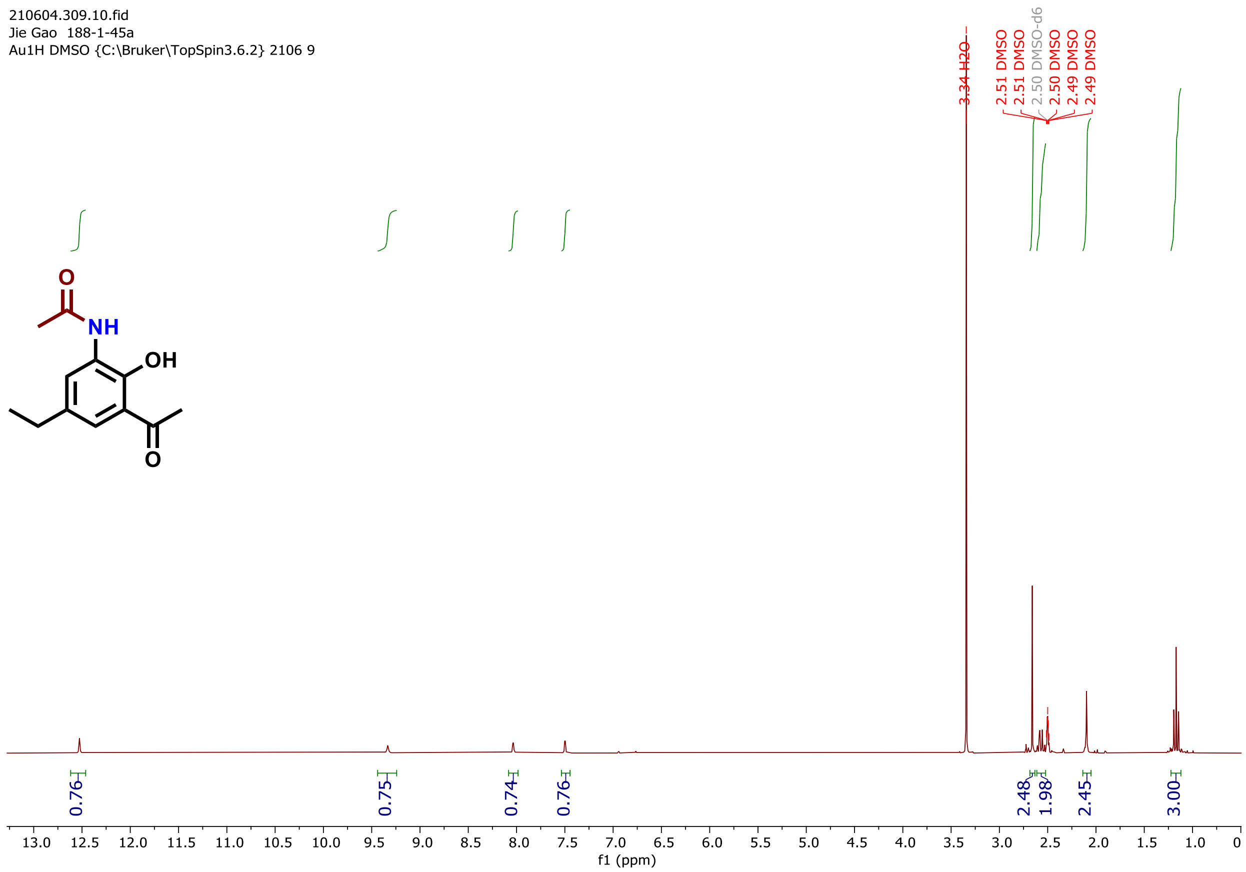

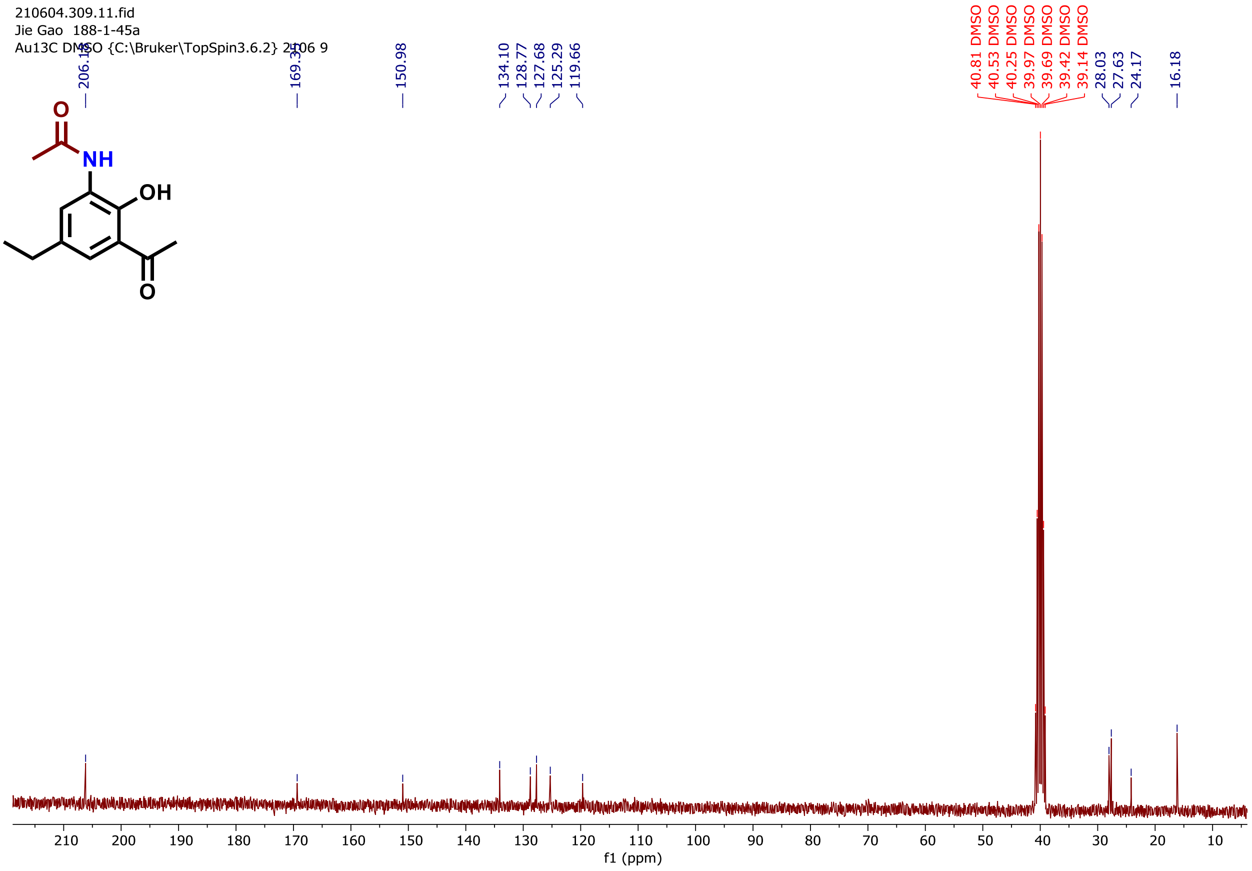

Supplementary Figure 46. NMR spectra of N-(3-acetyl-5-ethyl-2-hydroxyphenyl)acetamide.

5-acetamido-2-chlorobenzoic acid (25)

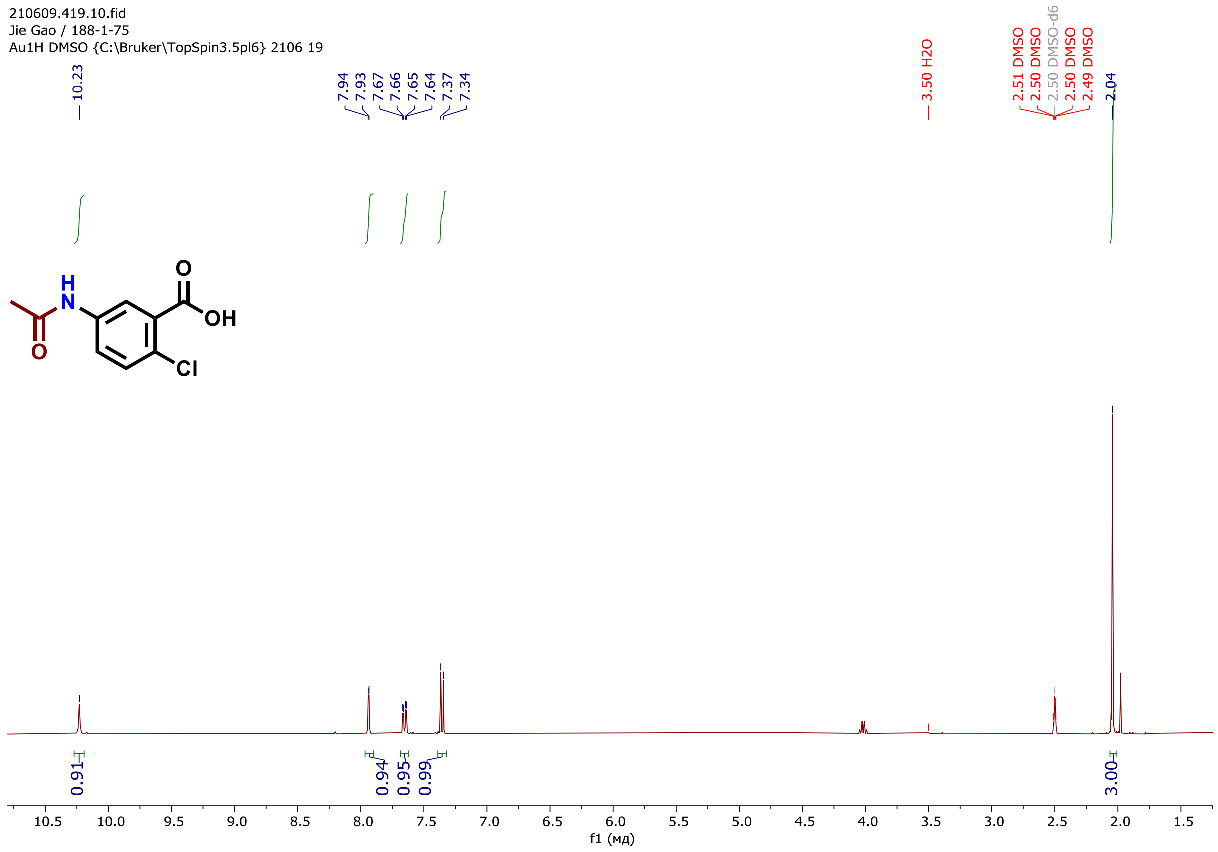

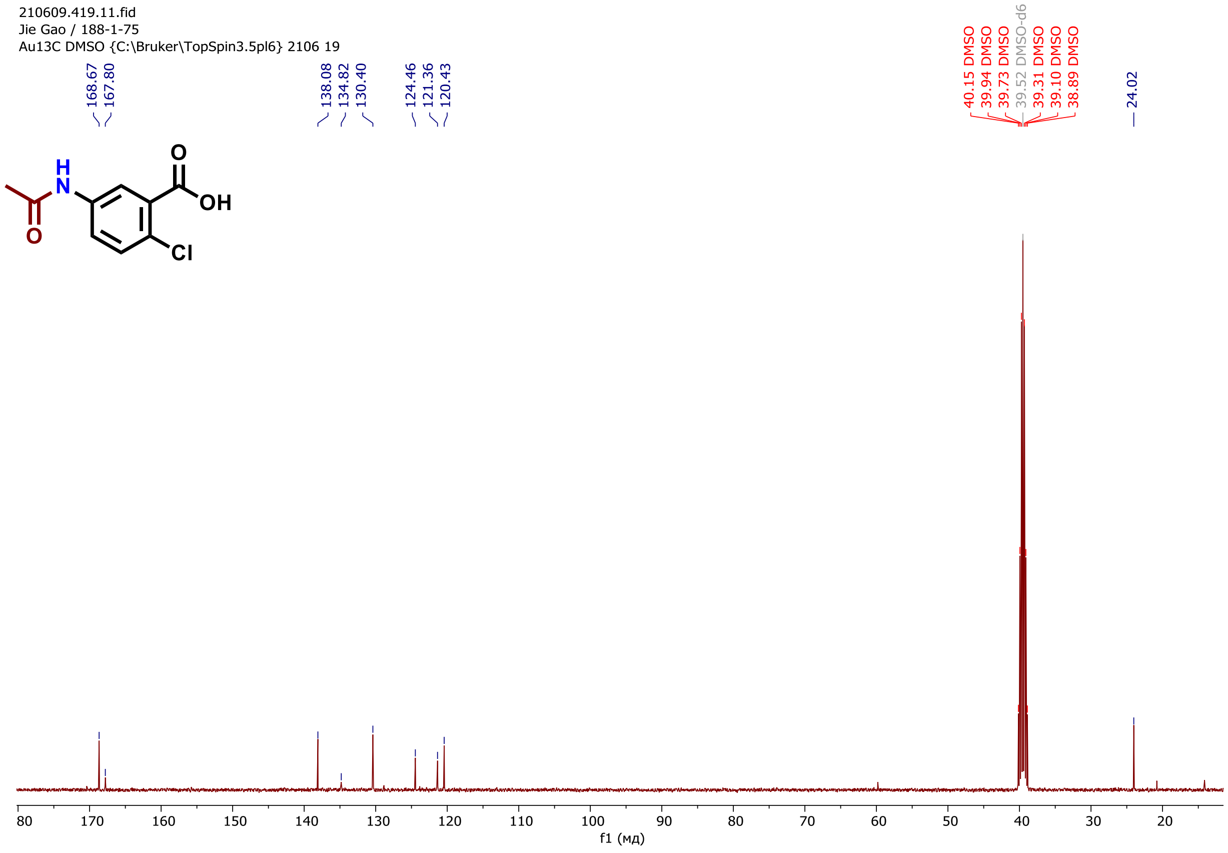

Supplementary Figure 47. NMR spectra of 5-acetamido-2-chlorobenzoic acid.

2-(4-acetamidophenyl)propanoic acid (26)

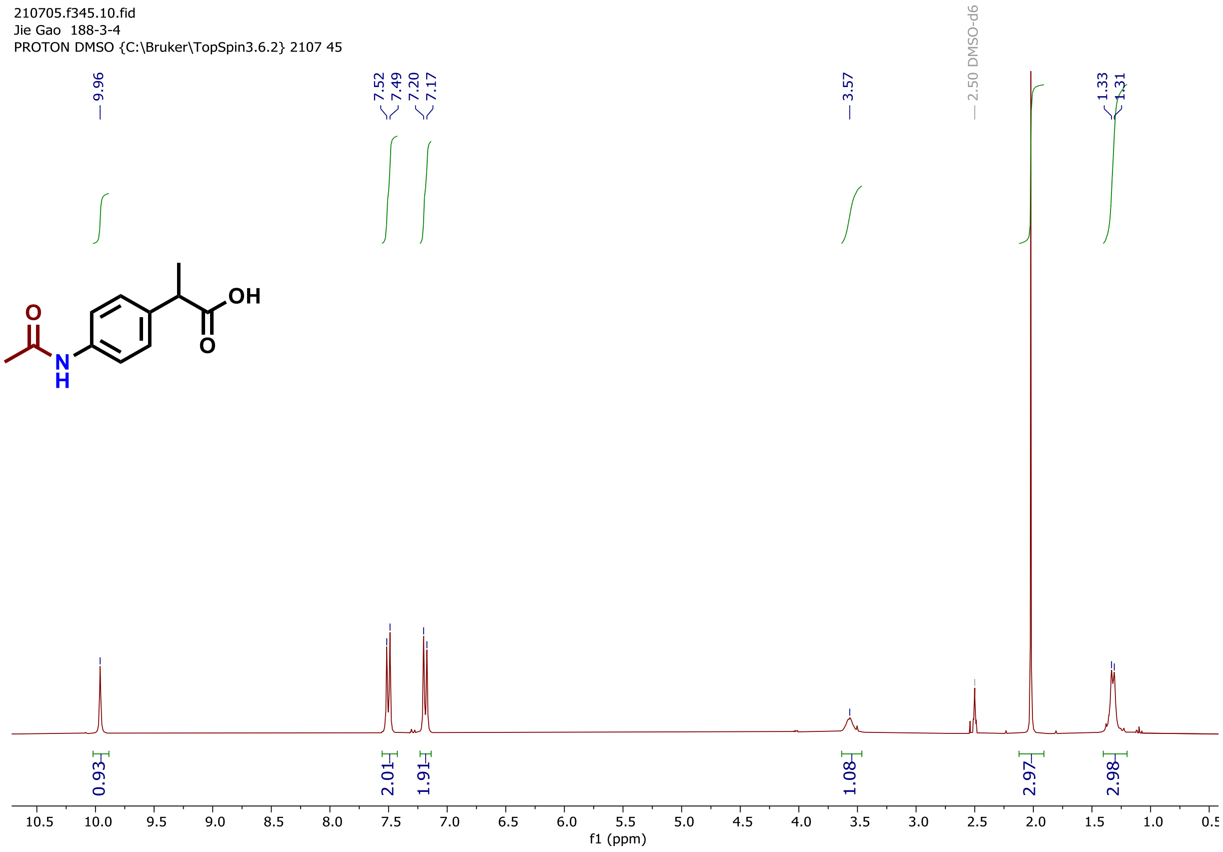

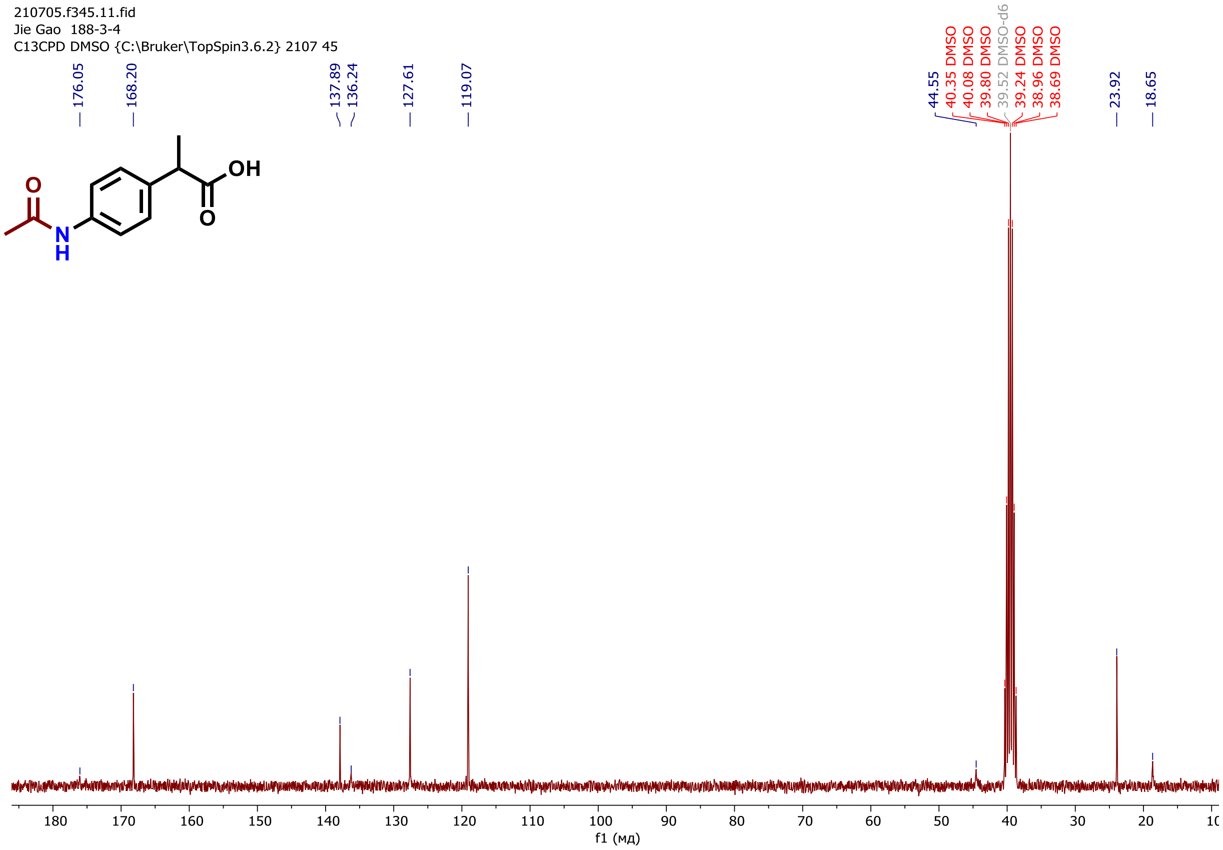

Supplementary Figure 48. NMR spectra of 2-(4-acetamidophenyl)propanoic acid.

ethyl 4-acetamidobenzoate (27)

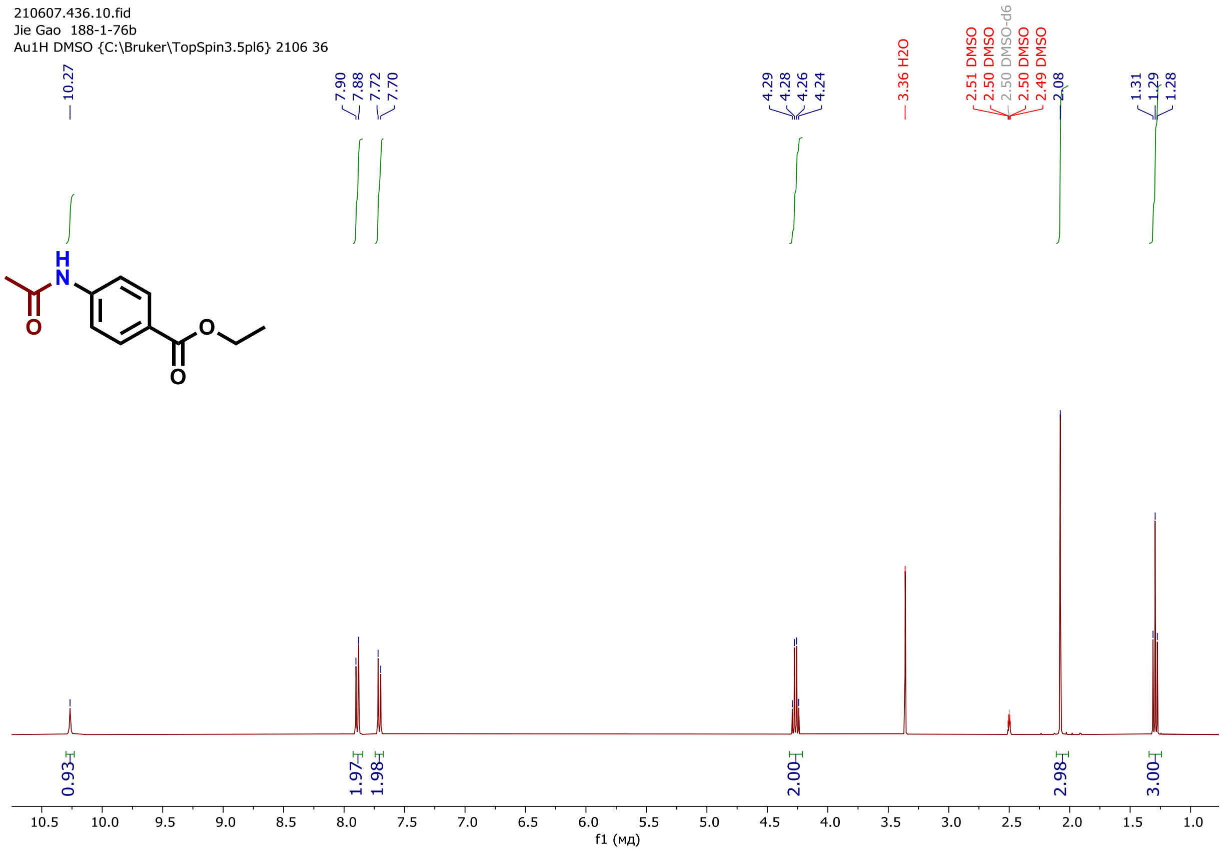

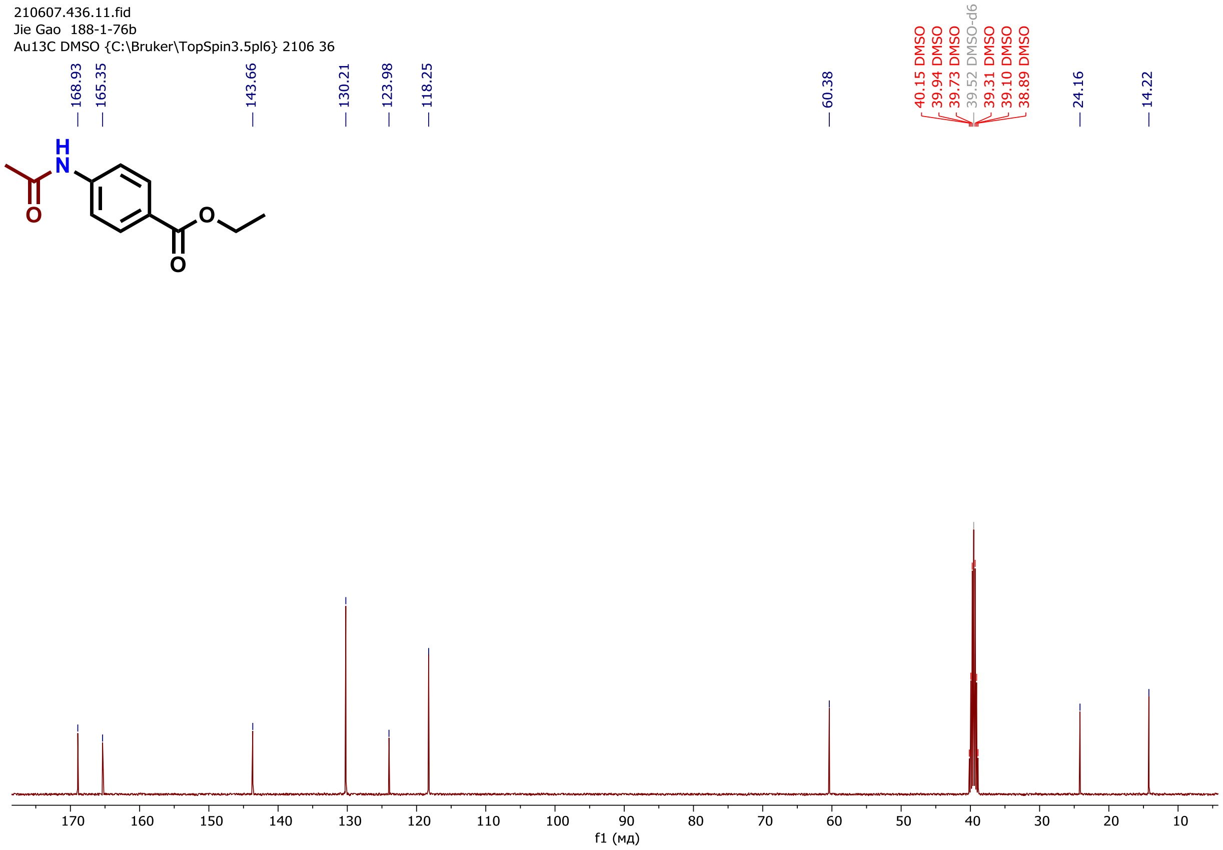

Supplementary Figure 49. NMR spectra of ethyl 4-acetamidobenzoate.

ethyl 3-(4-acetamidophenyl)propanoate (28)

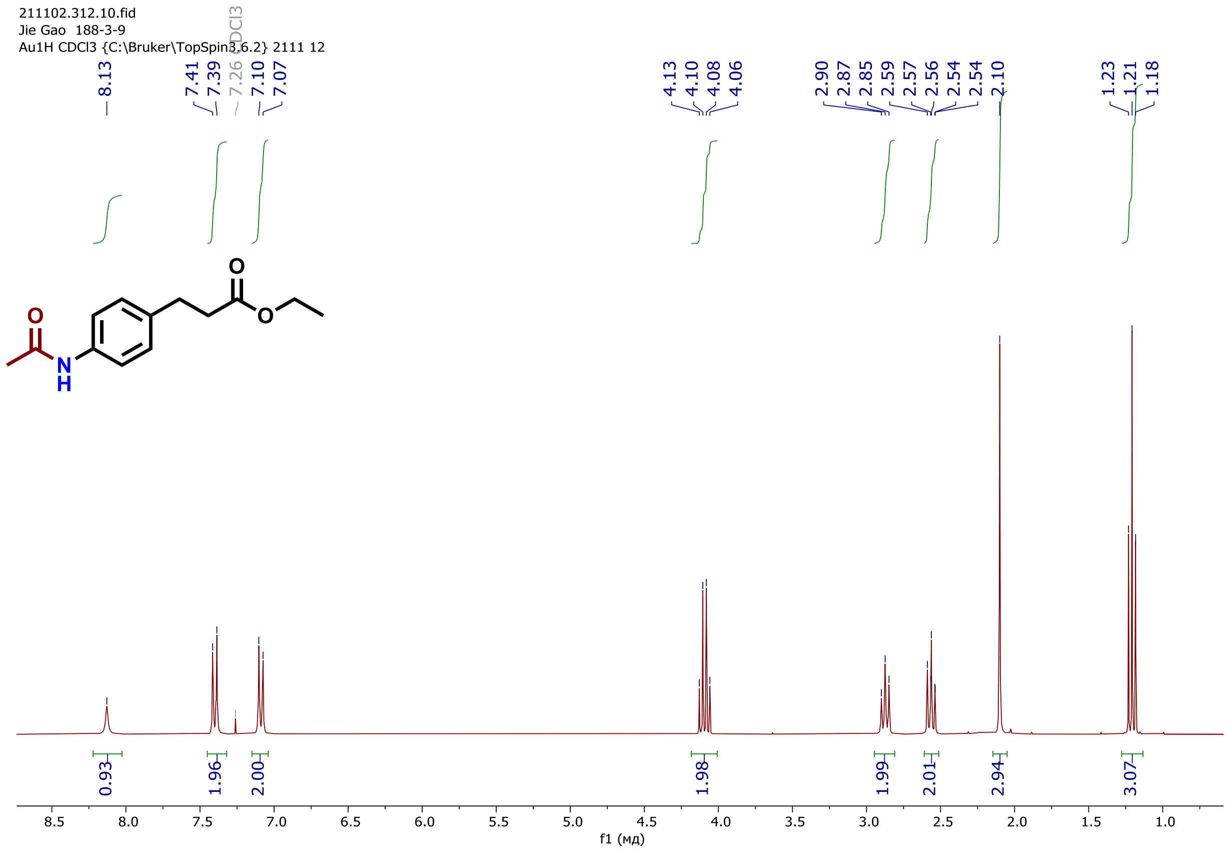

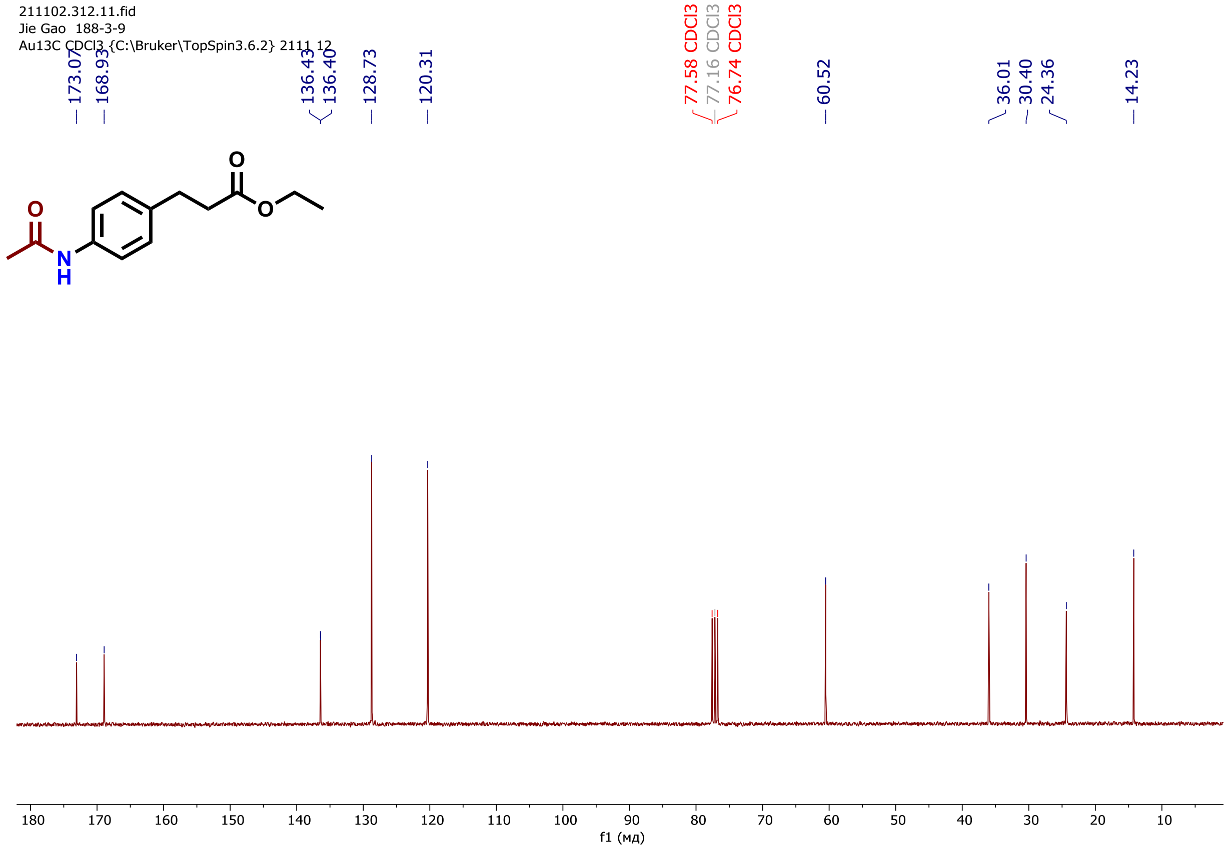

Supplementary Figure 50. NMR spectra of ethyl 3-(4-acetamidophenyl)propanoate .

methyl 4-acetamidobenzoate (29)

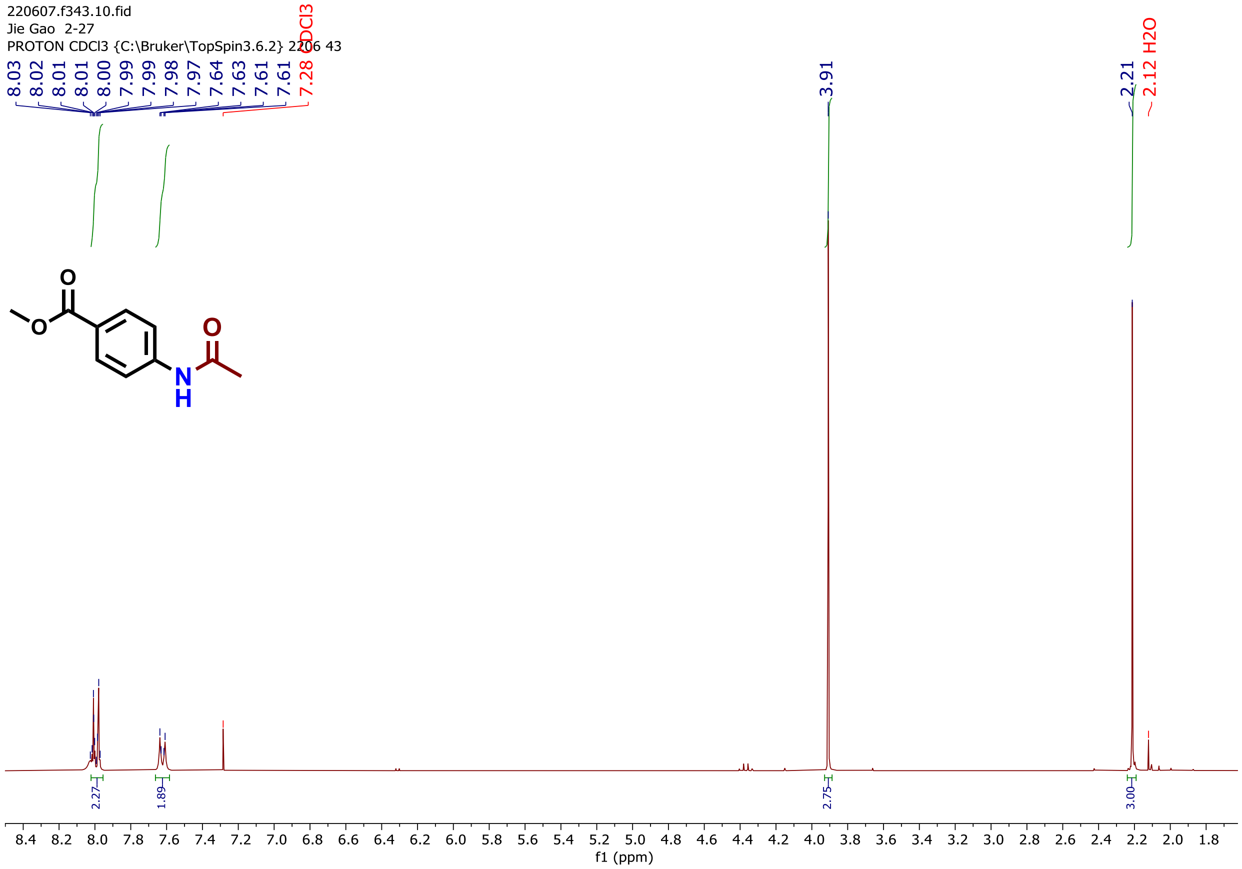

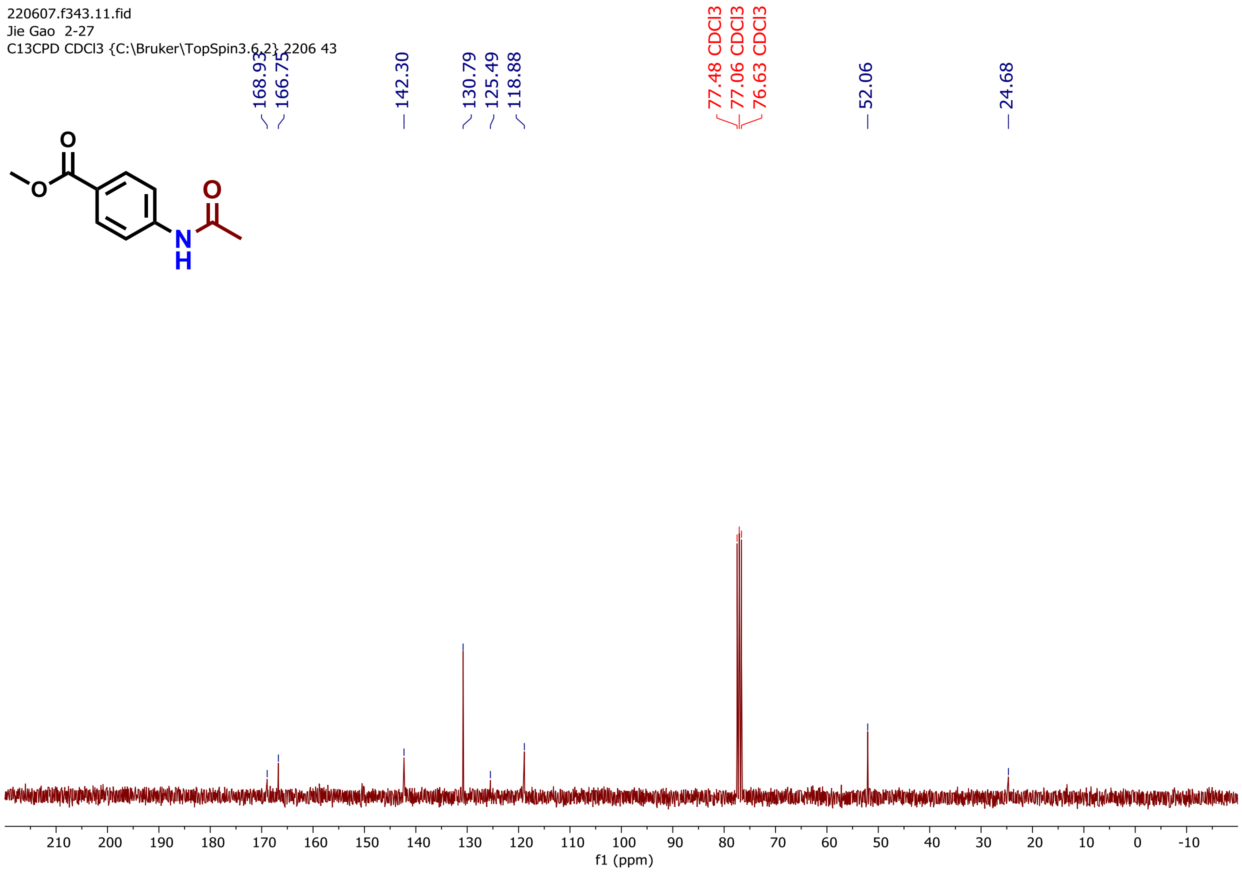

Supplementary Figure 51. NMR spectra of methyl 4-acetamidobenzoate.

N-(4-cyanophenyl)acetamide (30)

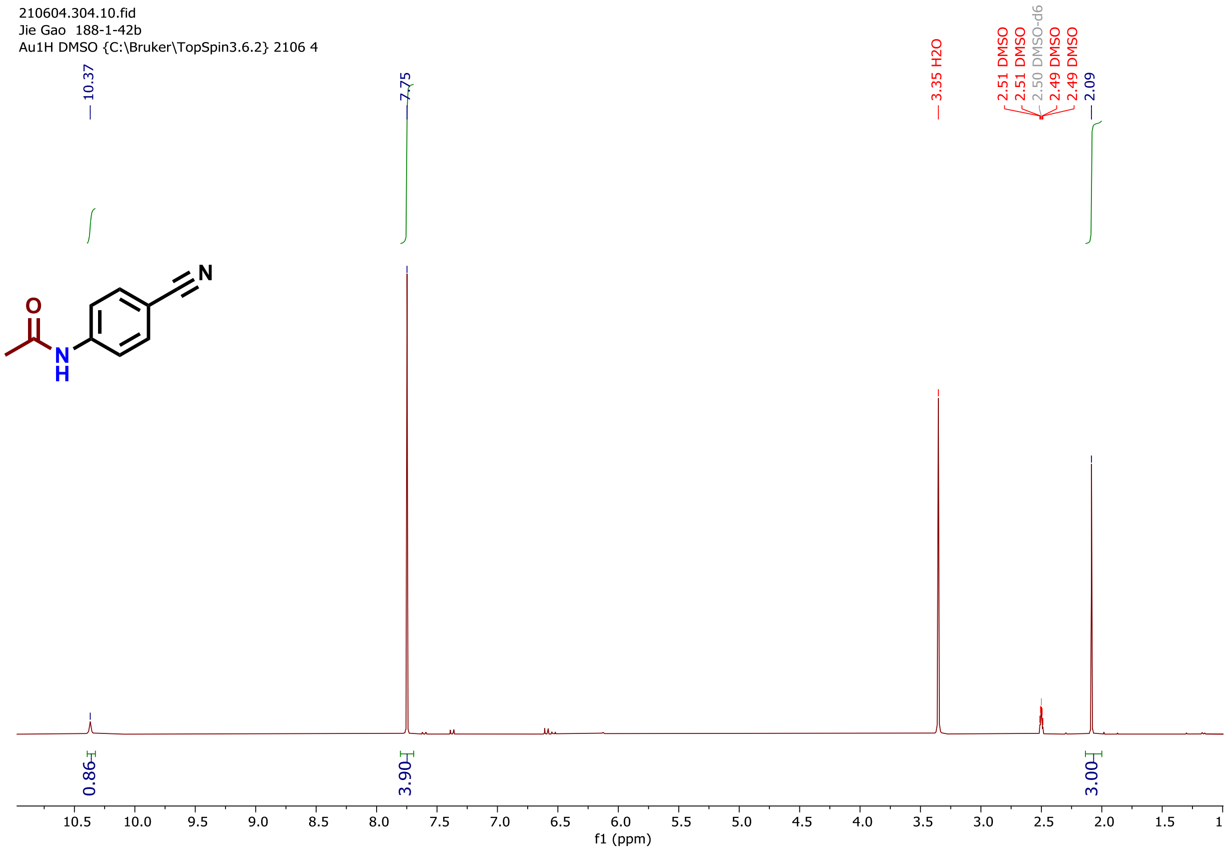

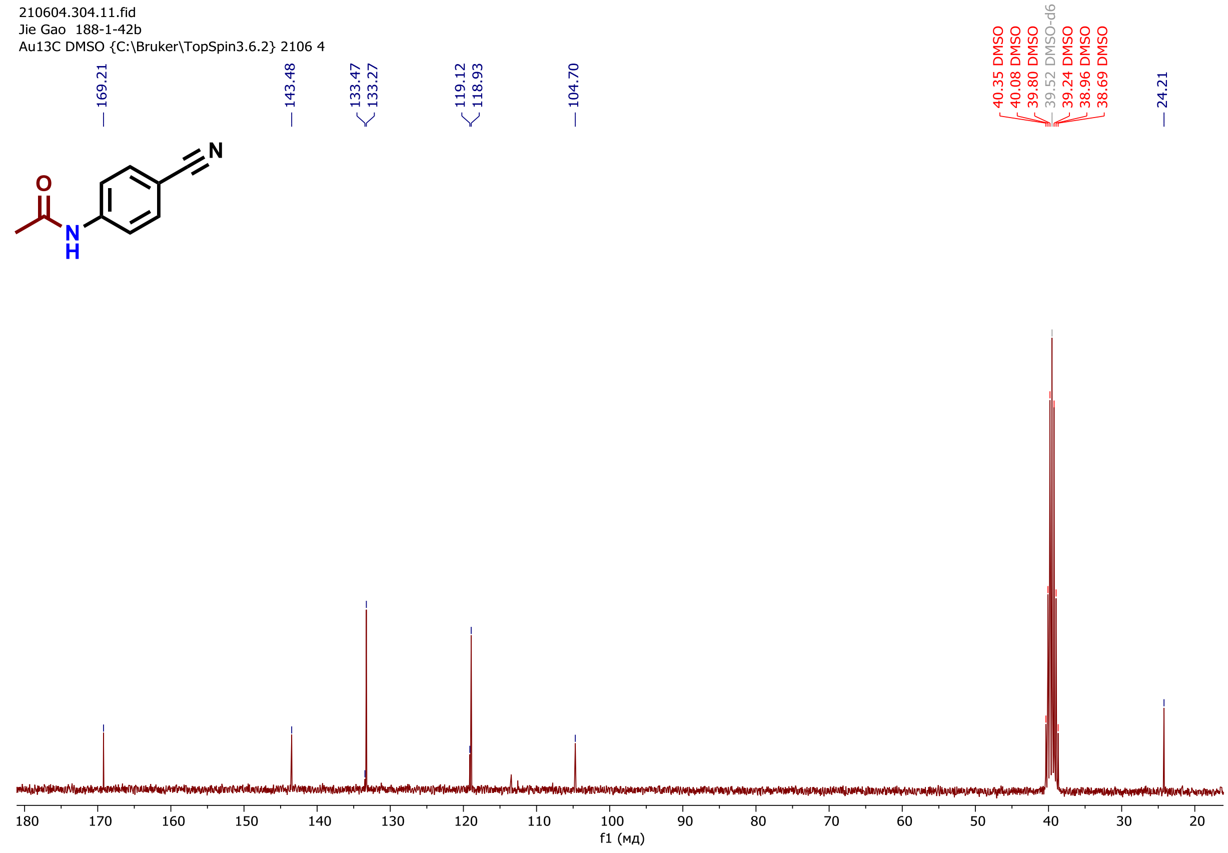

Supplementary Figure 52. NMR spectra of N-(4-cyanophenyl)acetamide.

N-(2-oxo-2H-chromen-6-yl)acetamide (31)

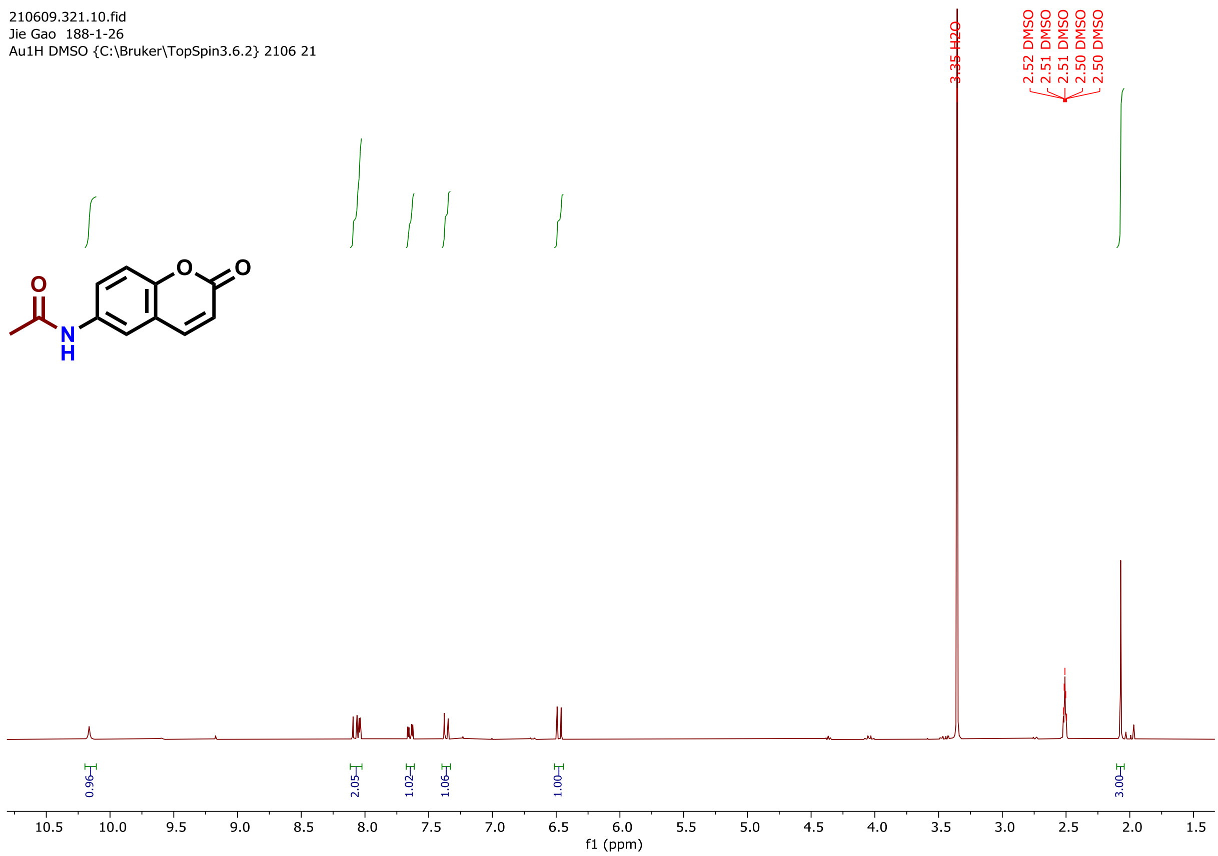

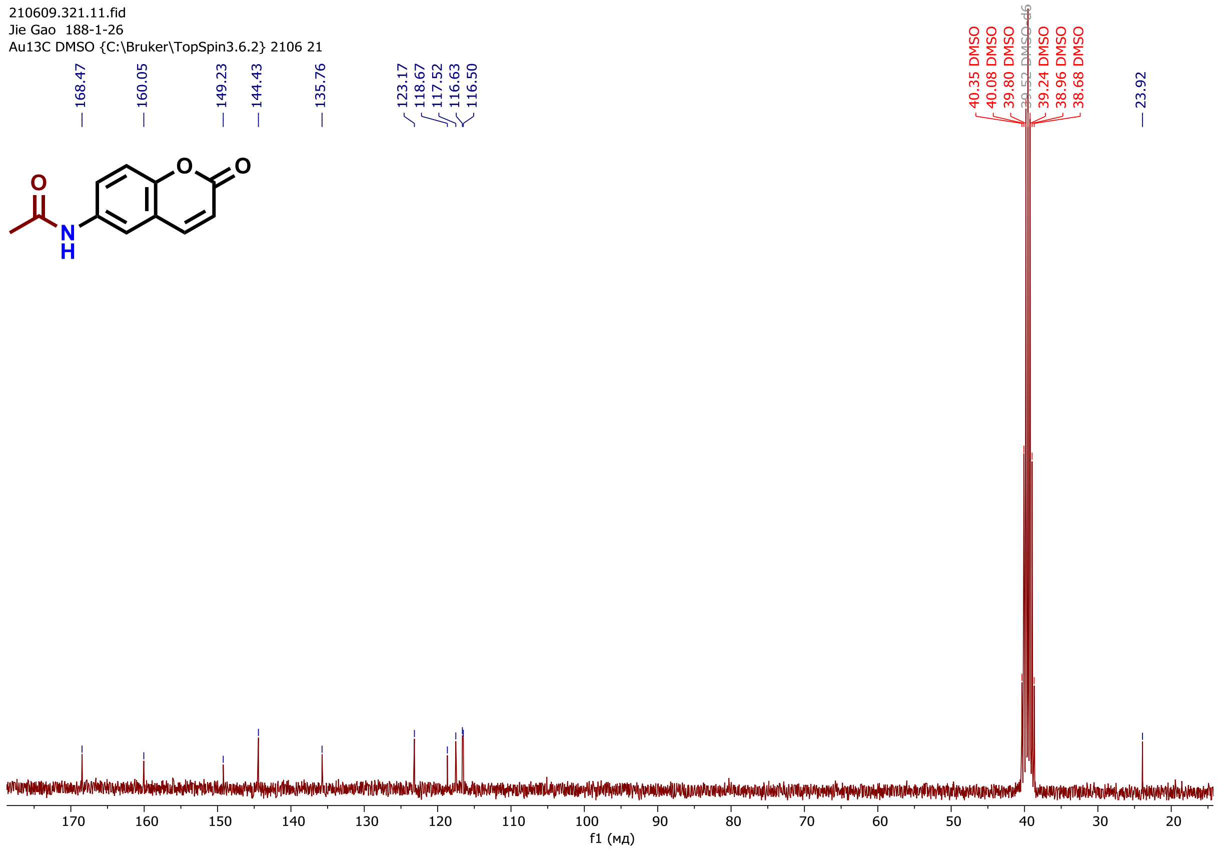

Supplementary Figure 53. NMR spectra of N-(2-oxo-2H-chromen-6-yl)acetamide.

N-(quinolin-8-yl)acetamide (32)

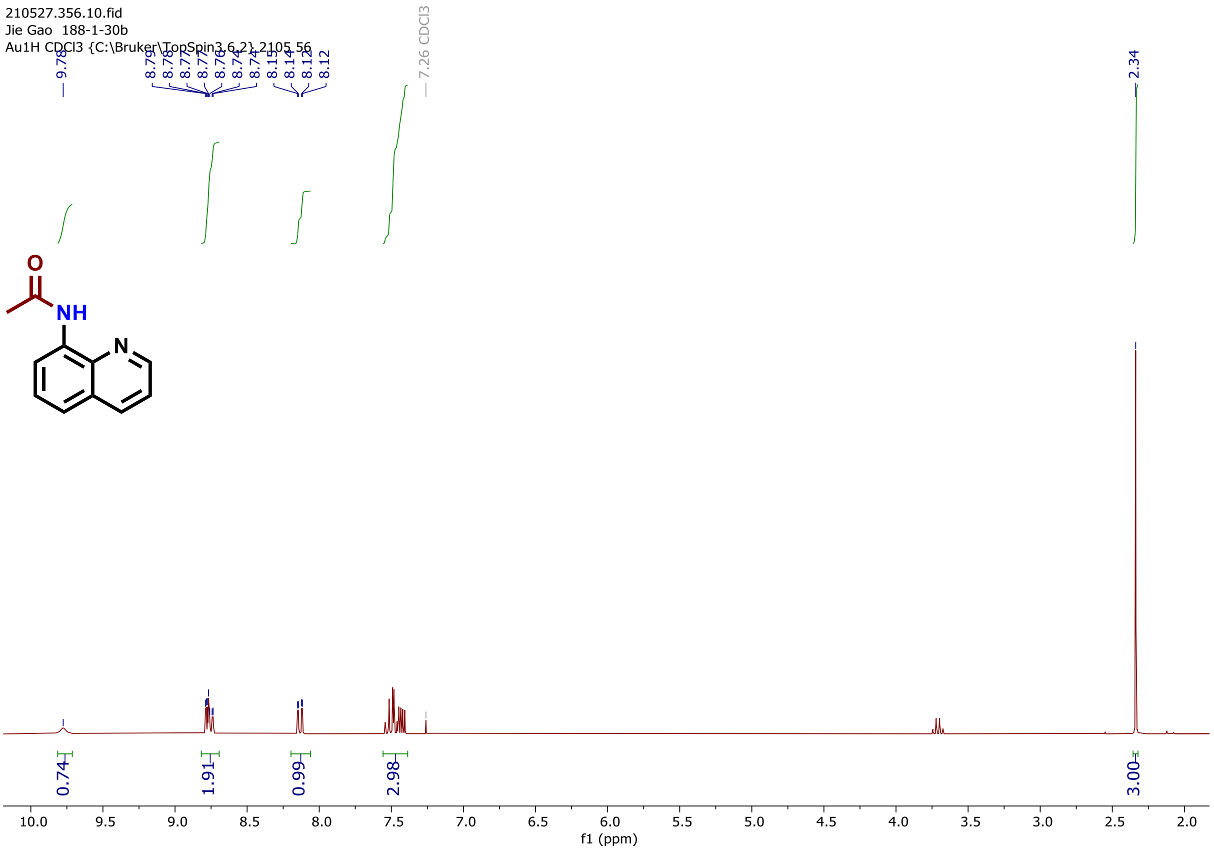

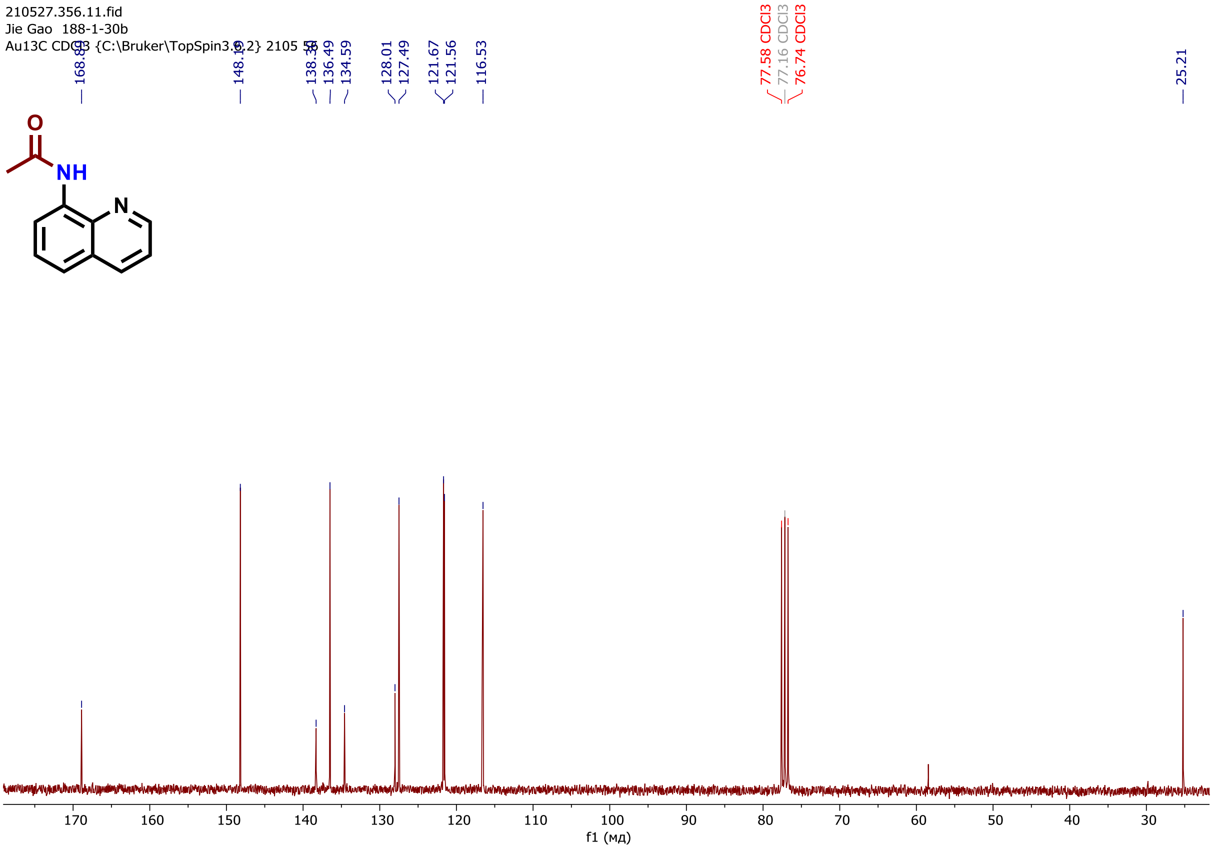

Supplementary Figure 54. NMR spectra of N-(quinolin-8-yl)acetamide.

N-(pyridin-3-yl)acetamide (33)

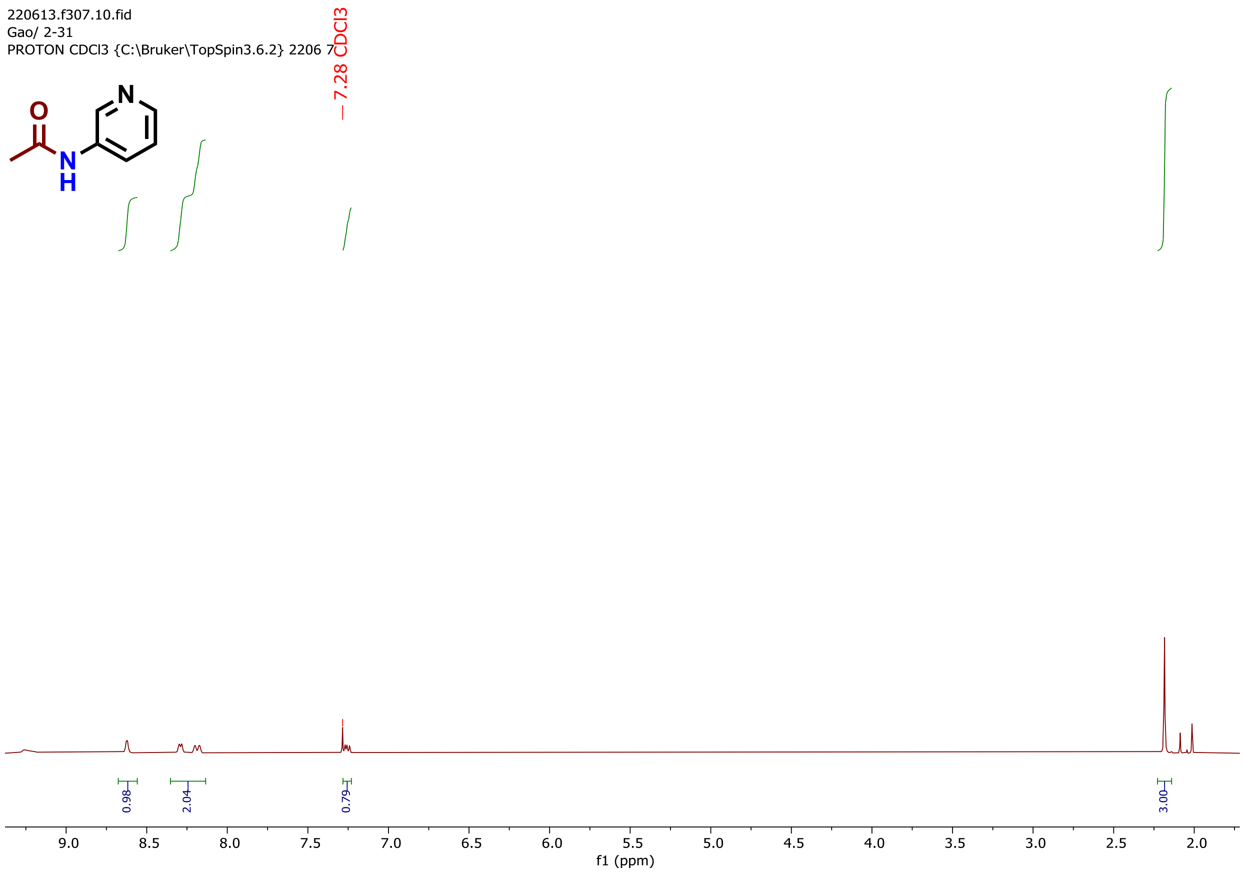

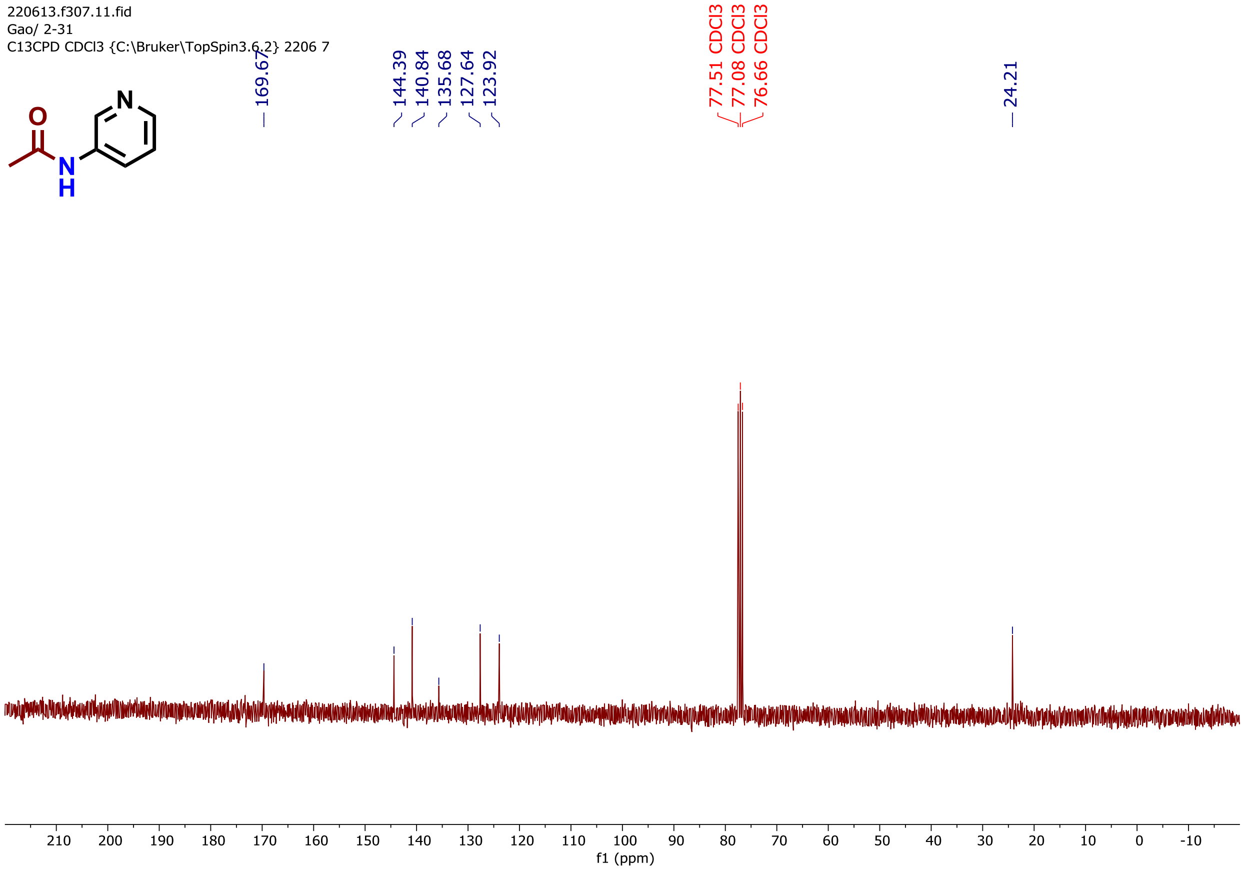

Supplementary Figure 55. NMR spectra of N-(pyridin-3-yl)acetamide.

N-(2-(4-fluorophenoxy)pyridin-3-yl)acetamide (34)

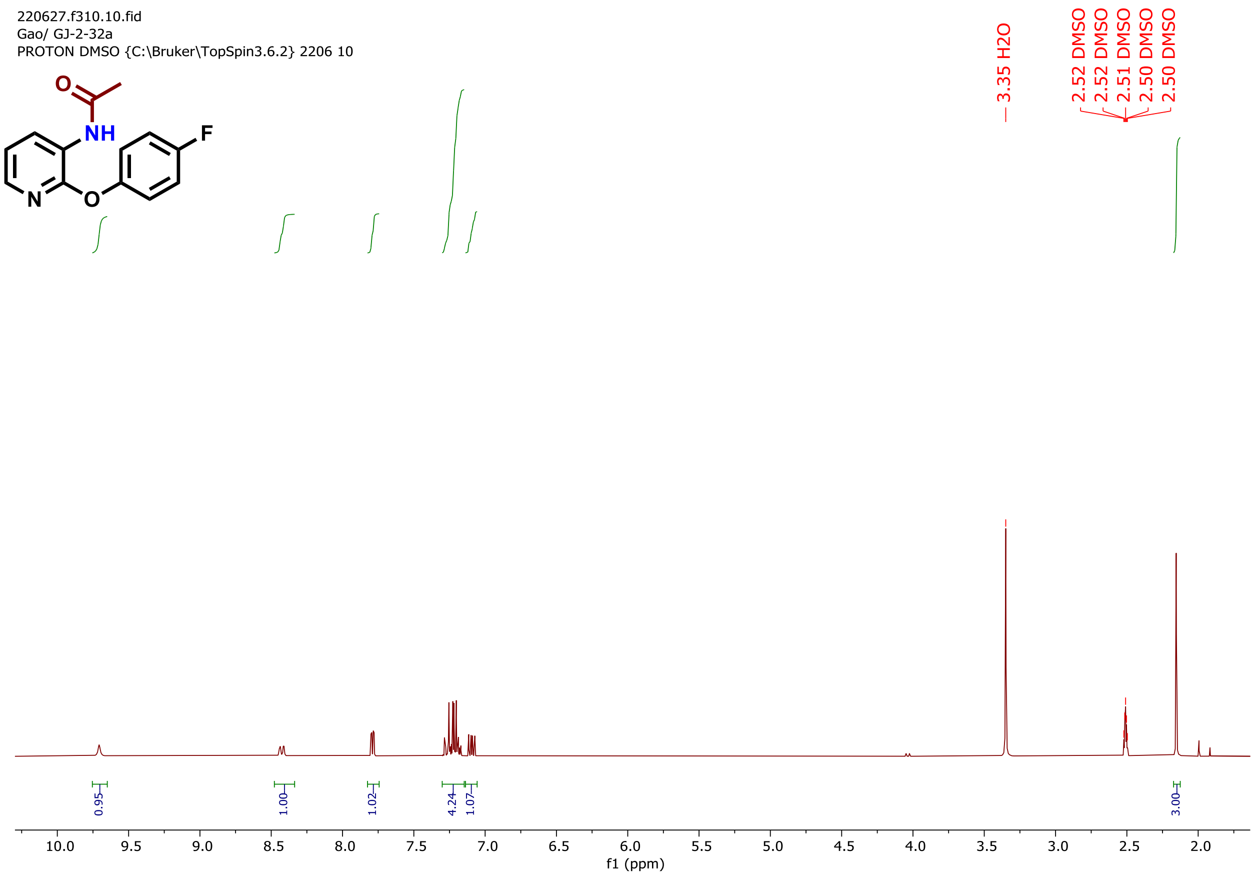

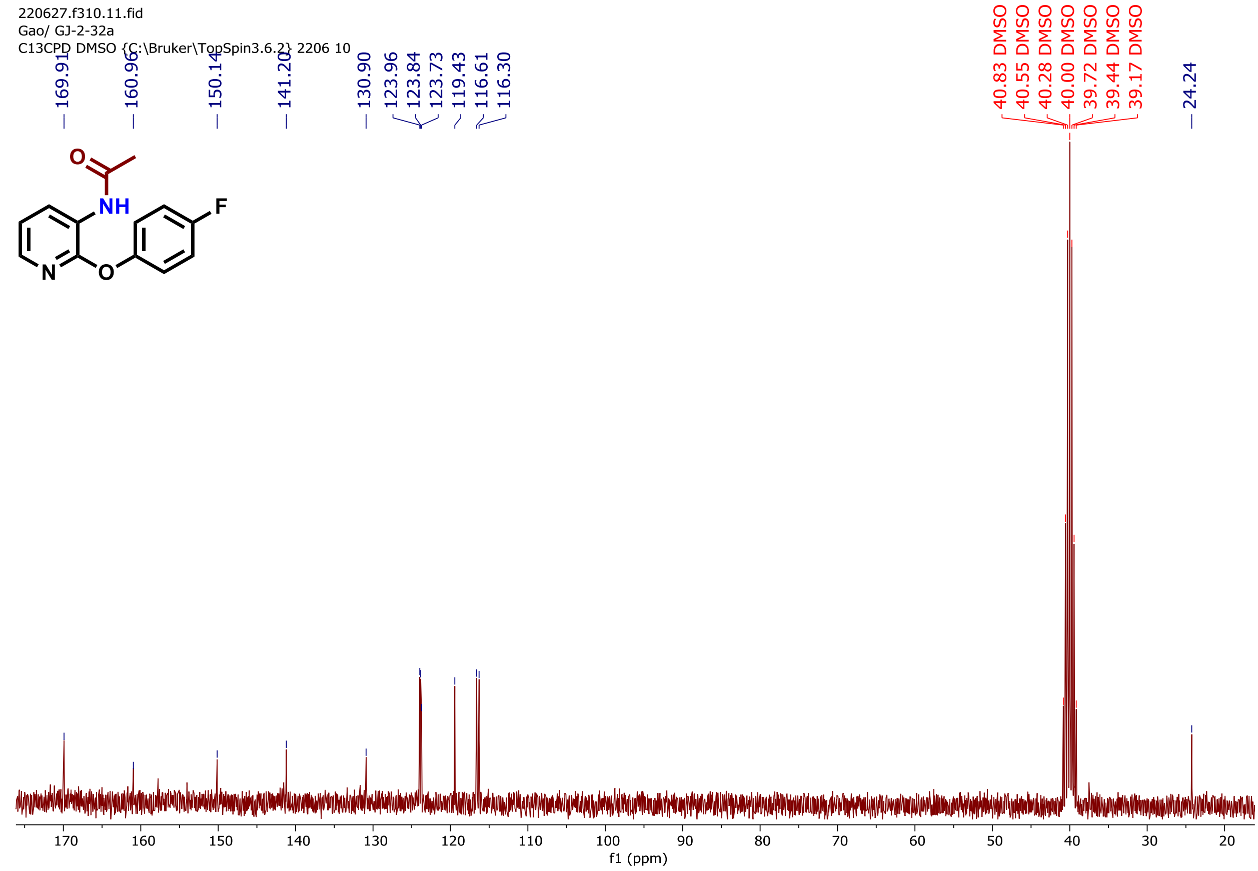

Supplementary Figure 56. NMR spectra of N-(2-(4-fluorophenoxy)pyridin-3-yl)acetamide.

N-(1H-indol-5-yl)acetamide (35)

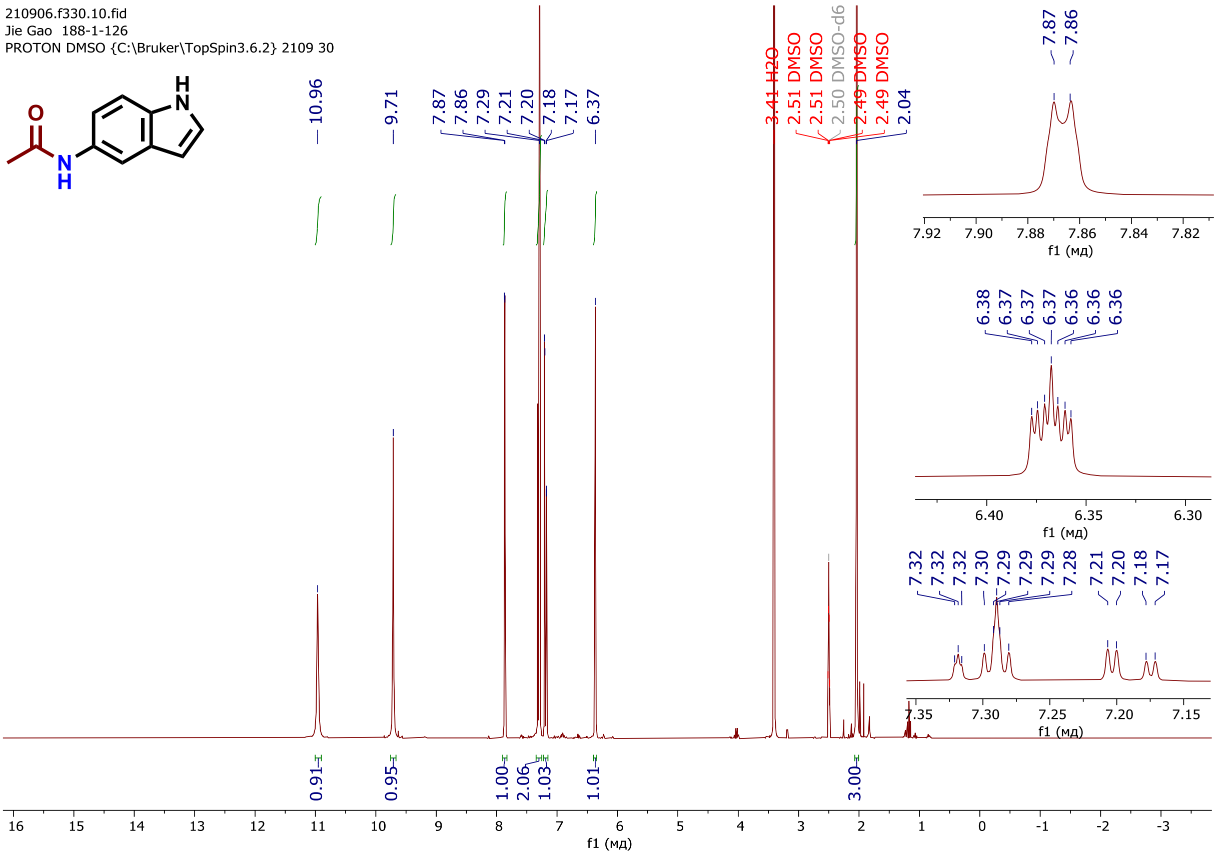

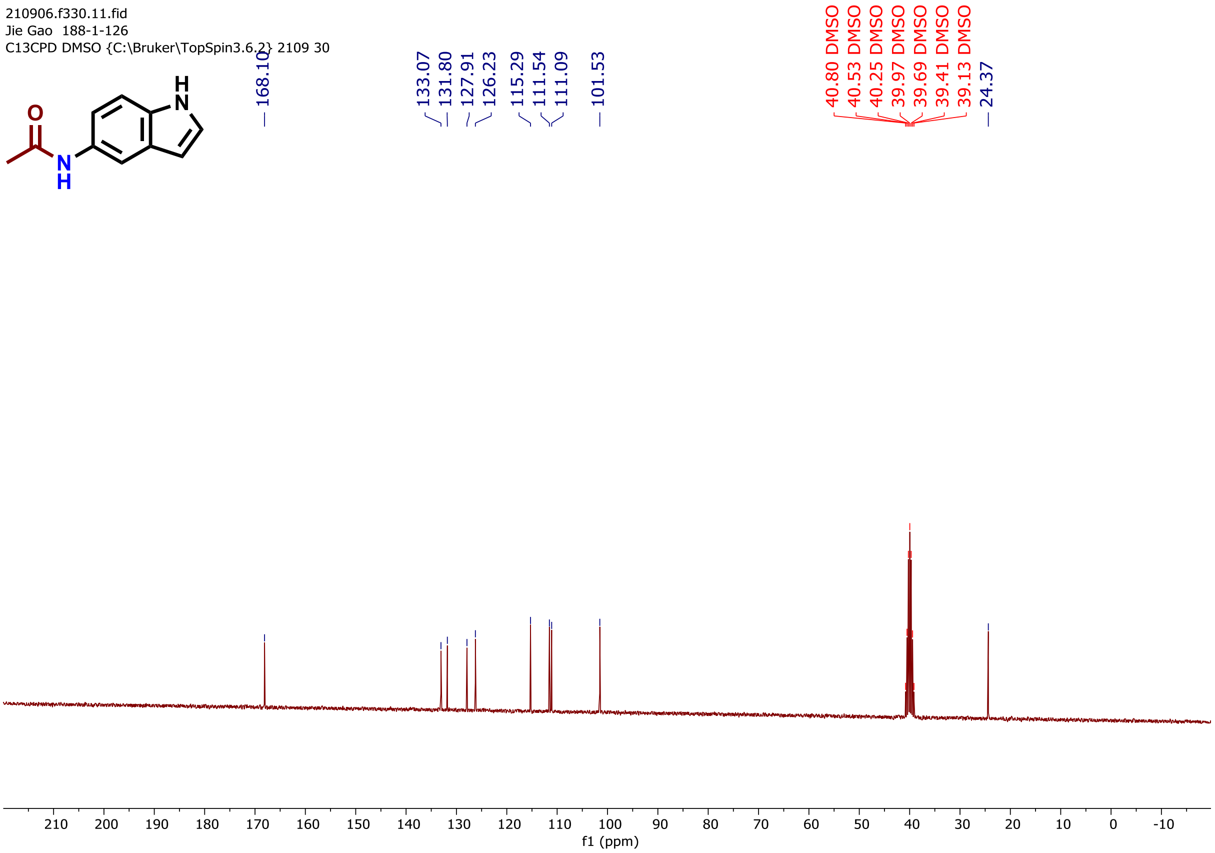

Supplementary Figure 57. NMR spectra of N-(1H-indol-5-yl)acetamide.

N-(benzo[d]thiazol-6-yl)acetamide (36)

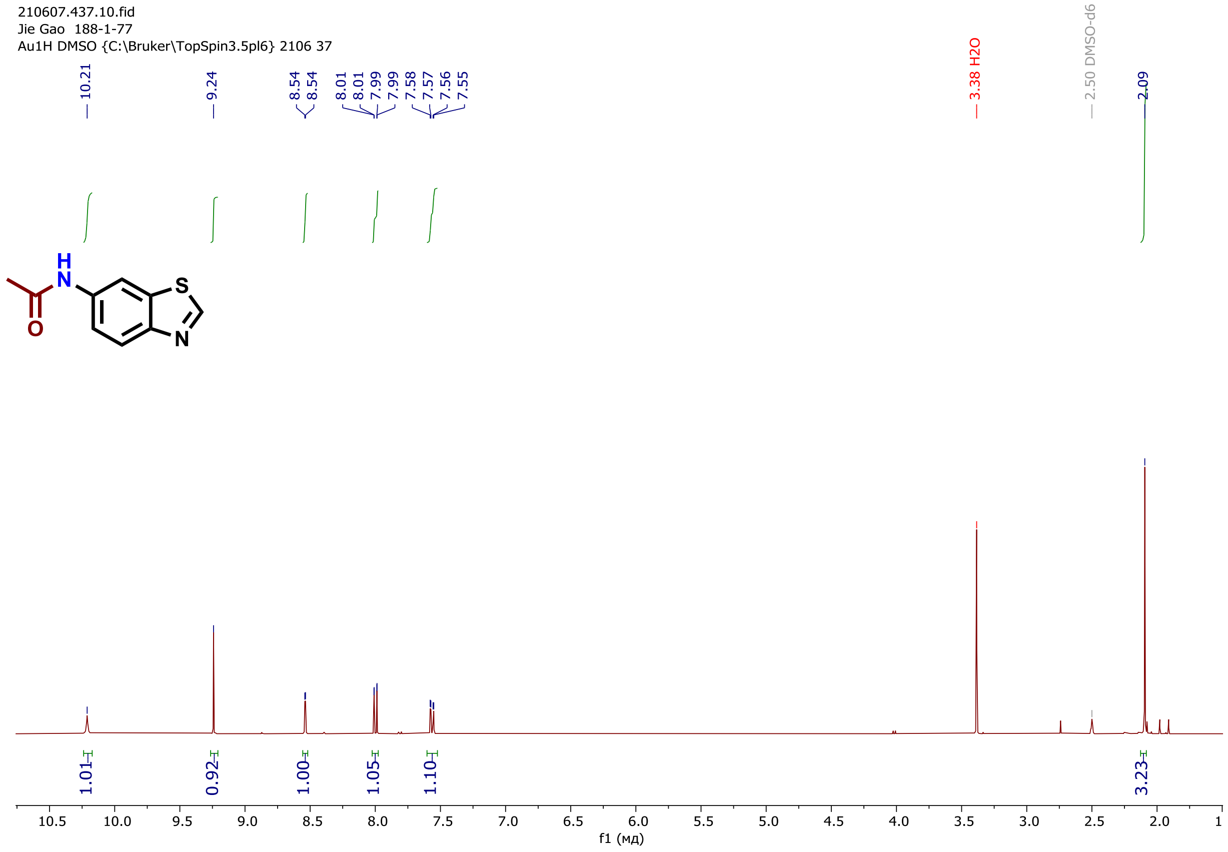

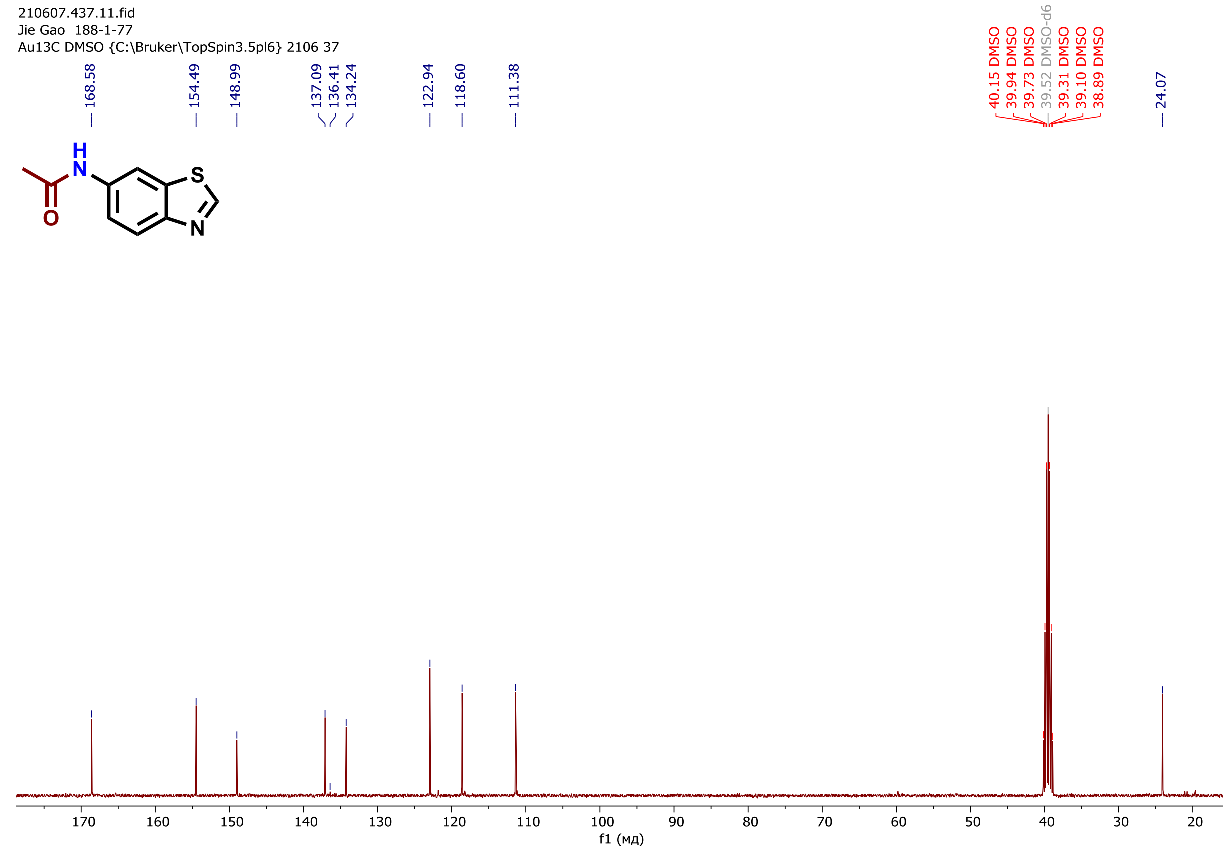

Supplementary Figure 58. NMR spectra of N-(benzo[d]thiazol-6-yl)acetamide.

N-(3-vinylphenyl)acetamide (37)

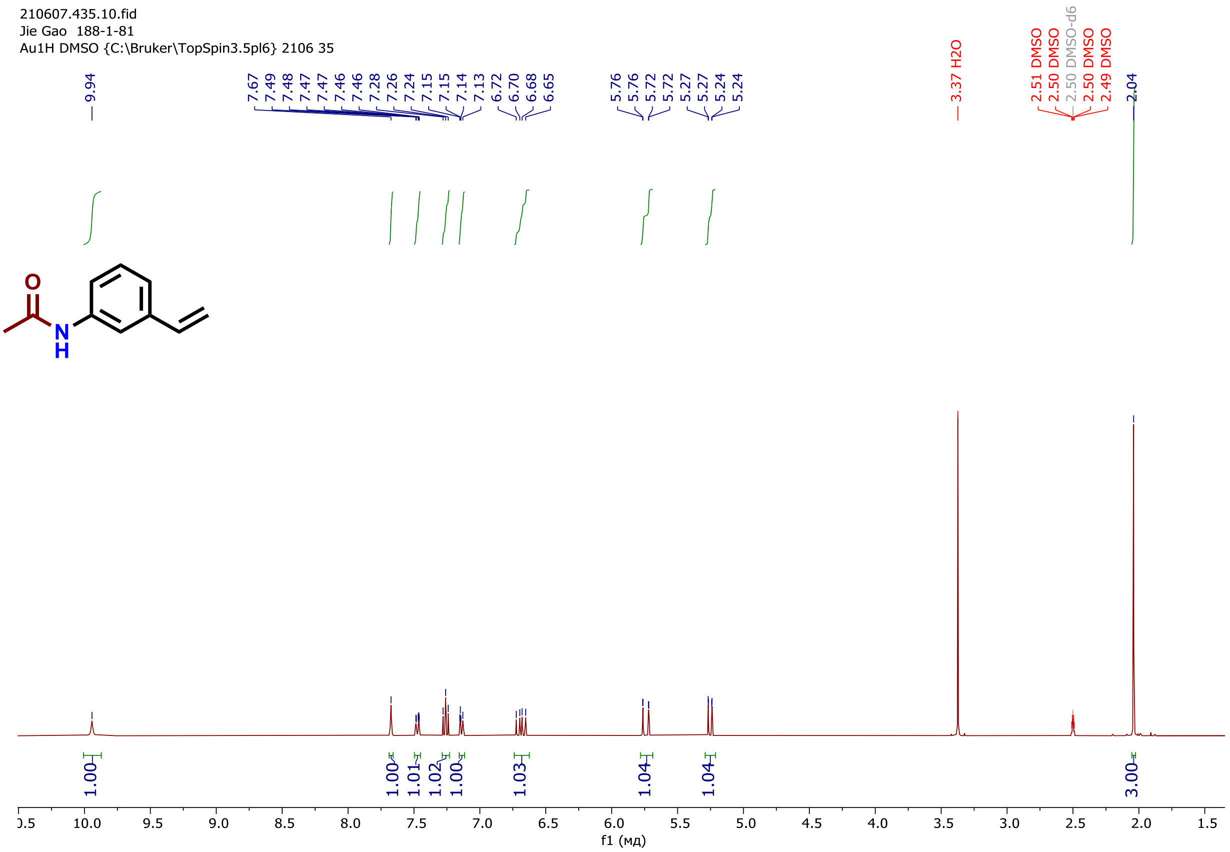

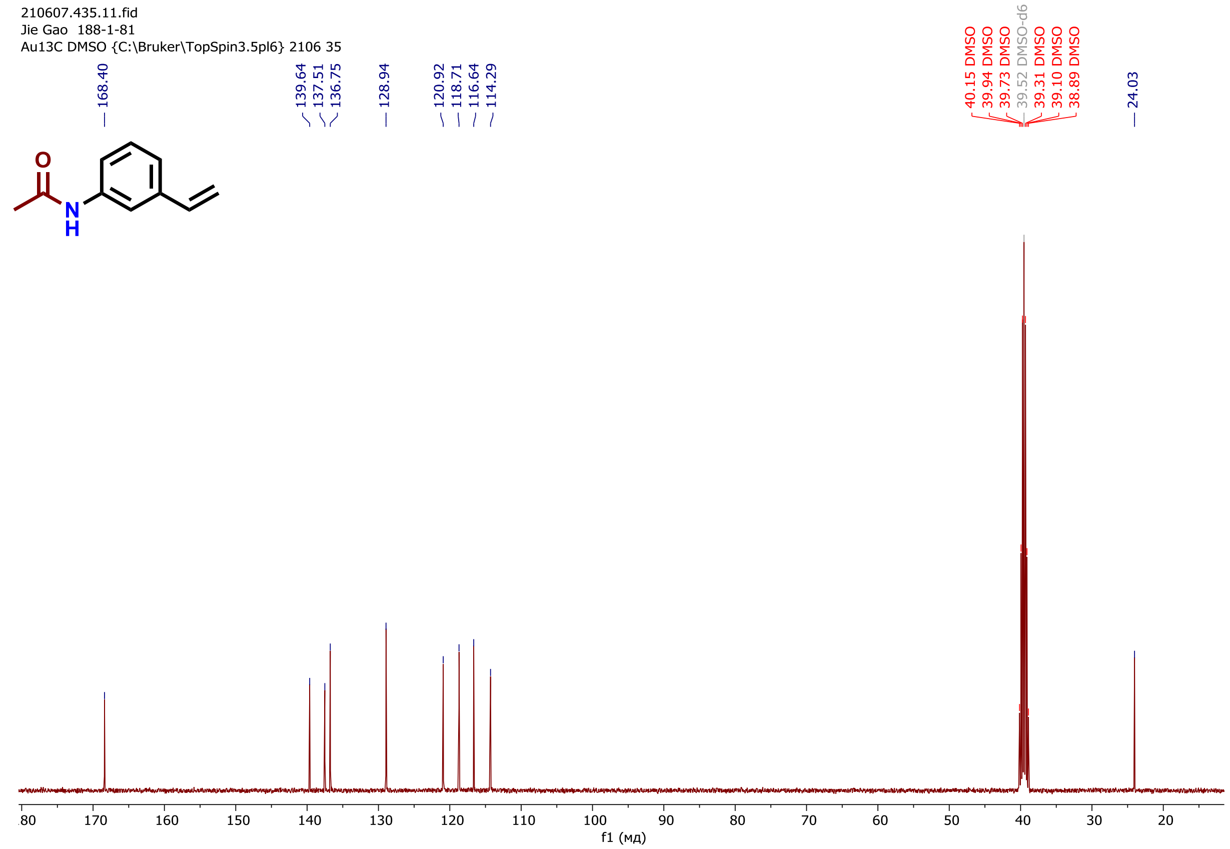

Supplementary Figure 59. NMR spectra of N-(3-vinylphenyl)acetamide.

N-phenylpropionamide (38)

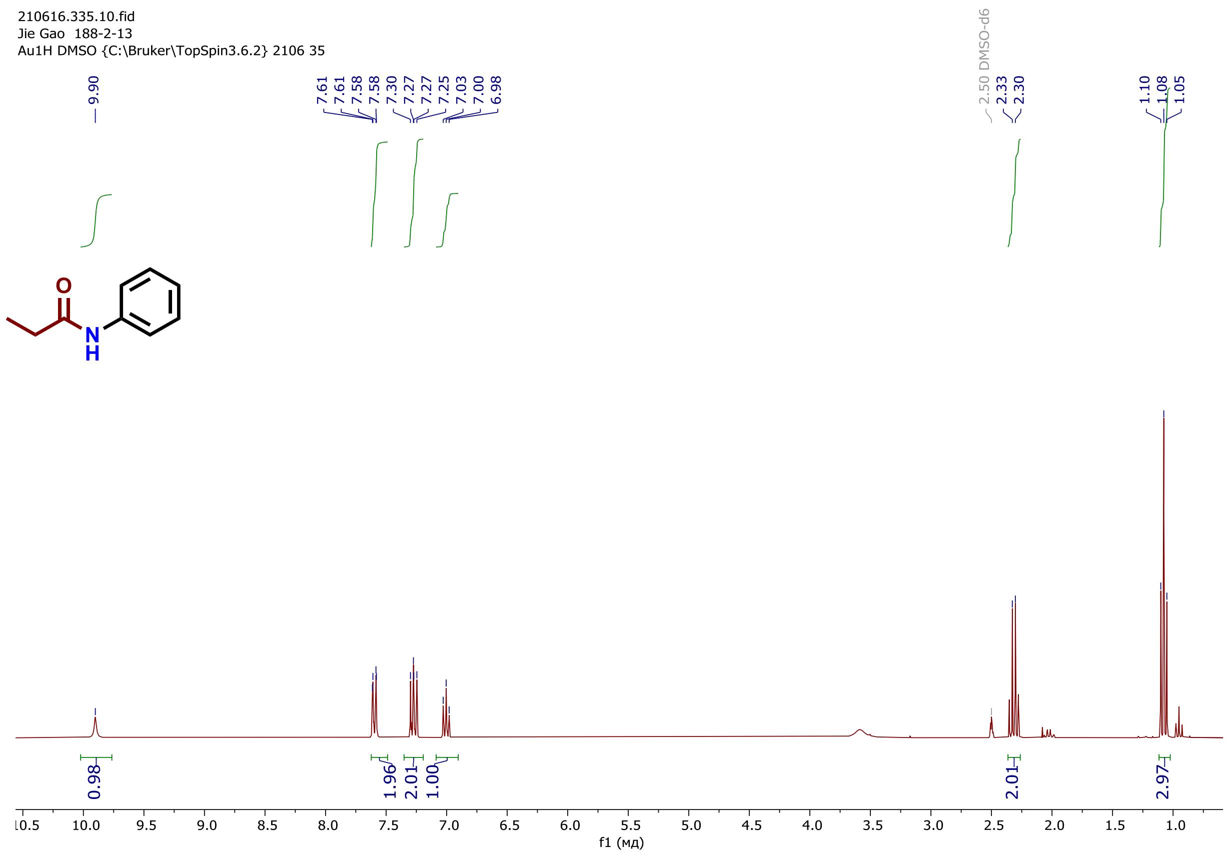

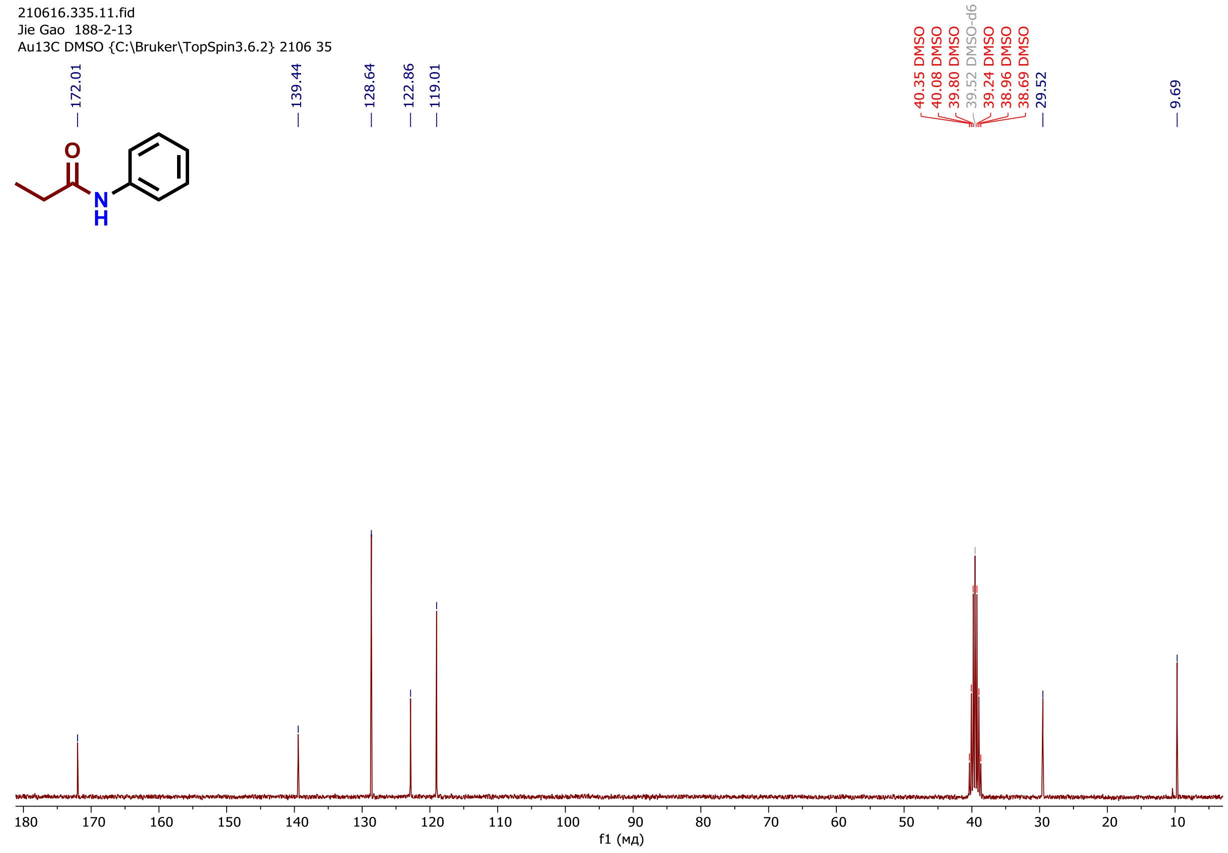

Supplementary Figure 60. NMR spectra of N-phenylpropionamide.

N-phenylhexanamide (39)

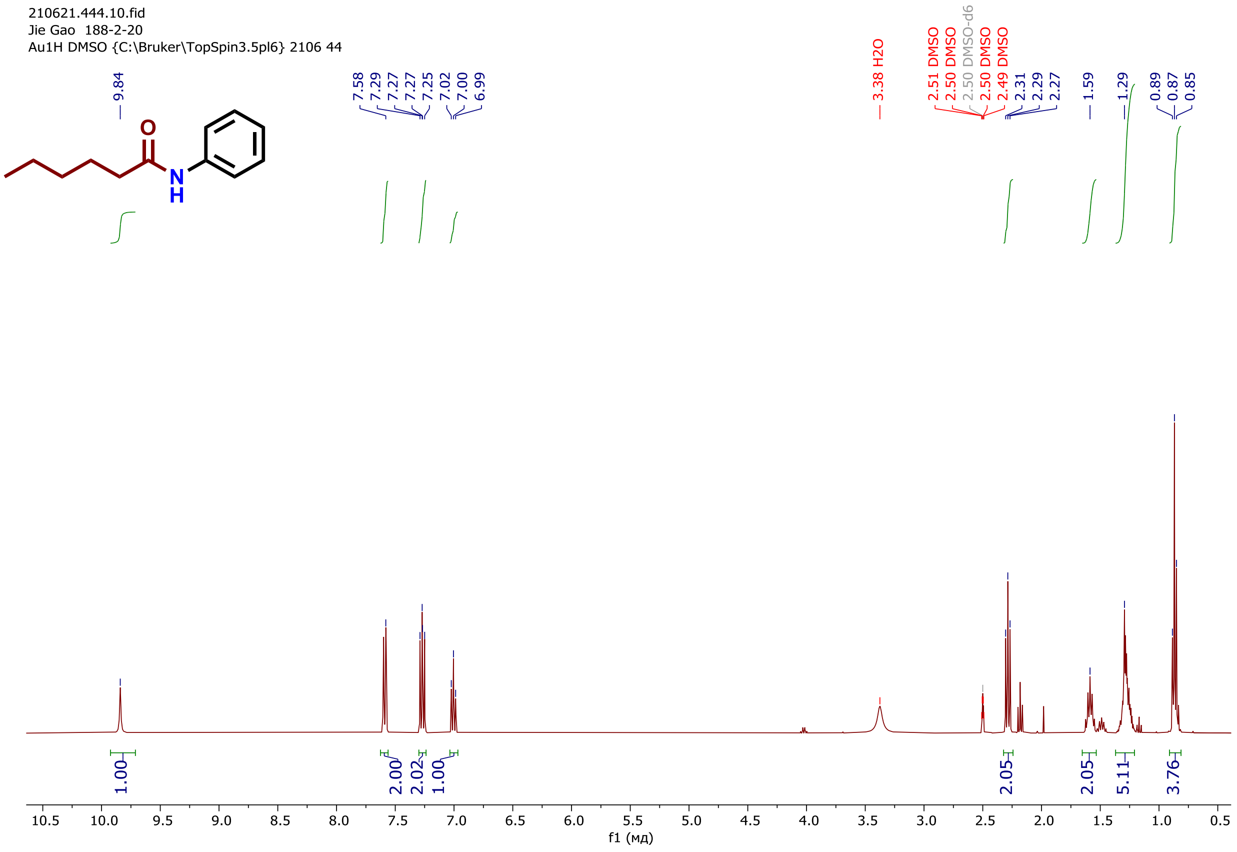

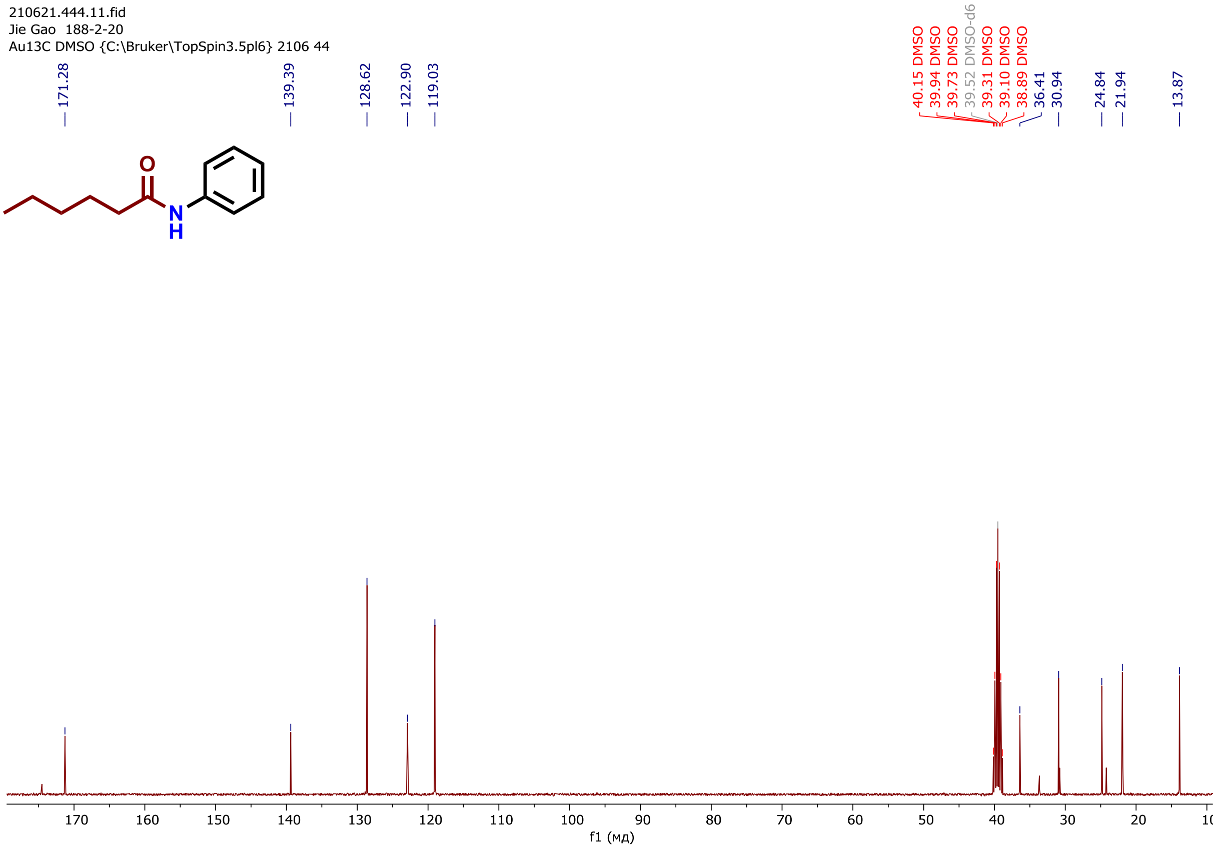

Supplementary Figure 61. NMR spectra of N-phenylhexanamide.

3-mercapto-N-phenylpropanamide (40)

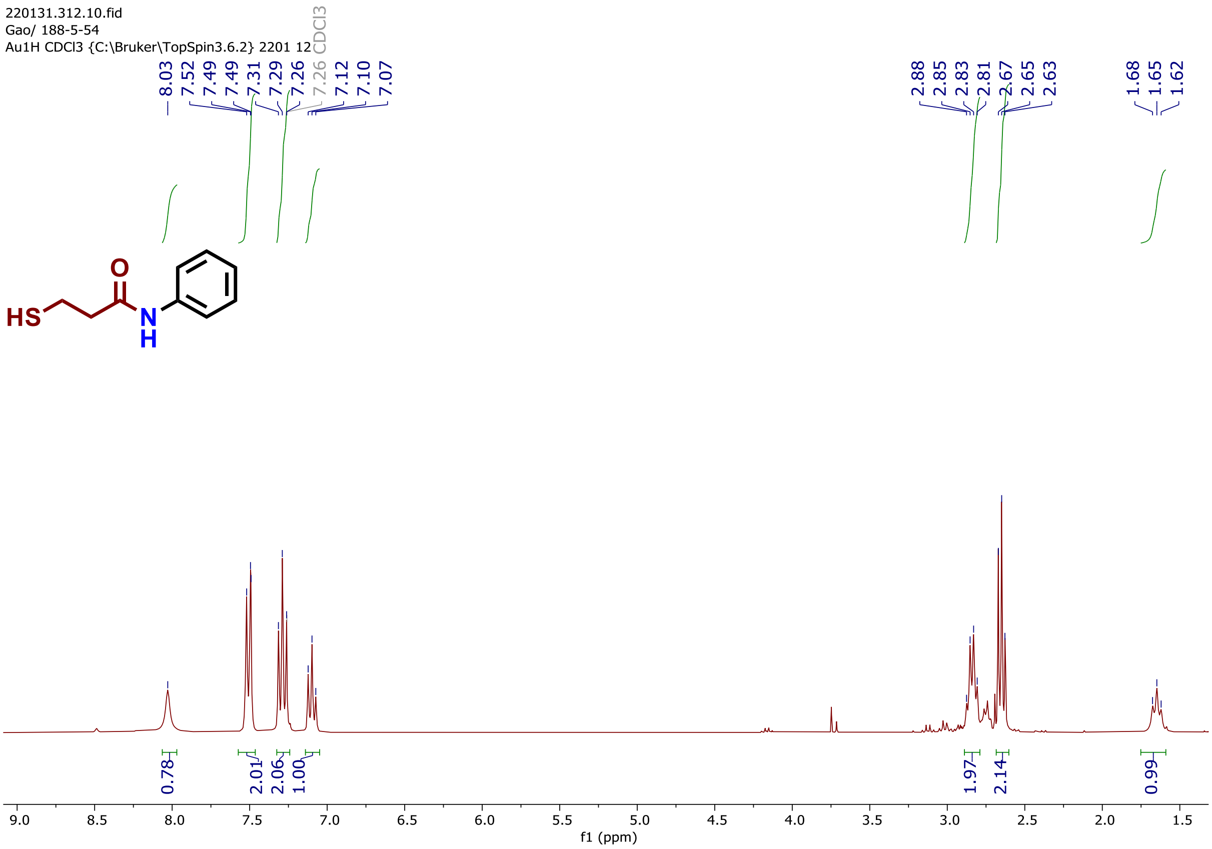

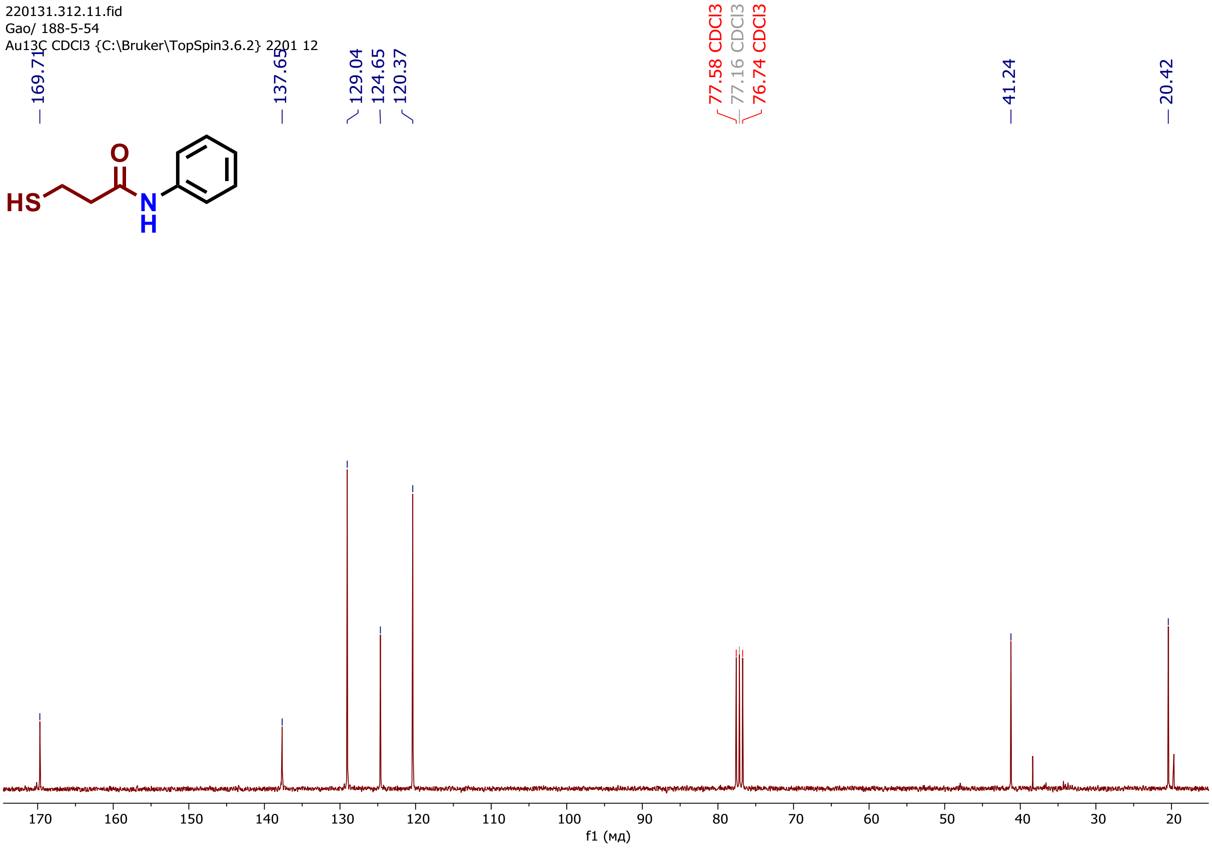

Supplementary Figure 62. NMR spectra of 3-mercapto-N-phenylpropanamide.

N-phenylcyclopropanecarboxamide (41)

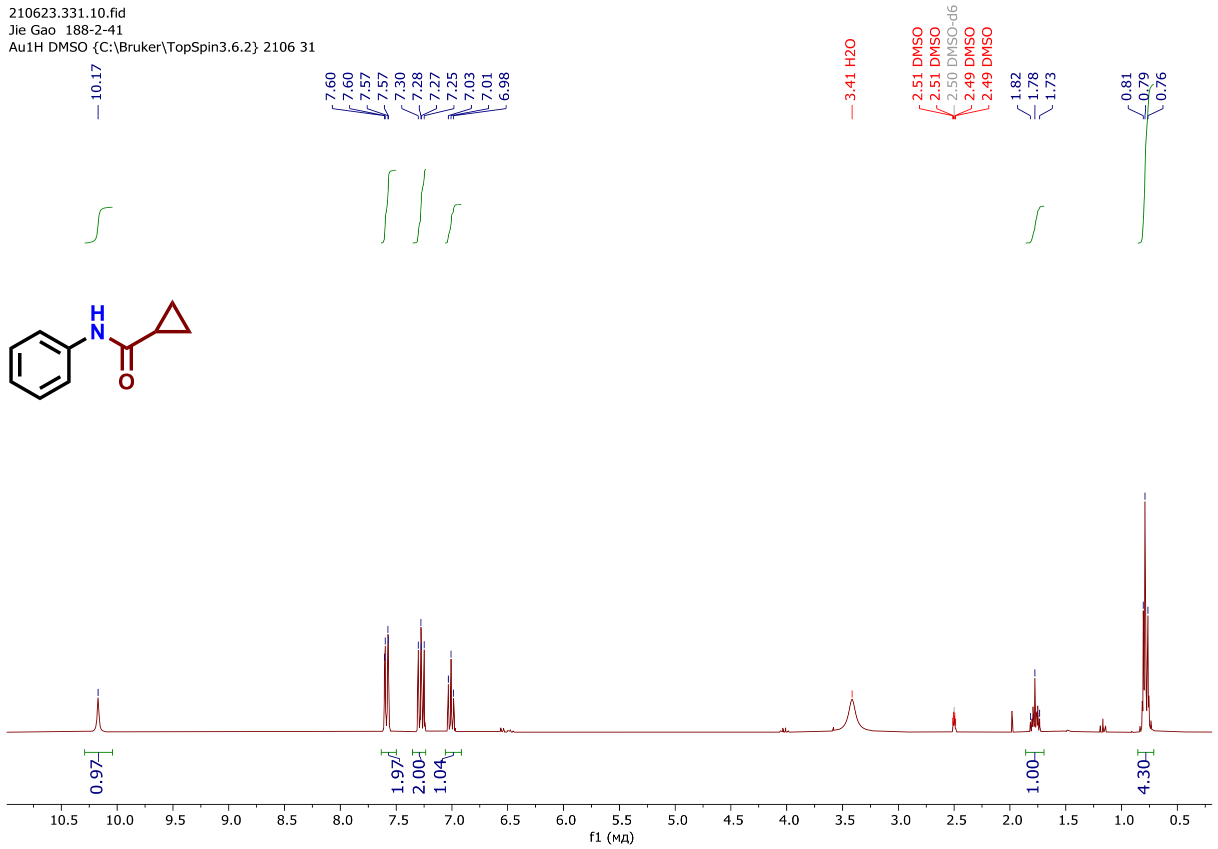

^{13}C NMR (75 MHz, DMSO) δ 172.09, 139.82, 129.13, 123.36, 119.44, 14.99, 7.57.

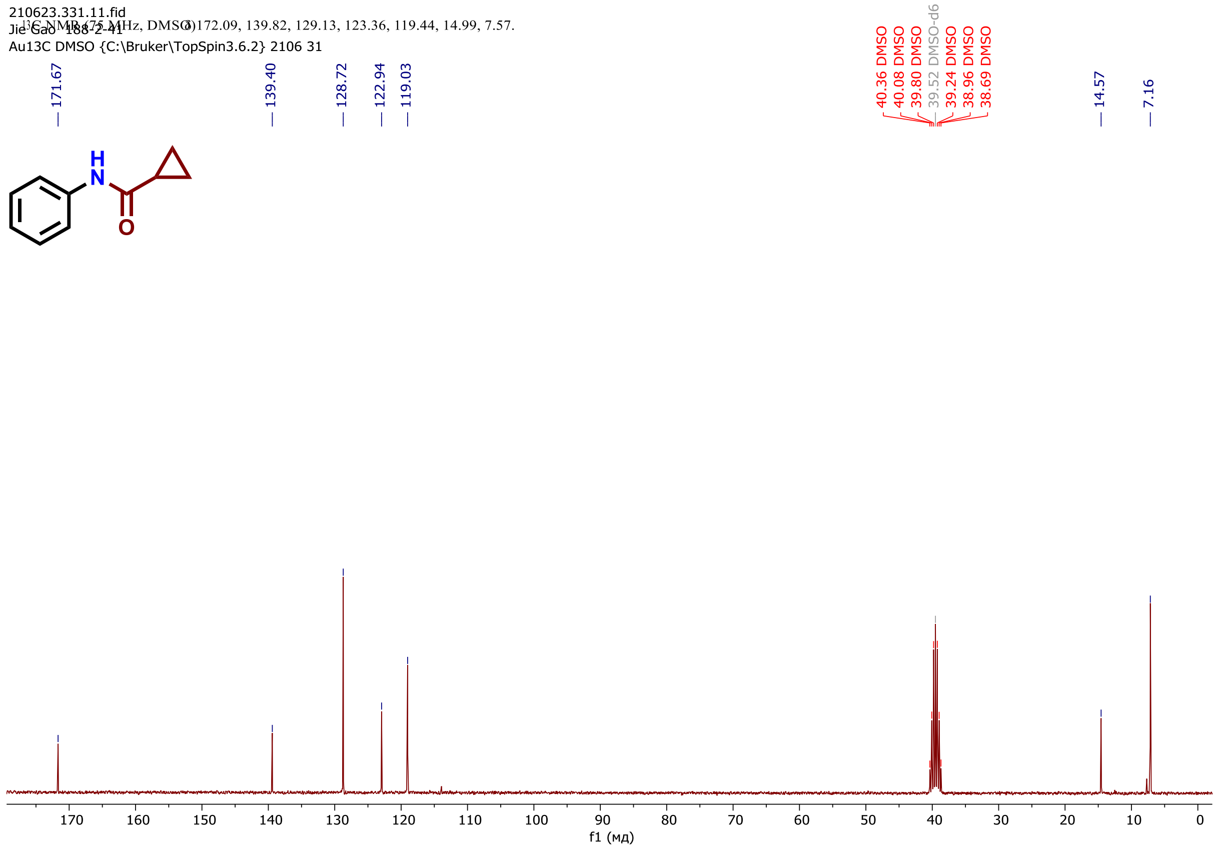

Supplementary Figure 63. NMR spectra of N-phenylcyclopropanecarboxamide.

N-phenylcyclobutanecarboxamide (42)

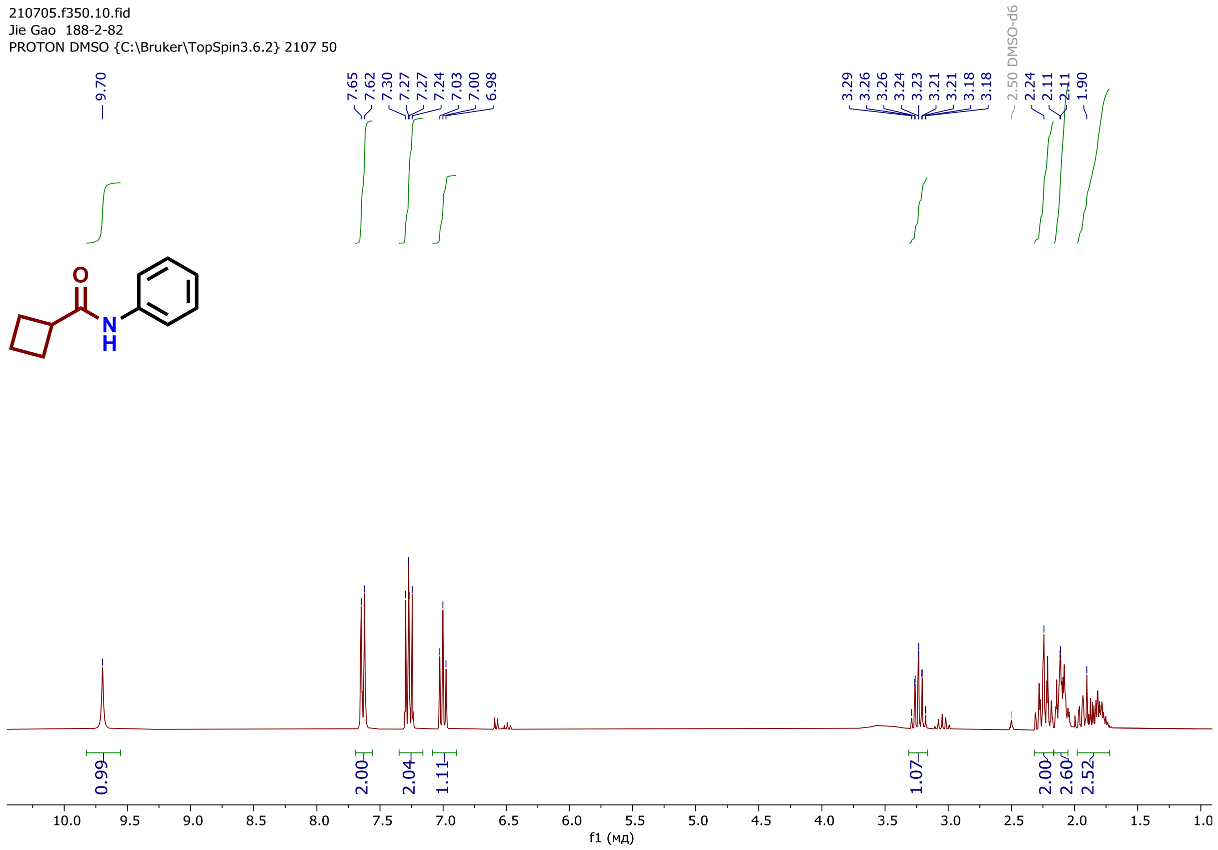

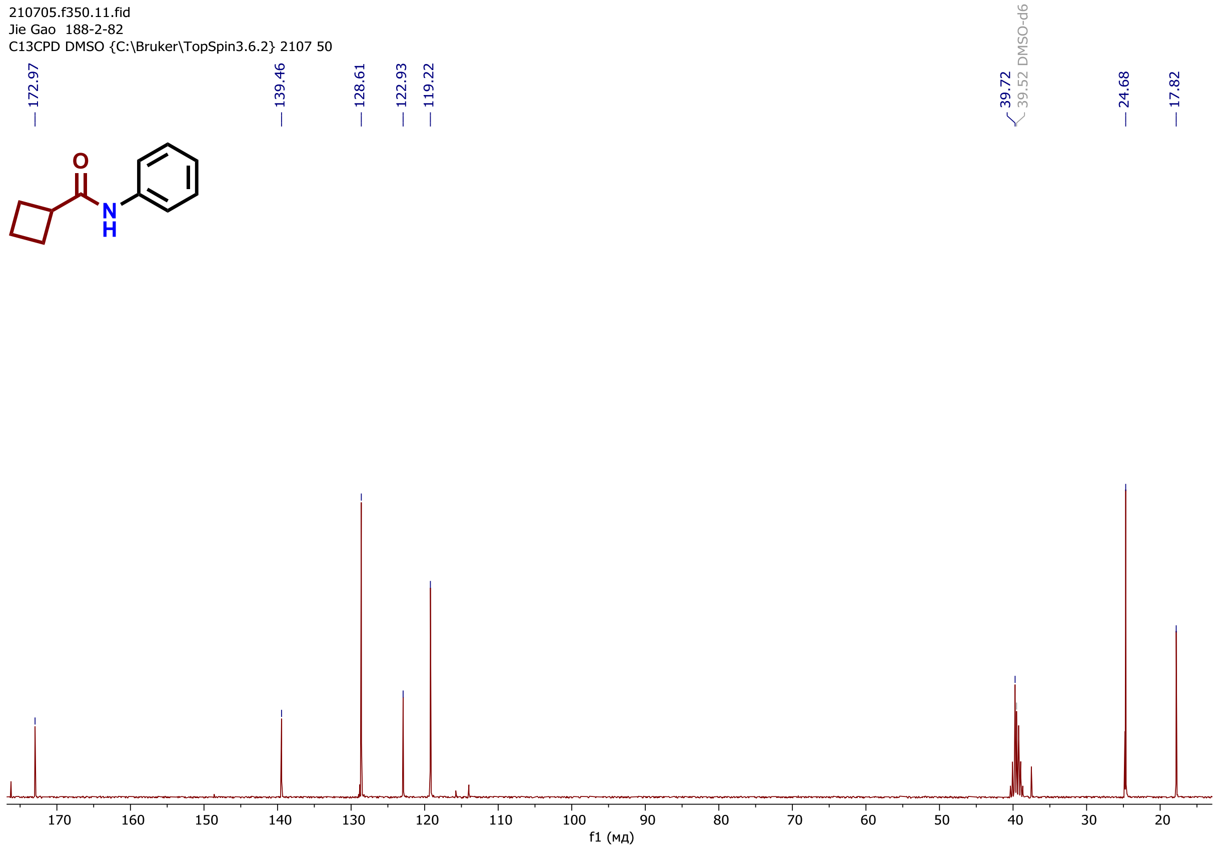

Supplementary Figure 64. NMR spectra of N-phenylcyclobutanecarboxamide.

2,2,2-trifluoro-N-phenylacetamide (43)

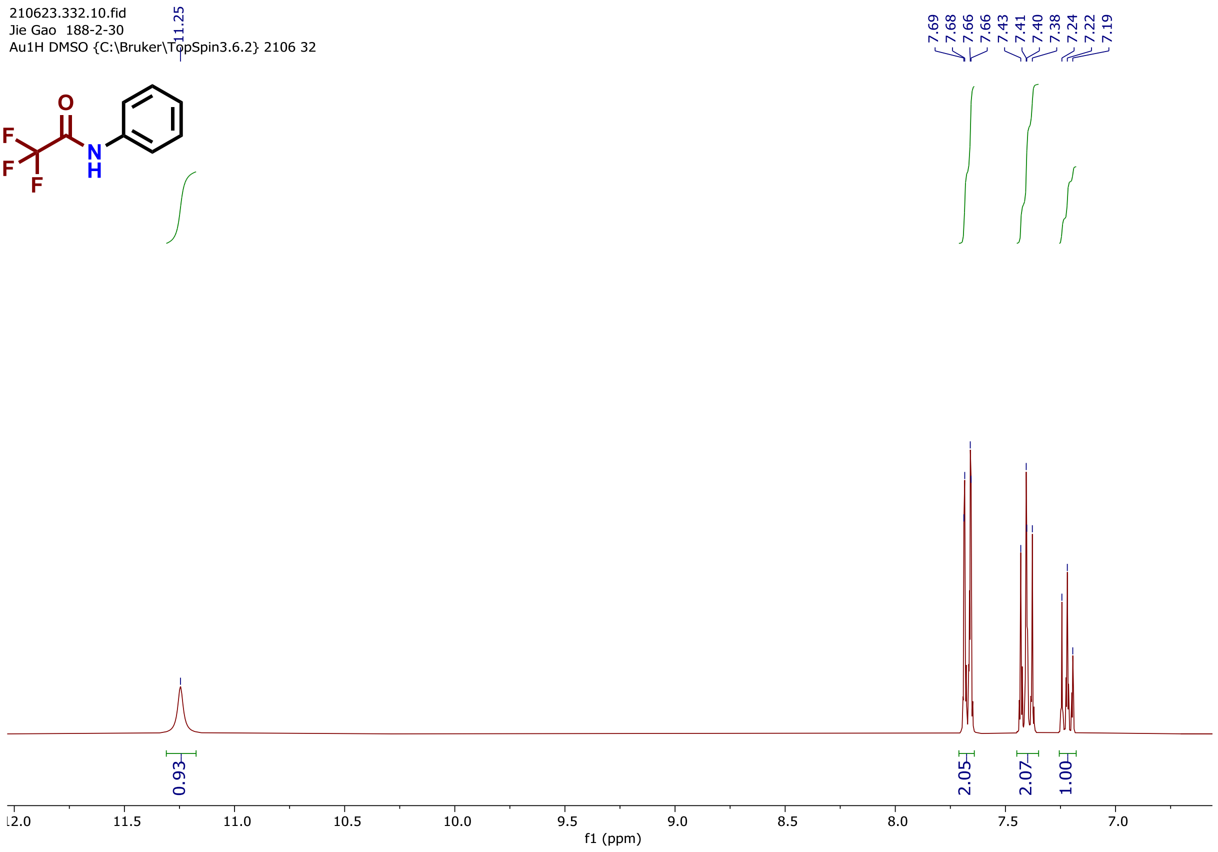

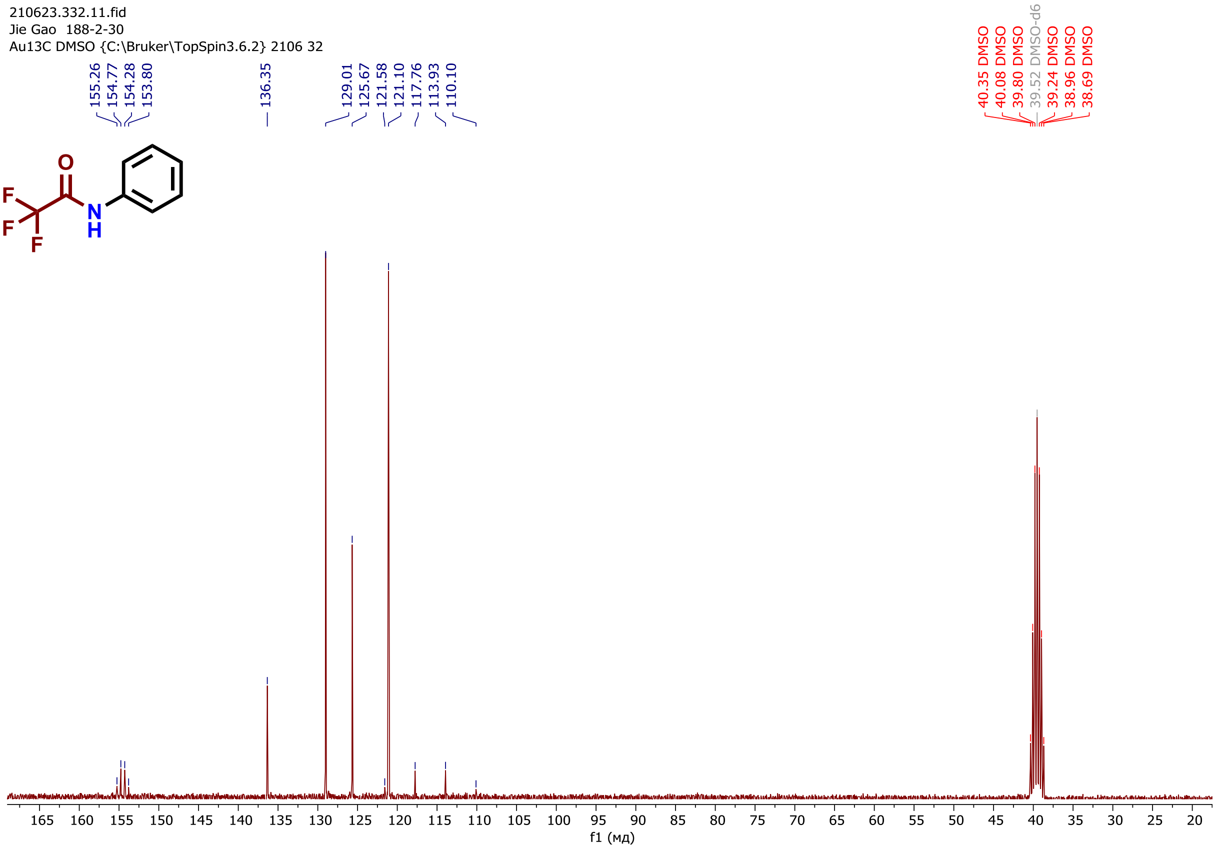

Supplementary Figure 65. NMR spectra of 2,2,2-trifluoro-N-phenylacetamide.

2-cyano-N-phenylacetamide (44)

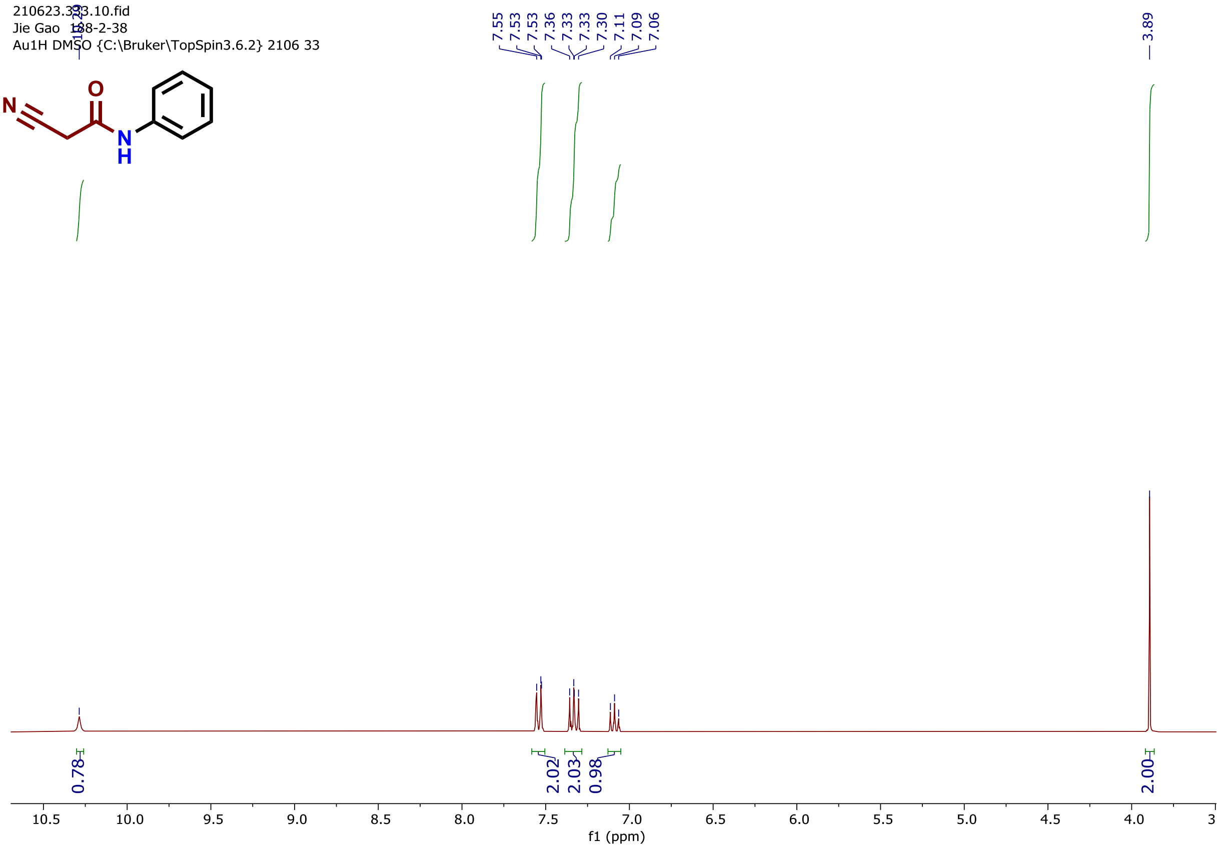

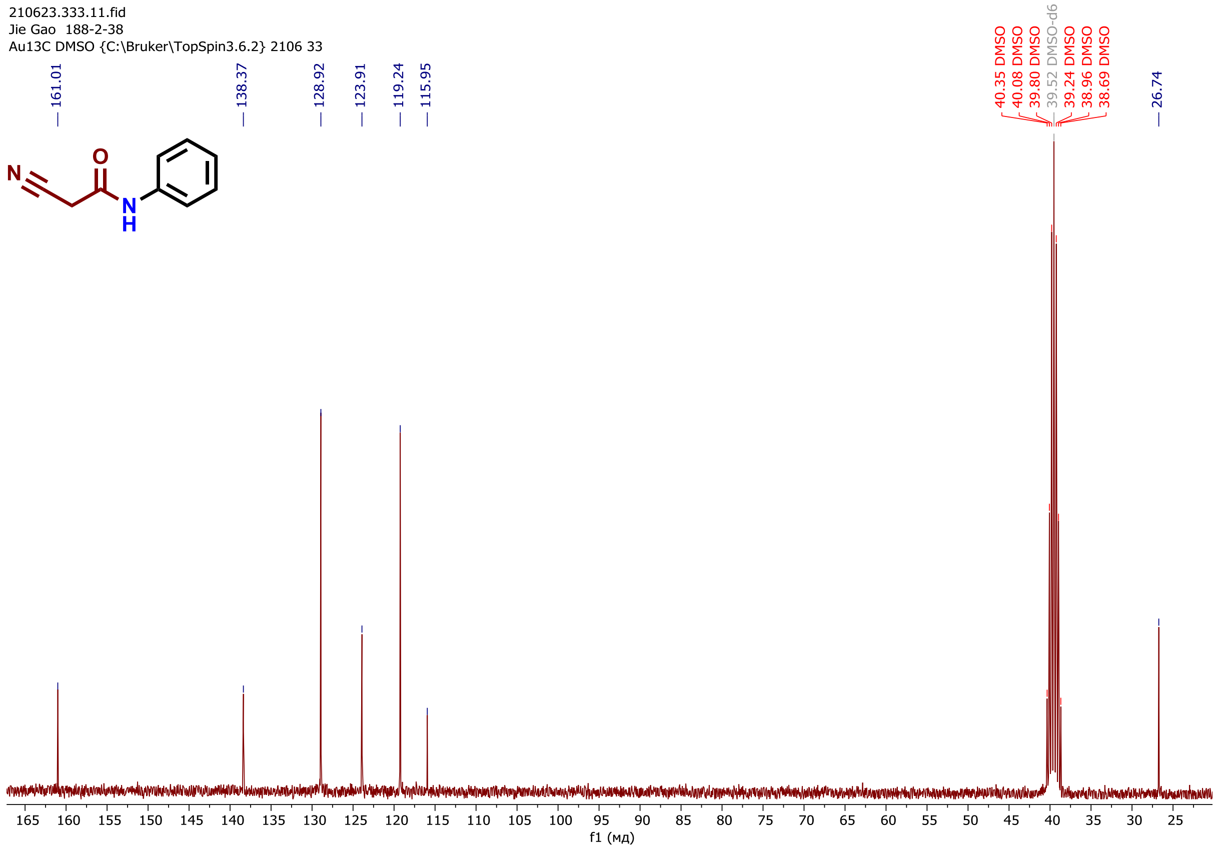

Supplementary Figure 66. NMR spectra of 2-cyano-N-phenylacetamide.

ethyl 2-methyl-3-oxo-3-(phenylamino)propanoate (45)

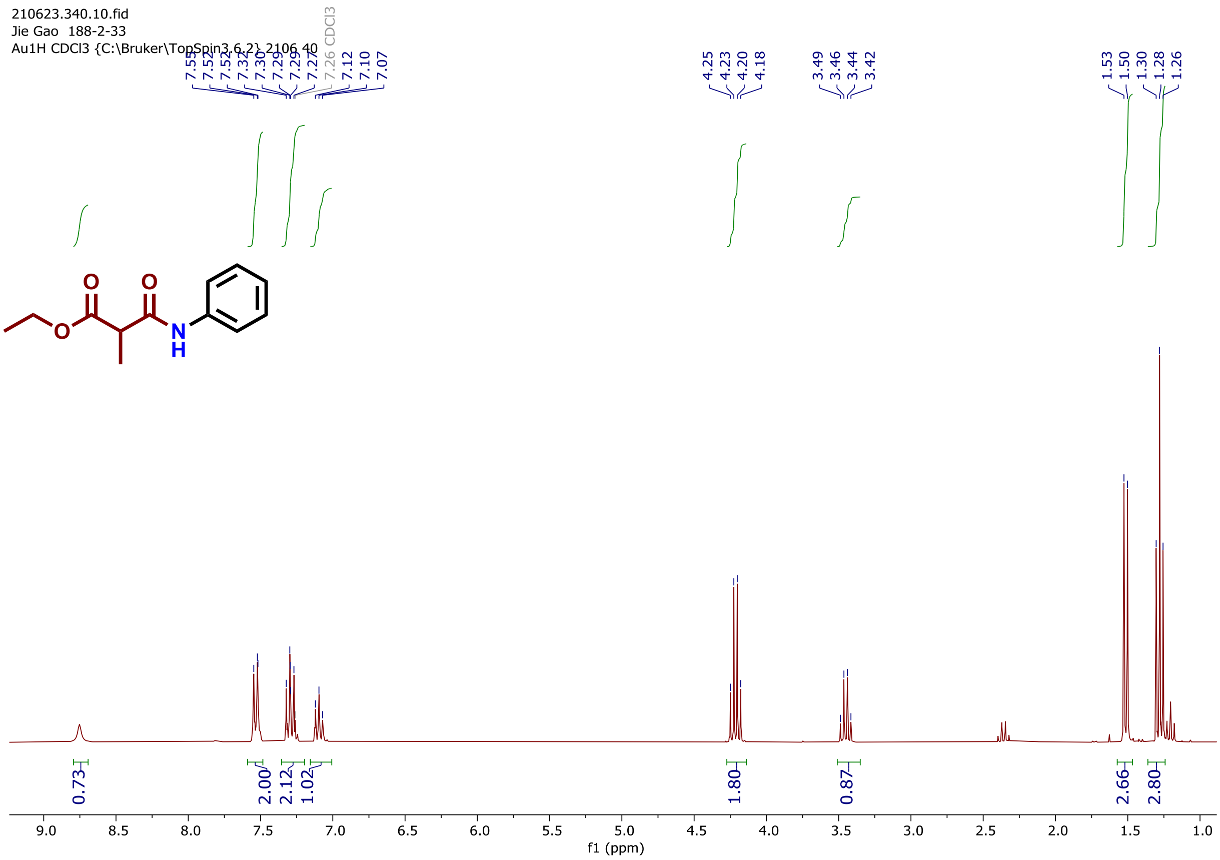

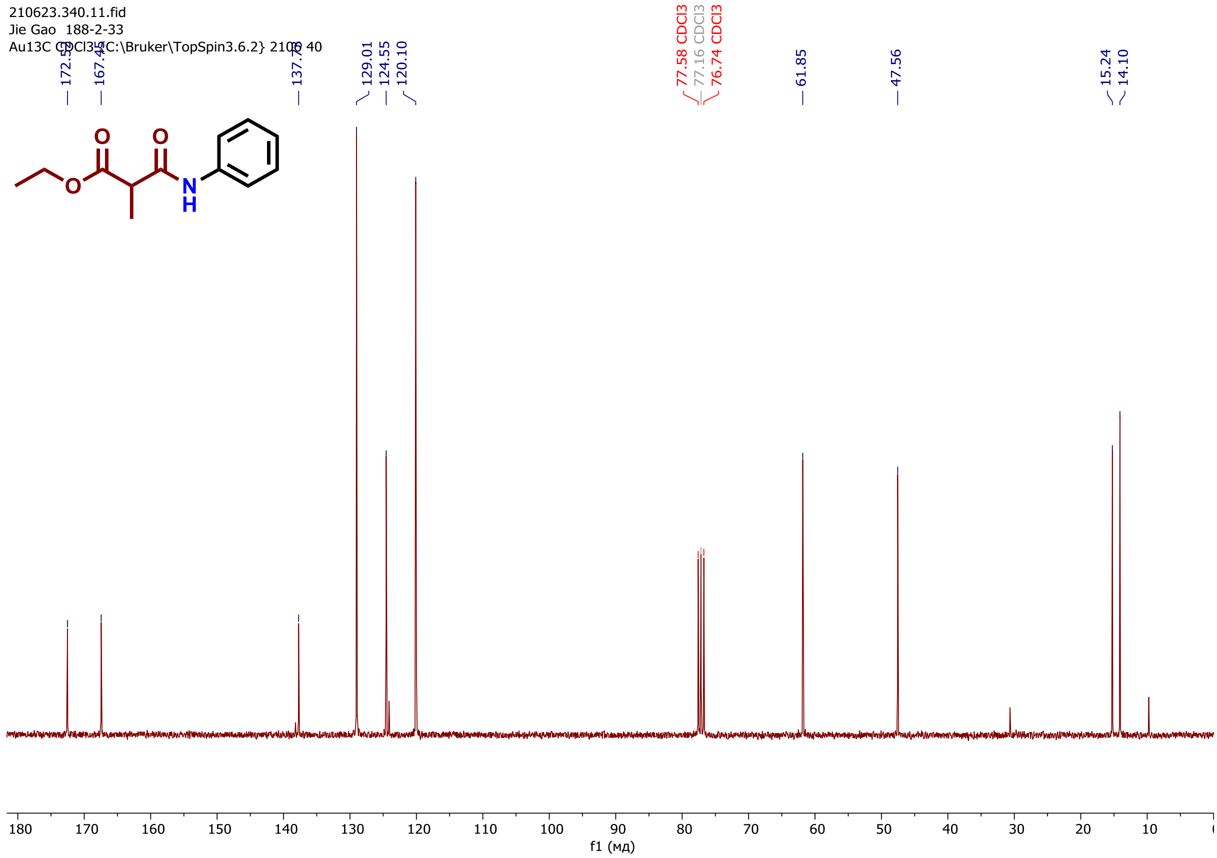

Supplementary Figure 67. NMR spectra of ethyl 2-methyl-3-oxo-3-(phenylamino)propanoate.

N,2-diphenylacetamide (46)

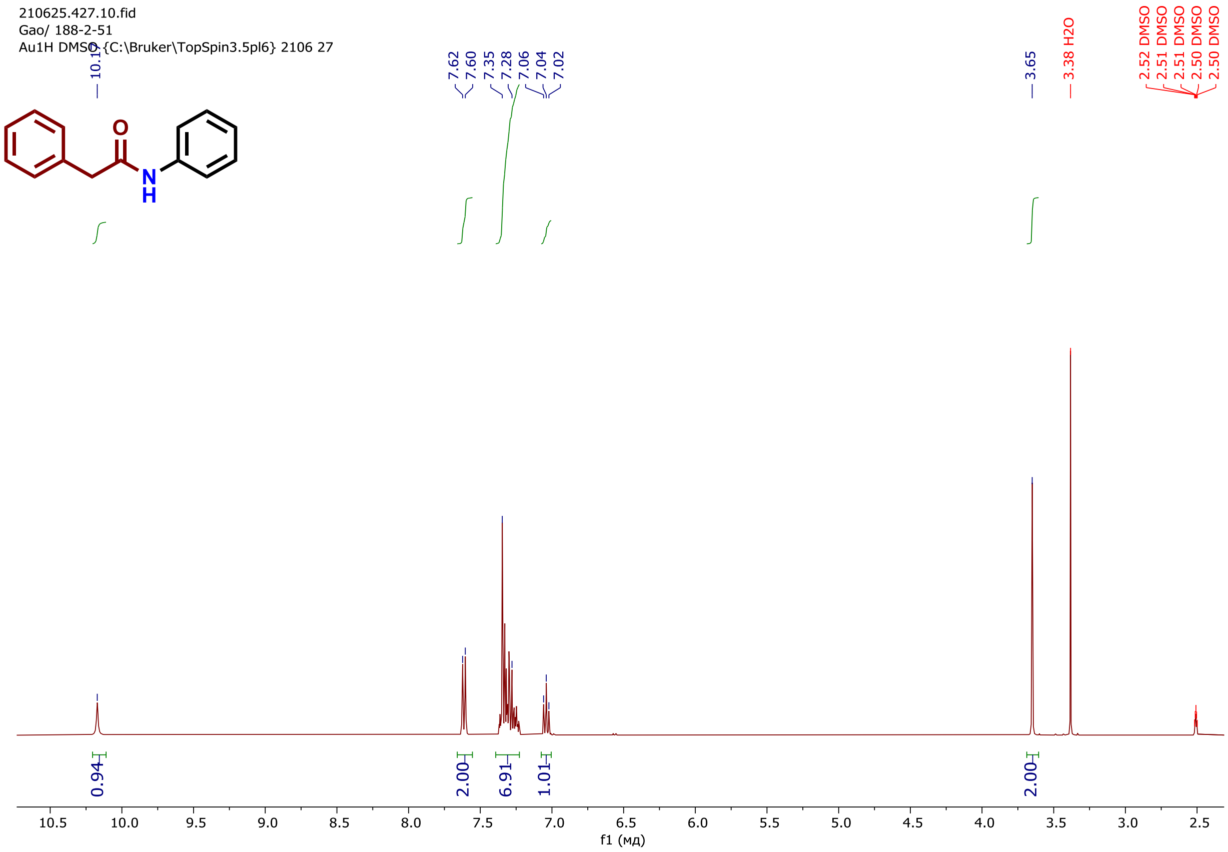

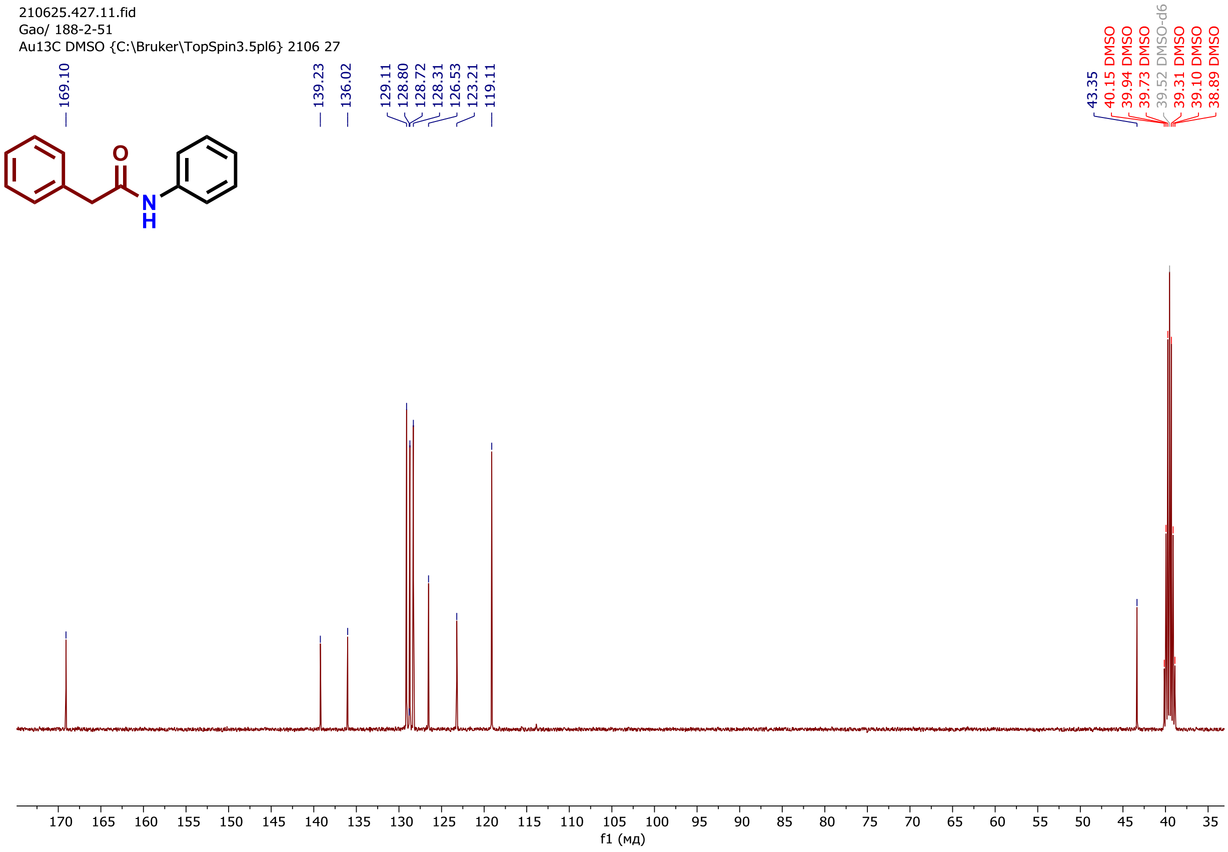

Supplementary Figure 68. NMR spectra of N,2-diphenylacetamide.

2-cyano-N,2-diphenylacetamide (47)

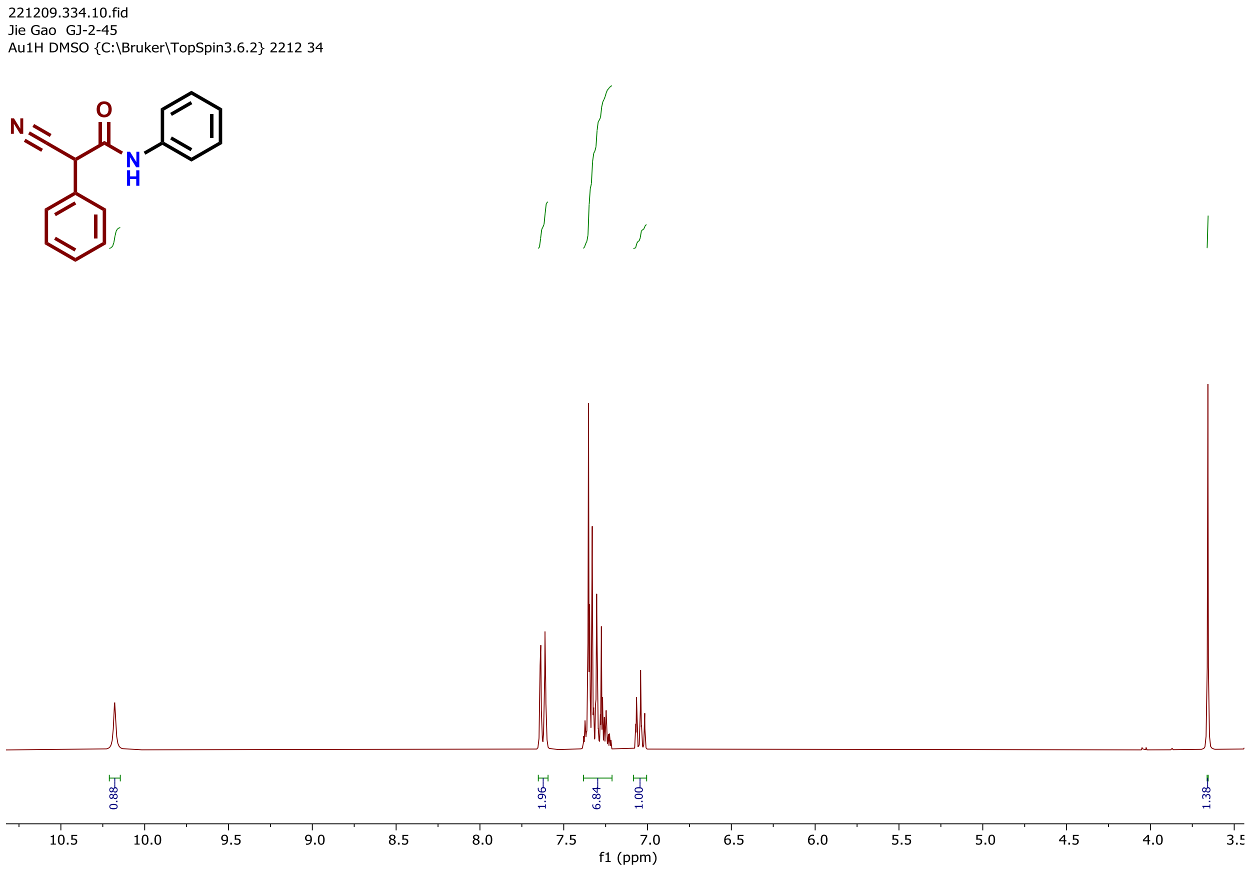

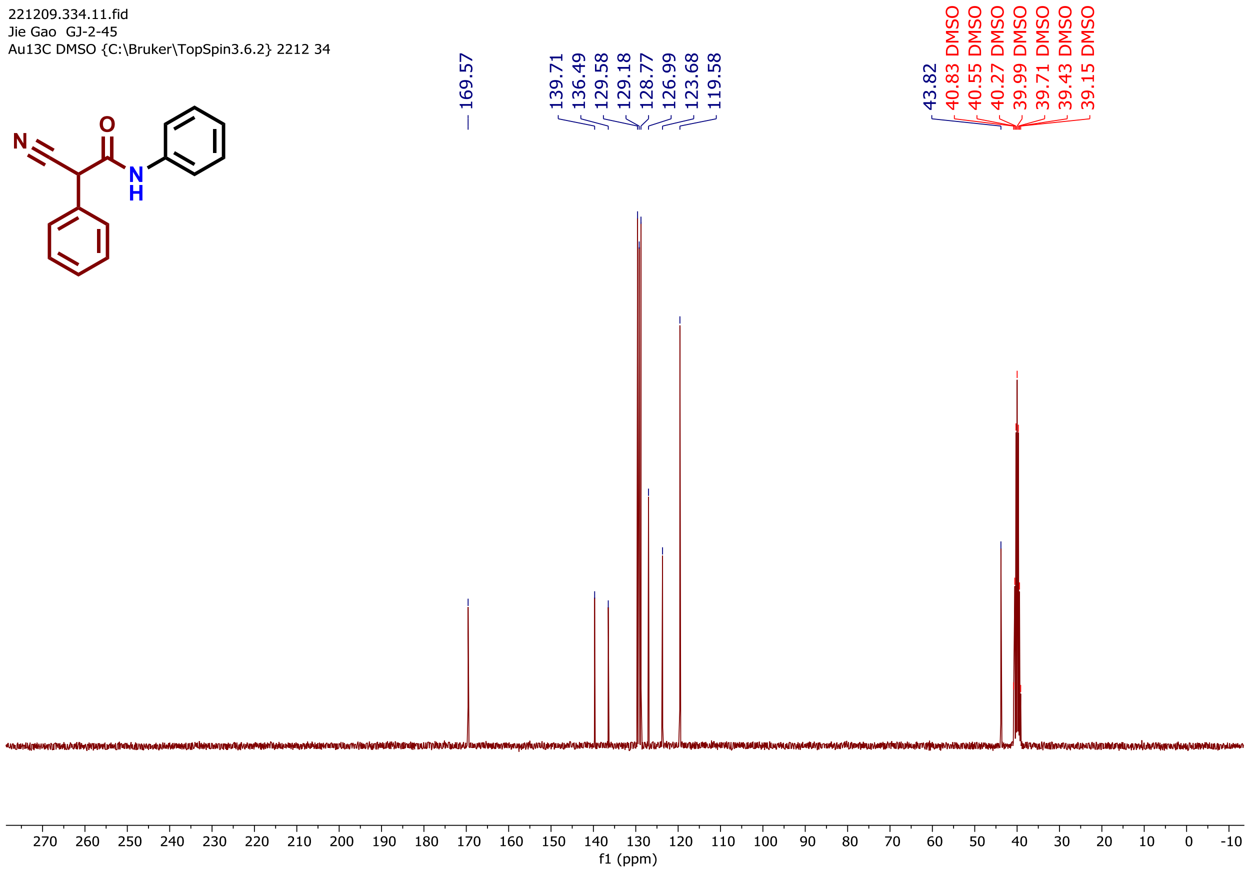

Supplementary Figure 69. NMR spectra of 2-cyano-N,2-diphenylacetamide.

dimethyl 3,3'-(1,4-phenylenebis(azanediyl))bis(3-oxopropanoate) (48)

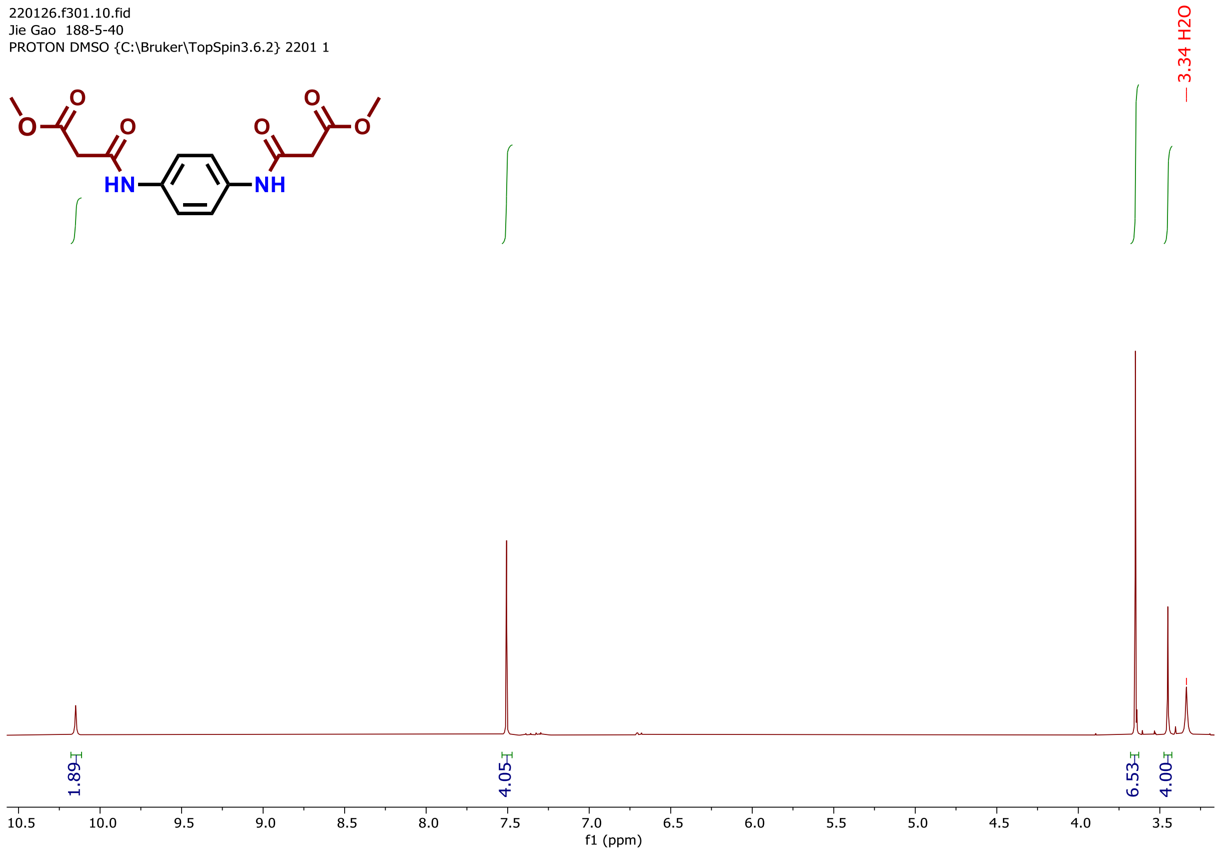

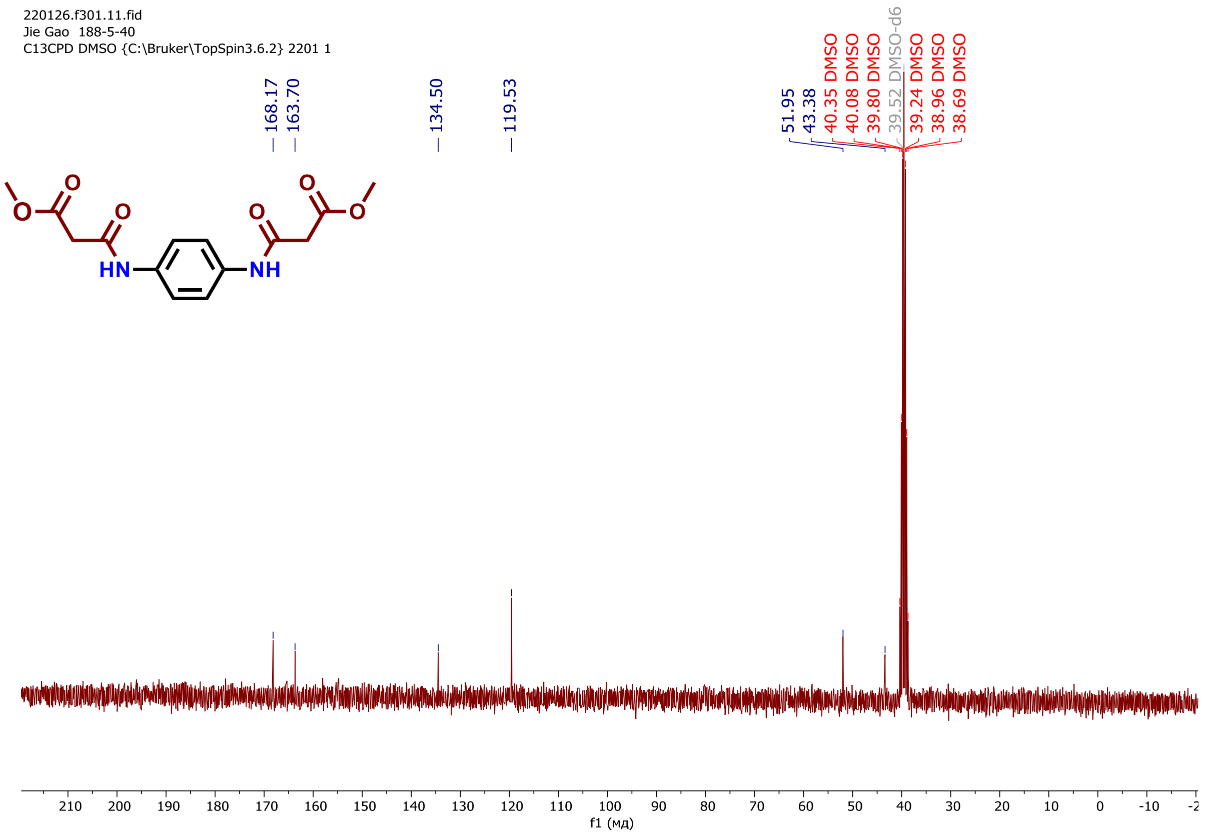

Supplementary Figure 70. NMR spectra of dimethyl 3,3'-(1,4-phenylenebis(azanediyl))bis(3-oxopropanoate)

N-phenylbenzamide (49)

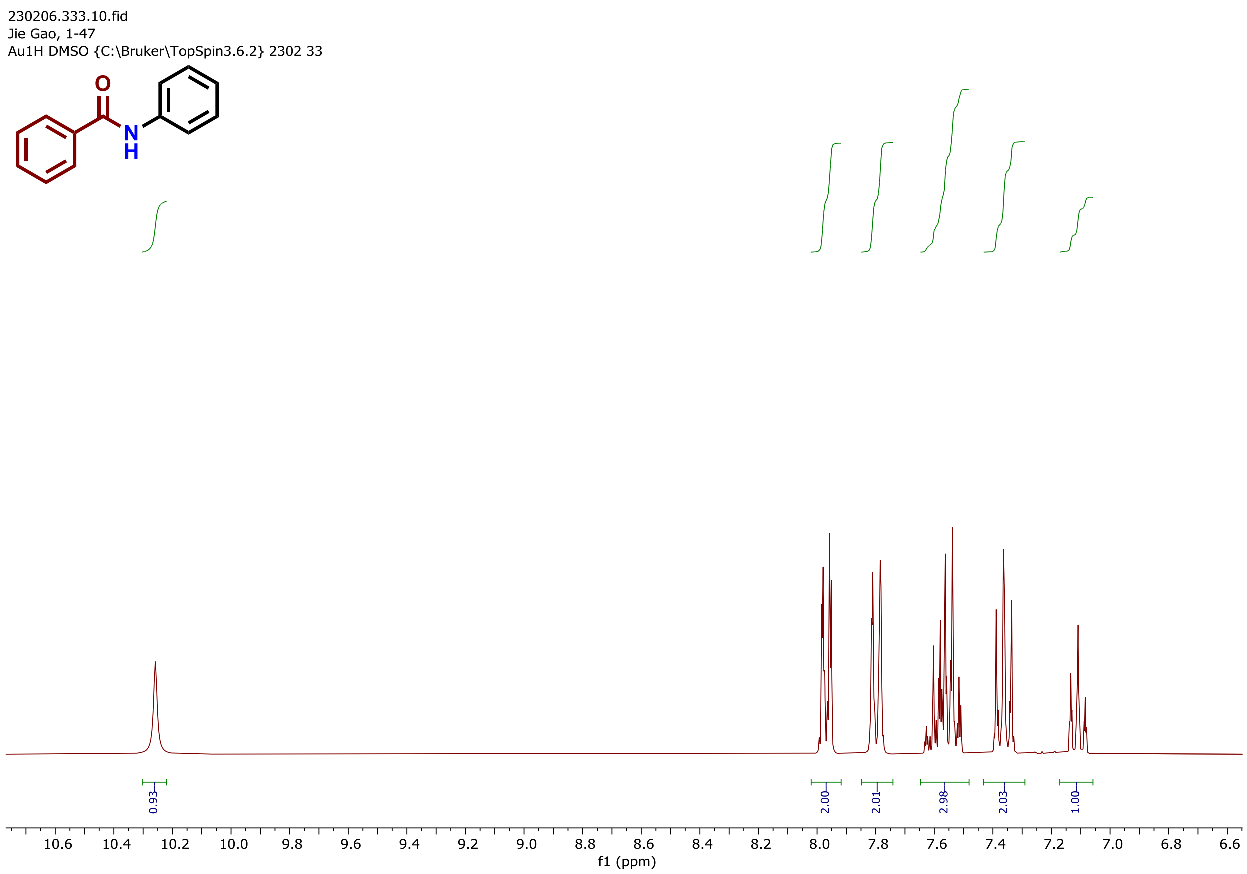

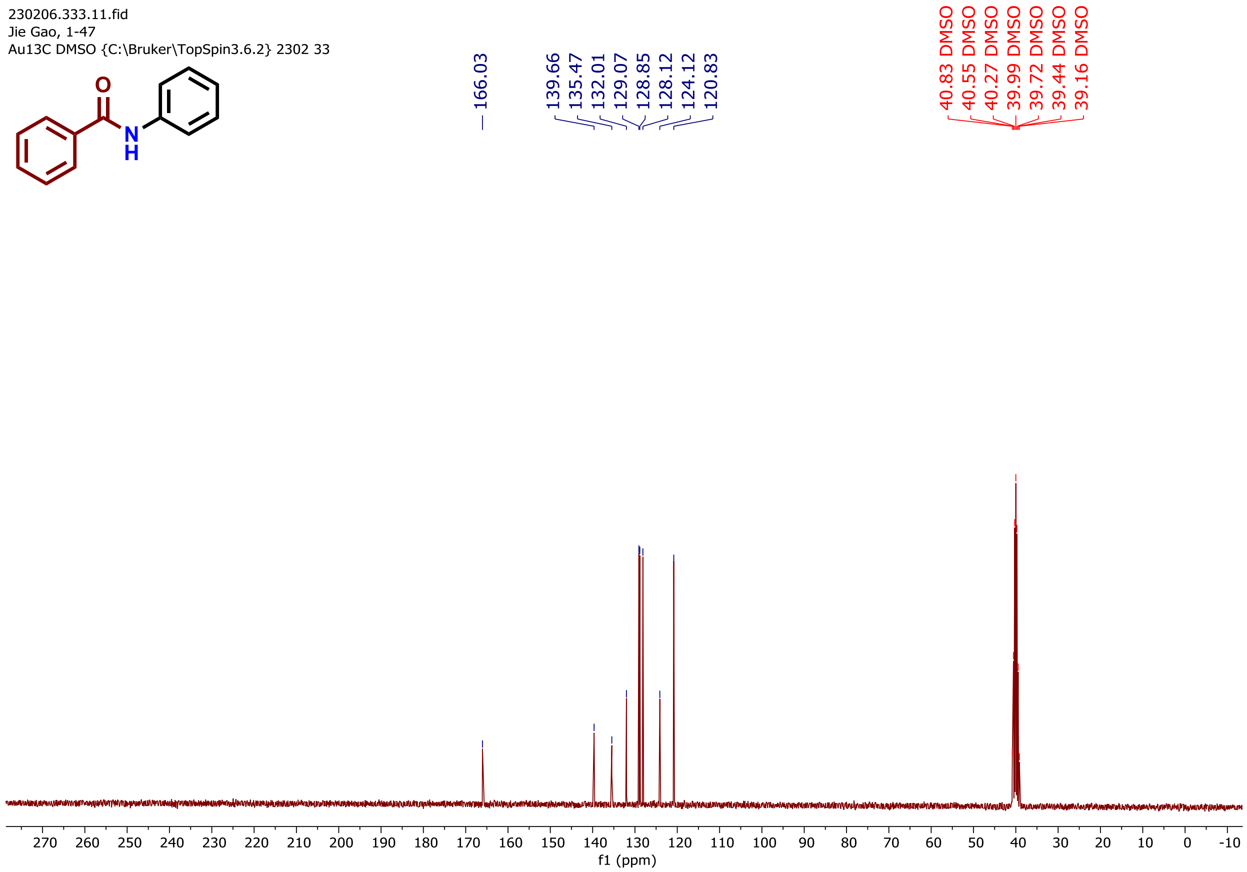

Supplementary Figure 71. NMR spectra of N-phenylbenzamide.

N-(4-cyanophenyl)benzamide (50)

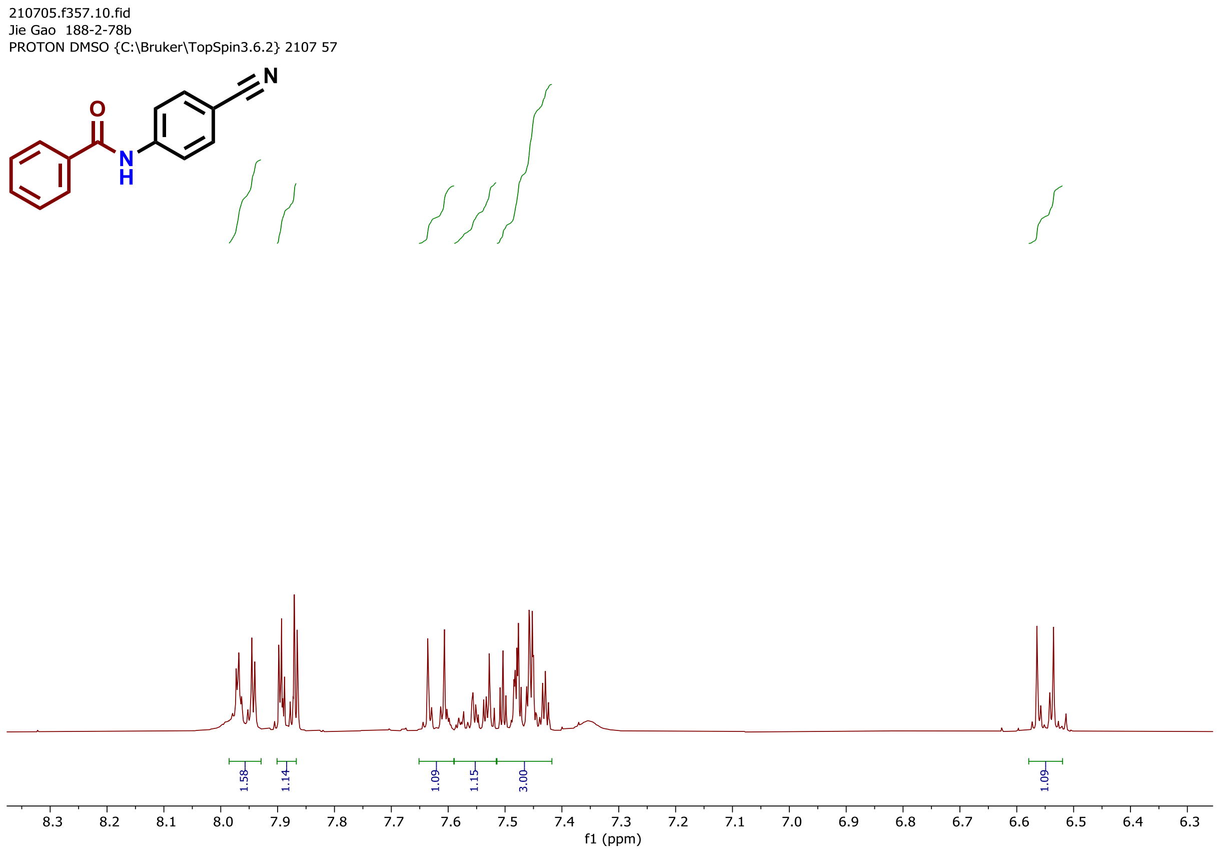

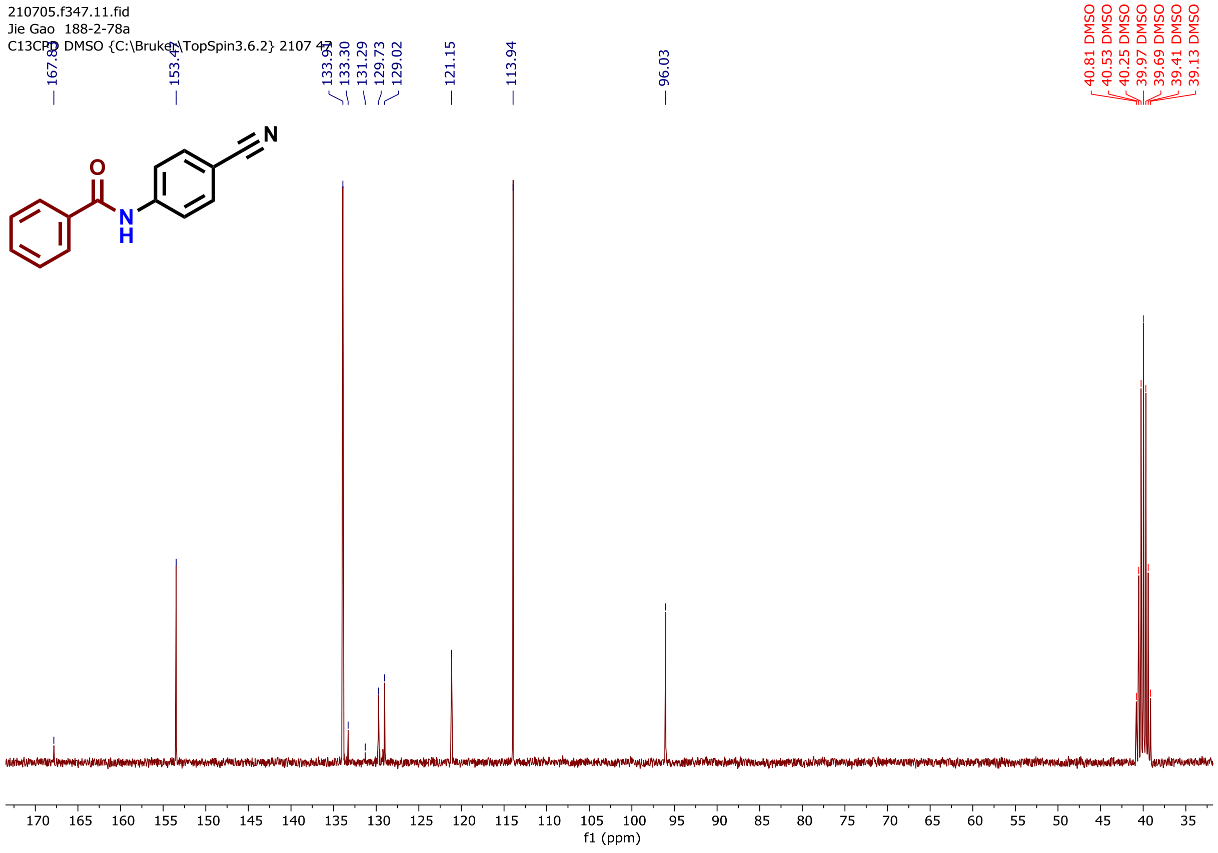

Supplementary Figure 72. NMR spectra of N-(4-cyanophenyl)benzamide.

4-fluoro-N-phenylbenzamide (51)

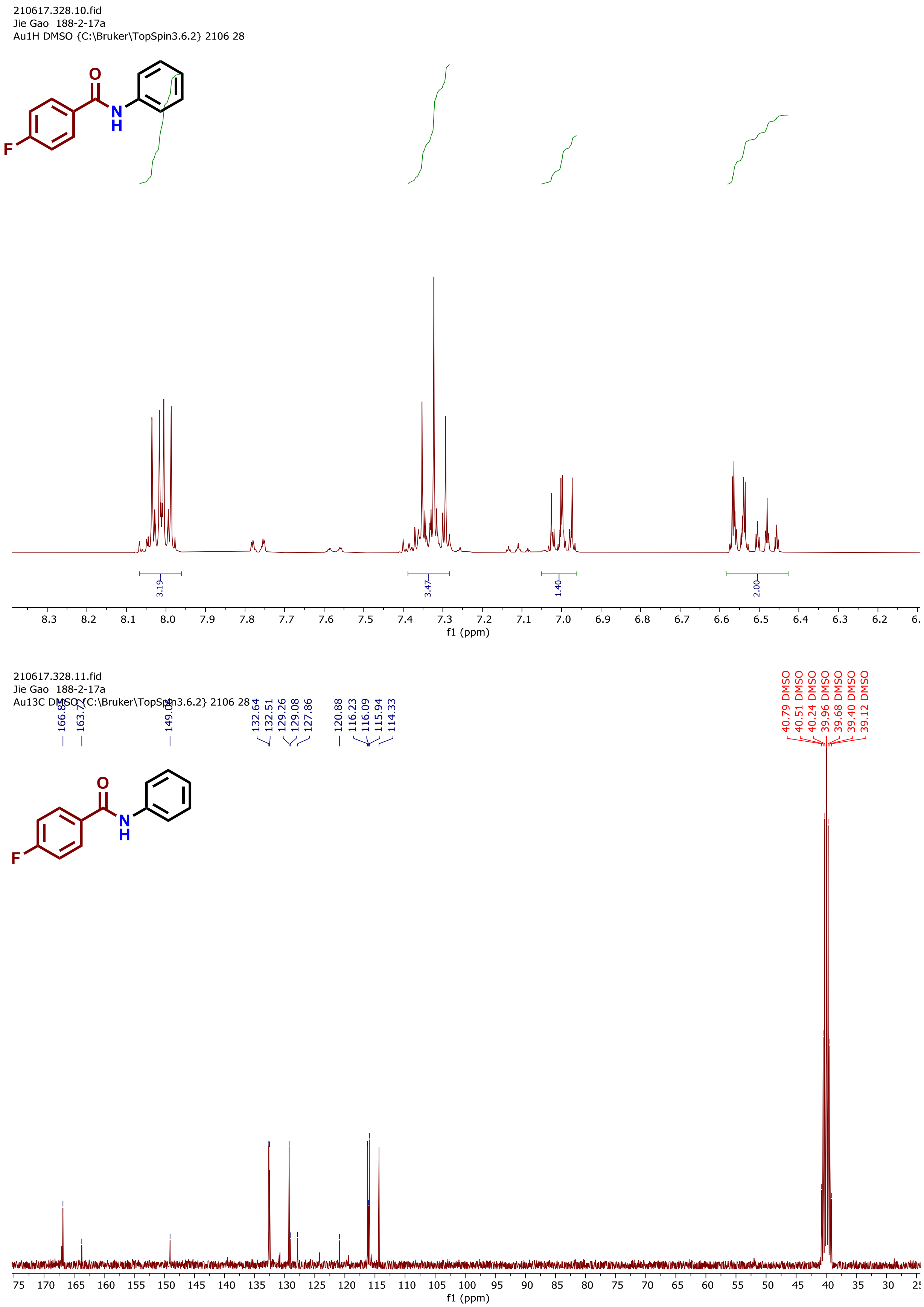

Supplementary Figure 73. NMR spectra of 4-fluoro-N-phenylbenzamide.

N-phenylfuran-3-carboxamide (52)

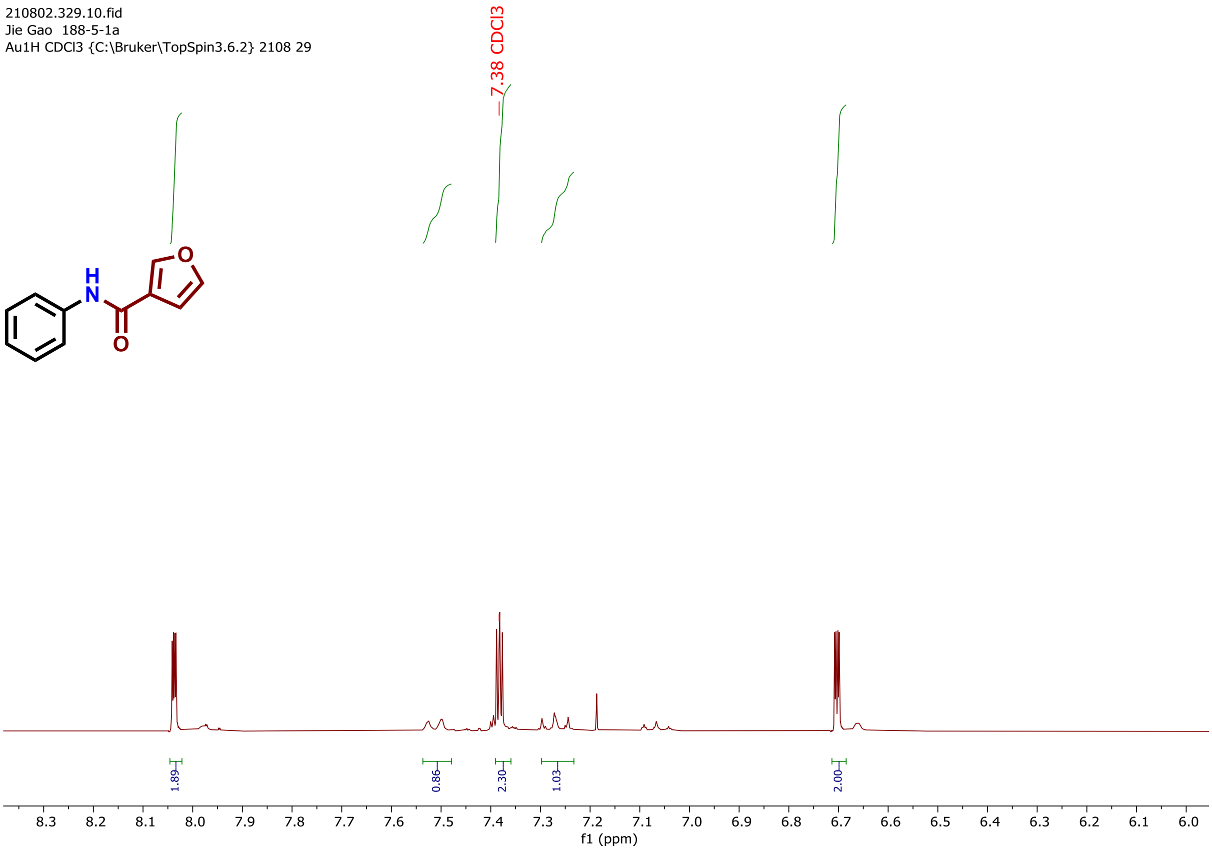

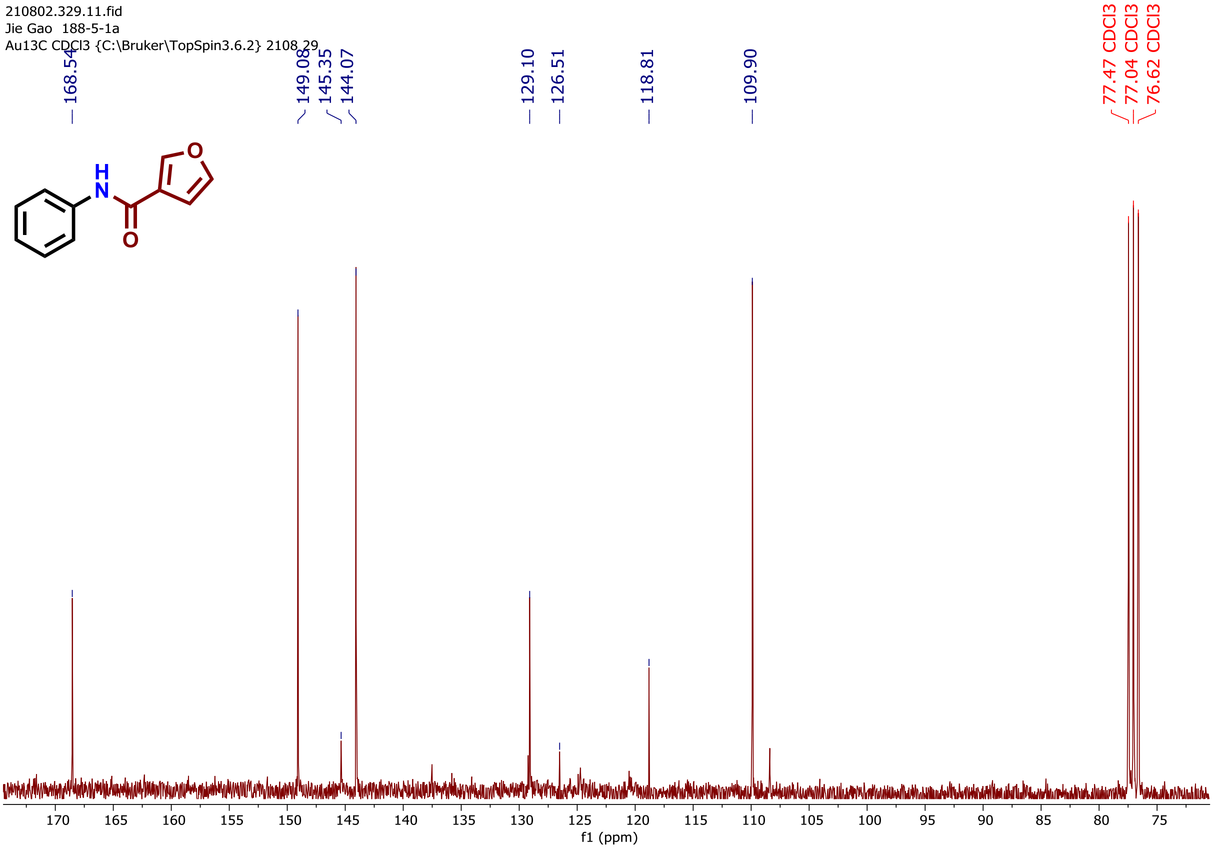

Supplementary Figure 74. NMR spectra of N-phenylfuran-3-carboxamide.

N-phenylthiophene-3-carboxamide (53)

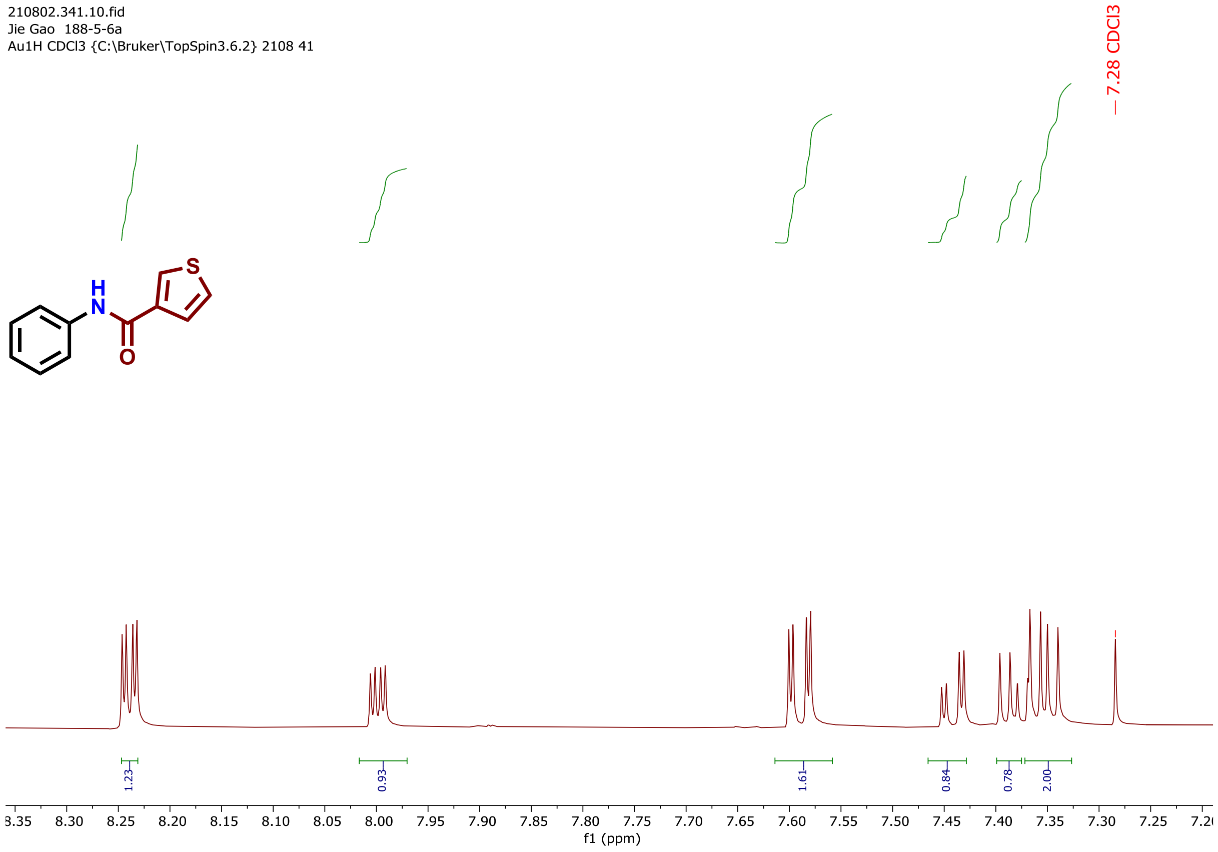

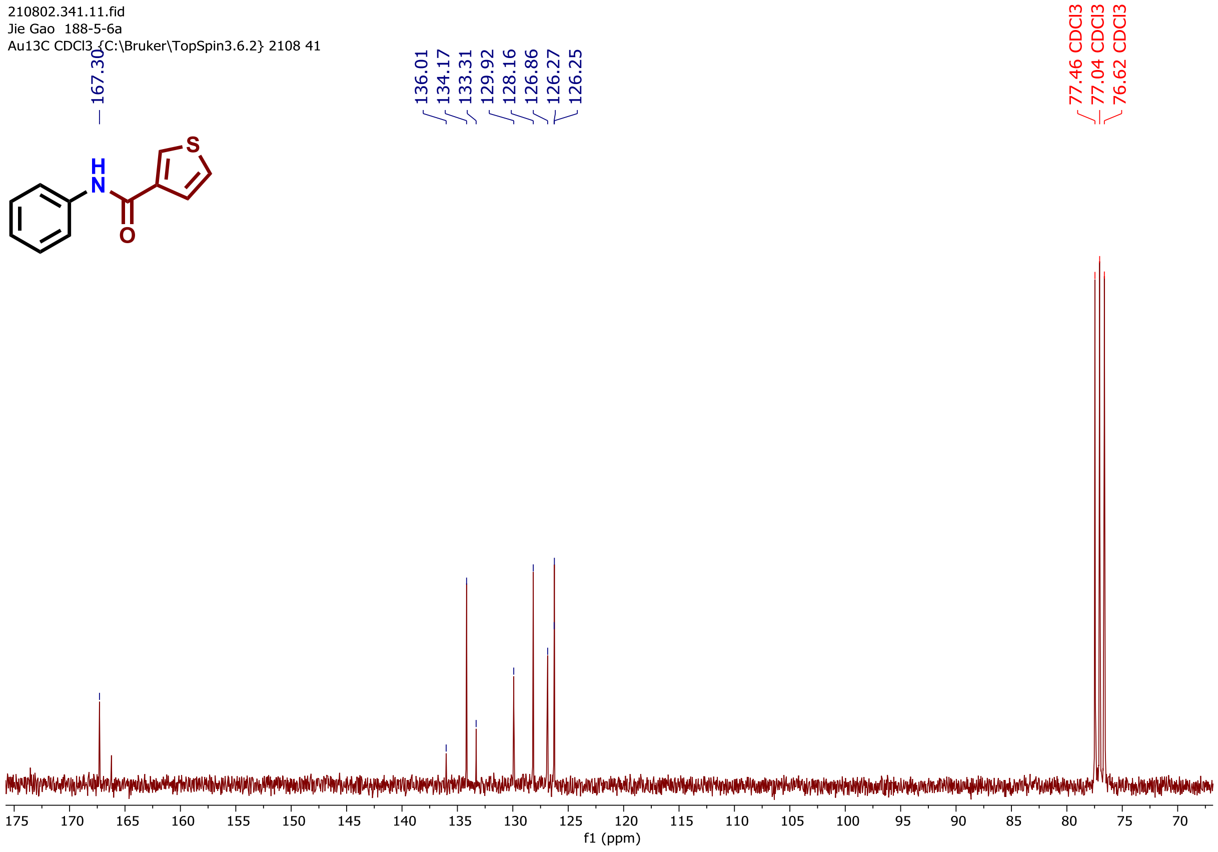

Supplementary Figure 75. NMR spectra of N-phenylthiophene-3-carboxamide.

methyl 5-(phenylcarbamoyl)furan-2-carboxylate (54)

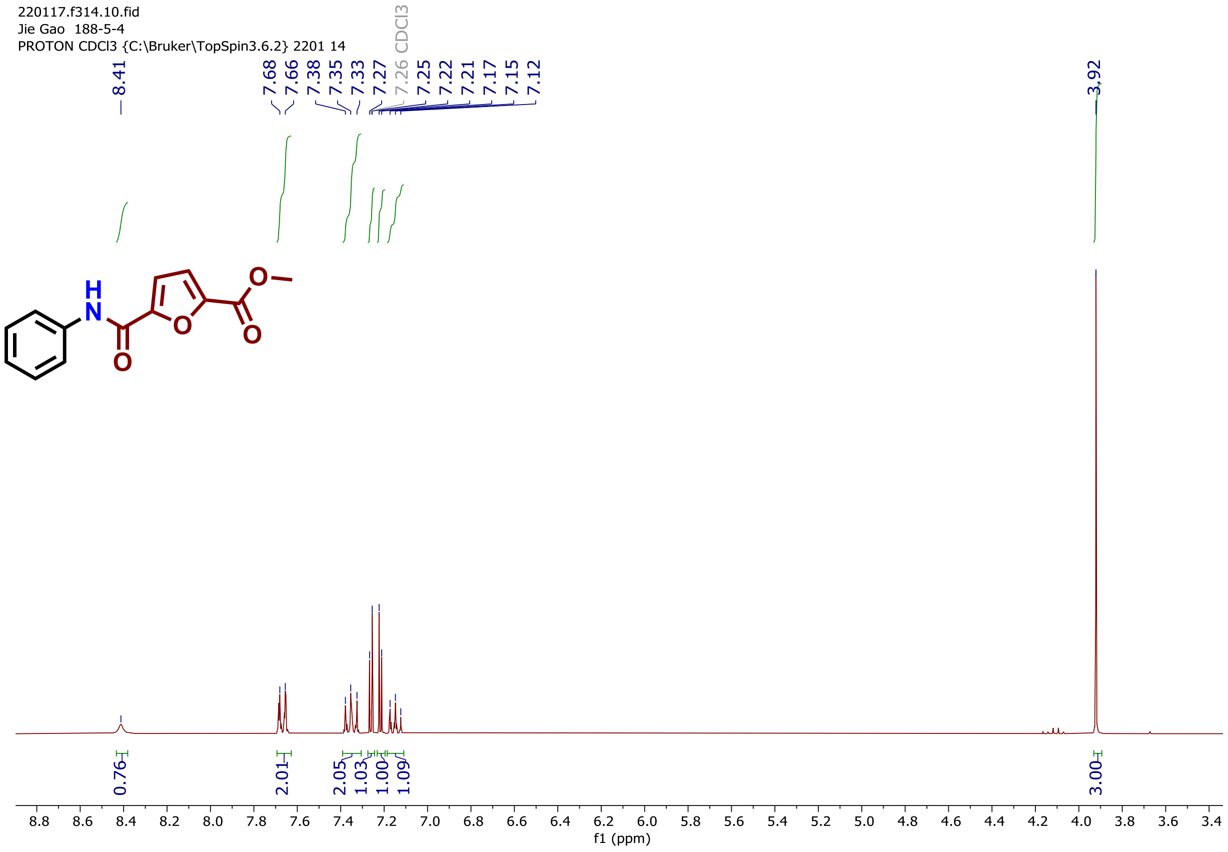

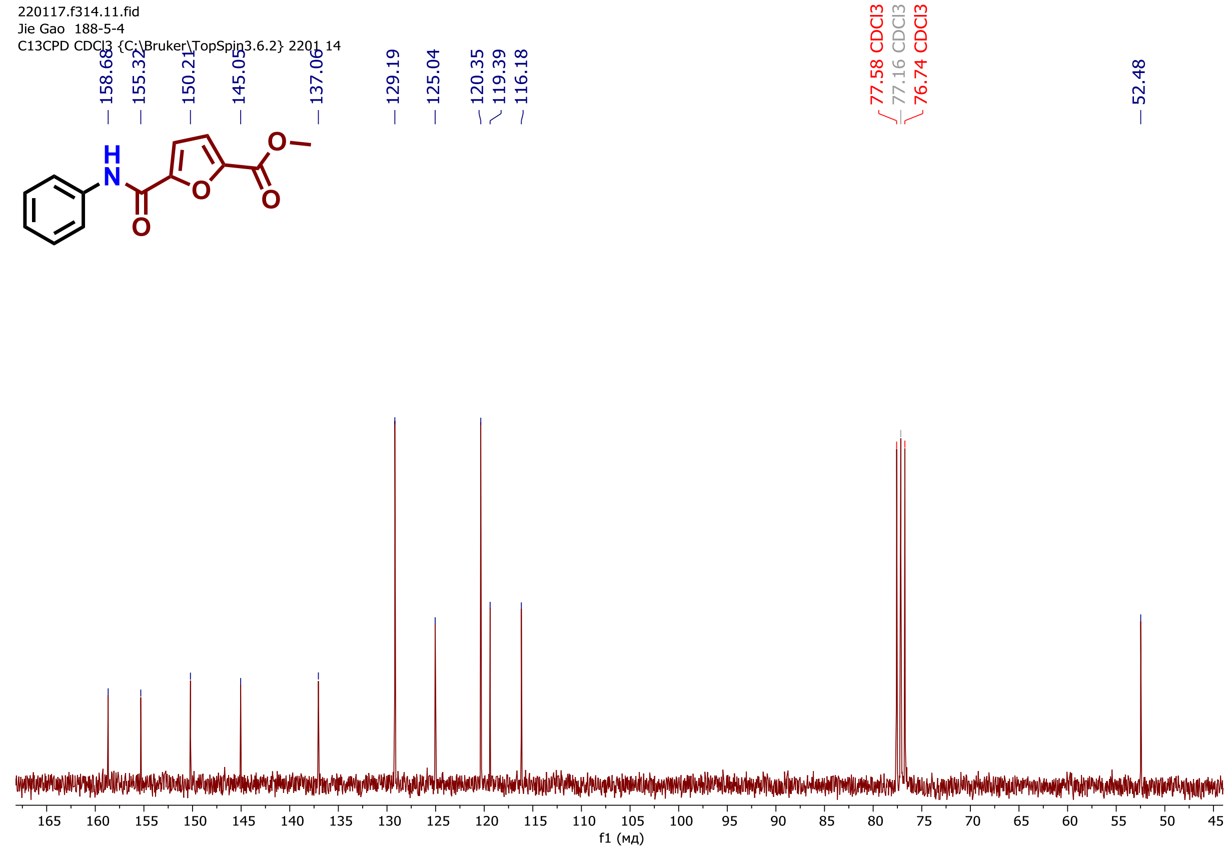

Supplementary Figure 76. NMR spectra of methyl 5-(phenylcarbamoyl)furan-2-carboxylate.

Acedoben (55)

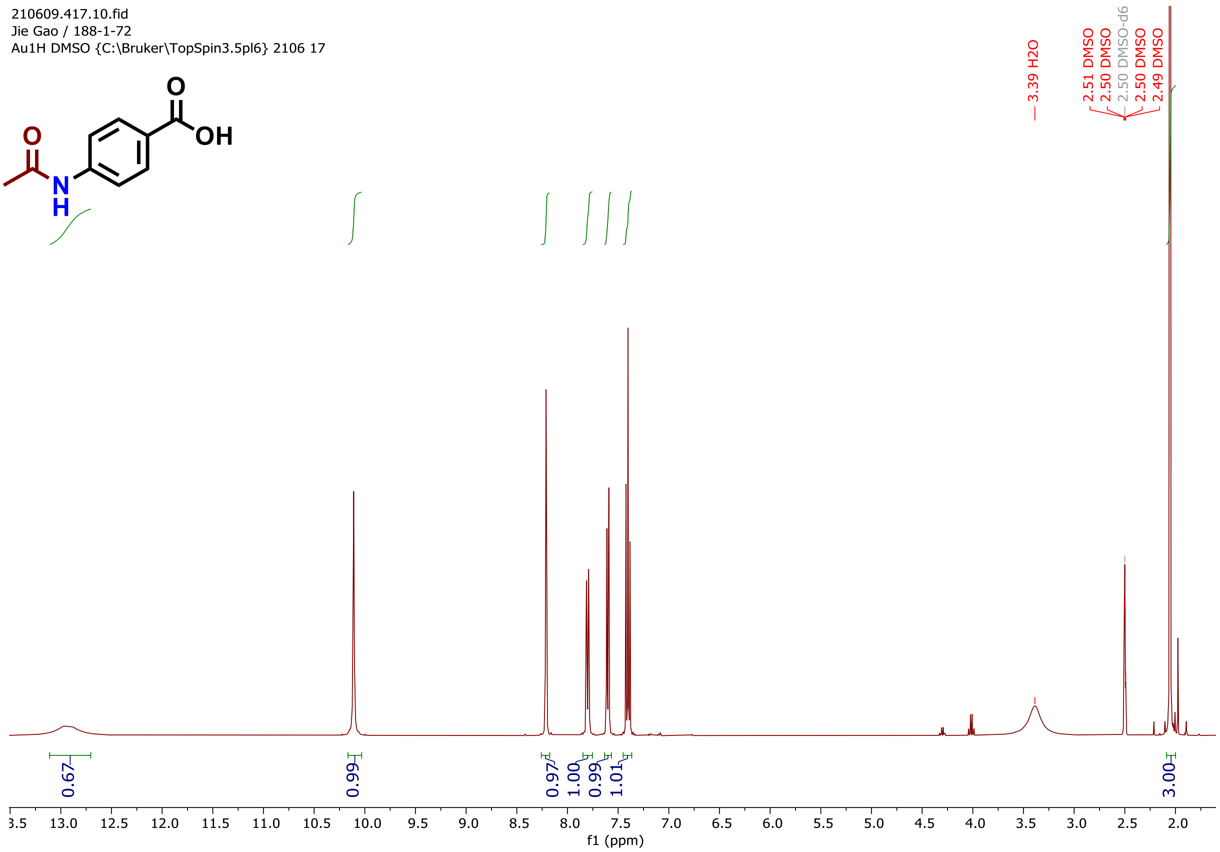

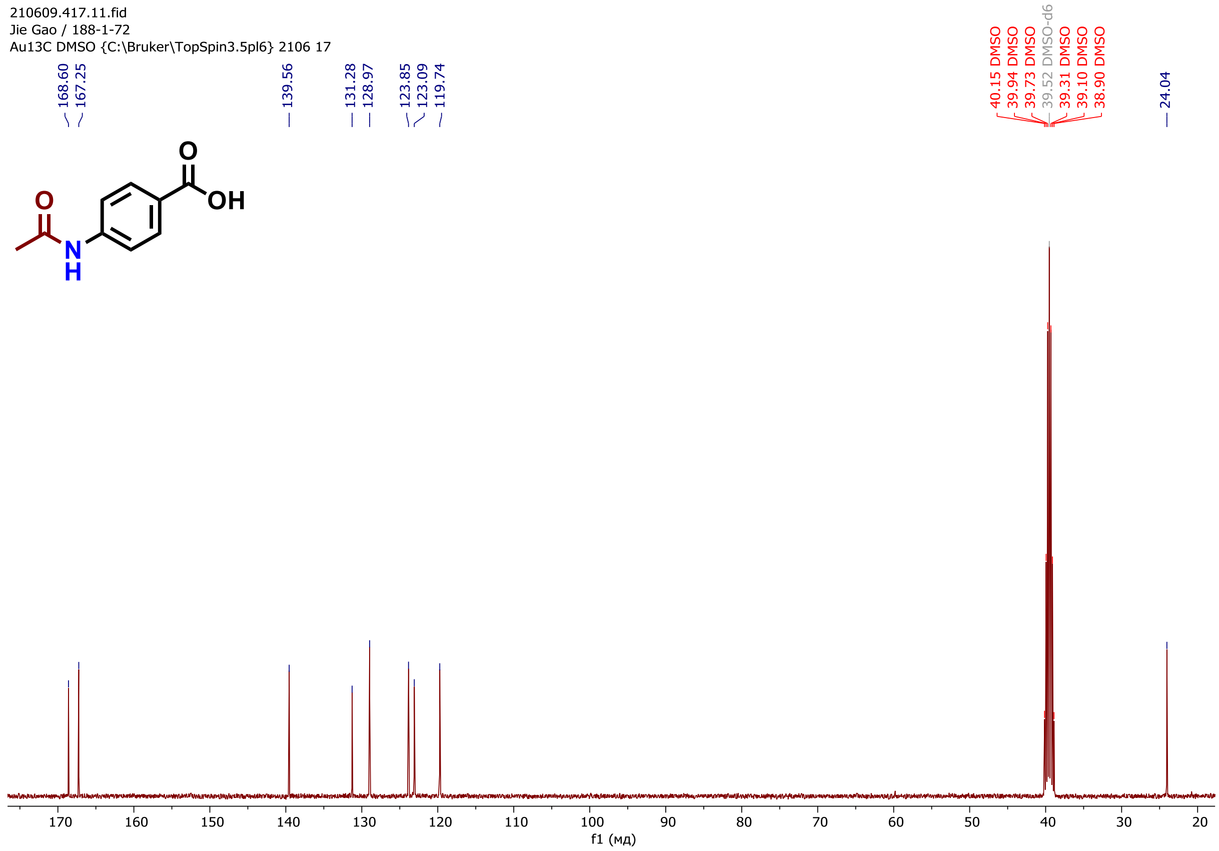

Supplementary Figure 77. NMR spectra of Acedoben.

Actarit (56)

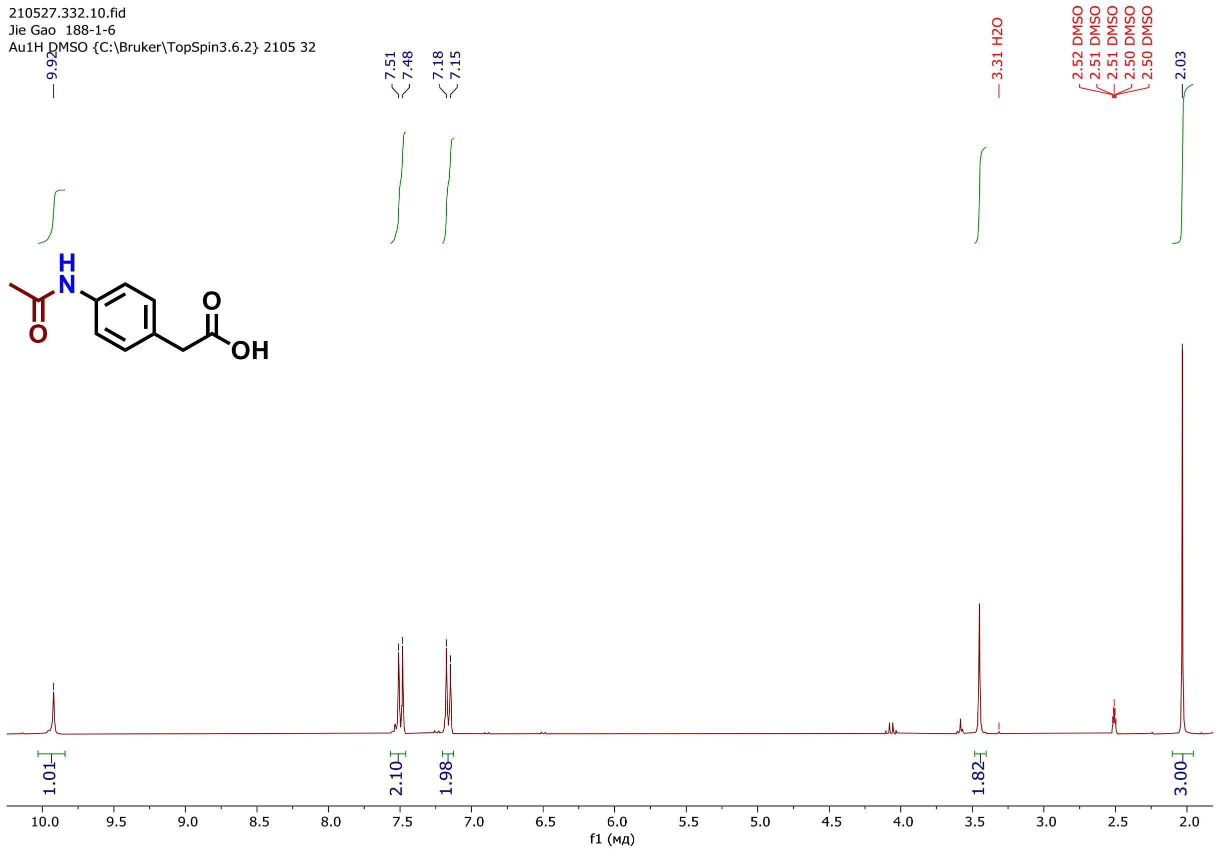

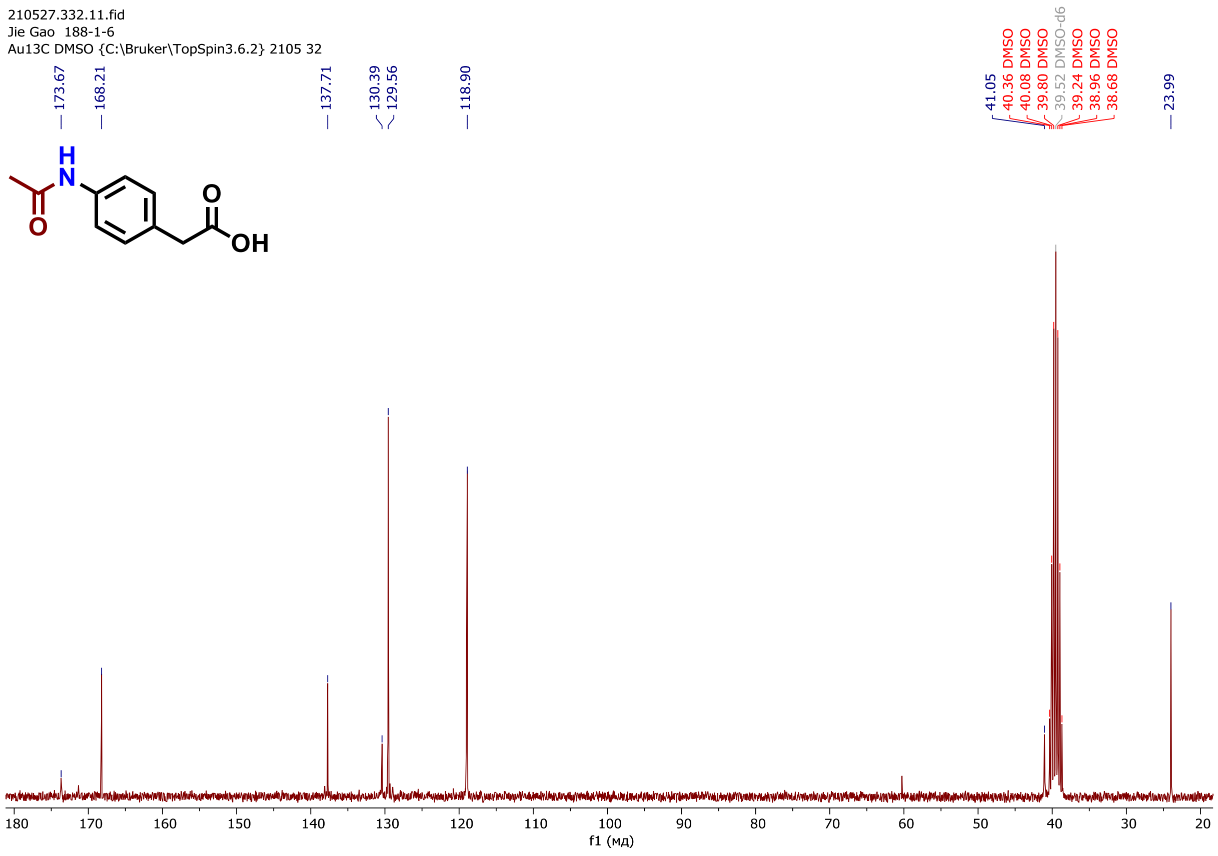

Supplementary Figure 78. NMR spectra of Actarit.

Phenacetin (57)

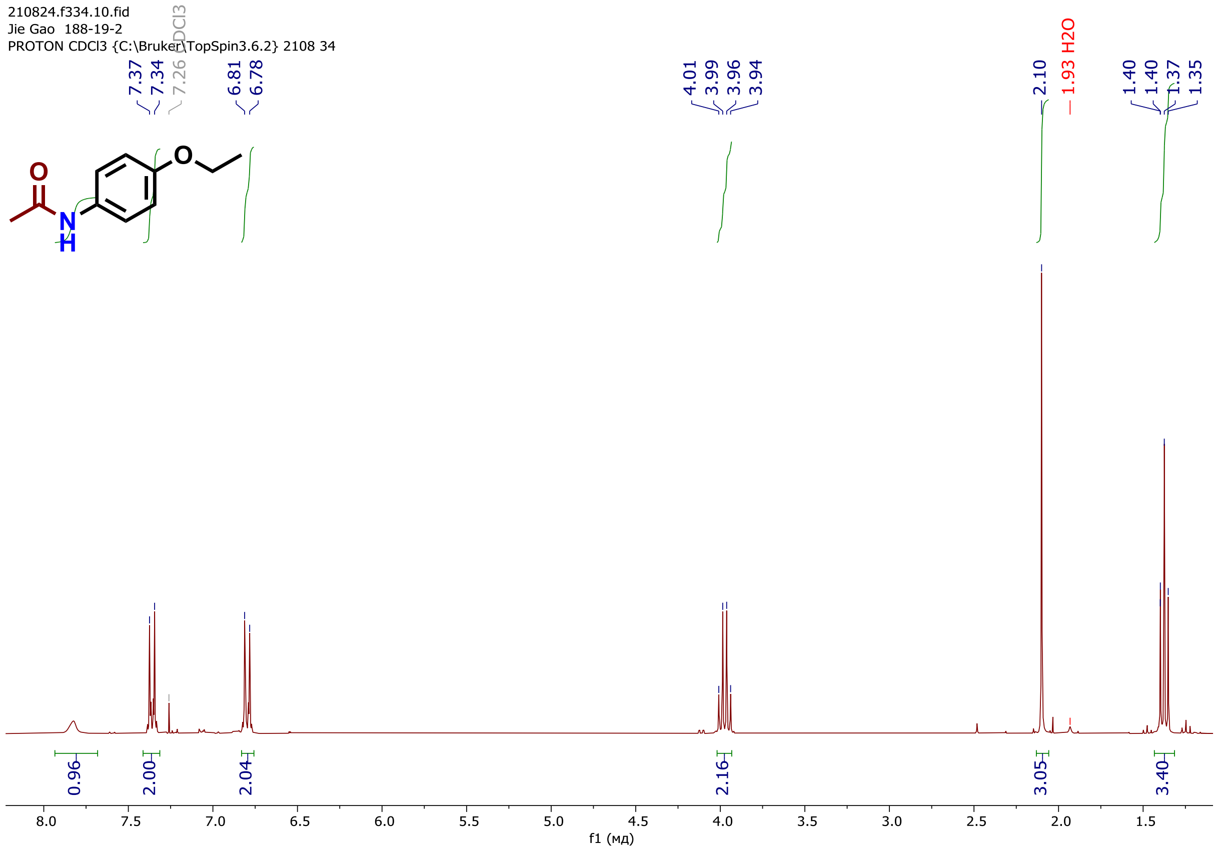

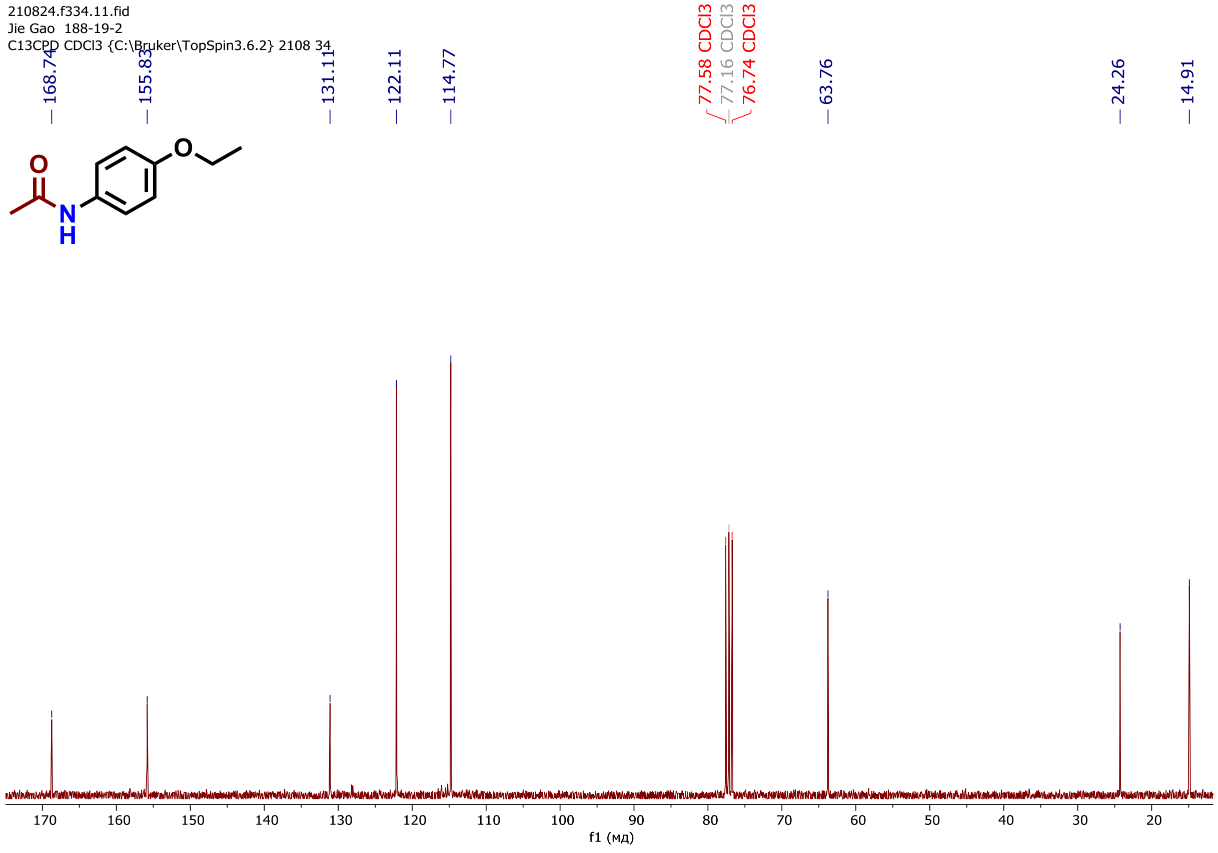

Supplementary Figure 79. NMR spectra of Phenacetin.

Propanil (58)

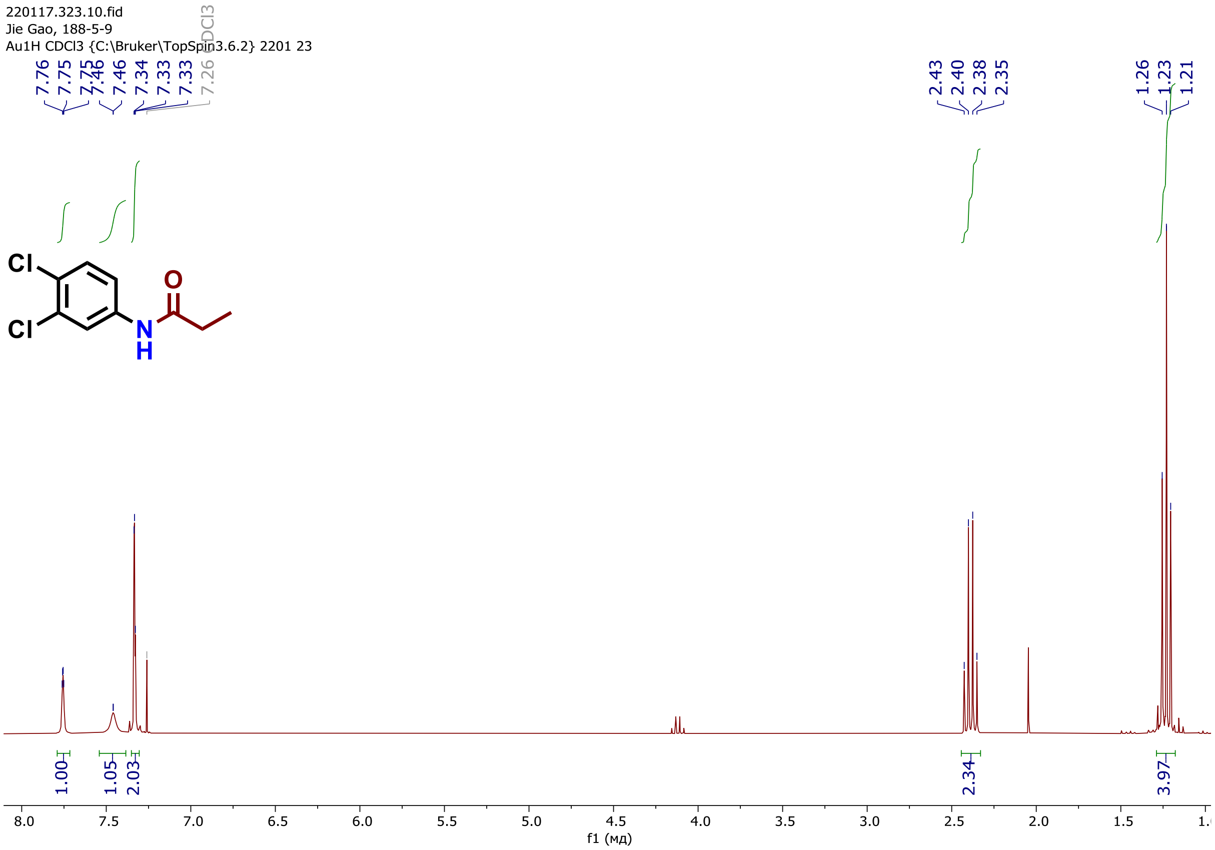

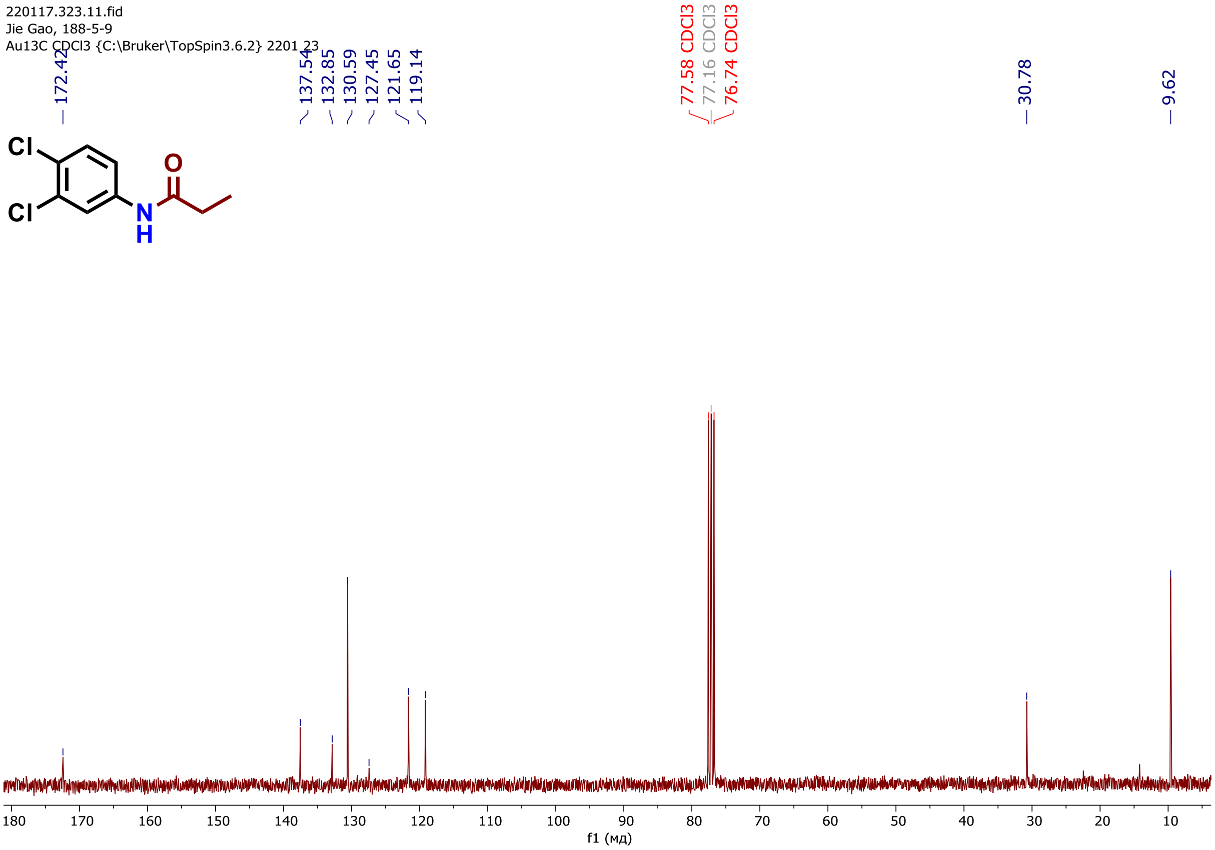

Supplementary Figure 80. NMR spectra of Propanil.

N-(4-acetylphenyl)-2-(4-chloro-2-methylphenoxy)acetamide (59)

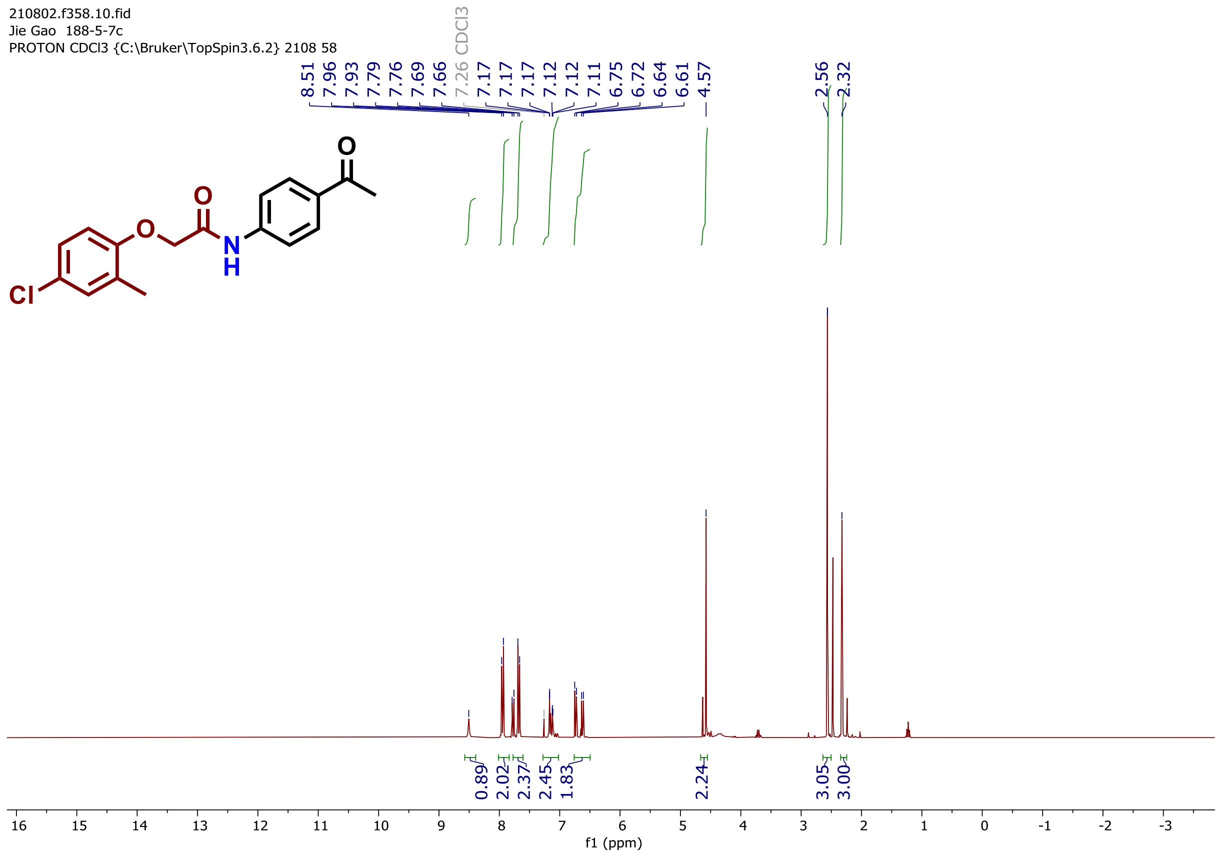

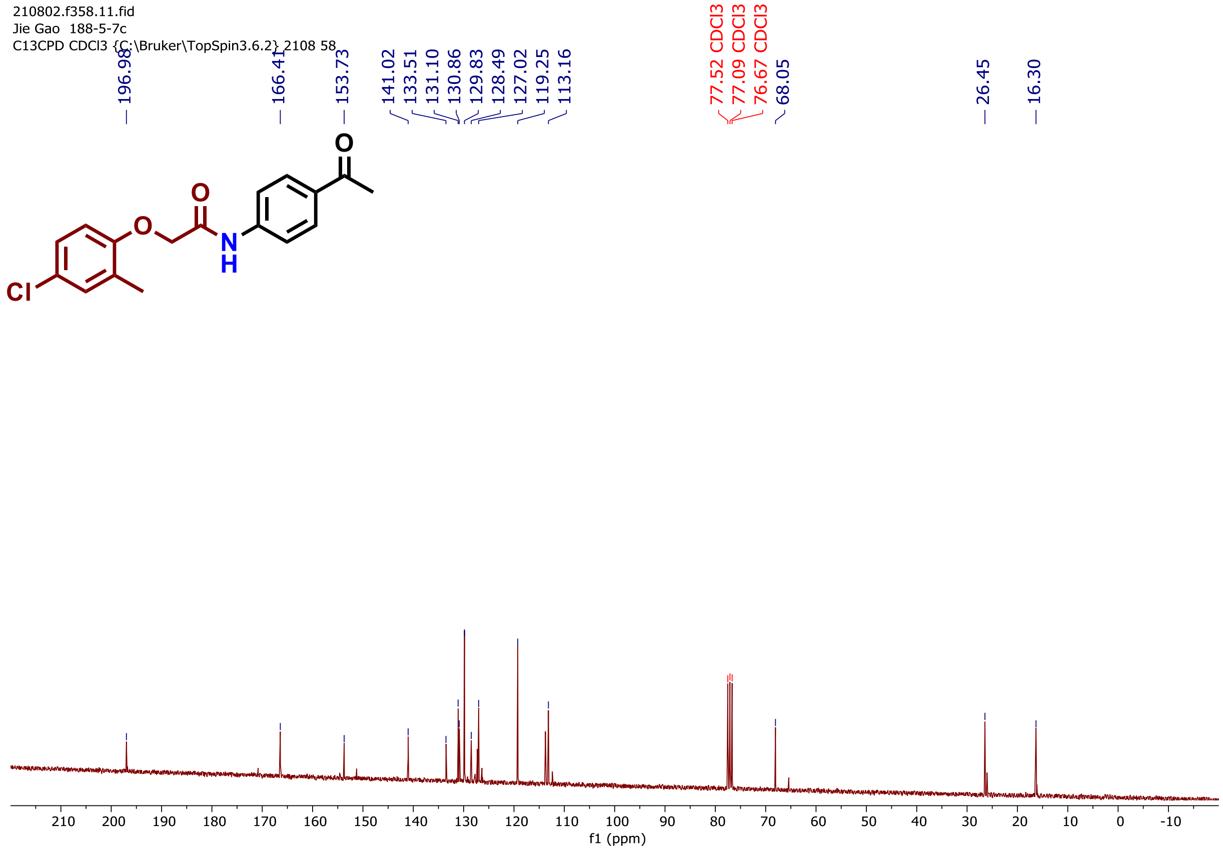

Supplementary Figure 81. NMR spectra of N-(4-acetylphenyl)-2-(4-chloro-2-methylphenoxy) acetamide.

N-(4-(3',6'-bis(diethylamino)-3-oxospiro[isoindoline-1,9'-xanthen]-2-yl)phenyl)acetamide (60)

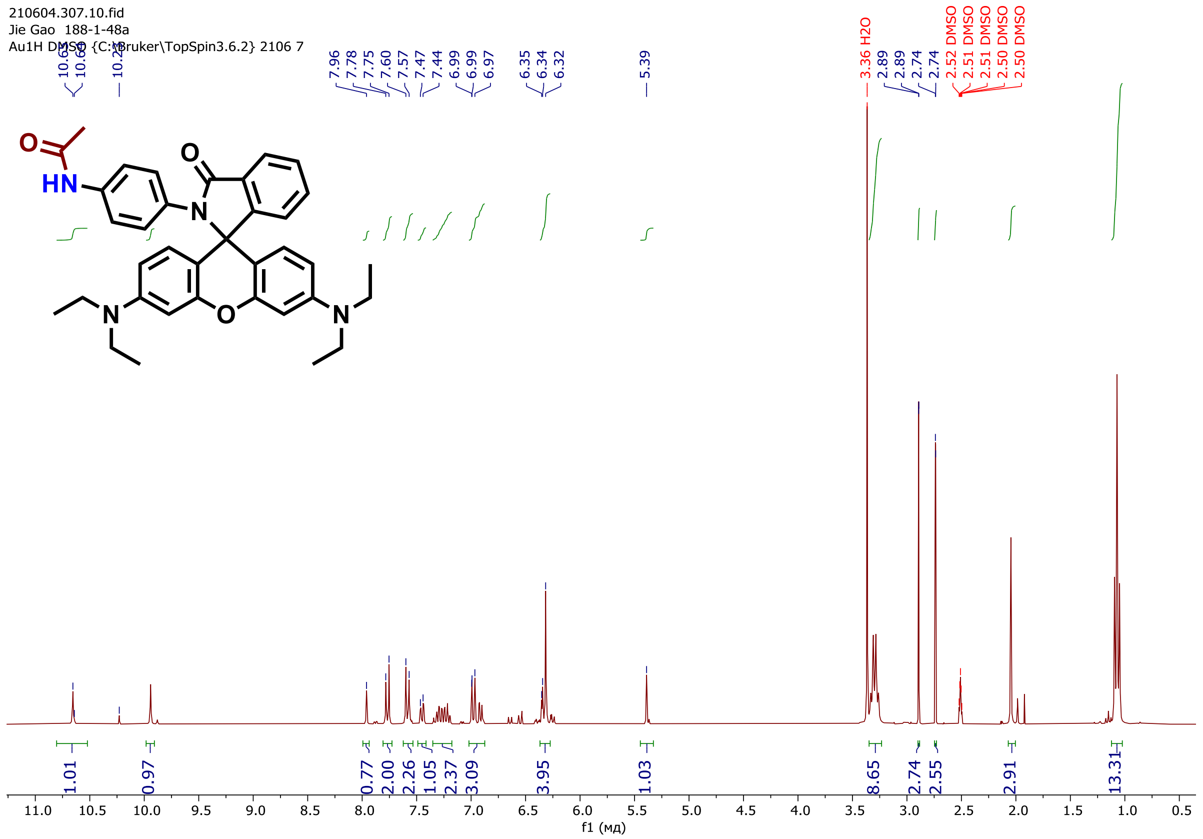

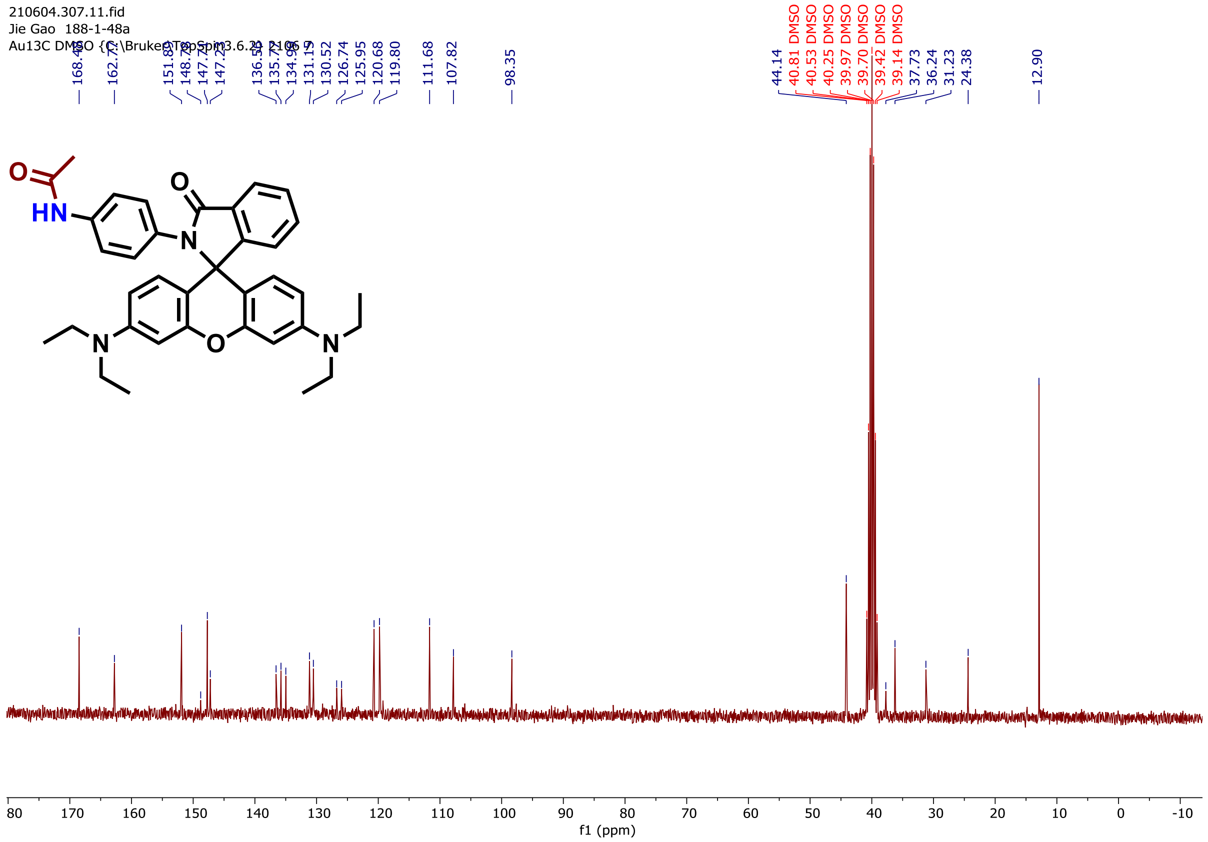

Supplementary Figure 82. NMR spectra of N-(4-(3',6'-bis(diethylamino)-3-oxospiro[isoindoline-1,9'-xanthen]-2-yl)phenyl)acetamide.

N-(4-(methylsulfonamido)-3-phenoxyphenyl)acetamide (61)

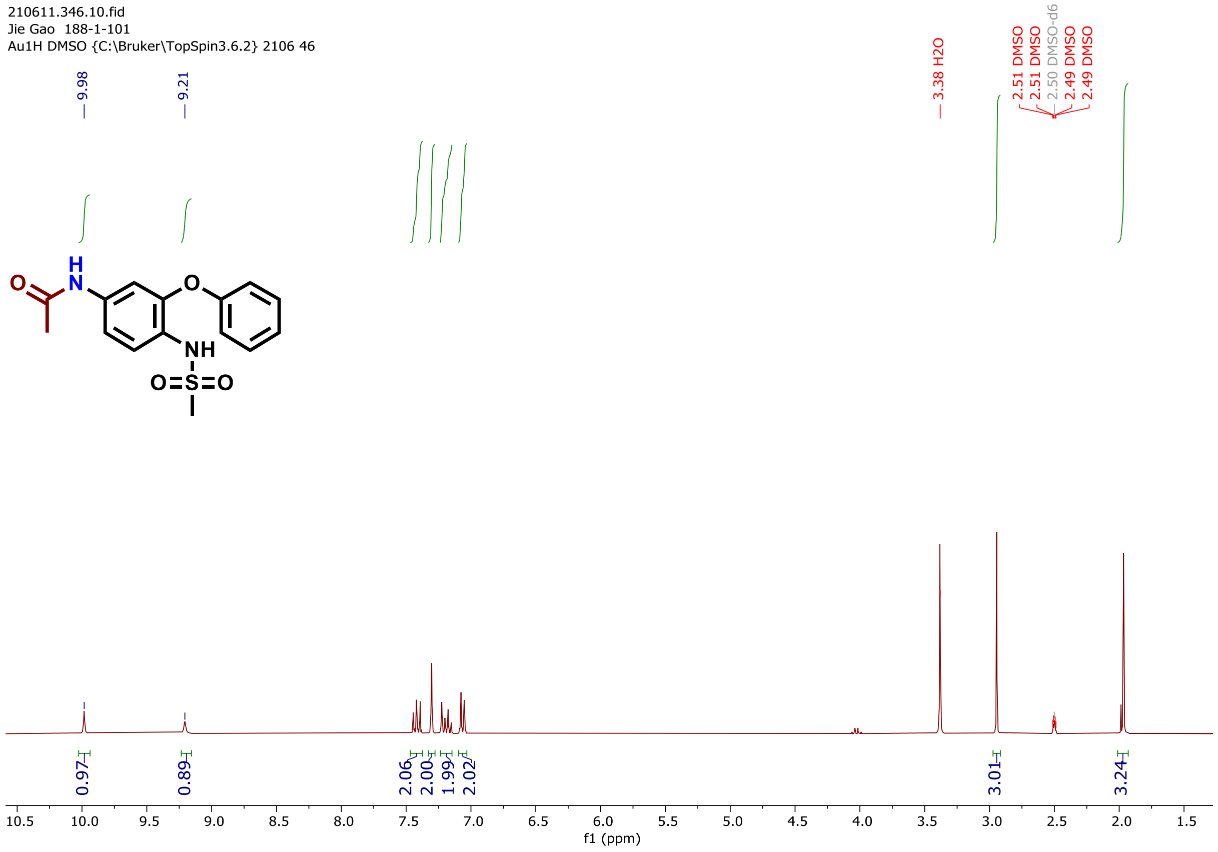

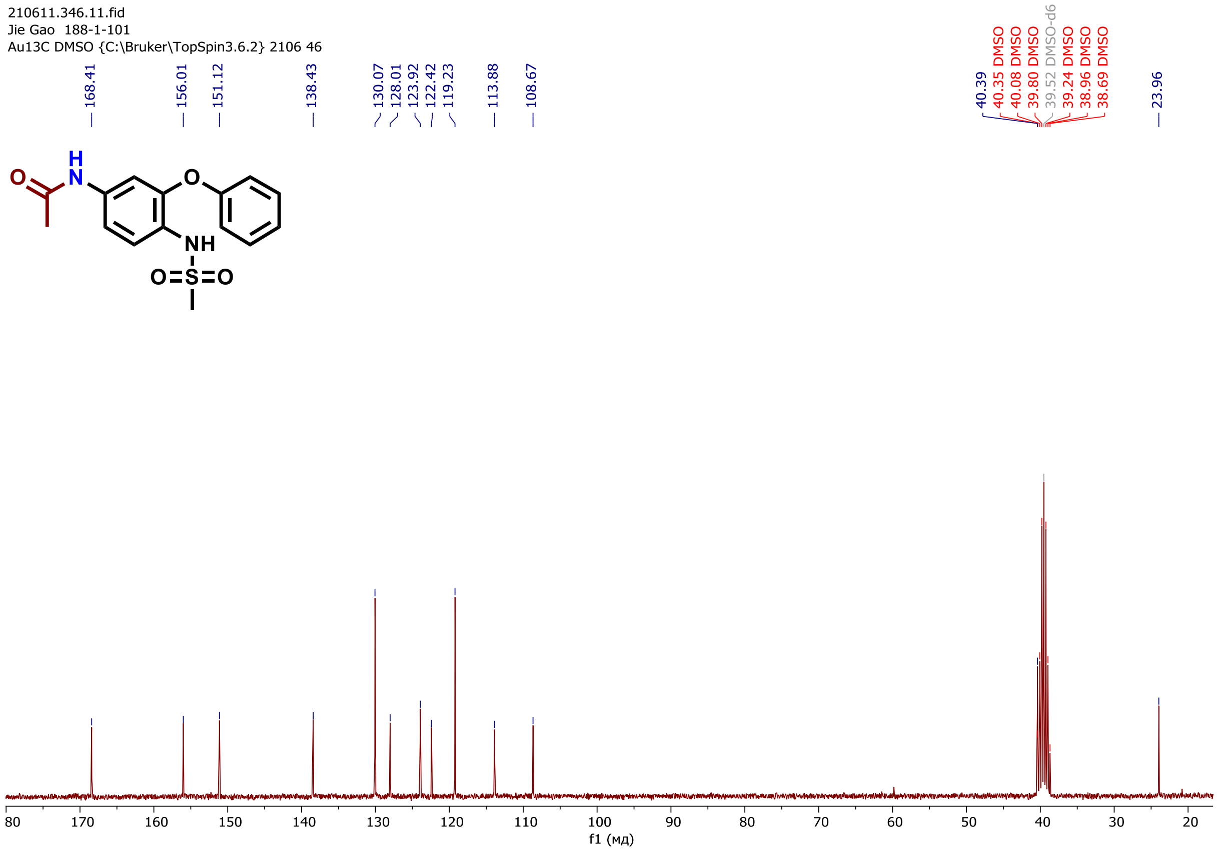

Supplementary Figure 83. NMR spectra of N-(4-(methylsulfonamido)-3-phenoxyphenyl)acetamide.

3-isopropyl 5-(2-methoxyethyl) 4-(3-acetamidophenyl)-2,6-dimethyl-1,4-dihydropyridine-3,5-dicarboxylate (62)

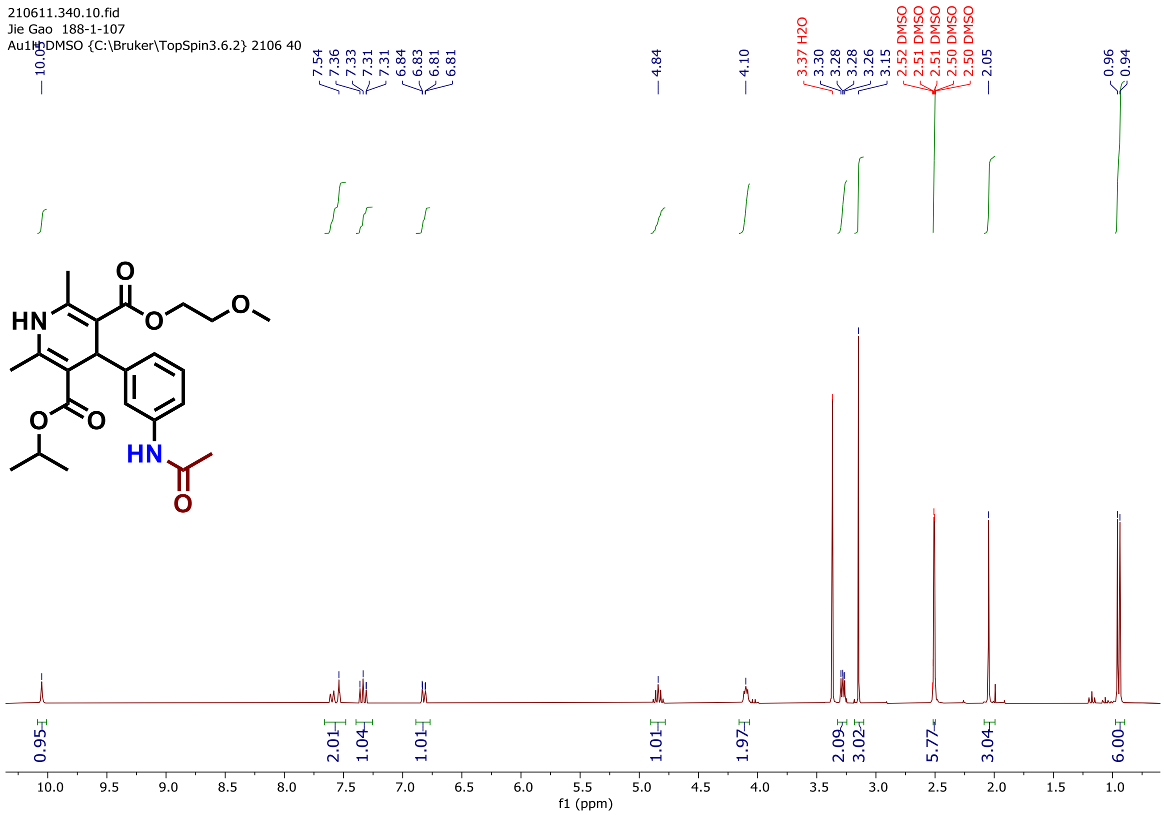

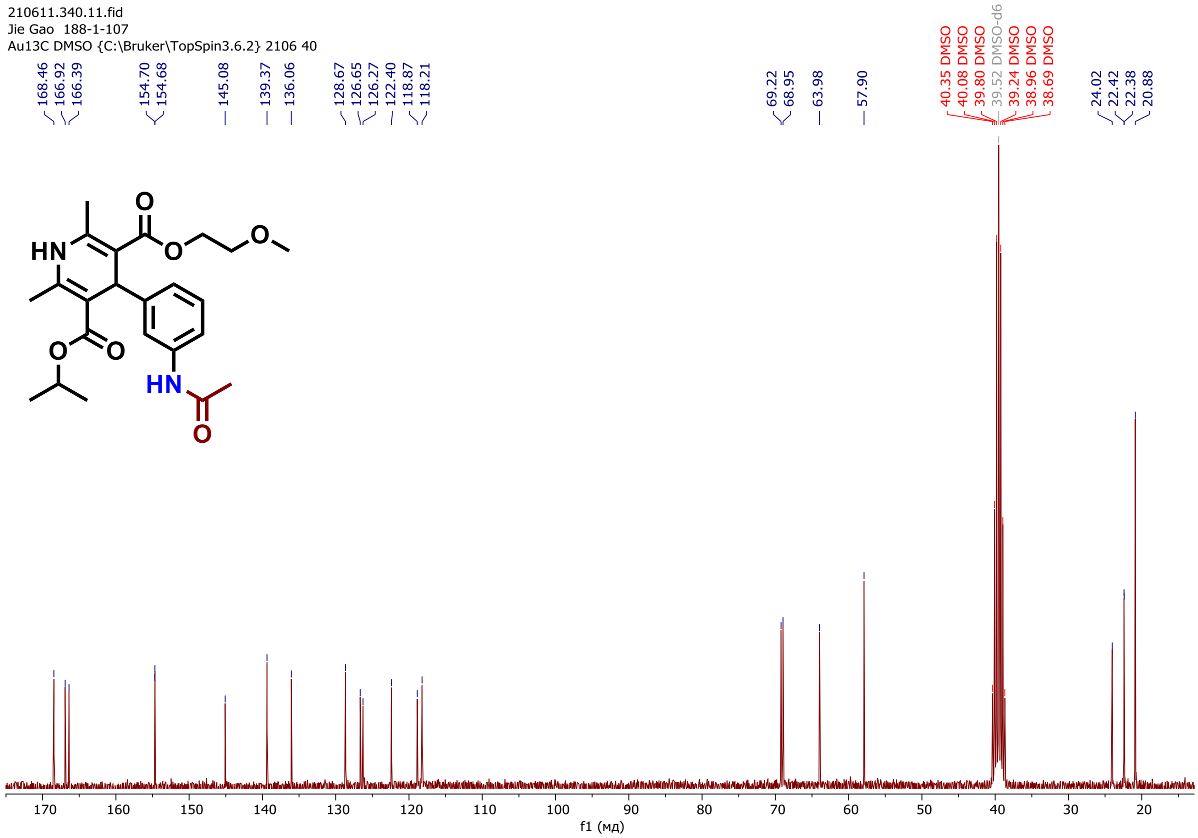

Supplementary Figure 84. NMR spectra of 3-isopropyl 5-(2-methoxyethyl) 4-(3-acetamidophenyl)-2,6-dimethyl-1,4-dihydropyridine-3,5-dicarboxylate.

3-ethyl 5-methyl 2-((2-acetamidoethoxy)methyl)-4-(2-chlorophenyl)-6-methyl-1,4-dihydropyridine-3,5-dicarboxylate (63)

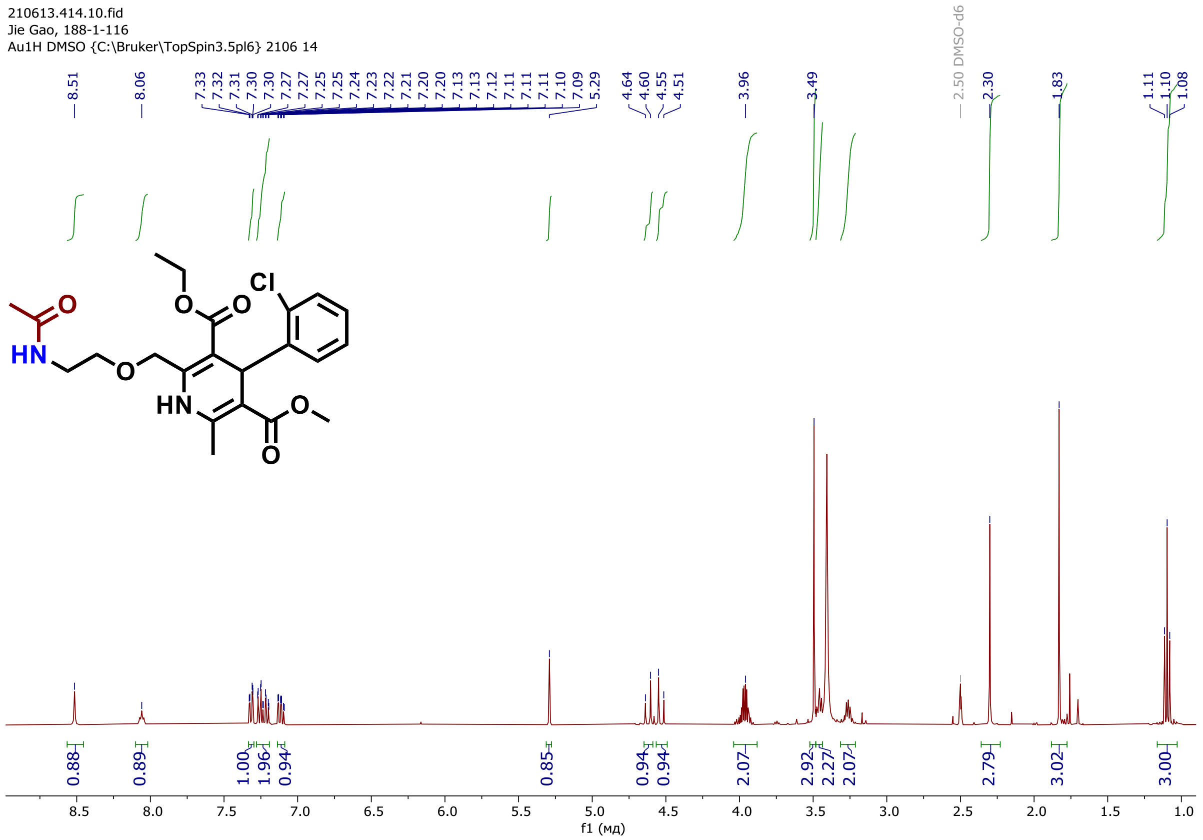

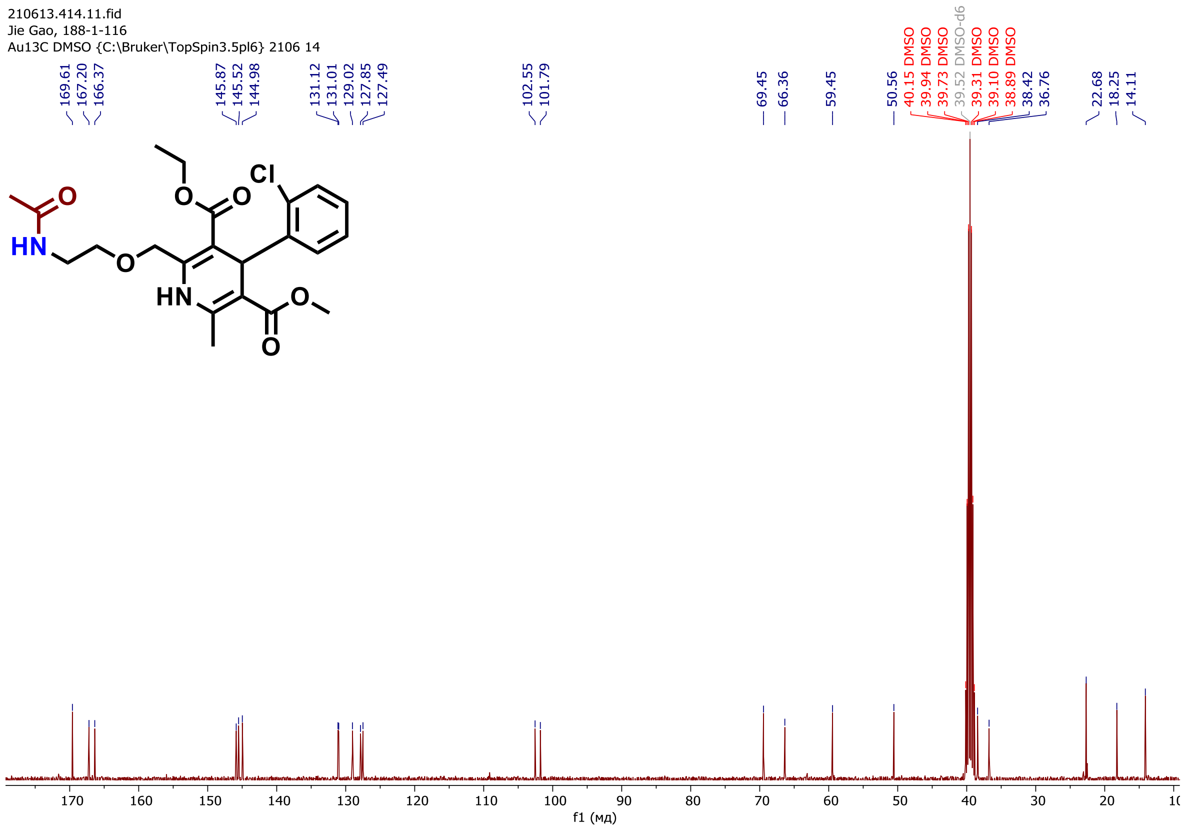

Supplementary Figure 85. NMR spectra of 3-ethyl 5-methyl 2-((2-acetamidoethoxy)methyl)-4-(2-chlorophenyl)-6-methyl-1,4-dihydropyridine-3,5-dicarboxylate.

3-ethyl 5-methyl 4-(2-chlorophenyl)-2-((2-(cyclobutanecarboxamido)ethoxy)methyl)-6-methyl-1,4-dihydropyridine-3,5-dicarboxylate (64)

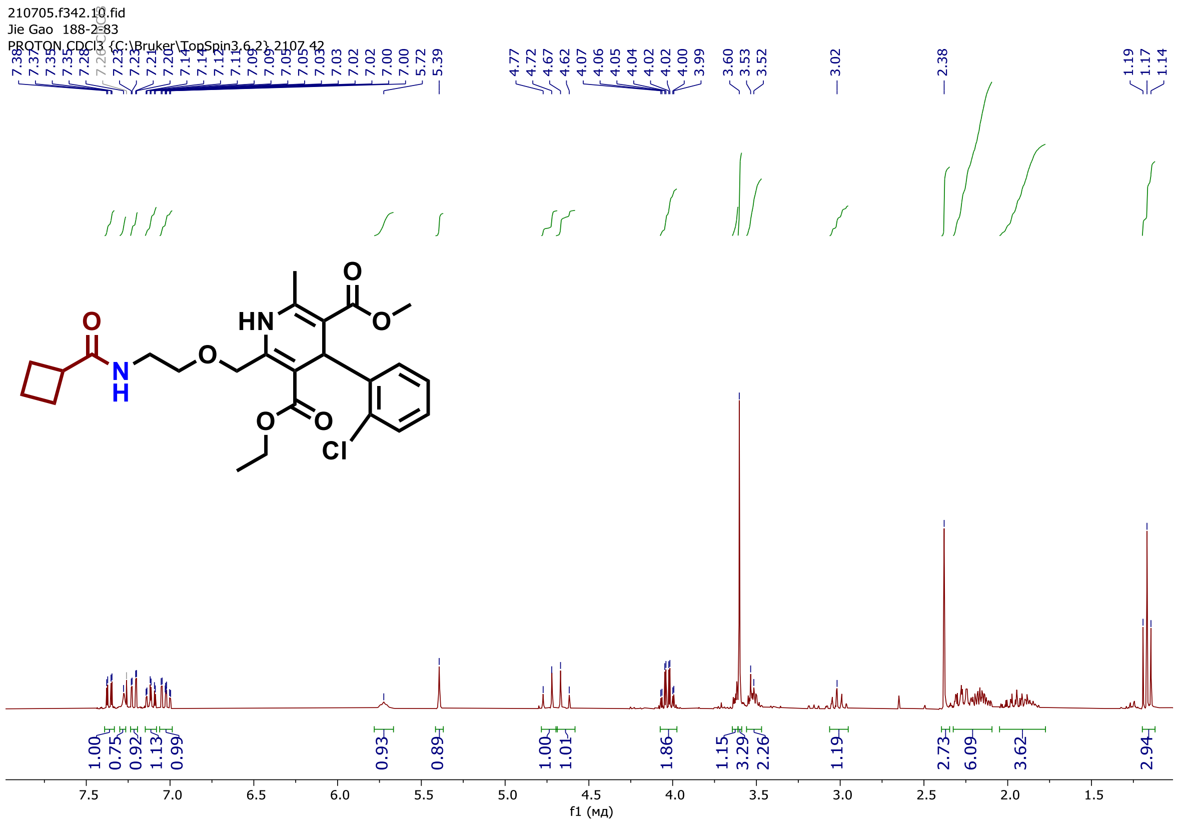

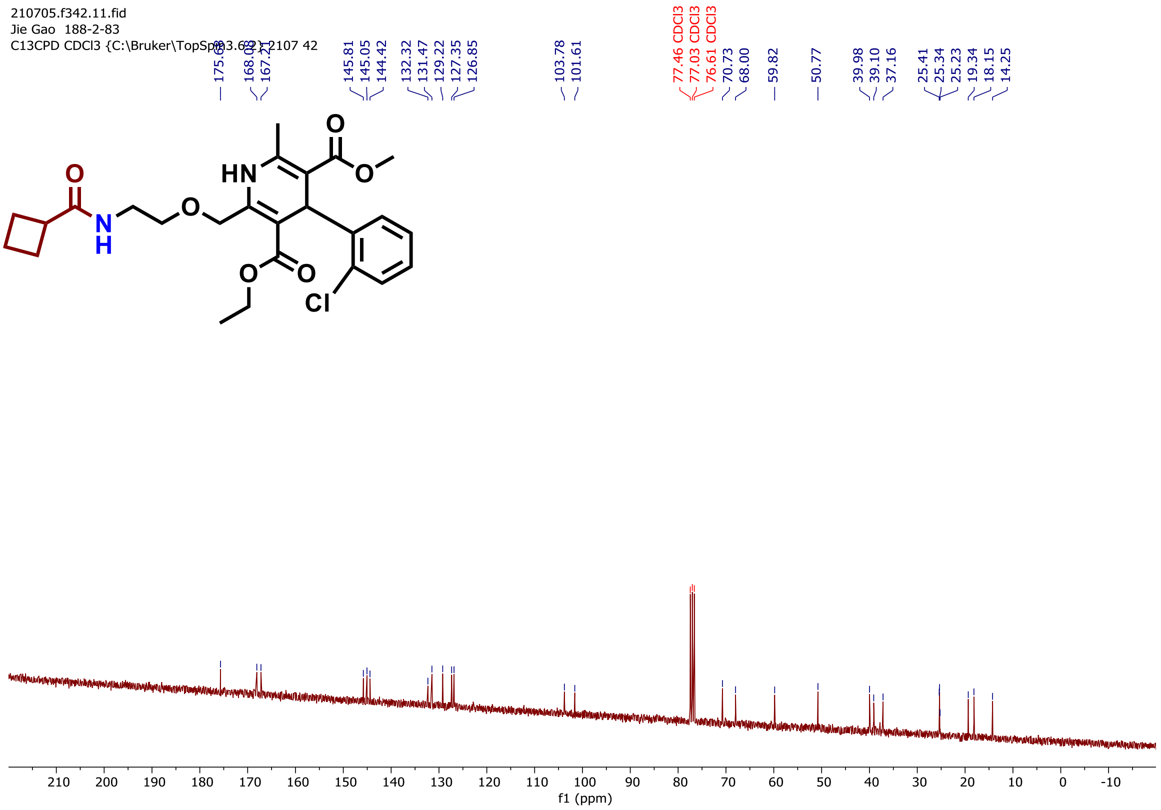

Supplementary Figure 86. NMR spectra of 3-ethyl 5-methyl 4-(2-chlorophenyl)-2-((2-(cyclobutanecarboxamido)ethoxy)methyl)-6-methyl-1,4-dihydropyridine-3,5-dicarboxylate.

5. HRMS Data.

5-acetamido-2-chlorobenzoic acid (25)

ESI-TOF Accurate Mass Report Page 1

Results file: E:\Projects\2206.PRO\SampleDB\2206.rpt
Last modified: Wednesday, June 22, 2022 09:47:12

Sample Summary:

Sample	File	Sample Name	User	Target	Formula	Expected Mass	Observed Mass	Error PPM	Error mDa
219	22062202	GJ-2-23	Jie Gao	213.0193	C9H8ClNO3	214.0271	214.0276	2.3	0.5

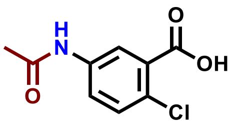

Supplementary Figure 87. HRMS data of 5-acetamido-2-chlorobenzoic acid

ethyl 3-(4-acetamidophenyl)propanoate (28)

ESI-TOF Accurate Mass Report Page 1

Results file: E:\Projects\2206.PRO\SampleDB\2206.rpt
Last modified: Thursday, June 09, 2022 12:25:52

Sample Summary:

Sample	File	Sample Name	User	Target	Formula	Expected Mass	Observed Mass	Error PPM	Error mDa
19	22060903	GJ-2-26	Jie Gao	235.1208	C13H17NO3	236.1286	236.1281	-2.1	-0.5
						258.1100	258.1105	1.9	0.5

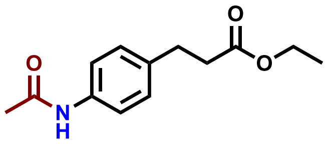

Supplementary Figure 88. HRMS data of ethyl 3-(4-acetamidophenyl)propanoate.

methyl 4-acetamidobenzoate (29)

ESI-TOF Accurate Mass Report

Page 1

Results file: E:\Projects\2206.PRO\SampleDB\2206.rpt
Last modified: Thursday, June 09, 2022 12:23:01

Sample Summary:

Sample	File	Sample Name	User	Target	Formula	Expected Mass	Observed Mass	Error PPM	Error mDa
20	22060904	GJ-2-27	Jie Gao	193.0739	C10H11NO3	194.0817	194.0819	1.0	0.2

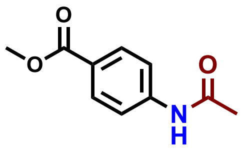

Supplementary Figure 89. HRMS data of methyl 4-acetamidobenzoate.

N-(2-(4-fluorophenoxy)pyridin-3-yl)acetamide (34)

Elemental Composition Report

Jie Gao 2-32

Mass	RA	Calc. Mass	mDa	PPM	Formula
247.0880	70.81	247.0883	-0.3	-1.2	C13 H12 N2 O2 F [M+H]$^+$

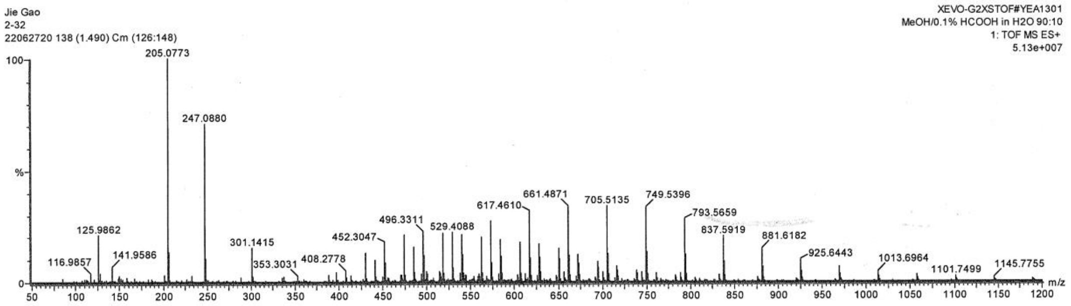

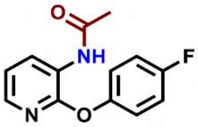

Supplementary Figure 90. HRMS data of N-(2-(4-fluorophenoxy)pyridin-3-yl)acetamide.

N-(benzo[d]thiazol-6-yl)acetamide (36)

ESI-TOF Accurate Mass Report

Page 1

Results file: E:\Projects\2206.PRO\SampleDB\2206.rpt
Last modified: Tuesday, June 21, 2022 17:21:13

Sample Summary:

Sample	File	Sample Name	User	Target	Formula	Expected Mass	Observed Mass	Error PPM	Error mDa
213	22062110	GJ-2-34	Jie Gao	192.0357	C9H8N2OS	193.0435	193.0439	2.1	0.4

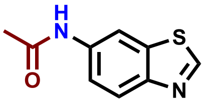

Supplementary Figure 91. HRMS data of N-(benzo[d]thiazol-6-yl)acetamide.

3-mercapto-N-phenylpropanamide (40)

Elemental Composition Report

Jie Gao / GJ-2-38

Mass	Calc. Mass	mDa	PPM	Formula	
182.0644	182.0640	0.4	2.2	C9 H11 N O S	$(M+H)^+$

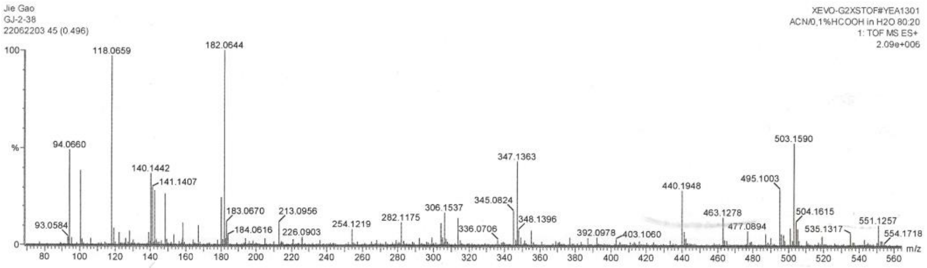

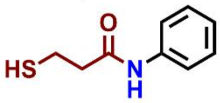

Supplementary Figure 92. HRMS data of 3-mercapto-N-phenylpropanamide.

2-cyano-N,2-diphenylacetamide (47)

ESI-TOF Accurate Mass Report Page 1

Results file: E:\Projects\2206.PRO\SampleDB\2206.rpt
Last modified: Thursday, June 09, 2022 12:52:39

Sample Summary:

Sample	File	Sample Name	User	Target	Formula	Expected Mass	Observed Mass	Error PPM	Error mDa
21	22060905	GJ-2-45	Jie Gao	236.0950	C15H12N2O	237.1028	237.1029	0.4	0.1

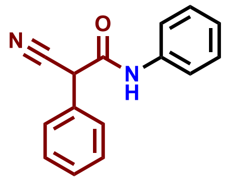

Supplementary Figure 93. HRMS data of 2-cyano-N,2-diphenylacetamide.

dimethyl 3,3'-(1,4-phenylenebis(azanediyl))bis(3-oxopropanoate) (48)

ESI-TOF Accurate Mass Report Page 1

Results file: E:\Projects\2206.PRO\SampleDB\2206.rpt
Last modified: Friday, June 10, 2022 09:32:10

Sample Summary:

Sample	File	Sample Name	User	Target	Formula	Expected Mass	Observed Mass	Error PPM	Error mDa
22	22060906	GJ-2-46	Jie Gao	308.1008	C14H16N2O6	309.1086	309.1091	1.6	0.5

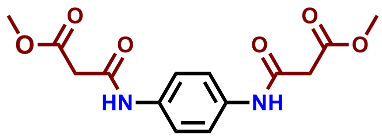

Supplementary Figure 94. HRMS data of dimethyl 3,3'-(1,4-phenylenebis(azanediyl)) bis(3-oxopropanoate)

methyl 5-(phenylcarbamoyl)furan-2-carboxylate (54)

ESI-TOF Accurate Mass Report

Page 1

Results file: E:\Projects\2206.PRO\SampleDB\2206.rpt
Last modified: Thursday, June 09, 2022 12:04:28

Sample Summary:

Sample	File	Sample Name	User	Target	Formula	Expected Mass	Observed Mass	Error PPM	Error mDa
23	22060907	GJ-2-52	Jie Gao	245.0688	C13H11NO4	246.0766	246.0770	1.6	0.4
						268.0580	268.0587	2.6	0.7

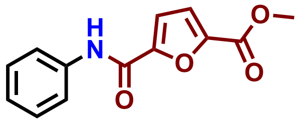

Supplementary Figure 95. HRMS data of methyl 5-(phenylcarbamoyl)furan-2-carboxylate.

N-(4-acetylphenyl)-2-(4-chloro-2-methylphenoxy)acetamide (59)

ESI-TOF Accurate Mass Report Page 1

Results file: E:\Projects\2206.PRO\SampleDB\2206.rpt
Last modified: Thursday, June 09, 2022 11:59:45

Sample Summary:

Sample	File	Sample Name	User	Target	Formula	Expected Mass	Observed Mass	Error PPM	Error mDa
24	22060908	GJ-2-57	Jie Gao	317.0819	C17H16ClNO3	318.0897	318.0903	1.9	0.6

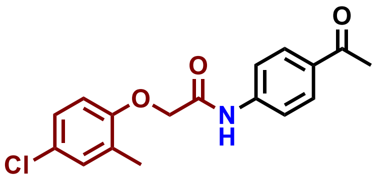

Supplementary Figure 96. HRMS data of N-(4-acetylphenyl)-2-(4-chloro-2-methylphenoxy)acetamide

N-(4-(methylsulfonamido)-3-phenoxyphenyl)acetamide (60)

ESI-TOF Accurate Mass Report Page 1

Results file: E:\Projects\2206.PRO\SampleDB\2206.rpt
Last modified: Thursday, June 09, 2022 11:58:32

Sample Summary:

Sample	File	Sample Name	User	Target	Formula	Expected Mass	Observed Mass	Error PPM	Error mDa
25	22060909	GJ-2-59	Jie Gao	574.2944	C36H38N4O3	575.3022<br>597.2836	575.3030<br>597.2850	1.4<br>2.3	0.8<br>1.4

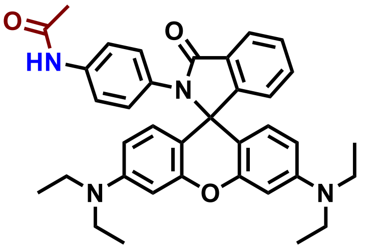

Supplementary Figure 97. HRMS data of N-(4-(methylsulfonamido)-3-phenoxyphenyl)acetamide.

N-(4-(methylsulfonamido)-3-phenoxyphenyl)acetamide (61)

ESI-TOF Accurate Mass Report Page 1

Results file: E:\Projects\2206.PRO\SampleDB\2206.rpt
Last modified: Thursday, June 09, 2022 11:56:40

Sample Summary:

Sample	File	Sample Name	User	Target	Formula	Expected Mass	Observed Mass	Error PPM	Error mDa
26	22060910	GJ-2-60	Jie Gao	320.0831	C15H16N2O4S	321.0909	321.0912	0.9	0.3
						343.0723	343.0730	2.0	0.7

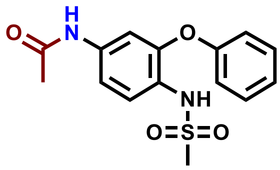

Supplementary Figure 98. HRMS data of N-(4-(methylsulfonamido)-3-phenoxyphenyl)acetamide.

3-isopropyl 5-(2-methoxyethyl) 4-(3-acetamidophenyl)-2,6-dimethyl-1,4-dihydropyridine-3,5-dicarboxylate (62)

ESI-TOF Accurate Mass Report Page 1

Results file: E:\Projects\2206.PRO\SampleDB\2206.rpt
Last modified: Tuesday, June 21, 2022 17:26:53

Sample Summary:

Sample	File	Sample Name	User	Target	Formula	Expected Mass	Observed Mass	Error PPM	Error mDa
215	22062112	GJ-2-61	Jie Gao	430.2104	C23H30N2O6	431.2182<br>453.1996	431.2175<br>453.2004	-1.6<br>1.8	-0.7<br>0.8

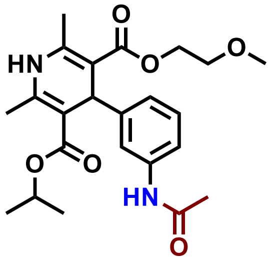

Supplementary Figure 99. HRMS data of 3-isopropyl 5-(2-methoxyethyl) 4-(3-acetamidophenyl) -2,6-dimethyl-1,4-dihydropyridine-3,5-dicarboxylate.

3-ethyl 5-methyl 4-(2-chlorophenyl)-2-((2-(cyclobutanecarboxamido)ethoxy)methyl)-6-methyl-1,4-dihydropyridine-3,5-dicarboxylate (64)

ESI-TOF Accurate Mass Report Page 1

Results file: E:\Projects\2206.PRO\SampleDB\2206.rpt
Last modified: Tuesday, June 21, 2022 17:13:21

Sample Summary:

Sample	File	Sample Name	User	Target	Formula	Expected Mass	Observed Mass	Error PPM	Error mDa
216	22062113	GJ-2-62	Jie Gao	490.1871	C25H31ClN2O6	491.1949<br>513.1763	491.1953<br>513.1777	0.8<br>2.7	0.4<br>1.4

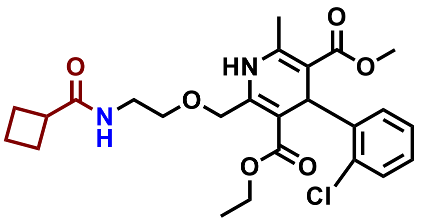

Supplementary Figure 100. HRMS data of 3-ethyl 5-methyl 4-(2-chlorophenyl)-2-((2-(cyclobutanecarboxamido)ethoxy)methyl)-6-methyl-1,4-dihydropyridine-3,5-dicarboxylate.

6. References.

1. Clark SJ, *et al*. First principles methods using CASTEP. Z. Kristallogr **220**, 567–570 (2005).

2. Perdew JP, Burke K, Ernzerhof M. Phys. Rev. Lett. **77**, 18 (1996).

3. Vanderbilt D. Soft self-consistent pseudopotentials in a generalized eigenvalue formalism. *Phys Rev B Condens Matter* **41**, 7892-7895 (1990).

4. Grimme S. Semiempirical GGA-type density functional constructed with a long-range dispersion correction. *J Comput Chem* **27**, 1787-1799 (2006).

5. Mehta M, *et al.* Hydrogen treated anatase TiO_2: a new experimental approach and further insights from theory. *Journal of Materials Chemistry A* **4**, 2670-2681 (2016).

6. Hirshfeld FL. Bonded-Atom Fragments for Describing Molecular Charge Densities. Theoret. Claim.Acta (Berl.) **44**, 129-138 (1977).

7. Kreikemeyer-Lorenzo D, Unterberger W, Duncan DA, Lerotholi TJ, Woodruff DP. The local structure of the azobenzene/aniline reaction intermediate on TiO_2(110). *Surface Science* **613**, 40-47 (2013).

8. Drillaud N, Banaszak-Leonard E, Pezron I, Len C. Synthesis and evaluation of a photochromic surfactant for organic reactions in aqueous media. *J Org Chem* **77**, 9553-9561 (2012).

9. Liu W, Li J, Querard P, Li CJ. Transition-Metal-Free C-C, C-O, and C-N Cross-Couplings Enabled by Light. *J Am Chem Soc* **141**, 6755-6764 (2019).

10. Zhang G, Zhao Y, Xuan L, Ding C. SO_2F_2-Activated Efficient Beckmann Rearrangement of Ketoximes for Accessing Amides and Lactams. *European Journal of Organic Chemistry* **2019**, 4911-4915 (2019).

11. Bell NL, *et al.* Cu(OTf)$_2$ -Mediated Cross-Coupling of Nitriles and N-Heterocycles with Arylboronic Acids to Generate Nitrilium and Pyridinium Products*. *Angew Chem Int Ed Engl* **60**, 7935-7940 (2021).

12. Namba T, *et al.* Atropisomeric Properties of N-Acyl/N-Sulfonyl 5H-Dibenzo[b,d]azepin-7(6H)-ones. *J Org Chem* **86**, 7563-7578 (2021).

13. Nicoletti M, Lorio MA. ^{1}H and ^{13}C NMR studies on 2-aminofluorene derivatives with carcinogenic and mutagenic activities. *Reson M. Chem* **24**, 221-224 (1986).

14. Breising VM, Kayser JM, Kehl A, Schollmeyer D, Liermann JC, Waldvogel SR. Electrochemical formation of N,N'-diarylhydrazines by dehydrogenative N-N homocoupling reaction. *Chem Commun* **56**, 4348-4351 (2020).

15. Nakajima M, Miyamoto K, Hirano K, Uchiyama M. Diaryl-lambda(3)-chloranes: Versatile Synthesis and Unique Reactivity as Aryl Cation Equivalent. *J Am Chem Soc* **141**, 6499-6503 (2019).

16. Tilden JAR, Lubben AT, Reeksting SB, Kociok-Kohn G, Frost CG. Pd(II)-Mediated C-H Activation for Cysteine Bioconjugation. *Chemistry* **28**, e202104385 (2022).

17. Bao ZP, Miao RG, Qi X, Wu XF. A novel construction of acetamides from rhodium-catalyzed aminocarbonylation of DMC with nitro compounds. *Chem Commun* **57**, 1955-1958 (2021).

18. Shi F, SmithIII MR, Maleczka RE. Aromatic Borylation/Amidation/Oxidation: A Rapid Route to 5-Substituted 3-Amidophenols. *Org. Lett.* **8**, 1411–1414 (2006).

19. Saeedian Moghadam E, *et al.* Benzimidazole derivatives act as dual urease inhibitor and anti-helicobacter pylori agent; synthesis, bioactivity, and molecular docking study. *Synthetic Communications* **52**, 936-948 (2022).

20. Qin C, Feng P, Ou Y, Shen T, Wang T, Jiao N. Selective C_{sp2}-C_{sp} bond cleavage: the nitrogenation of alkynes to amides. *Angew Chem Int Ed Engl* **52**, 7850-7854 (2013).

21. Liang Y, Lin F, Adeli Y, Jin R, Jiao N. Efficient Electrocatalysis for the Preparation of (Hetero)aryl Chlorides and Vinyl Chloride with 1,2-Dichloroethane. *Angew Chem Int Ed Engl* **58**, 4566-4570 (2019).

22. Nawrat CC, Lewis W, Moody CJ. Synthesis of amino-1,4-benzoquinones and their use in Diels-Alder approaches to the aminonaphthoquinone antibiotics. *J Org Chem* **76**, 7872-7881 (2011).

23. Zhu Y, Shi J, Yu W. Photoinduced Site-Selective C(sp^3)-H Chlorination of Aliphatic Amides. *Org Lett* **22**, 8899-8903 (2020).

24. Gao Y, *et al.* Dichloroimidazolidinedione-Activated Beckmann Rearrangement of Ketoximes for Accessing Amides and Lactams. *J Org Chem* **83**, 2040-2049 (2018).

25. Ghosh S, Purkait A, Jana CK. Environmentally benign decarboxylative N-, O-, and S-acetylations and acylations. *Green Chemistry* **22**, 8721-8727 (2020).

26. Artault M, Vitse K, Martin-Mingot A, Thibaudeau S. Direct Superacid-Promoted Difluoroethylation of Aromatics. *Chemistry* **28**, e202103926 (2022).

27. Sahoo B, *et al.* Site-Selective, Remote sp^3 C-H Carboxylation Enabled by the Merger of Photoredox and Nickel Catalysis. *Chemistry* **25**, 9001-9005 (2019).

28. Wippert NA, Jung N, Brase S. Synthesis of Arylamides via Ritter-Type Cleavage of Solid-Supported Aryltriazenes. *ACS Comb Sci* **21**, 568-572 (2019).

29. Mudshinge SR, Potnis CS, Xu B, Hammond GB. HCl*DMPU-Assisted One-pot and Metal-free Conversion of Aldehydes to Nitriles. *Green Chem* **22**, 4161-4164 (2020).

30. Cervi A, Vo Y, Chai CLL, Banwell MG, Lan P, Willis AC. Gold(I)-Catalyzed Intramolecular Hydroarylation of Phenol-Derived Propiolates and Certain Related Ethers as a Route to Selectively Functionalized Coumarins and 2*H*-Chromenes. *J Org Chem* **86**, 178-198 (2021).

31. Zhu L, *et al.* Copper-Mediated Remote C-H Bond Chalcogenation of Quinolines on the C5 Position. *Org Lett* **17**, 5528-5531 (2015).

32. Kiely-Collins HJ, Sechi I, Brennan PE, McLaughlin MG. Mild, calcium catalysed Beckmann rearrangements. *Chem Commun* **54**, 654-657 (2018).

33. Gøgsig TM, Søbjerg LS, Lindhardt AT, Jensen KL, Skrydstrup T. Direct Vinylation and Difluorovinylation of Arylboronic Acids Using Vinyl- and 2,2-Difluorovinyl Tosylates via the Suzuki−Miyaura Cross Coupling. *J. Org. Chem*. **73**, 3404 – 3410 (2008).

34. Zhu YP, Sergeyev S, Franck P, Orru RV, Maes BU. Amine Activation: Synthesis of N-(Hetero)arylamides from Isothioureas and Carboxylic Acids. *Org Lett* **18**, 4602-4605 (2016).

35. Wang Y, Zhu D, Tang L, Wang S, Wang Z. Highly Efficient Amide Synthesis from Alcohols and Amines by Virtue of a Water-Soluble Gold/DNA Catalyst. *Angew Chem Int Ed Engl* **50**, 8917-8921 (2011).

36. Qu E, Li S, Bai J, Zheng Y, Li W. Nickel-Catalyzed Reductive Cross-Coupling of N-Acyl and N-Sulfonyl Benzotriazoles with Diverse Nitro Compounds: Rapid Access to Amides and Sulfonamides. *Org Lett* **24**, 58-63 (2022).

37. Liu X, Zhu Q, Chen D, Wang L, Jin L, Liu C. Aminoazanium of DABCO: An Amination Reagent for Alkyl and Aryl Pinacol Boronates. *Angew Chem Int Ed Engl* **59**, 2745-2749 (2020).

38. Liang X, *et al.* Identification of Novel Fused Heteroaromatics-Based MALT1 Inhibitors by High-Throughput Screening to Treat B Cell Lymphoma. *J Med Chem* **64**, 9217-9237 (2021).

39. Li D, Oku N, Shinozaki Y, Kurokawa Y, Igarashi Y. 4-Hydroxy-3-methyl-2(1*H*)-quinolone, originally discovered from a Brassicaceae plant, produced by a soil bacterium of the genus Burkholderia sp.: determination of a preferred tautomer and antioxidant activity. *Beilstein J Org Chem* **16**, 1489-1494 (2020).

40. Zhang LY, Zhang C, Wang T, Shi YL, Ban MT, Cui DM. Copper-Catalyzed Tandem Reactions of 2-Amine-[1,3,5]triazines with Nitriles. *J Org Chem* **84**, 536-543 (2019).

41. Wen Q, Jin J, Mei Y, Lu P, Wang Y. Copper-Mediated Cyanation of Aryl Halides by Activation of Benzyl Cyanideas the Cyanide Source. *European Journal of Organic Chemistry* **56**,4032-4036 (2013).

42. Chen C, Ling L, Luo M, Zeng X. Chromium-Catalyzed Ligand-Free Amidation of Esters with Anilines. *Bulletin of the Chemical Society of Japan* **94**, 762-766 (2021).

43. Fu Z, Wang X, Tao S, Bu Q, Wei D, Liu N. Manganese Catalyzed Direct Amidation of Esters with Amines. *J Org Chem* **86**, 2339-2358 (2021).

44. Lu H, Geng Z, Li J, Zou D, Wu Y, Wu Y. Metal-Free Reduction of Aromatic Nitro Compounds to Aromatic Amines with B_2pin_2 in Isopropanol. *Org Lett* **18**, 2774-2776 (2016).

45. Urgin K, *et al.* Pharmacomodulation of the Antimalarial Plasmodione: Synthesis of Biaryl- and N-Arylalkylamine Analogues, Antimalarial Activities and Physicochemical Properties. *Molecules* **22**, 161 (2017).

46. Zhao L, *et al.* Cyclic (Alkyl)(amino)carbene Ligand-Promoted Nitro Deoxygenative Hydroboration with Chromium Catalysis: Scope, Mechanism, and Applications. *J Am Chem Soc* **143**, 1618-1629 (2021).

47. Zhang G, Ji X, Yu H, Yang L, Jiao P, Huang H. Palladium-catalyzed hydroaminocarbonylation of alkenes with amines promoted by weak acid. *Tetrahedron Letters* **57**, 383-386 (2016).

48. Schiller IC, Jacobson KA, Wen Z, Malisetty A, Schmalzing G, Markwardt F. Dihydropyridines Potentiate ATP-Induced Currents Mediated by the Full-Length Human P2X5 Receptor. *Molecules* **27**, 1846 (2022).
